# Sarcopenia-related traits and 10 digestive system disorders: insight from genetic correlation and Mendelian randomization

**DOI:** 10.3389/fpubh.2024.1412842

**Published:** 2024-07-10

**Authors:** Tao Yang, Zheng Liu, Mingzhu Xiu, Xiaoman Qing, Sha Liu, Wanmeng Xiao, Muhan Lü

**Affiliations:** ^1^Department of Gastroenterology, The Affiliated Hospital of Southwest Medical University, Luzhou, China; ^2^The Affiliated Hospital of Southwest Medical University, Luzhou, China; ^3^Human Microecology and Precision Diagnosis and Treatment of Luzhou Key Laboratory, Luzhou, China

**Keywords:** sarcopenia, digestive system disorders, genetic correlation, Mendelian randomization, GWAS

## Abstract

**Introduction:**

Despite observational studies suggest hypotheses indicating a potential link, the precise causal connection between sarcopenia and digestive system illnesses has not been clearly defined.

**Methods:**

We first use Linkage Disequilibrium Score Regression (LDSC) testing to determine the genetic correlation of traits associated with sarcopenia and 10 specific gastrointestinal diseases. Subsequently, we performed a set of bidirectional Mendelian Randomization (MR) analyses to gauge the genetic inclination towards sarcopenia-related traits in relation to each gastrointestinal condition, individually, across the FinnGen, UK Biobank, and other extensive collaborative consortia. The analytical outcomes were synthesized using a fixed-effects meta-analytic model. For outcomes indicating substantial causal impacts, mediation MR analyses were executed. Additionally, a battery of sensitivity analyses was conducted to evaluate the study’s strength and dependability.

**Results:**

Our findings established a strong causal link between appendicular lean mass and gastroesophageal reflux disease (OR  =  0.8607; 95% CI: 0.8345–0.8877; *p* <  0.0001) and a noteworthy correlation with nonalcoholic fatty liver disease (OR  =  0.7981; 95% CI: 0.7281–0.8749; *p* < 0.0001), as per the meta-analysis data. We also evaluated the intermediary role of metabolic disorders in the association between appendicular lean mass and the aforementioned diseases. The intermediary effect towards gastroesophageal reflux disease is quantified as 0.0087 (95% CI, 8e-04, 0.0183), accounting for 5.9398% (95% CI, 0.5462, 12.4940%) of the overall effect. For non-alcoholic fatty liver, the intermediary impact is 0.0150 (95% CI, 0.0050, 0.0270), representing 19.7808% (95% CI, 6.5936, 35.6055%) of the total effect.

**Conclusion:**

The findings posit that augmenting muscle mass may serve as a preventative strategy against gastroesophageal reflux disease and non-alcoholic fatty liver, highlighting the critical role of metabolic disorder management in reducing the risks of these sarcopenia-related conditions.

## 1 Introduction

Sarcopenia, a systemic and progressive condition affecting skeletal muscles, is characterized by a rapid decline in both muscle mass and strength (1). This condition is linked to numerous negative outcomes including increased risk of falls, reduced mobility, frailty, and mortality. Predominantly impacting the older demographic, it is a common issue among seniors, with estimates suggesting that it impacts between 10 and 16% of the older adult worldwide (2, 3). Sarcopenia can also develop in individuals during middle age and is associated with various health conditions. Although prior research in epidemiology has hinted at a possible link between sarcopenia and a range of gastrointestinal disorders, such as gastroesophageal reflux disease (GERD) (4), non-alcoholic fatty liver disease (NAFLD) (5) and gastrointestinal cancers (6), the existence of a causal relationship remains to be conclusively proven. This is largely due to the ongoing challenges posed by confounding factors and the risk of measurement errors.

In order to surmount these constraints, (7) devised a dependable technique for establishing causal connections between modifiable risk factors and health consequences. This approach utilizes genetic variants that are closely associated with the exposure in question as instrumental variables, a strategy known as Mendelian Randomization (MR). This technique has successfully navigated the common obstacles of confounding factors, the risk of reverse causality, and regression dilution bias, and it has become a favored method in the realm of observational epidemiology (8, 9).

To our knowledge, comprehensive MR studies that explore the causal links between sarcopenia and various gastrointestinal conditions, such as digestive tract tumors, have not yet been extensively undertaken. This study employs a bidirectional MR method to rigorously evaluate the causal relationship between sarcopenia and 10 distinct digestive system conditions, aiming to clarify the hypotheses from previous observational studies concerning their causal linkage. Elucidating the relationship between sarcopenia and digestive system diseases is crucial, as it could guide the development of preventive and treatment strategies. It is particularly important to determine if addressing sarcopenia could reduce the risk of gastrointestinal diseases, which could have a profound impact on the evolution of preventive medical approaches.

## 2 Materials and methods

### 2.1 Study design

**Figure 1:**
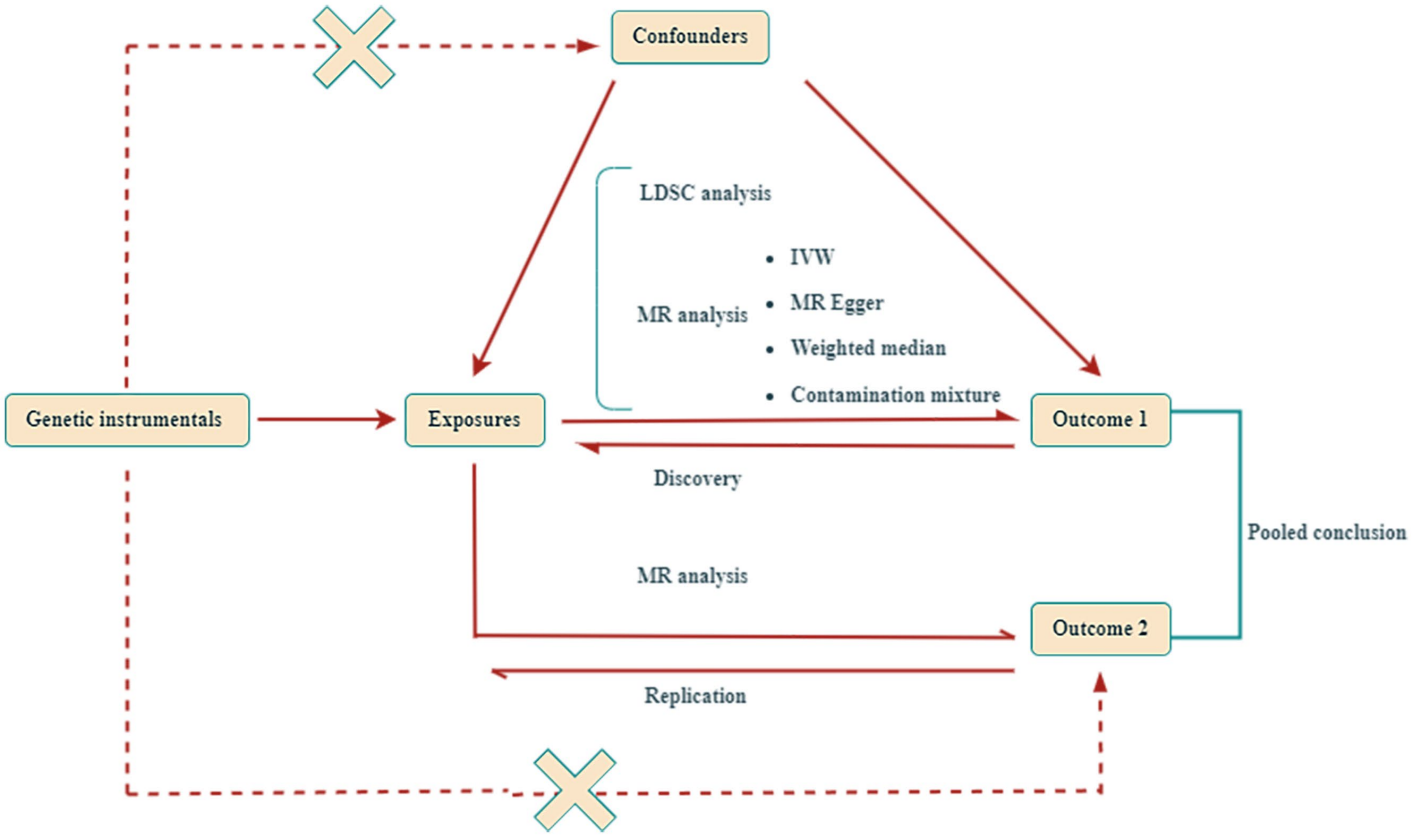
Design of this Mendelian randomization (MR) study. This study utilized two independent genome-wide association studies (GWAS) datasets for gastrointestinal diseases. LDSC, Linkage Disequilibrium Score; IVW, inverse-variance weighted.

As outlined in Figure 1, the design of our study commenced with the calculation of genetic correlations among traits associated with sarcopenia and 10 distinct gastrointestinal disorders. Following this, we carried out a series of bidirectional MR analyses to quantify the genetic predispositions to sarcopenia-related traits in relation to each gastrointestinal disease, separately within the FinnGen, UK Biobank, and additional large-scale collaborative groups. The results of these analyses were pooled using a fixed-effects meta-analytic model. Subsequently, for those outcomes demonstrating significant causal effects, we conducted mediation MR analyses. It is important to note that all research studies included herein had received approval from the appropriate ethics committees, and all participants had given their informed consent.

### 2.2 Data source for sarcopenia-related traits

Building on the knowledge from prior MR studies, we pinpointed appendicular lean mass (representing muscle mass), low grip strength (a measure of muscular power), and walking pace (indicative of muscle function) as critical sarcopenia metrics. For our analysis, we obtained instrumental variables for appendicular lean mass (ALM) from a Genome-Wide Association Study (GWAS) within the UK Biobank, which comprised 450,243 participants. ALM was quantified via bioelectrical impedance analysis (BIA), representing the total mass of the limbs excluding fat. We also identified instrumental variables for low grip strength from a GWAS meta-analysis involving 256,523 participants, with thresholds set at under 30 kg for men and under 20 kg for women. In addition, we sourced genetic instrumental variables for walking speed from GWAS data within the UK Biobank, which included 459,915 participants.

### 2.3 Data source for 10 digestive system disorders and metabolic disorders

Our study encompassed a comprehensive examination of ten distinct gastrointestinal disorders, categorized into four conditions affecting the upper gastrointestinal tract (GERD, esophageal cancer, gastric cancer, and gastroduodenal ulcer), one condition of the lower gastrointestinal tract (colorectal cancer), two pancreatic conditions (chronic pancreatitis and pancreatic cancer), and three liver-related diseases (NAFLD, liver cirrhosis, and liver cancer). The genetic associations at the summary level were obtained from studies conducted by the FinnGen consortium and the UK Biobank. Supplementary GWAS data were incorporated from multiple studies, such as those on gastroesophageal reflux disease by (10) (with 129,080 cases and 473,524 controls, as well as 20,381 cases and 464,217 controls), chronic pancreatitis (1,424 cases and 476,104 controls), esophageal cancer (98 cases and 475,308 controls), gastric cancer (1,029 cases and 475,087 controls), pancreatic cancer (1,196 cases and 475,049 controls), non-alcoholic fatty liver disease by (11) (8,434 cases and 770,180 controls), and liver cirrhosis (122 cases and 347,284 controls). Additionally, we considered metabolic disorders as possible mediators, and the pertinent findings are available through the FinnGen consortium. Supplementary Table S1 provides comprehensive details regarding the sources of the data presented.

### 2.4 Instruments selection

In our endeavor to ensure the accuracy and dependability of our dataset, we implemented a rigorous preprocessing protocol. We pinpointed independent Single Nucleotide Polymorphisms (SNPs) with a high degree of specificity (r2 < 0.001 and a clumping window of 10,000 kb) to act as instrumental variables for traits related to sarcopenia. These SNPs were chosen based on genome-wide significance (*p* < 5.0 × 10^-8) and a minimum minor allele frequency (MAF) of 0.01. We excluded palindromic SNPs from our set of instrumental variables and evaluated their suitability by calculating the *F*-statistic for each SNP linked to sarcopenia traits. The *F*-statistic was determined using the formula ((N − k − 1)/k) * (R2 /(1 − R2)), with N denoting the number of samples and k being the number of SNPs (12). SNPs with an *F*-statistic below 10 were excluded from our Mendelian Randomization (MR) analysis to reduce the likelihood of weak instrument bias. Conversely, for the reverse MR analysis, we applied a less stringent significance threshold of *p* < 5.0 × 10^-6 to detect SNPs associated with the ten gastrointestinal diseases, considering the scarcity of SNPs meeting the genome-wide significance threshold (*p* < 5.0 × 10^-8).

### 2.5 Statistical analysis

#### 2.5.1 Genetic correlation analysis

Employing the Linkage Disequilibrium Score (LDSC) test, we estimated the genetic correlation (rg) between traits associated with sarcopenia and a set of ten gastrointestinal disorders. The LDSC approach assesses the correlation by examining the relationship between test statistics and linkage disequilibrium, which helps to distinguish between genuine polygenic effects and potential biases (13, 14). This method entails the multiplication of z-scores from variants of one trait with those from another trait’s variants. The genetic covariance is derived by regressing this multiplied value against the LD score. This covariance, when divided by SNP heritability, provides an estimate of the genetic correlation. We established a *p* value threshold of *p* < 0.0019 (0.05/27, applying the stringent Bonferroni correction) to denote statistical significance (15). *p* values ranging from 0.002 to 0.05 were taken as suggestive of a possible genetic correlation.

#### 2.5.2 MR analysis

In our exploration of the causal relationships between sarcopenia-related traits and various digestive diseases, we deployed four distinct methodologies: inverse variance weighting (IVW), MR Egger, weighted median, and contamination mixture, with IVW serving as the primary analytical tool (16). Particularly, the contamination mixture method has proven its resilience and accuracy in MR analyses where instrumental variables may not be valid, showing the lowest mean square error among robust techniques in diverse real-world contexts (17). To ensure the credibility of our results, we measured the heterogeneity across different SNPs using Cochran’s Q statistic (18). In cases where substantial heterogeneity was identified in the MR analysis, we employed the IVW random effects model. Additionally, we availed ourselves of the MR-PRESSO tool to address any residual and outlier values that could indicate MR pleiotropy (19). For both scenarios, a *p*-value below 0.05 indicated the existence of heterogeneity or horizontal pleiotropy. Moreover, we performed a leave-one-out analysis to evaluate the specific contribution of each SNP to the overall results of our study (20).

Our analysis involved a meta-synthesis aimed at assessing the overall causal link between traits associated with sarcopenia and a set of ten gastrointestinal diseases, combining outcomes from both the exploratory and confirmatory phases of MR investigations. From the analysis, we omitted results that indicated pleiotropy. The selection of the effect model was guided by the degree of heterogeneity present in the data; a fixed-effects model was applied when heterogeneity was negligible (I2 ≤ 50%), and a random-effects model was used in the presence of significant heterogeneity (I2 > 50%). The findings from the meta-synthesis were taken as the conclusive causal determinations. Nonetheless, in scenarios where there was a single MR result, that particular result was decisive for the final causal interpretation. Statistical significance was established at a *p*-value below 0.0004 (0.05/114, adhering to stringent Bonferroni correction), while *p*-values ranging from 0.0004 to 0.05 were viewed as suggestive of a potential causal correlation.

Previous studies have shown that muscle mass loss is closely related to metabolic syndrome (21), in order to evaluate the potential role of metabolic disorders as mediators in the causal pathway between ALM and diseases like GERD or NAFLD, we conducted a two-stage MR analysis. The first stage was dedicated to determining the causal influence of ALM on metabolic disorders through Univariate MR (UVMR), quantified by the coefficient β1. The second stage utilized both UVMR and Multivariate MR (MVMR) to gauge the causal effect of metabolic disorders on GERD and NAFLD, taking into account the influence of ALM, with the MVMR coefficients represented as β2. The mediation effect of metabolic disorders was ascertained by the ratio of β1 to β2, and the 95% confidence interval for this ratio was established using the Delta method (22, 23). All analyses were performed with R packages (Two Sample MR、MVMR and MR-PRESSO) in R 4.3.1.

## 3 Results

### 3.1 Genetic correlation

**Figure 2:**
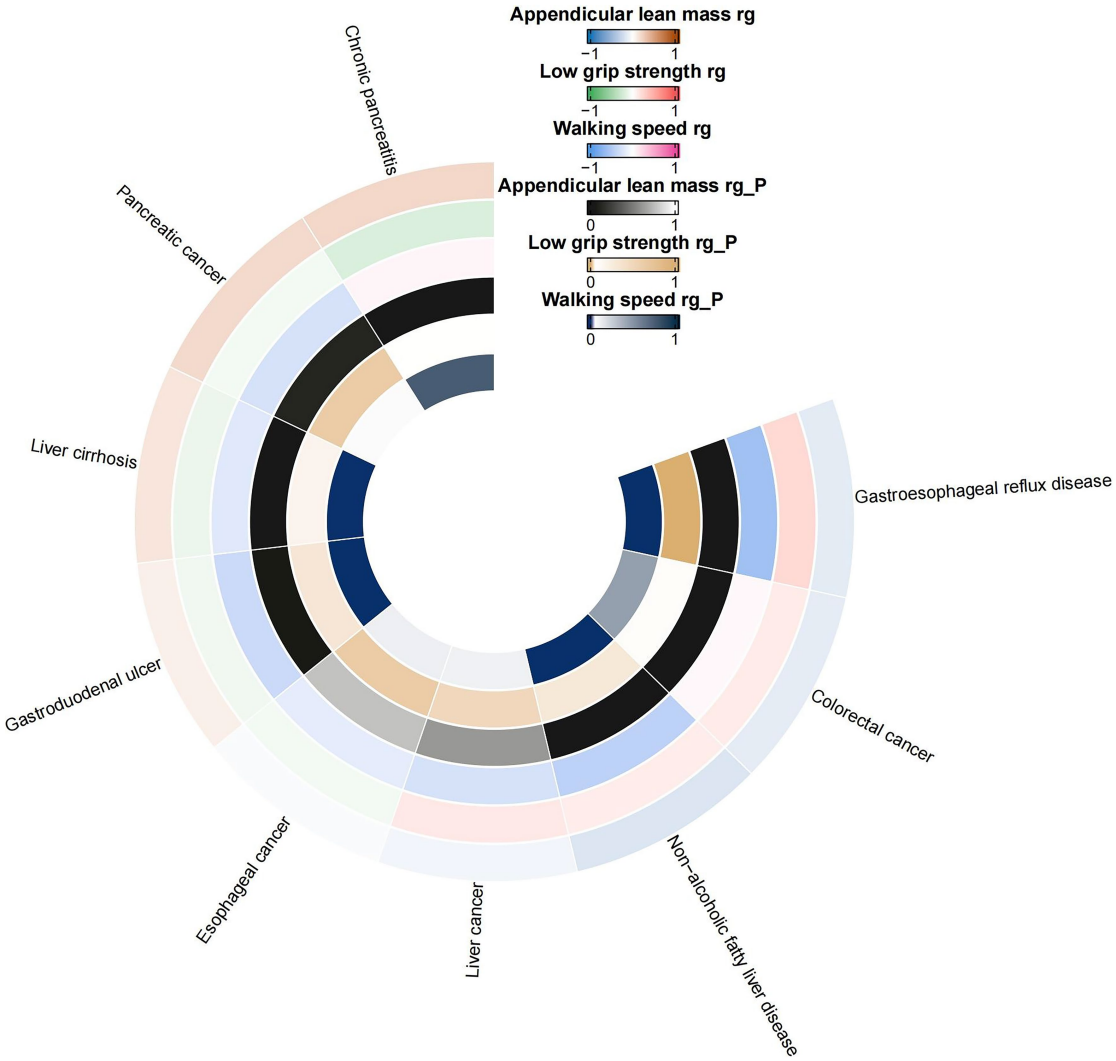
Circular heat map of suggestive genetic correlation between sarcopenia-related traits and 10 digestive system disorders. rg, genetic correlation.

Employing the LDSC regression technique, we assessed the genetic links between sarcopenia-associated characteristics and a variety of gastrointestinal conditions. As illustrated in Figure 2, the LDSC regression disclosed a noteworthy genetic association between ALM and GERD with a correlation coefficient (rg) of-0.1388 and a highly significant *p*-value of less than 0.0001. Similarly, ALM showed a notable genetic relationship with chronic pancreatitis, with an rg of 0.2122 and a p-value of 0.0006. The analysis also hinted at a potential genetic link between ALM and colorectal cancer (rg = −0.1260, *p* = 0.0028), NAFLD (rg = −0.1747, *p* = 0.0062), and liver cirrhosis (rg = 0.1364, *p* = 0.0043). In the case of reduced grip strength, a substantial genetic correlation with GERD was observed (rg = 0.2389, *p* < 0.0001). Moreover, a significant genetic correlation was detected between walking speed and several conditions: GERD (rg = −0.5952, *p* < 0.0001), NAFLD (rg = −0.4302, *p* < 0.0001), gastroduodenal ulcer (rg = −0.3490, *p* < 0.0001), and liver cirrhosis (rg = −0.2116, *p* = 0.0002). For comprehensive details on all genetic correlation results, please refer to Supplementary Table S2.

### 3.2 MR analysis

#### 3.2.1 Discovery results

Utilizing a discovery cohort, we delved into the causal interactions between characteristics associated with sarcopenia and a set of ten gastrointestinal diseases (refer to Supplementary Tables S3–S5 for specifics). The analysis uncovered a causal link where an increment in ALM per unit of log odds ratio (logOR) was correlated with four gastrointestinal conditions: GERD with an OR of 0.8598 (95% CI: 0.8220 to 0.8992; *p* < 0.0001), gastric cancer (OR: 1.1300; 95% CI: 1.0344 to 1.2343; *p* = 0.0067), colorectal cancer (OR: 0.7805; 95% CI: 0.6096 to 0.9992; *p* = 0.0493), and NAFLD (OR: 0.8124; 95% CI: 0.7342 to 0.8990; *p* < 0.0001). In addition, we detected a causal effect of diminished grip strength on GERD (OR: 1.2720; 95% CI: 1.0684 to 1.5145; *p* = 0.0069) and pancreatic cancer (OR: 1.7395; 95% CI: 1.1336 to 2.6693; *p* = 0.0113). Furthermore, a relationship was pinpointed between walking speed and several disorders: GERD (OR: 0.1184; 95% CI: 0.0819 to 0.1711; *p* < 0.0001), colorectal cancer (OR: 2.8346; 95% CI: 1.0351 to 7.7626; *p* = 0.0426), NAFLD (OR: 0.3961; 95% CI: 0.2111 to 0.7435; *p* = 0.0039), and gastroduodenal ulcer (OR: 0.9901; 95% CI: 0.9824 to 0.9979; *p* = 0.0127). The leave-one-out analysis revealed results that were consistent with the initial findings (Supplementary Figure S1). Sensitivity analyses indicated that certain findings displayed heterogeneity, yet no signs of pleiotropy were observed.

In the reverse MR analysis, we identified causal relationships between two types of digestive disorders and ALM, specifically gastroesophageal reflux disease (β: -0.0812; 95% CI: −0.1224 to-0.0400; *p* = 0.0001) and liver cancer (β: 9.7125; 95% CI: 1.500 to 17.9247; *p* = 0.0204). Additionally, we found a causal association between two types of digestive disorders and low grip strength, including gastroesophageal reflux disease (β: 0.1968; 95% CI: 0.1430 to 0.2506; *p* < 0.0001) and esophageal cancer (β: 0.0386; 95% CI: 0.0157 to 0.0616; *p* = 0.0009). Furthermore, a causal relationship was observed between two types of digestive disorders and walking pace, such as gastroesophageal reflux disease (β: -0.1329; 95% CI: −0.1456 to-0.1201; *p* < 0.0001) and liver cancer (β: -6.3579; 95% CI: −11.6900 to −1.0259; *p* = 0.0194). Analysis conducted through leave-one-out validation demonstrated outcomes that were analogous (Supplementary Figure S1). Sensitivity analysis indicated that some results displayed heterogeneity, with significant pleiotropy (*p* = 0.0110) detected only in the causal relationship between liver cancer and ALM. No pleiotropy was observed in the other causal relationships.

#### 3.2.2 Replication results

In our replication cohort, we identified a causal relationship between ALM and two digestive disorders: GERD (OR: 0.8615; 95% CI: 0.8255 to 0.8991; *p* < 0.0001) and NAFLD (OR: 0.7350; 95% CI: 0.5911 to 0.9141; *p* = 0.0057). Furthermore, we observed associations between low grip strength and GERD (OR: 1.1363; 95% CI: 1.0119 to 1.2759; *p* = 0.0307), as well as between walking pace and GERD (OR: 0.3196; 95% CI: 0.2222 to 0.4595; *p* < 0.0001), and gastroduodenal ulcer (OR: 0.4050; 95% CI: 0.1646 to 0.9963; *p* = 0.0491). Sensitivity analysis indicated some heterogeneity in the findings, but no evidence of pleiotropy was observed. Contrary to our expectations, the reverse MR analysis failed to identify any causal relationships between the ten examined digestive diseases and traits related to sarcopenia. For comprehensive details on all MR analysis and sensitivity analyses results, please refer to Supplementary Tables S5, S6.

#### 3.2.3 Combined results from the meta-analysis

**Figure 3:**
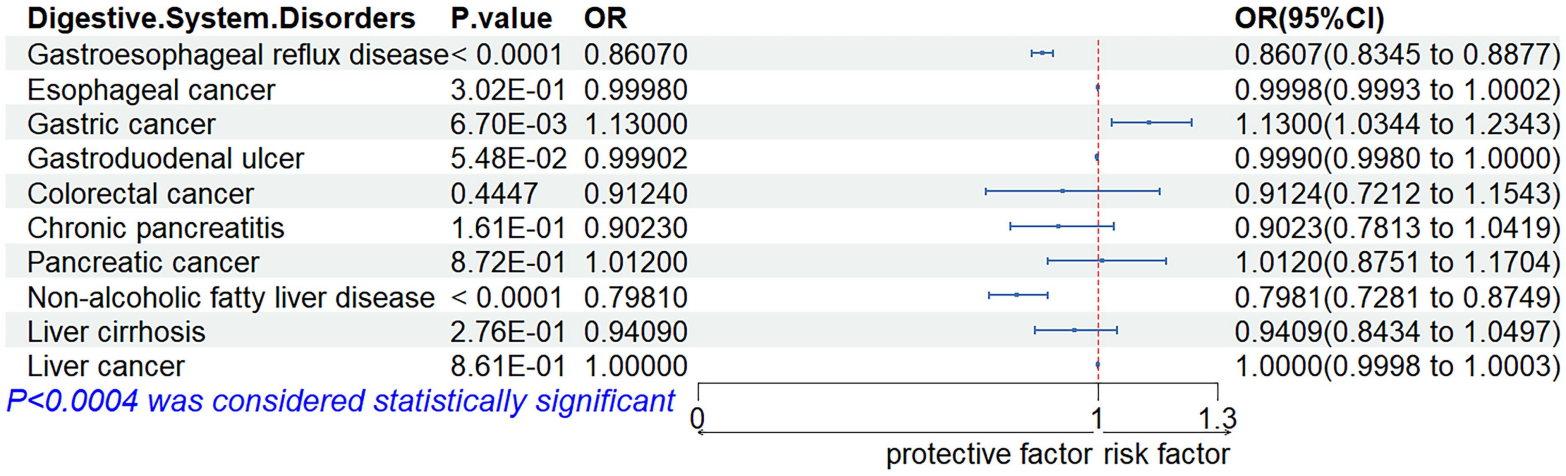
Associations of genetic liability to appendicular lean mass with digestive system disorders.

**Figure 4:**
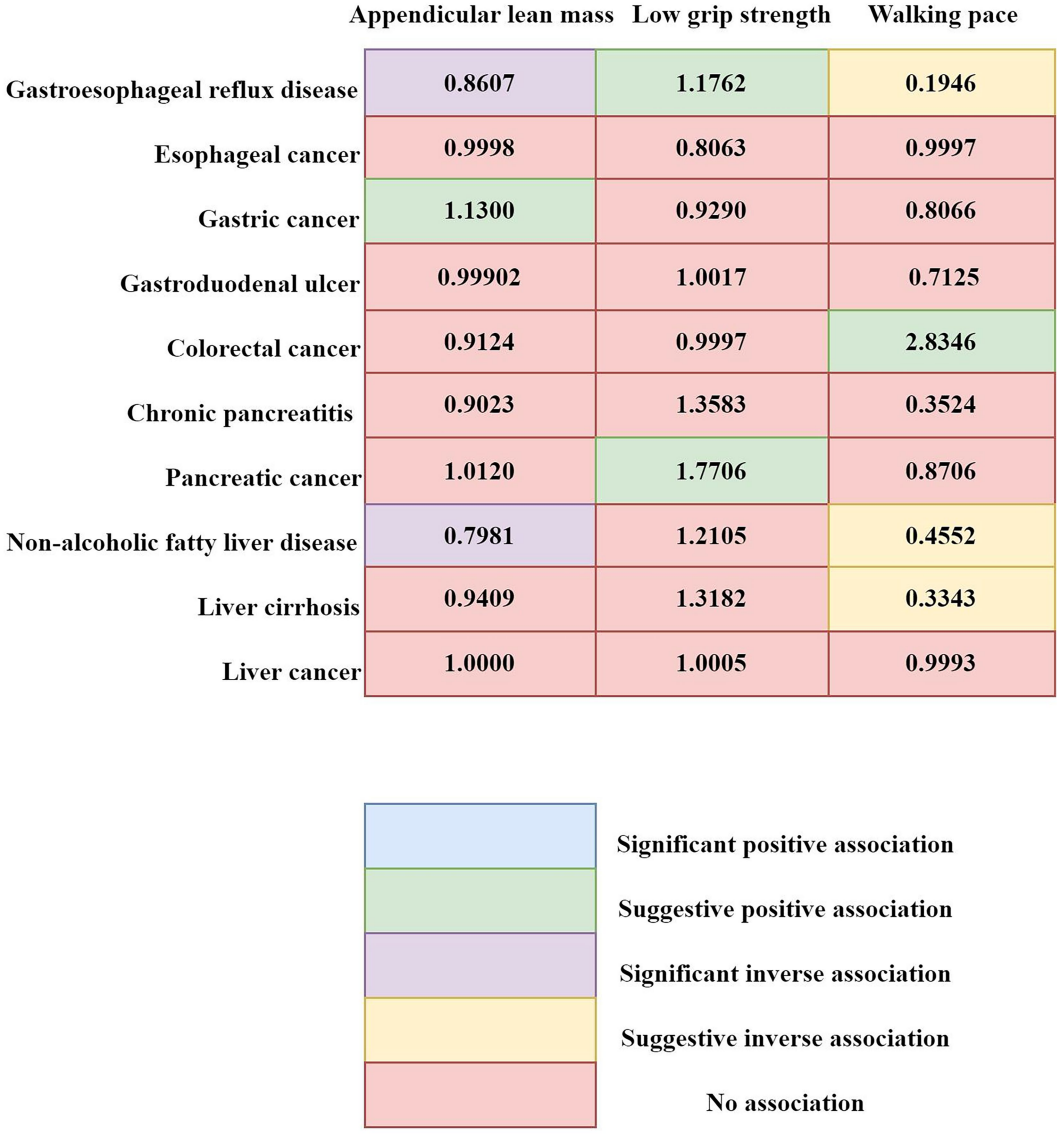
Summary of associations of genetically predicted sarcopenia-related traits and 10 digestive system disorders. Numbers in the boxes are ORs for associations of exposure and each gastrointestinal outcome. Statistical significance was established at a *p*-value <0.0004, while *p*-values ranging from 0.0004 to 0.05 were viewed as suggestive of a potential causal correlation.

The outcomes of our meta-analysis are detailed in Figures 3, 4 and Supplementary Table S6. We identified a robust causal relationship between ALM and GERD (OR = 0.8607; 95% CI: 0.8345–0.8877; *p* < 0.001), as well as a significant association with NAFLD (OR = 0.7981; 95% CI: 0.7281–0.8749; *p* = 0.0001). Additionally, the meta-analysis yielded suggestive evidence for a potential causal effect of ALM on gastric cancer (OR = 1.1300; 95% CI: 1.0344 to 1.2343; *p* = 0.0067). Evidence was also found suggesting that low grip strength may have a causal effect on GERD (OR = 1.1762; 95% CI: 1.0680 to 1.2955; *p* = 0.0010) and pancreatic cancer (OR = 1.7706; 95% CI: 1.2193 to 2.5710; *p* = 0.0027). Moreover, our meta-analysis provided suggestive evidence for potential causal effects of walking speed on GERD (OR = 0.1946; 95% CI: 0.0736 to 0.5149; *p* = 0.0010) and colorectal cancer (OR = 0.4552; 95% CI: 0.2502 to 0.8282; *p* = 0.0100). In the reverse MR analysis, we observed suggestive evidence of potential causal relationships between chronic pancreatitis and walking speed (β = −0.0031; 95% CI: −0.0059 to −0.0004; *p* = 0.0259), as well as between esophageal cancer and low grip strength (β = 0.0386; 95% CI: 0.0157–0.0616; p = 0.0010).

#### 3.2.4 Mediation analysis

In our analysis, we assessed the mediating impact of metabolic disorders on the relationship between ALM and gastroesophageal reflux disease as well as non-alcoholic fatty liver. The mediating effect in the pathway to gastroesophageal reflux disease is 0.0087 (95% CI, 8e-04, 0.0183), representing 5.9398% (95% CI, 0.5462, 12.4940%) of the total effect. For the pathway to non-alcoholic fatty liver, the mediating effect is 0.0150 (95% CI, 0.0050, 0.0270), contributing to 19.7808% (95% CI, 6.5936, 35.6055%) of the total effect (refer to Supplementary Table S7 for specifics).

## 4 Discussion

This study, to our knowledge, is the first to delve into the genetic connections and causal relationships between traits associated with sarcopenia and a spectrum of ten digestive system disorders, using GWAS summary statistics. The research reveals the genetic ties between ALM and various conditions including GERD, chronic pancreatitis, colorectal cancer, NAFLD, and cirrhosis. It also brings to light the association between reduced hand grip strength and GERD, and points to a possible relationship between walking pace and a range of disorders such as GERD, NAFLD, gastroduodenal ulcers, and cirrhosis. The MR analysis provided strong causal evidence, suggesting that an increased ALM is significantly associated with a lower risk of GERD and NAFLD. Additionally, suggestive evidence proposes a causal link between sarcopenia-related traits and gastric cancer, pancreatic cancer, colorectal cancer, and cirrhosis. In the reverse MR analysis, we detected suggestive evidence that may link chronic pancreatitis to walking speed and esophageal cancer to reduced grip strength.

In a cohort study based on health check-ups, sarcopenia emerged as a distinct predictive element for the development of GERD (4). A separate five-year longitudinal cohort investigation highlighted that diminished back muscle strength and the presence of sarcopenia are key contributors to the onset of GERD (24). Our research corroborates these findings. The genetic connection between traits associated with sarcopenia and GERD was also a significant discovery in this study. Further MR analyses have yielded robust evidence that genetically predicted elevated levels of ALM are inversely associated with a lower likelihood of GERD. Additionally, there is suggestive evidence that an individual’s walking pace may serve as a protective factor against GERD, while a weak grip could potentially raise the risk. This MR study expands upon previous research on sarcopenia and gastroesophageal reflux disease by including not only the loss of muscle mass, which has traditionally been the focus, but also by considering gait speed and grip strength in its definition of sarcopenia. This approach addresses a notable gap in the literature. The findings indicate that the risk of gastroesophageal reflux is primarily influenced by muscle mass, rather than aspects of strength or functional performance. This novel insight suggests that interventions aimed at increasing muscle mass could be particularly effective in reducing the risk of GERD, offering a new perspective on the relationship between sarcopenia and gastroesophageal reflux.

Evidence from prior research has indicated a link between sarcopenia and GERD, potentially connected to metabolic syndrome (4). It has been documented that sarcopenia may lead to insulin intolerance, a hallmark of metabolic syndrome, establishing a reciprocal and detrimental relationship (25). Furthermore, the loss of muscle mass due to sarcopenia can result in reduced physical activity, which in turn can cause an escalation of visceral adiposity (26). Since skeletal muscles play a pivotal role in insulin sensitivity, sarcopenic patients are prone to the progression of metabolic syndrome, which may impair the functionality of the esophagus and stomach as mechanical barriers, thus precipitating GERD (4). Our advanced mediation analysis substantiates this link, highlighting the role of metabolic disorders in this context, especially the 5.9398% influence of ALM on GERD. Conversely, there is no supporting evidence that reduced grip strength or walking velocity have a mediating impact on GERD via metabolic pathways. These insights contribute to a clearer understanding of the mechanisms underlying the relationship between sarcopenia and GERD.

Previous inquiries, including both observational and MR studies, have sought to understand the connection between sarcopenia and NAFLD, yielding incongruent results. A cross-sectional analysis demonstrated a significant and solitary link between reduced muscle mass and strength with the prevalence of NAFLD (5). In parallel, an MR investigation established a causal effect of genetically driven muscle wasting on the likelihood of NAFLD (27). A recent synthesis of 19 observational studies, along with data from the National Health and Nutrition Examination Survey (NHANES), has corroborated these findings (28, 29). On the other hand, an MR study did not establish a causal link between sarcopenia and NAFLD (30). Our research has uncovered a genetic link between ALM, walking velocity, and the risk of NAFLD. MR studies have provided robust evidence that a higher genetically predicted ALM is associated with a reduced risk of alcoholic fatty liver. There is also suggestive evidence that faster walking speeds may lower the risk of NAFLD. Conversely, no genetic or causal ties have been identified between diminished grip strength and NAFLD. These findings suggest that the risk of NAFLD may be primarily influenced by muscle mass rather than strength or functional capacity, indicating that interventions aimed at increasing muscle mass could be most effective in the prevention of NAFLD.

Research has established that skeletal muscle, being the principal tissue for insulin-mediated glucose metabolism, is pivotal in the process of insulin signaling (31). A reduction in skeletal muscle mass is associated with the development of insulin resistance and dysregulation of blood glucose levels, which can ultimately result in NAFLD (32). Our subsequent mediation analysis supports this relationship, indicating that it is through the exacerbation of metabolic disorders that skeletal muscle mass influences NAFLD, with a notable 18% effect of ALM. The analysis also revealed that neither low grip strength nor walking speed have a significant mediating role in the development of NAFLD through metabolic pathways.

Indeed, the precise pathways through which sarcopenia contributes to the risk of NAFLD remain to be fully understood. Current hypotheses suggest a connection with inflammatory processes and oxidative stress (33–35). There is evidence to suggest that a deficiency in Vitamin D could be a common factor in the development of both sarcopenia and NAFLD, potentially influencing this relationship (36–38). Moreover, skeletal muscle, recognized as an endocrine tissue, releases myokines—peptides like interleukin-6 (IL-6) and irisin (33). The diminished production of these advantageous myokines due to muscle loss could be a contributing factor in the pathogenesis of NAFLD.

In a propensity score-matched Asian cohort investigation, sarcopenia was identified as a potential significant contributor to the risk of developing colorectal, pancreatic, gastric, esophageal, and hepatocellular carcinomas (6). Nevertheless, our research uncovered a mere hint of a genetic link between ALM and colorectal cancer, without establishing any genetic connection to other gastrointestinal cancers related to sarcopenia. Simultaneously, the MR study offered only preliminary insights, suggesting a possible connection between a rise in ALM and an elevated likelihood for gastric cancer. It also indicated that a weakened grip strength might be associated with a greater risk of pancreatic cancer, while an increased walking pace could potentially lower the chances of developing colorectal cancer. However, the study did not establish a direct causal relationship between genetically determined sarcopenia and the development of esophageal or hepatocellular carcinomas. Our findings do not align with those from prior Asian cohort research, which might be due to population-specific causal interactions. Given that the cohort research comprised solely of individuals from the Asian population, these results may not be directly relevant to Europeans. Currently, there is a dearth of observational studies on the interplay between sarcopenia and gastrointestinal cancers in the European demographic. Future research should prioritize the initiation of high-caliber cohort studies to confirm and expand upon our results.

Our analysis has yielded robust findings, indicating a genetic link between ALM and chronic pancreatitis, as well as between walking velocity and the occurrence of gastroduodenal ulcers. There is also suggestive data pointing to a genetic association with ALM, brisk walking, and the likelihood of liver cirrhosis. Concurrently, MR studies have provided suggestive evidence that an increased walking pace may lower the risk of cirrhosis. However, no direct causal link was observed between sarcopenia, determined by genetics, and either chronic pancreatitis or gastroduodenal ulcers. In the inverse MR studies, we identified only suggestive evidence for a potential causal effect of genetic predisposition to chronic pancreatitis on walking speed, and a genetic predisposition to esophageal cancer associated with reduced grip strength. For the remaining eight gastrointestinal conditions, no causal connections to sarcopenia were discerned. Observational research in the past has indicated that sarcopenia is associated with a notable rise in the occurrence of cirrhosis and gastroduodenal ulcers (39, 40). Additionally, gastrointestinal malignancies and chronic pancreatitis have been shown to markedly elevate the risk of developing sarcopenia (41, 42). Despite these findings, the observational nature of most prior studies leaves the causality of these associations in question. In contrast, MR analysis offers a more robust approach by mitigating the influence of confounding elements and the issue of reverse causality, thus providing more dependable evidence regarding these relationships.

Our investigation offers a number of distinct benefits. Initially, this marks the inaugural use of MR to bidirectionally assess the causal links between sarcopenia and an array of gastrointestinal disorders. This MR approach, with its capacity to pinpoint causality, stands out over traditional observational studies by circumventing confounding influences and the potential for reverse causality. Secondly, the inclusion of a wide range of gastrointestinal conditions in our study allows for the most comprehensive evaluation yet of the association between sarcopenia and these diseases. Lastly, we have performed both discovery and replication analyses on gastrointestinal disease GWAS data from two distinct sources. By excluding pleiotropy and synthesizing the findings, we have ensured the dependability of our results.

We must concede that there are certain limitations to this research. Initially, the constraints on the number of accessible GWAS studies or the occurrence of significant pleiotropy in some findings result in conclusions derived from a single data source, which may compromise their strength. Furthermore, since the study’s participants were primarily of European ancestry, the broader applicability of our findings to populations of different ethnicities is uncertain. It is imperative that these results be substantiated through further research involving a more heterogeneous group of individuals. In addition, although our study, through GWAS data aggregation, suggests the potential utility of augmenting muscle mass in preventing gastroesophageal reflux disease and non-alcoholic fatty liver disease, further comprehensive investigations are imperative to corroborate our findings and elucidate the underlying mechanisms.

## 5 Conclusion

To encapsulate the findings, this research discloses the causal relationships that sarcopenia has with a multitude of gastrointestinal conditions and the pivotal part that metabolic disorders play as mediators in these pathways. The study suggests that increasing muscle mass could be a constructive approach to preventing gastroesophageal reflux disease and non-alcoholic fatty liver, underscoring the significance of metabolic disorder management as a vital strategy in curbing the risks associated with these diseases that are linked to sarcopenia.

## Data availability statement

The datasets presented in this study can be found in online repositories. The names of the repository/repositories and accession number(s) can be found in the article/Supplementary material.

## Ethics statement

Written informed consent from the patients/participants or the patients’/participants’ legal guardian/next of kin was not required to participate in this study in accordance with the national legislation and the institutional requirements.

## Author contributions

TY: Conceptualization, Data curation, Formal analysis, Funding acquisition, Investigation, Methodology, Project administration, Resources, Software, Supervision, Validation, Visualization, Writing – original draft, Writing – review & editing. ZL: Investigation, Writing – original draft. MX: Data curation, Formal analysis, Writing – review & editing. XQ: Software, Supervision, Writing – review & editing. SL: Resources, Supervision, Writing – review & editing. WX: Resources, Supervision, Writing – original draft. ML: Resources, Supervision, Writing – review & editing.

## References

[1] Cruz-Jentoft AJ Sayer AA. Sarcopenia. Lancet. (2019) 393:2636–46. doi: 10.1016/s0140-6736(19)31138-9 31171417

[2] Yuan S Larsson SC. Epidemiology of sarcopenia: prevalence, risk factors, and consequences. Metab Clin Exp. (2023) 144:155533. doi: 10.1016/j.metabol.2023.155533 36907247

[3] Zhang FM Wu HF Shi HP Yu Z Zhuang CL. Sarcopenia and malignancies: epidemiology, clinical classification and implications. Ageing Res Rev. (2023) 91:102057. doi: 10.1016/j.arr.2023.102057 37666432

[4] Kim YM Kim JH Baik SJ Jung DH Park JJ Youn YH. Association between skeletal muscle attenuation and gastroesophageal reflux disease: a health check-up cohort study. Sci Rep. (2019) 9:20102. doi: 10.1038/s41598-019-56702-6 31882910 PMC6934459

[5] Gan D Wang L Jia M Ru Y Ma Y Zheng W. Low muscle mass and low muscle strength associate with nonalcoholic fatty liver disease. Clin Nutr. (2020) 39:1124–30. doi: 10.1016/j.clnu.2019.04.023 31053512

[6] Sun MY Chang CL Lu CY Wu SY Zhang JQ. Sarcopenia as an independent risk factor for specific cancers: a propensity score-matched Asian population-based cohort study. Nutrients. (2022) 14:1910. doi: 10.3390/nu14091910 35565877 PMC9105218

[7] Smith GD Ebrahim S. ‘Mendelian randomization’: can genetic epidemiology contribute to understanding environmental determinants of disease?. Int J Epidemiol. (2019) 32:1–22. doi: 10.1093/ije/dyg070 12689998

[8] Lee K Lim CY. Mendelian randomization analysis in observational epidemiology. J Lipid Atheroscler. (2019) 8:67–77. doi: 10.12997/jla.2019.8.2.67 32821701 PMC7379124

[9] Lawlor DA Harbord RM Sterne JA Timpson N Davey Smith G. Mendelian randomization: using genes as instruments for making causal inferences in epidemiology. Stat Med. (2008) 27:1133–63. doi: 10.1002/sim.3034 17886233

[10] Sakaue S Kanai M Tanigawa Y Karjalainen J Kurki M Koshiba S. A cross-population atlas of genetic associations for 220 human phenotypes. Nat Genet. (2021) 53:1415–24. doi: 10.1038/s41588-021-00931-xIF:30.8.Q1 34594039 PMC12208603

[11] Ghodsian N Abner E Emdin CA Gobeil É Taba Haas ME. Electronic health record-based genome-wide meta-analysis provides insights on the genetic architecture of non-alcoholic fatty liver disease. Cell Rep Med. (2021) 2:100437. doi: 10.1016/j.xcrm.2021.100437IF:14.3.Q1 34841290 PMC8606899

[12] Burgess S Thompson SG. Avoiding bias from weak instruments in Mendelian randomization studies. Int J Epidemiol. (2011) 40:755–64. doi: 10.1093/ije/dyr036 21414999

[13] Bulik-Sullivan BK Loh PR Finucane HK Ripke S Yang J Patterson N. LD score regression distinguishes confounding from polygenicity in genome-wide association studies. Nat Genet. (2015) 47:291–5. doi: 10.1038/ng.3211 25642630 PMC4495769

[14] ReproGen Consortium Psychiatric Genomics Consortium Genetic Consortium for Anorexia Nervosa of the Wellcome Trust Case Control Consortium 3 Bulik-Sullivan B Finucane HK Anttila V. An atlas of genetic correlations across human diseases and traits. Nat Genet. (2015) 47:1236–41. doi: 10.1038/ng.3406 26414676 PMC4797329

[15] Curtin F Schulz P. Multiple correlations and Bonferroni's correction. Biol Psychiatry. (1998) 44:775–7. doi: 10.1016/s0006-3223(98)00043-2 9798082

[16] Hemani G Zheng J Elsworth B Wade KH Haberland V Baird D. The MR-base platform supports systematic causal inference across the human phenome. eLife. (2018) 7:e34408. doi: 10.7554/eLife.34408 29846171 PMC5976434

[17] Burgess S Foley CN Allara E Staley JR Howson JMM. A robust and efficient method for Mendelian randomization with hundreds of genetic variants. Nat Commun. (2020) 11:376. doi: 10.1038/s41467-019-14156-4 31953392 PMC6969055

[18] Bowden J Davey Smith G Haycock PC Burgess S. Consistent estimation in Mendelian randomization with some invalid instruments using a weighted median estimator. Genet Epidemiol. (2016) 40:304–14. doi: 10.1002/gepi.21965 27061298 PMC4849733

[19] Verbanck M Chen CY Neale B Do R. Detection of widespread horizontal pleiotropy in causal relationships inferred from Mendelian randomization between complex traits and diseases. Nat Genet. (2018) 50:693–8. doi: 10.1038/s41588-018-0099-7 29686387 PMC6083837

[20] Burgess S Bowden J Fall T Ingelsson E Thompson SG. Sensitivity analyses for robust causal inference from Mendelian randomization analyses with multiple genetic variants. Epidemiology. (2017) 28:30–42. doi: 10.1097/ede.0000000000000559 27749700 PMC5133381

[21] Nishikawa H Asai A Fukunishi S Nishiguchi S Higuchi K. Metabolic syndrome and sarcopenia. Nutrients. (2021) 13:3519. doi: 10.3390/nu13103519 34684520 PMC8541622

[22] Carter AR Sanderson E Hammerton G Richmond RC Davey Smith G Heron J. Mendelian randomisation for mediation analysis: current methods and challenges for implementation. Eur J Epidemiol. (2021) 36:465–78. doi: 10.1007/s10654-021-00757-1 33961203 PMC8159796

[23] MacKinnon DP Lockwood CM Hoffman JM West SG Sheets V. A comparison of methods to test mediation and other intervening variable effects. Psychol Methods. (2002) 7:83–104. doi: 10.1037/1082-989x.7.1.83 11928892 PMC2819363

[24] Imagama S Ando K Kobayashi K Machino M Tanaka S Morozumi M. Increase in lumbar kyphosis and spinal inclination, declining back muscle strength, and sarcopenia are risk factors for onset of GERD: a 5-year prospective longitudinal cohort study. Eur Spine J. (2019) 28:2619–28. doi: 10.1007/s00586-019-06139-2 31506765

[25] Gaster M Vach W Beck-Nielsen H Schrøder HD. GLUT4 expression at the plasma membrane is related to fibre volume in human skeletal muscle fibres. APMIS. (2002) 110:611–9. doi: 10.1034/j.1600-0463.2002.1100903.x 12529013

[26] Choi KM. Sarcopenia and sarcopenic obesity. Endocrinol Metab. (2013) 28:86–9. doi: 10.3803/EnM.2013.28.2.86 PMC3811714 24396659

[27] Ye C Kong L Wang Y Zheng J Xu M Xu Y. Causal associations of sarcopenia-related traits with cardiometabolic disease and Alzheimer's disease and the mediating role of insulin resistance: a Mendelian randomization study. Aging Cell. (2023) 22:e13923. doi: 10.1111/acel.13923 37403750 PMC10497819

[28] Cai C Song X Chen Y Chen X Yu C. Relationship between relative skeletal muscle mass and nonalcoholic fatty liver disease: a systematic review and meta-analysis. Hepatol Int. (2020) 14:115–26. doi: 10.1007/s12072-019-09964-1 31290072 PMC6994447

[29] Zhao X Shi X Gu H Zhou W Zhang Q. Association between handgrip strength, nonalcoholic fatty liver disease, advanced hepatic fibrosis and its modifiers: evidence from the NHANES database of the USA. J Gastroenterol Hepatol. (2023) 38:1734–42. doi: 10.1111/jgh.16150 36805682

[30] Zhao ZH Zou J Huang X Fan YC Wang K. Assessing causal relationships between sarcopenia and nonalcoholic fatty liver disease: a bidirectional Mendelian randomization study. Front Nutr. (2022) 9:971913. doi: 10.3389/fnut.2022.971913 36438727 PMC9682105

[31] DeFronzo RA Jacot E Jequier E Maeder E Wahren J Felber JP. The effect of insulin on the disposal of intravenous glucose. Results from indirect calorimetry and hepatic and femoral venous catheterization. Diabetes. (1981) 30:1000–7. doi: 10.2337/diab.30.12.1000 7030826

[32] Bhanji RA Narayanan P Allen AM Malhi H Watt KD. Sarcopenia in hiding: the risk and consequence of underestimating muscle dysfunction in nonalcoholic steatohepatitis. Hepatology. (2017) 66:2055–65. doi: 10.1002/hep.29420 28777879

[33] Joo SK Kim W. Interaction between sarcopenia and nonalcoholic fatty liver disease. Clin Mol Hepatol. (2023) 29:S68–s78. doi: 10.3350/cmh.2022.0358 36472051 PMC10029947

[34] Beyer I Mets T Bautmans I. Chronic low-grade inflammation and age-related sarcopenia. Curr Opin Clin Nutr Metab Care. (2012) 15:12–22. doi: 10.1097/MCO.0b013e32834dd297 22108098

[35] Wree A Kahraman A Gerken G Canbay A. Obesity affects the liver - the link between adipocytes and hepatocytes. Digestion. (2011) 83:124–33. doi: 10.1159/000318741 21042023

[36] Eliades M Spyrou E Agrawal N Lazo M Brancati FL Potter JJ. Meta-analysis: vitamin D and non-alcoholic fatty liver disease. Aliment Pharmacol Ther. (2013) 38:246–54. doi: 10.1111/apt.12377 23786213

[37] Barchetta I Angelico F Ben MD Baroni MG Pozzilli P Morini S. Strong association between non alcoholic fatty liver disease (NAFLD) and low 25(OH) vitamin D levels in an adult population with normal serum liver enzymes. BMC Med. (2011) 9:85. doi: 10.1186/1741-7015-9-85 21749681 PMC3148980

[38] Visser M Deeg DJ Lips P. Low vitamin D and high parathyroid hormone levels as determinants of loss of muscle strength and muscle mass (sarcopenia): the longitudinal aging study Amsterdam. J Clin Endocrinol Metab. (2003) 88:5766–72. doi: 10.1210/jc.2003-030604 14671166

[39] Choi YI Chung JW Park DK Ko KP Kim KO Kwon KA. Sarcopenia is independently associated with an increased risk of peptic ulcer disease: a Nationwide population-based study. Medicina (Kaunas). (2020) 56:121. doi: 10.3390/medicina56030121 32168799 PMC7143528

[40] Cui Y Zhang M Guo J Jin J Wang H Wang X. Correlation between sarcopenia and cirrhosis: a meta-analysis. Front Nutr. (2023) 10:1342100. doi: 10.3389/fnut.2023.1342100 38268669 PMC10805929

[41] Surov A Wienke A. Prevalence of sarcopenia in patients with solid tumors: a meta-analysis based on 81,814 patients. JPEN J Parenter Enteral Nutr. (2022) 46:1761–8. doi: 10.1002/jpen.2415 35633306

[42] Bundred J Thakkar RG Pandanaboyana S. Systematic review of sarcopenia in chronic pancreatitis: prevalence, impact on surgical outcomes, and survival. Expert Rev Gastroenterol Hepatol. (2022) 16:665–72. doi: 10.1080/17474124.2022.2091544 35712996

