# Supplementary material for: Sarcopenia-related traits and 10 digestive system disorders: insight from genetic correlation and Mendelian randomization
Source: Front Public Health. 2024 Jul 10;12:1412842. doi: 10.3389/fpubh.2024.1412842 (PMC11267997; doi:10.3389/fpubh.2024.1412842)
Supplement: Supplementary file 1 [file Data_Sheet_1.PDF]

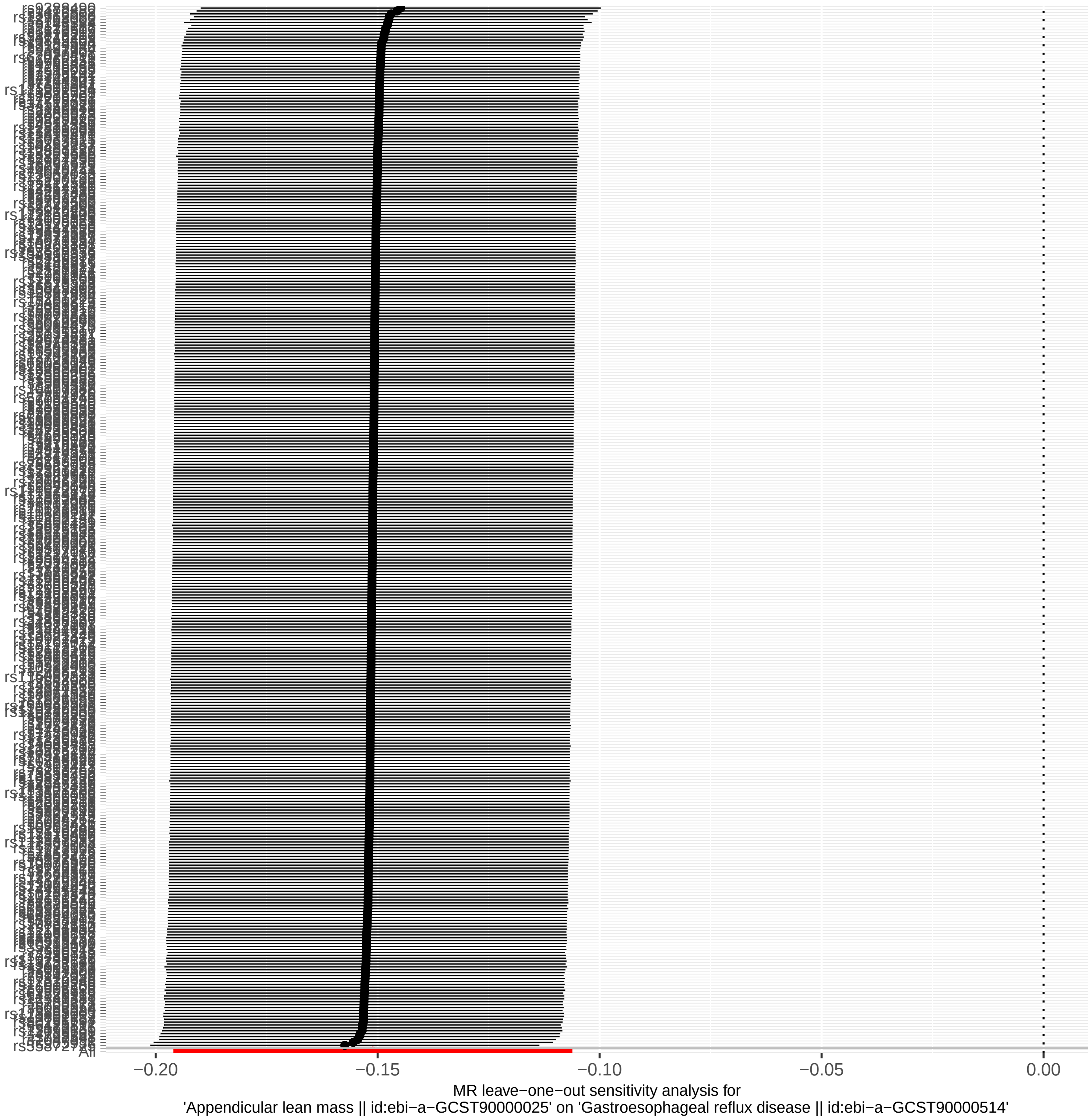
All
−0.20
−0.15
−0.10
−0.05
0.00
MR leave−one−out sensitivity analysis for
'Appendicular lean mass || id:ebi−a−GCST90000025' on 'Gastroesophageal reflux disease || id:ebi−a−GCST90000514'

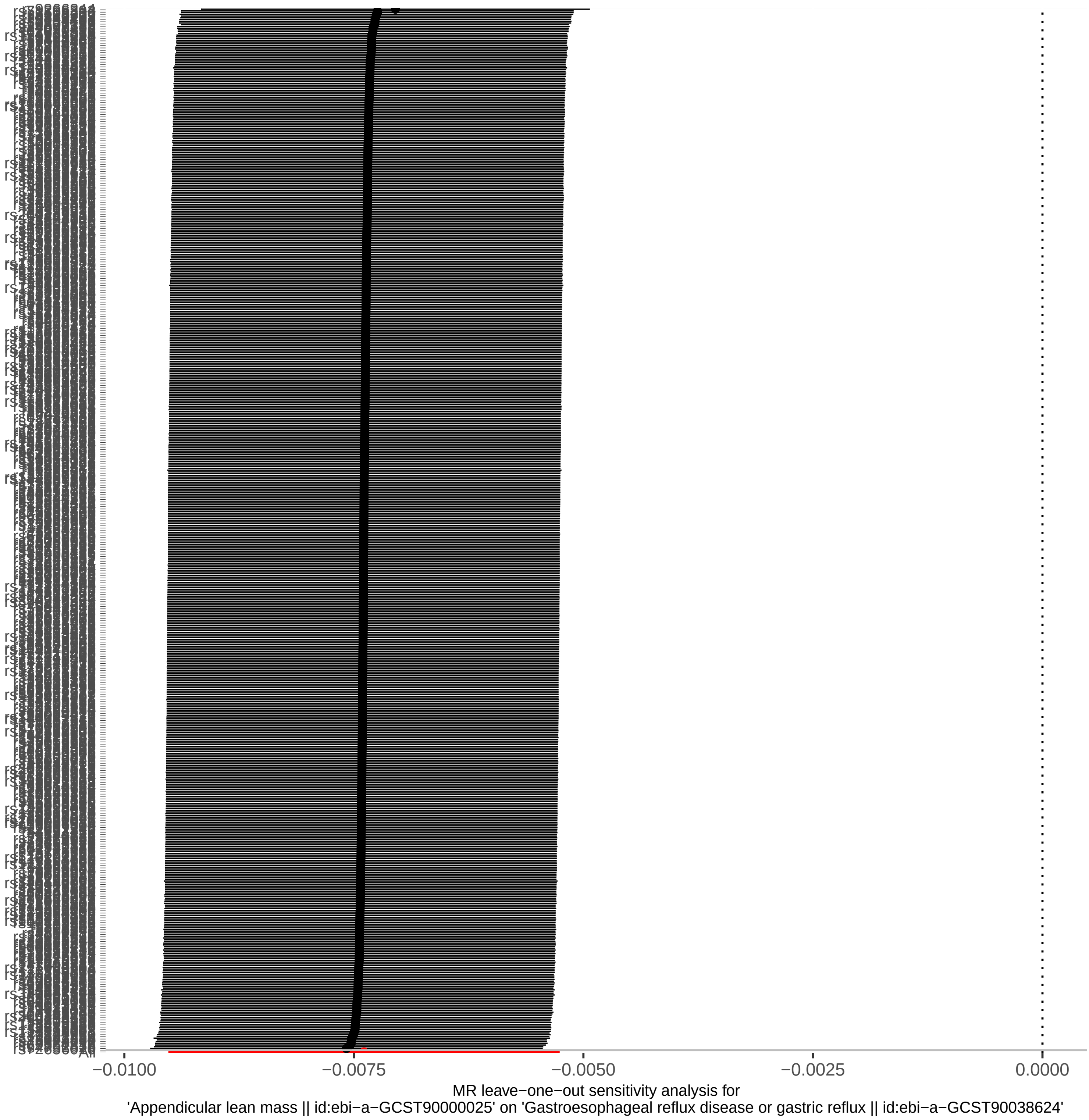

All
−0.0100
−0.0075
−0.0050
−0.0025
0.0000
MR leave−one−out sensitivity analysis for
'Appendicular lean mass || id:ebi−a−GCST90000025' on 'Gastroesophageal reflux disease or gastric reflux || id:ebi−a−GCST90038624'

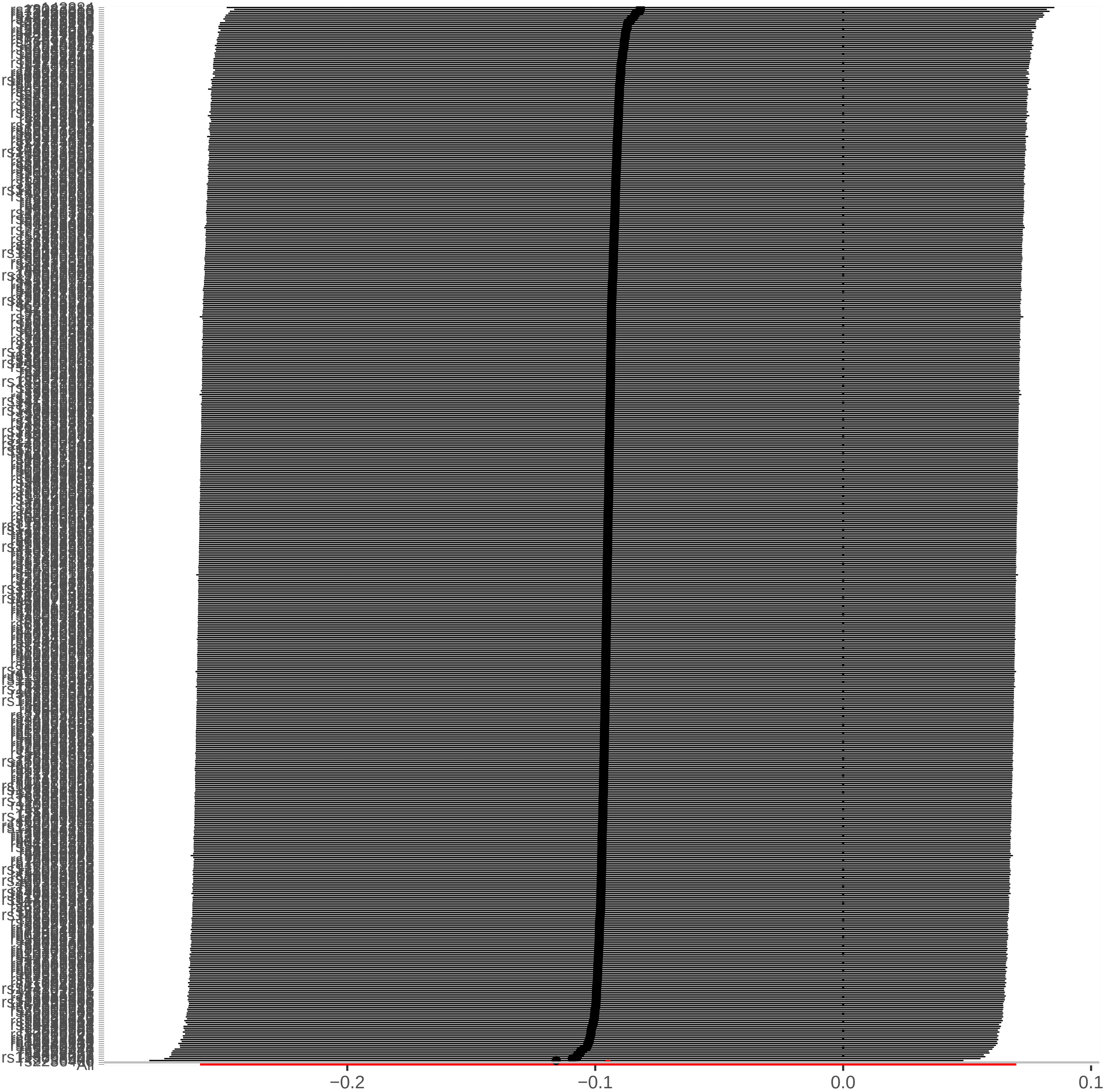

MR leave−one−out sensitivity analysis for
'Appendicular lean mass || id:ebi−a−GCST90000025' on 'Chronic pancreatitis || id:finn−b−K11_CHRONPANC'

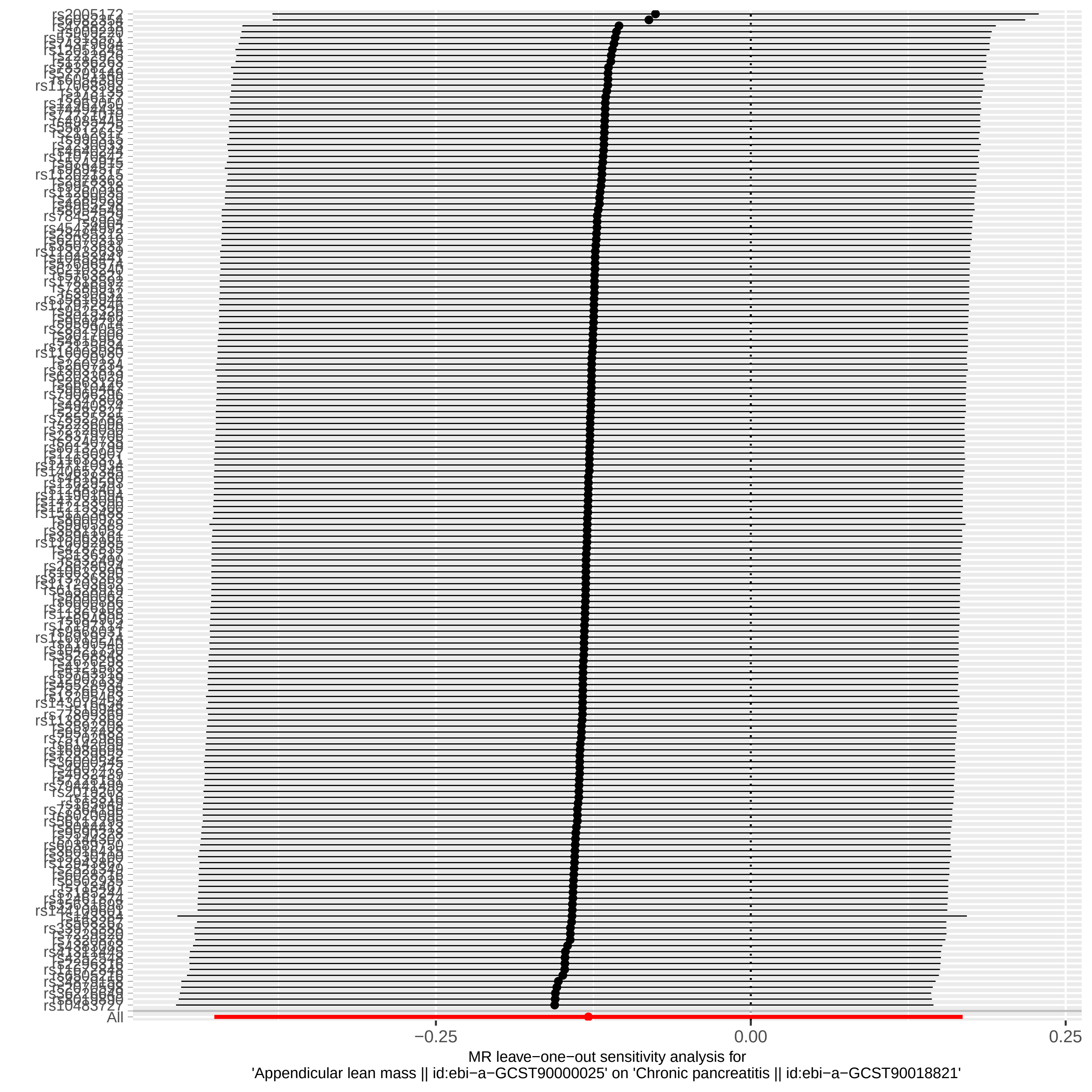
rs2005172
rs10483727
All
−0.25
0.00
0.25
MR leave−one−out sensitivity analysis for
'Appendicular lean mass || id:ebi−a−GCST90000025' on 'Chronic pancreatitis || id:ebi−a−GCST90018821'

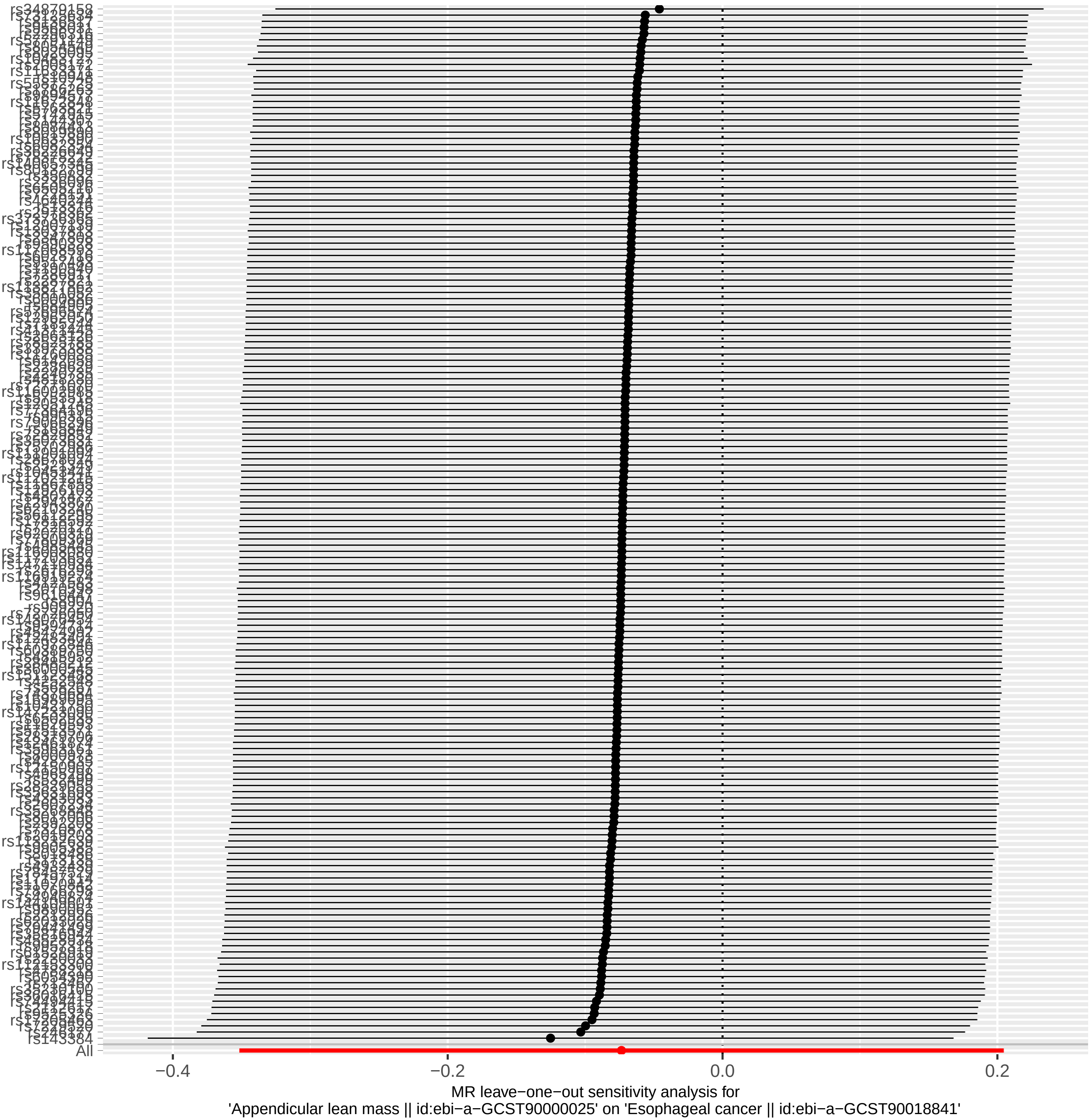
rs34879158
rs143384
All
−0.4
−0.2
0.0
0.2
MR leave−one−out sensitivity analysis for
'Appendicular lean mass || id:ebi−a−GCST90000025' on 'Esophageal cancer || id:ebi−a−GCST90018841'

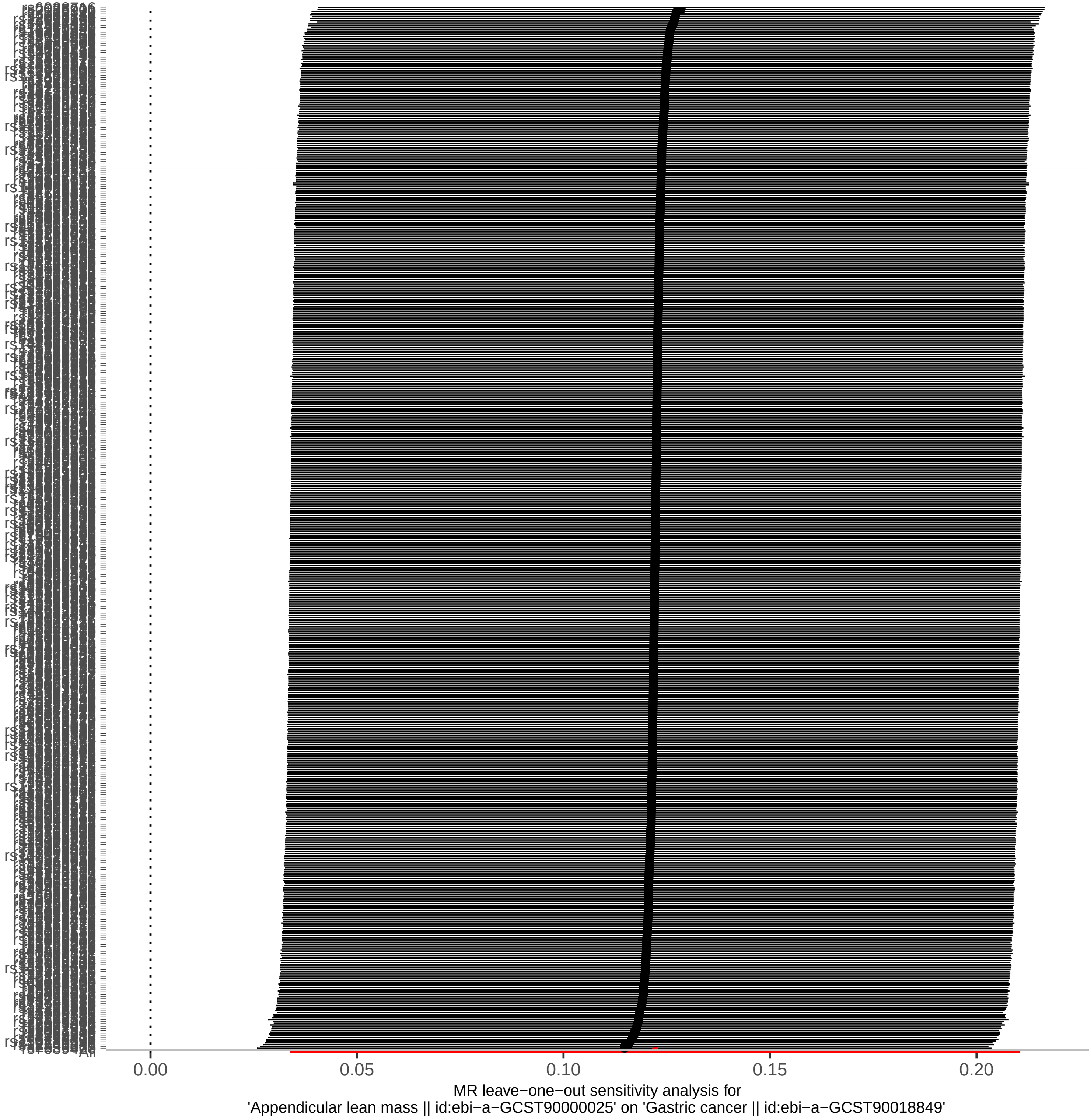

All
0.00
0.05
0.10
0.15
0.20
MR leave−one−out sensitivity analysis for
'Appendicular lean mass || id:ebi−a−GCST90000025' on 'Gastric cancer || id:ebi−a−GCST90018849'

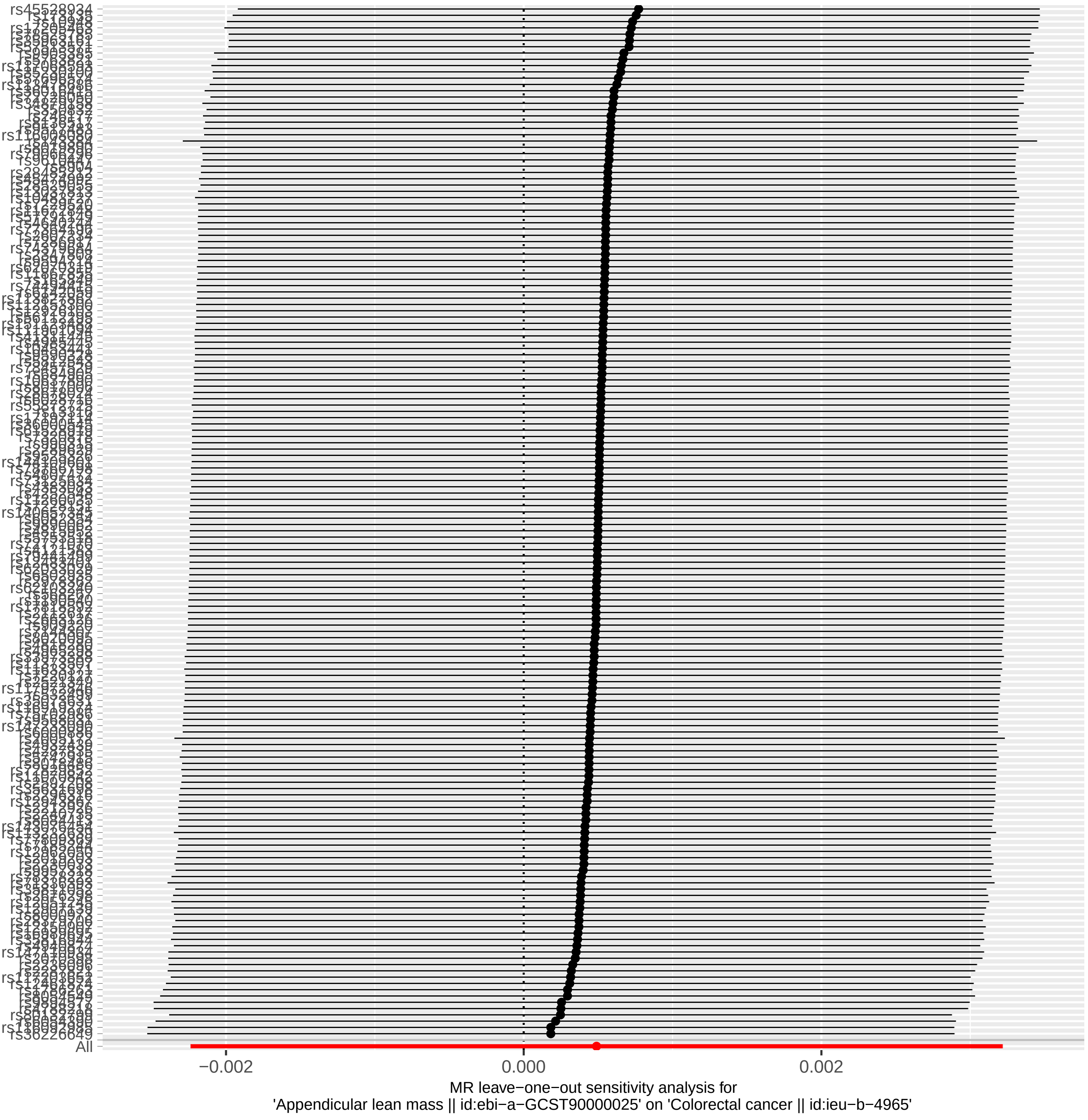
rs45528934
All
−0.002
0.000
0.002
MR leave−one−out sensitivity analysis for
'Appendicular lean mass || id:ebi−a−GCST90000025' on 'Colorectal cancer || id:ieu−b−4965'

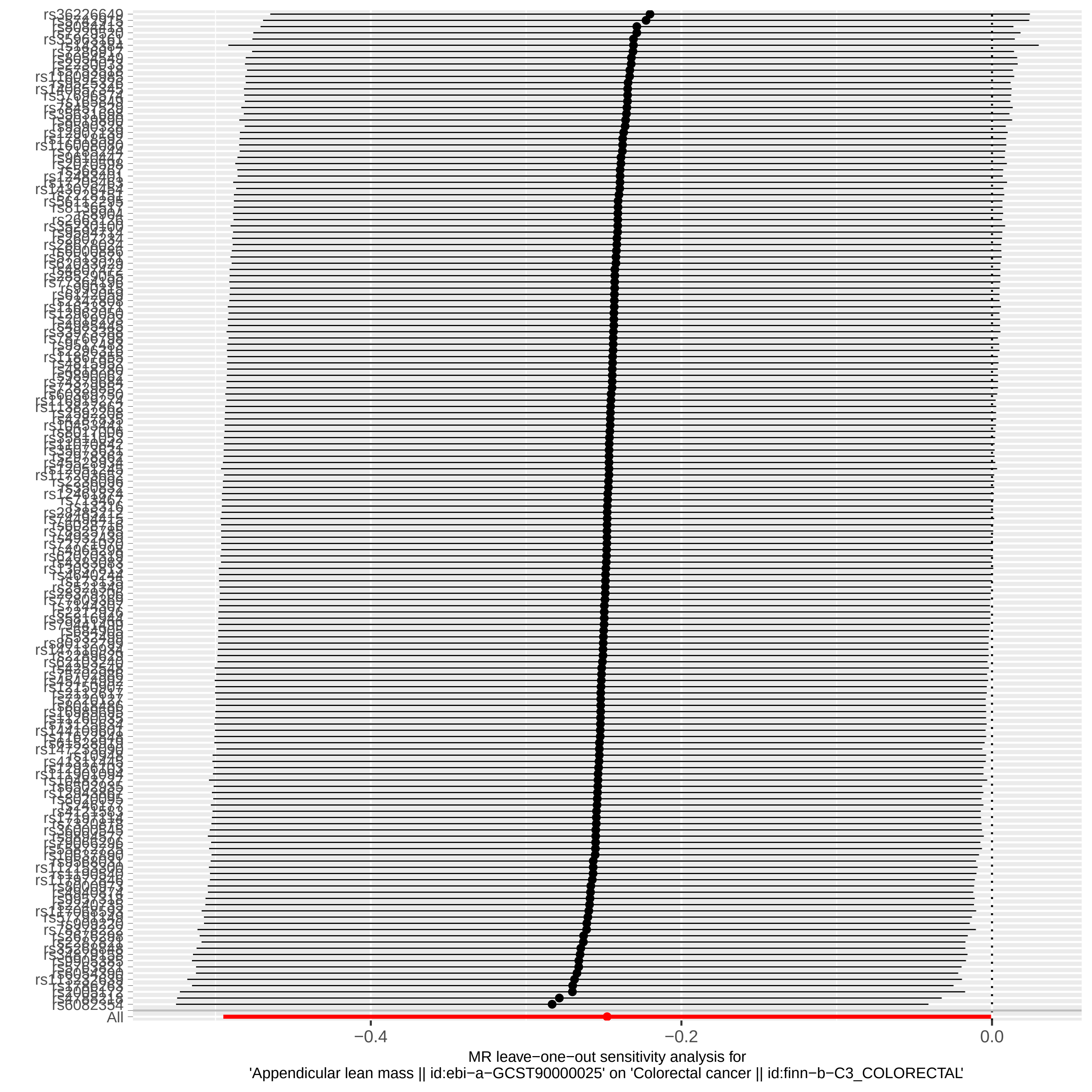
rs36226649
rs6082354
All
−0.4
−0.2
0.0
MR leave−one−out sensitivity analysis for
'Appendicular lean mass || id:ebi−a−GCST90000025' on 'Colorectal cancer || id:finn−b−C3_COLORECTAL'

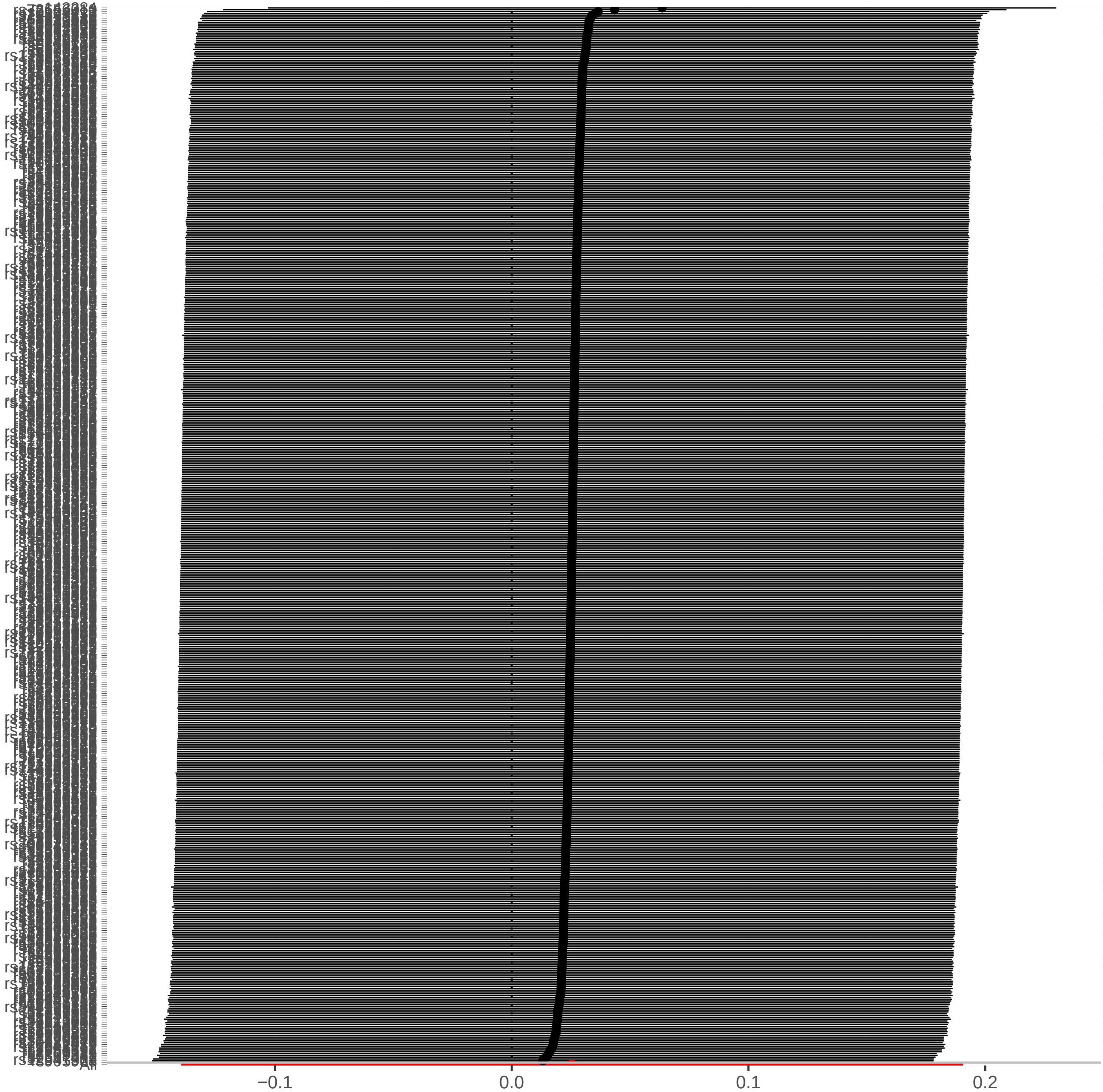

MR leave−one−out sensitivity analysis for
'Appendicular lean mass || id:ebi−a−GCST90000025' on 'Pancreatic cancer || id:ebi−a−GCST90018893'

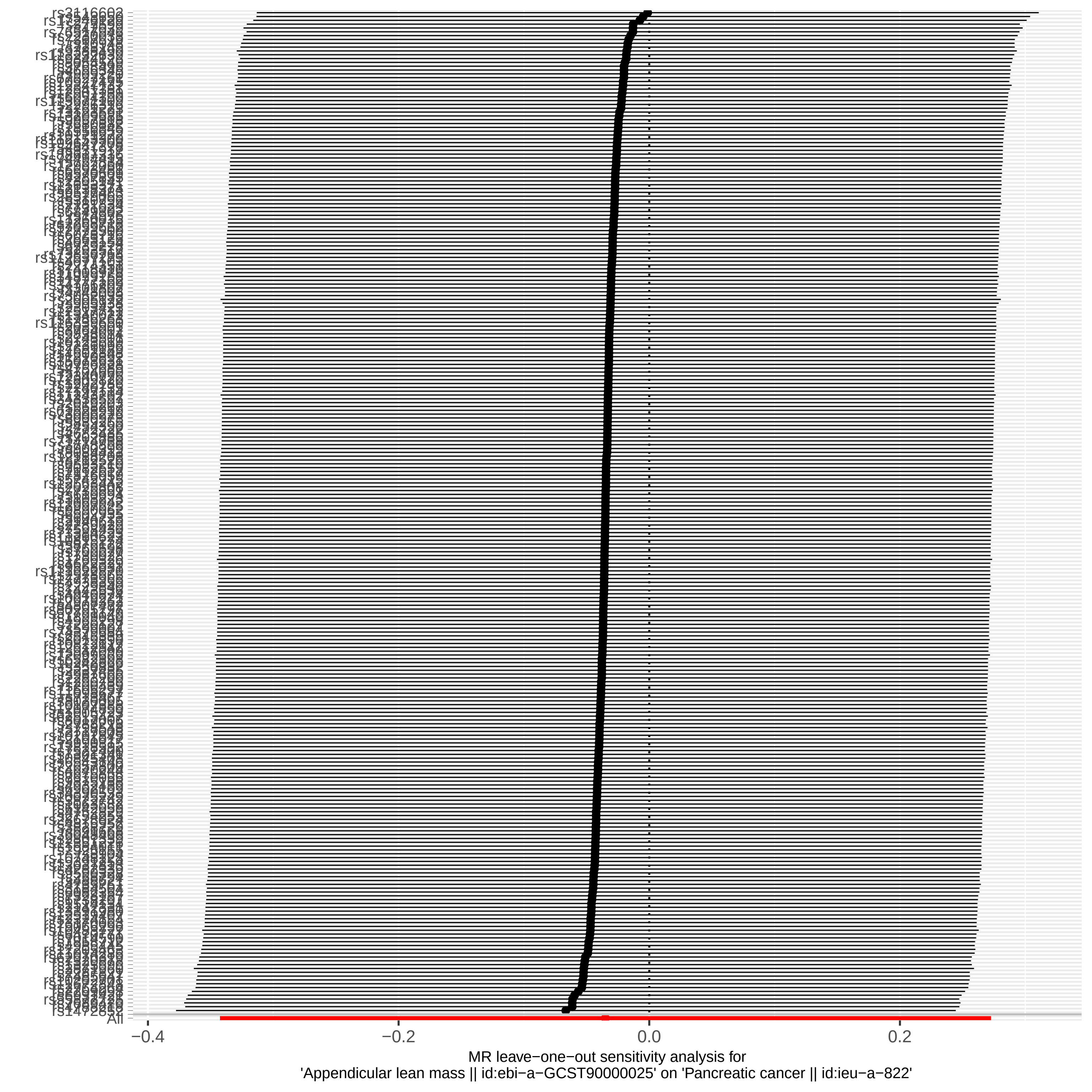
rs3116602
All
−0.4
−0.2
0.0
0.2
MR leave−one−out sensitivity analysis for
'Appendicular lean mass || id:ebi−a−GCST90000025' on 'Pancreatic cancer || id:ieu−a−822'

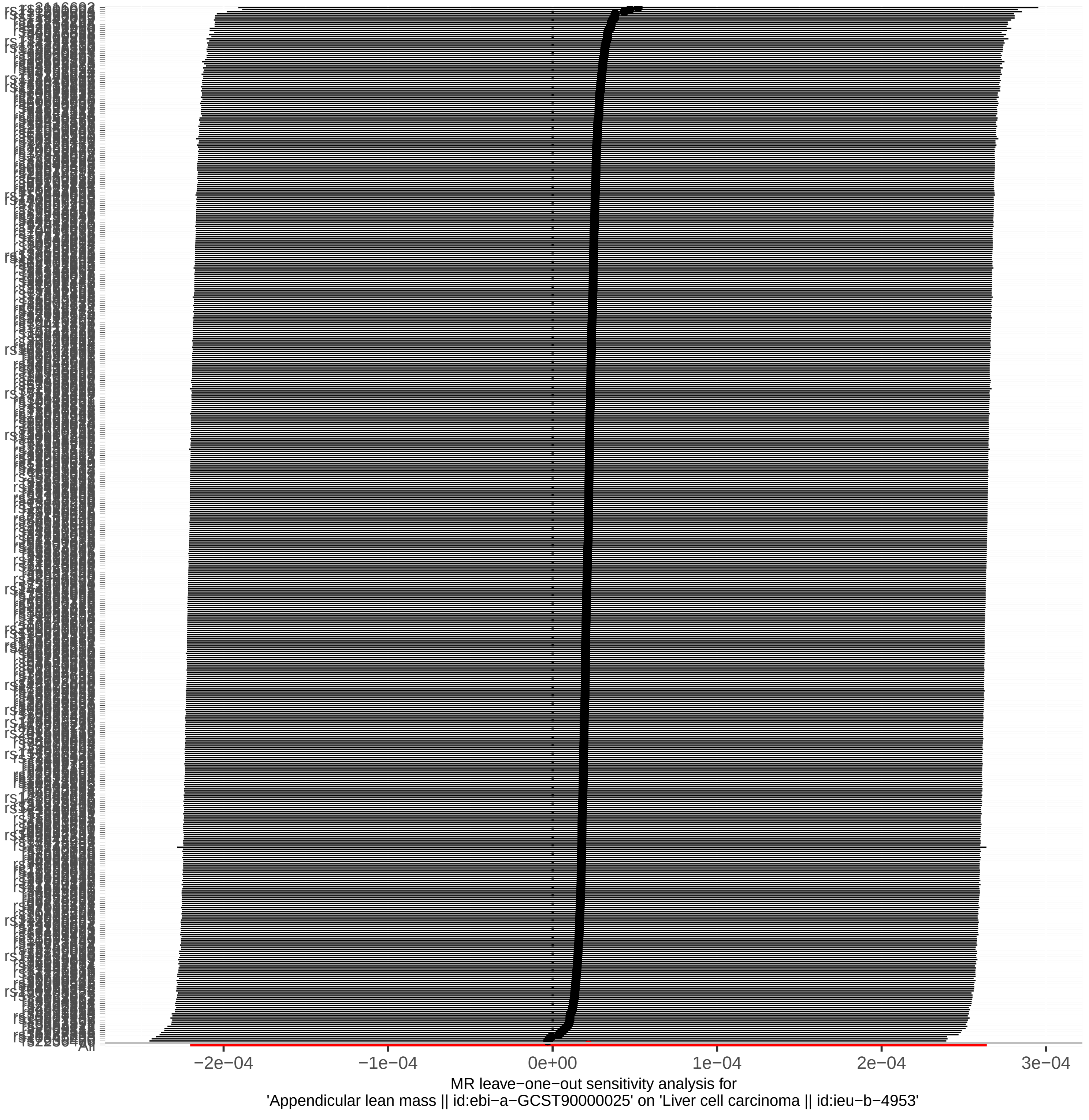

−2e−04
−1e−04
0e+00
1e−04
2e−04
3e−04
All
MR leave−one−out sensitivity analysis for
'Appendicular lean mass || id:ebi−a−GCST90000025' on 'Liver cell carcinoma || id:ieu−b−4953'

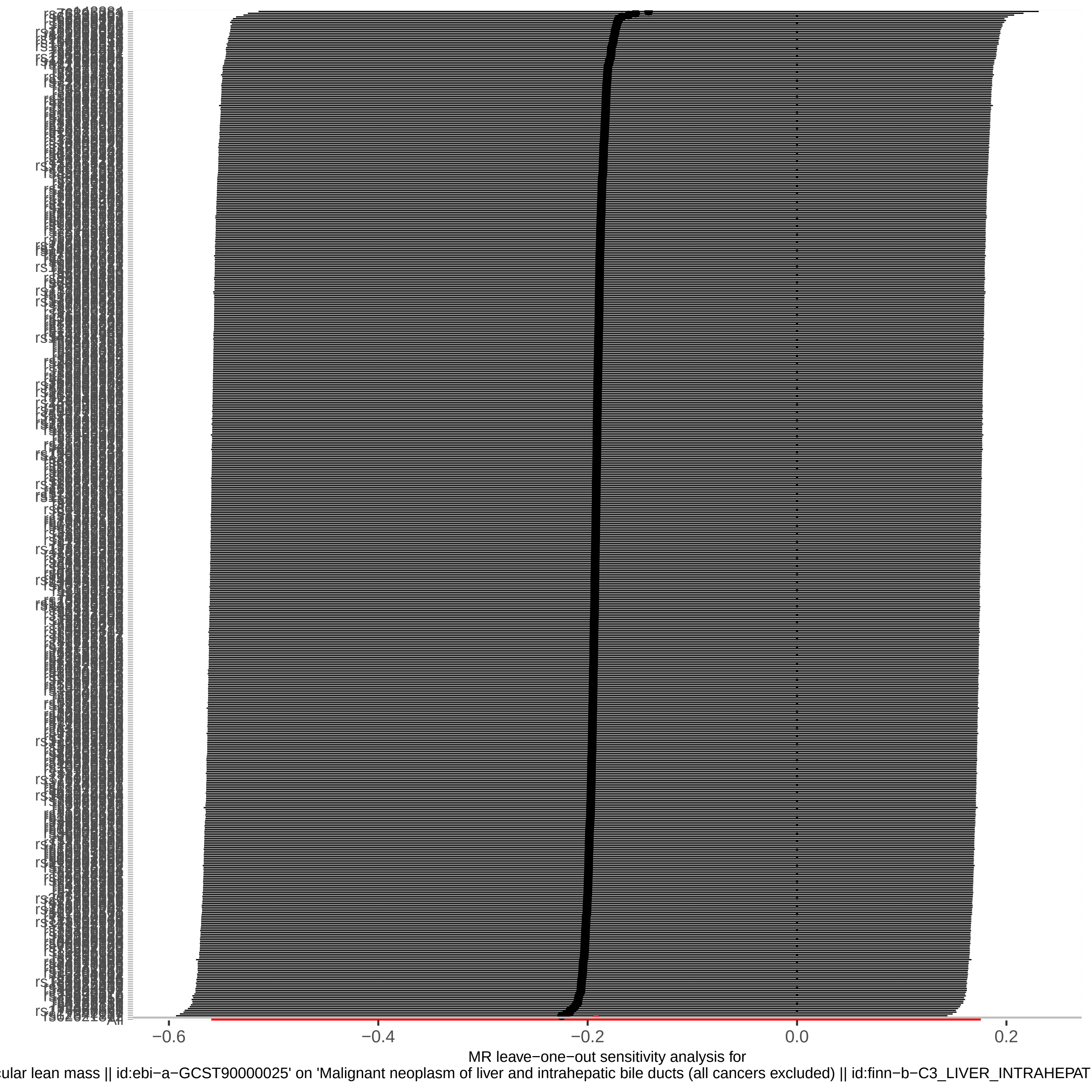

All
−0.6
−0.4
−0.2
0.0
0.2
MR leave−one−out sensitivity analysis for
cular lean mass || id:ebi−a−GCST90000025' on 'Malignant neoplasm of liver and intrahepatic bile ducts (all cancers excluded) || id:finn−b−C3_LIVER_INTRAHEPAT

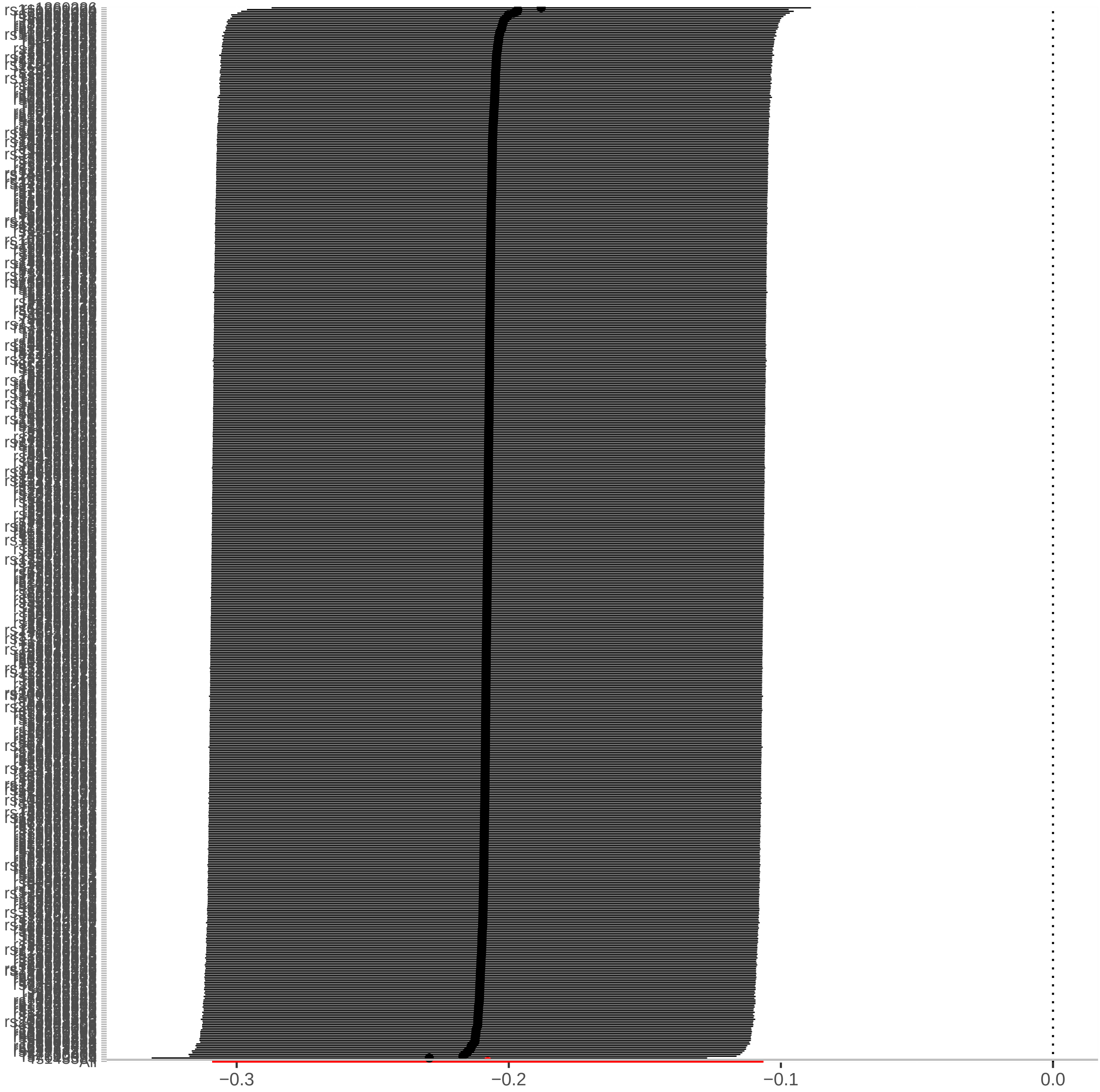

MR leave−one−out sensitivity analysis for
'Appendicular lean mass || id:ebi−a−GCST90000025' on 'Nonalcoholic fatty liver disease || id:ebi−a−GCST90054782'

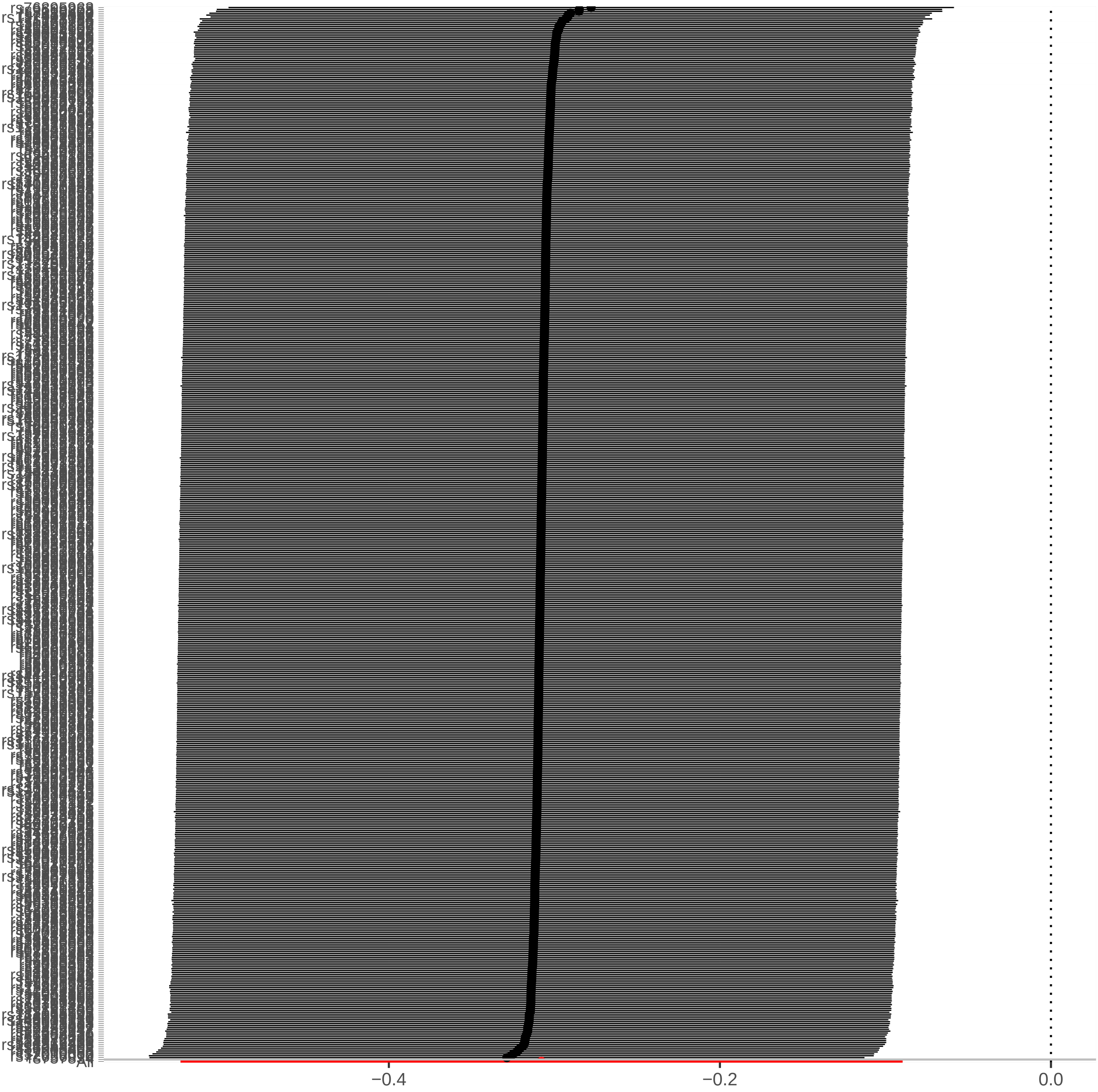

MR leave−one−out sensitivity analysis for
'Appendicular lean mass || id:ebi−a−GCST90000025' on 'Nonalcoholic fatty liver disease || id:finn−b−NAFLD'

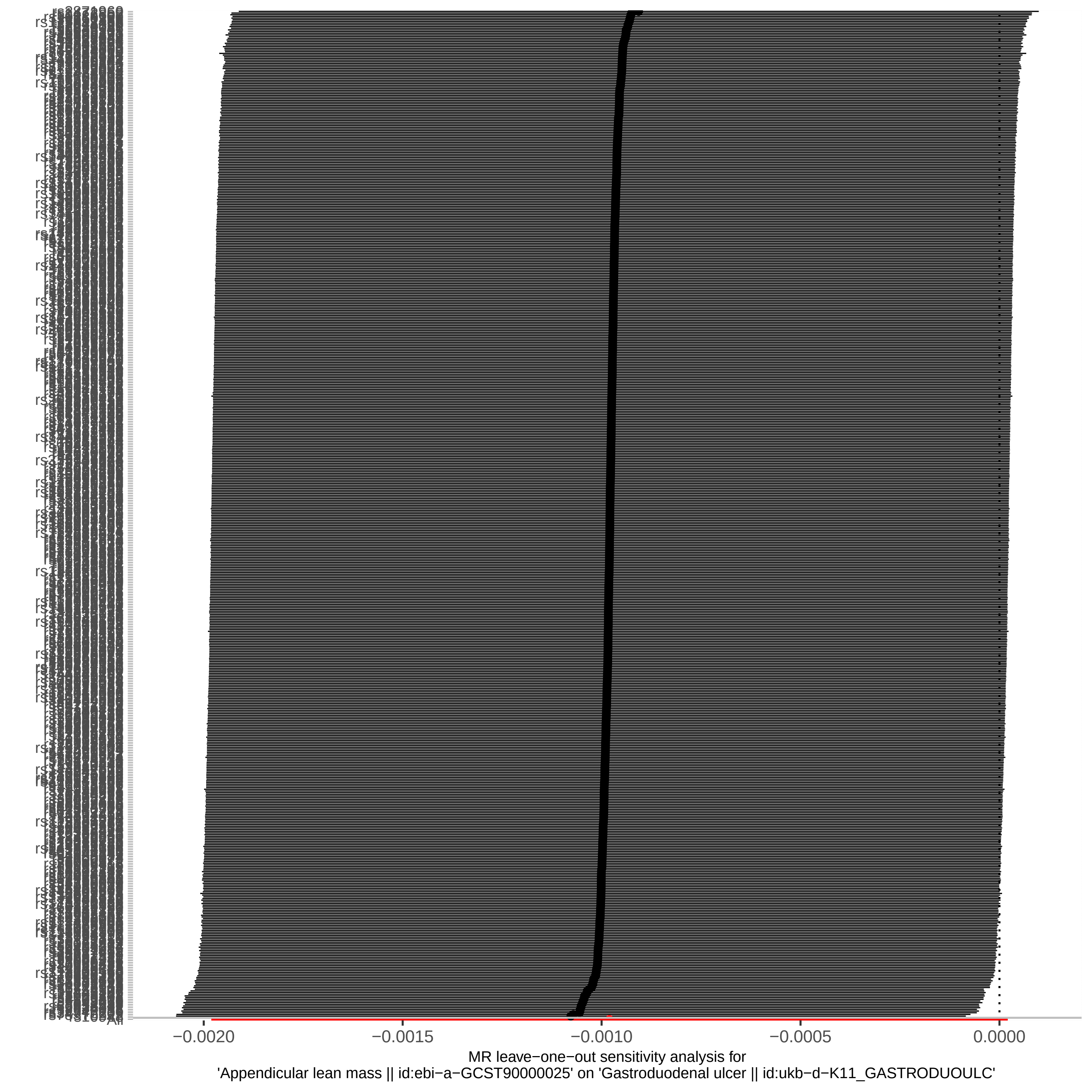

−0.0020
−0.0015
−0.0010
−0.0005
0.0000
MR leave−one−out sensitivity analysis for
'Appendicular lean mass || id:ebi−a−GCST90000025' on 'Gastroduodenal ulcer || id:ukb−d−K11_GASTRODUOULC'

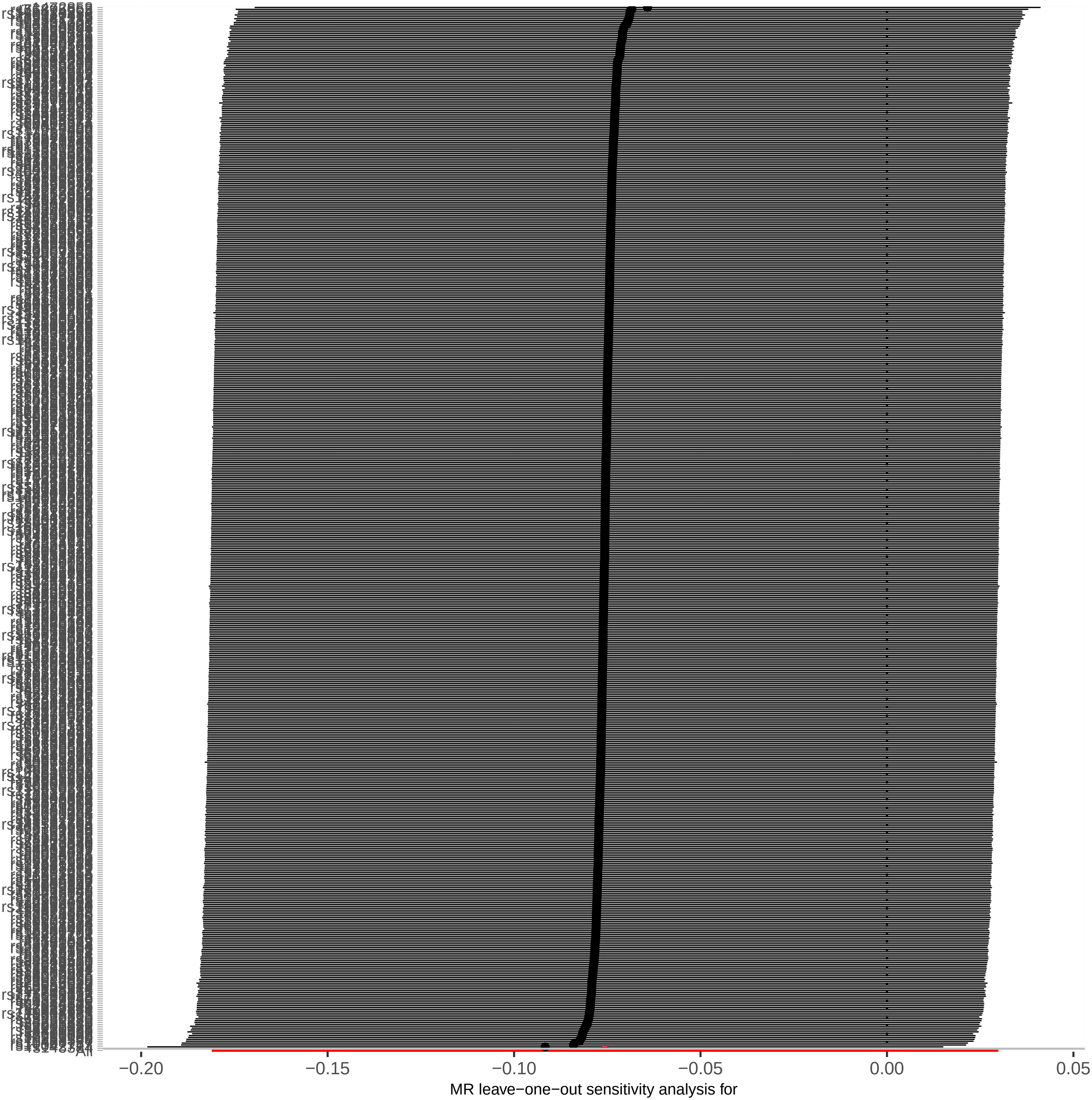

MR leave−one−out sensitivity analysis for
'Appendicular lean mass || id:ebi−a−GCST90000025' on 'Gastroduodenal ulcer || id:finn−b−K11_GASTRODUOULC'

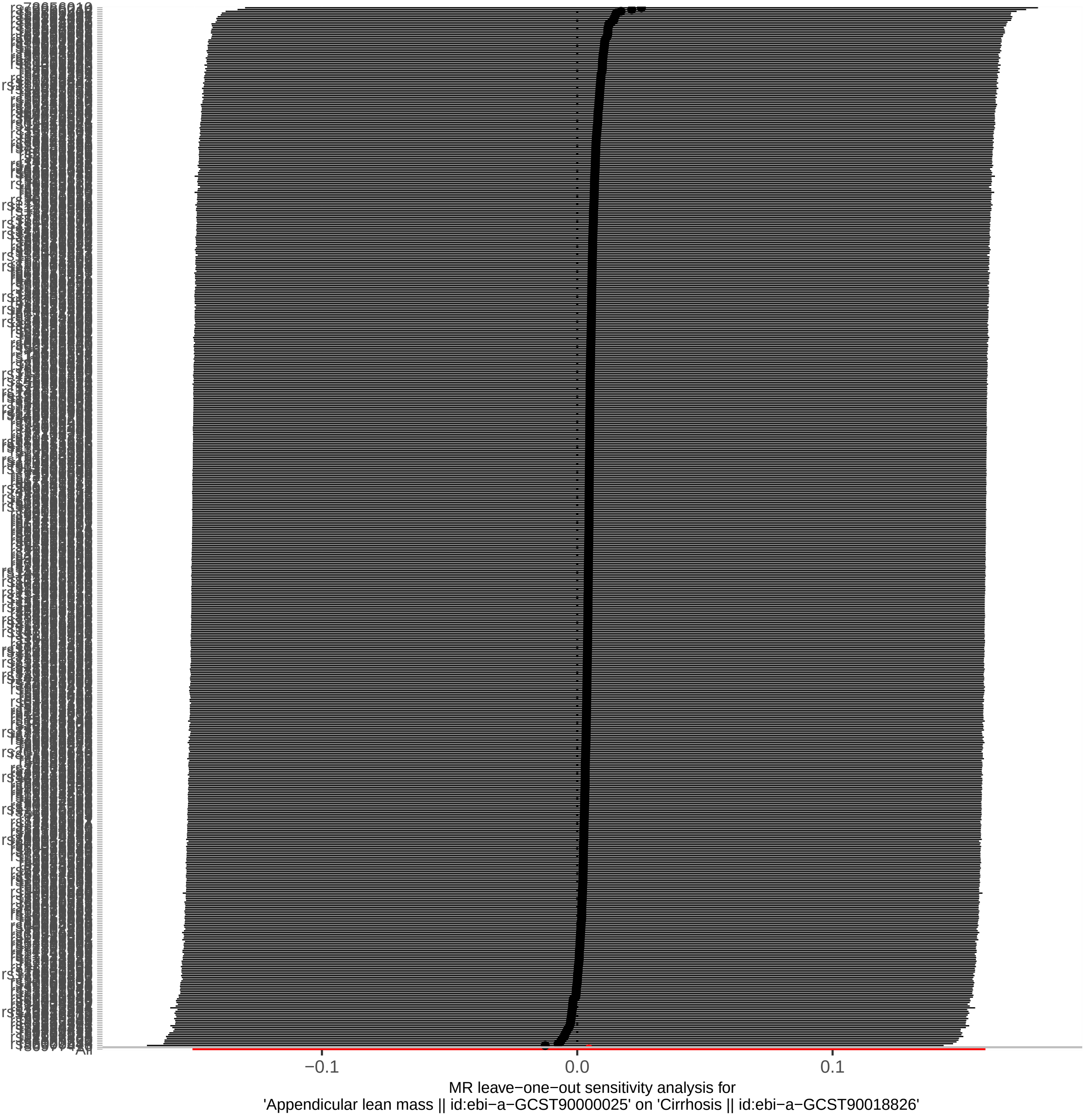

All
−0.1
0.0
0.1
MR leave−one−out sensitivity analysis for
'Appendicular lean mass || id:ebi−a−GCST90000025' on 'Cirrhosis || id:ebi−a−GCST90018826'

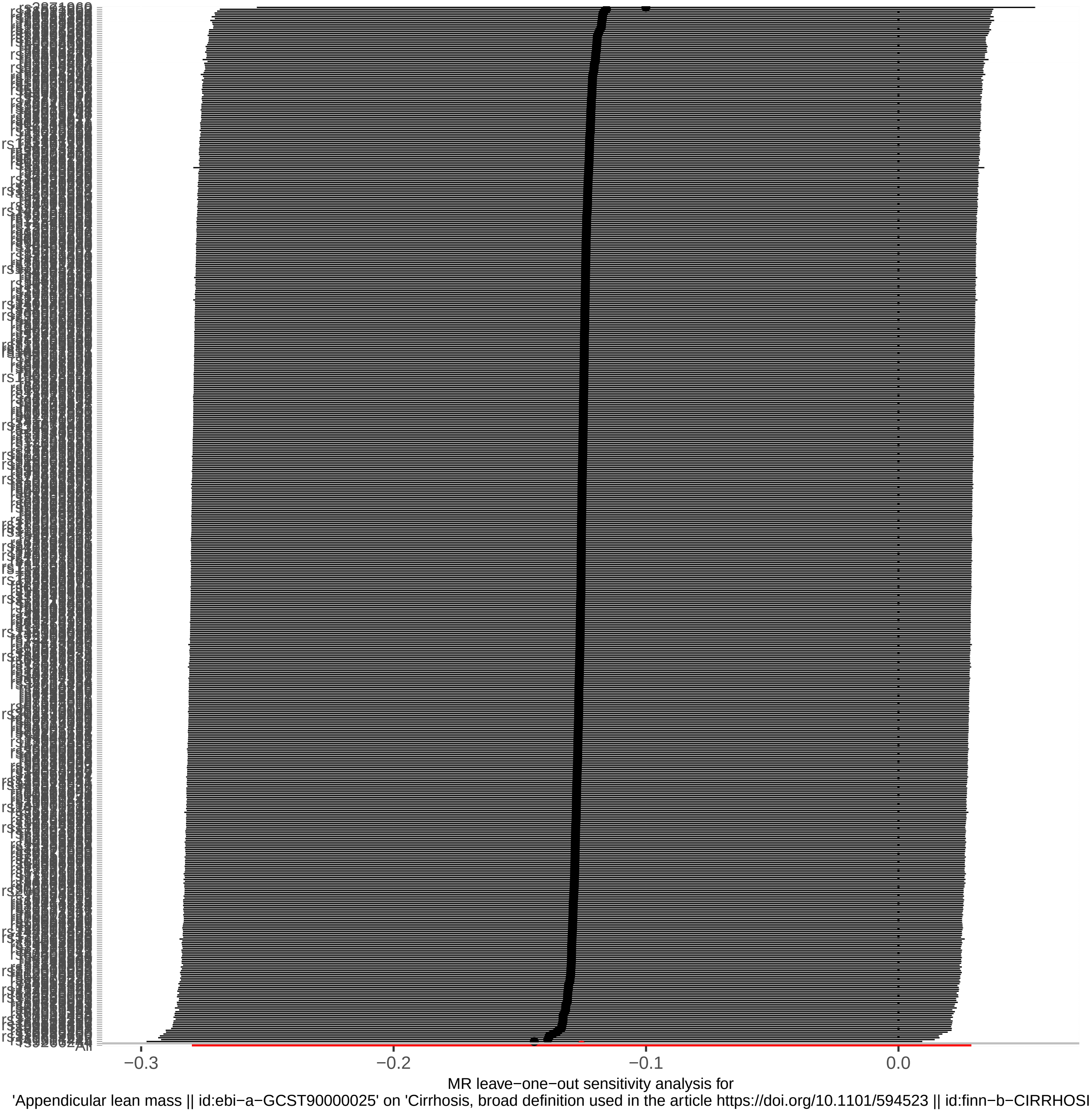

All
−0.3
−0.2
−0.1
0.0
MR leave−one−out sensitivity analysis for
'Appendicular lean mass || id:ebi−a−GCST90000025' on 'Cirrhosis, broad definition used in the article https://doi.org/10.1101/594523 || id:finn−b−CIRRHOSI

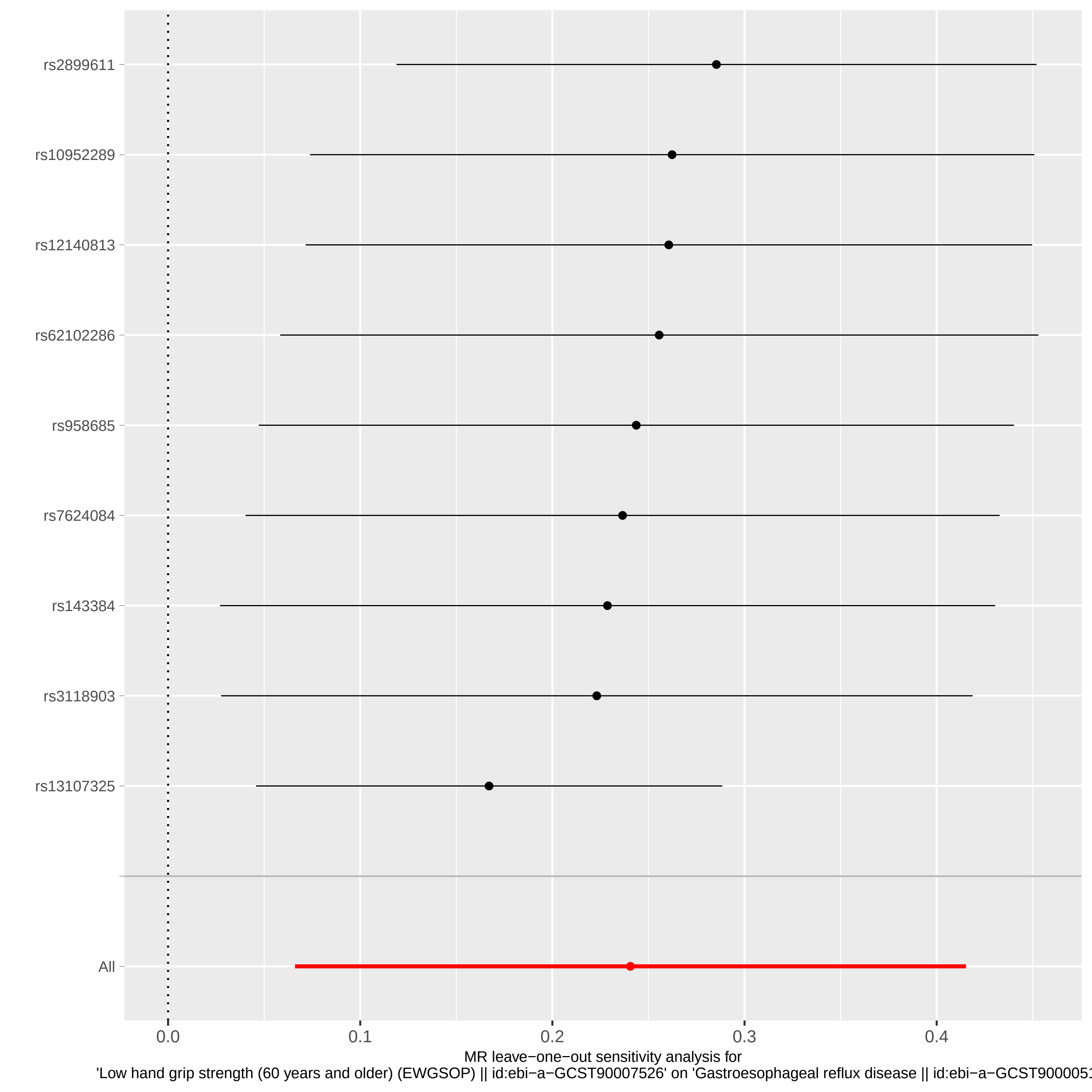

rs2899611
rs10952289
rs12140813
rs62102286
rs958685
rs7624084
rs143384
rs3118903
rs13107325
All
0.0
0.1
0.2
0.3
0.4
MR leave−one−out sensitivity analysis for
'Low hand grip strength (60 years and older) (EWGSOP) || id:ebi−a−GCST90007526' on 'Gastroesophageal reflux disease || id:ebi−a−GCST900005

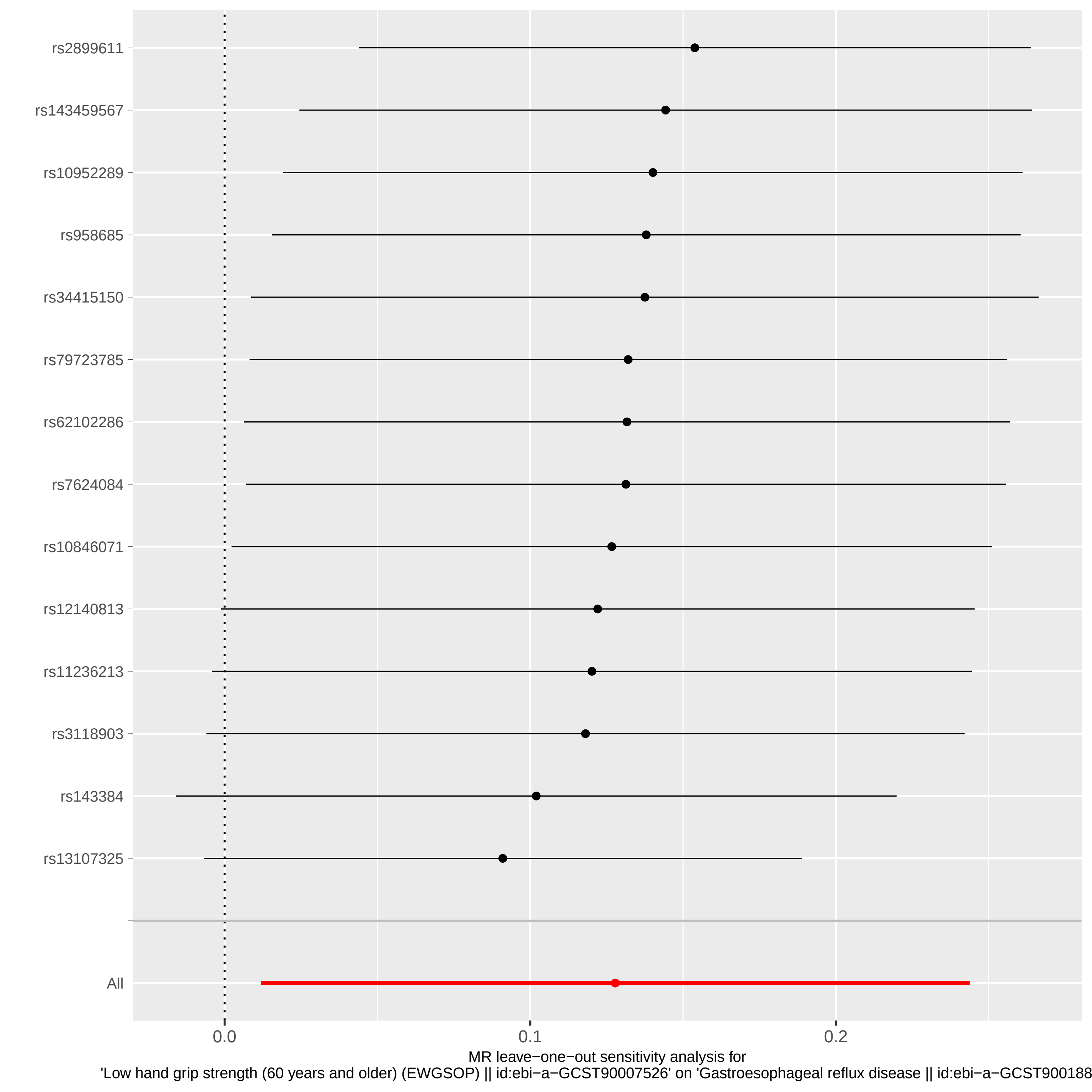
rs2899611
rs143459567
rs10952289
rs958685
rs34415150
rs79723785
rs62102286
rs7624084
rs10846071
rs12140813
rs11236213
rs3118903
rs143384
rs13107325
All
0.0
0.1
0.2
MR leave−one−out sensitivity analysis for
'Low hand grip strength (60 years and older) (EWGSOP) || id:ebi−a−GCST90007526' on 'Gastroesophageal reflux disease || id:ebi−a−GCST900188

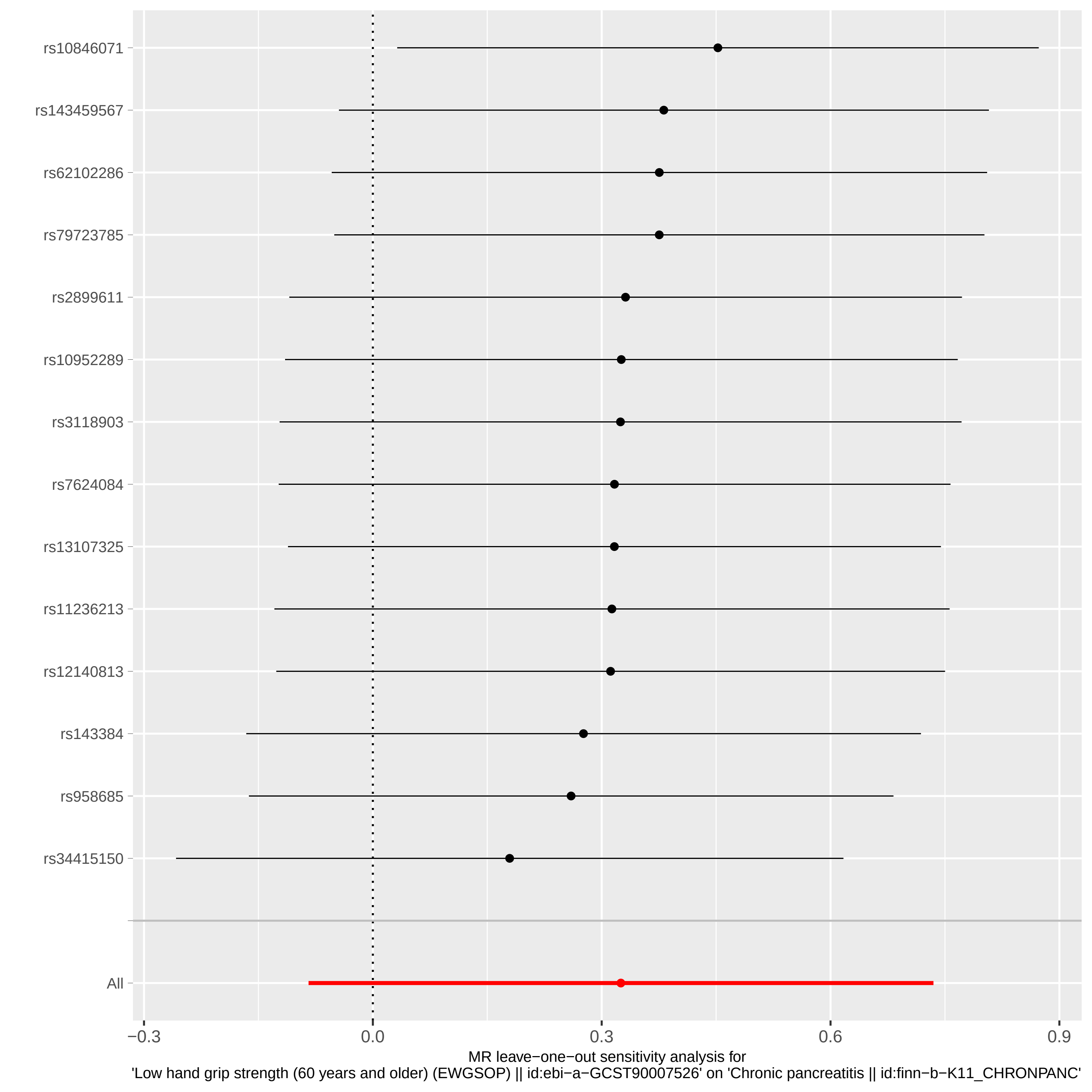
rs10846071
rs143459567
rs62102286
rs79723785
rs2899611
rs10952289
rs3118903
rs7624084
rs13107325
rs11236213
rs12140813
rs143384
rs958685
rs34415150
All
−0.3
0.0
0.3
0.6
0.9
MR leave−one−out sensitivity analysis for
'Low hand grip strength (60 years and older) (EWGSOP) || id:ebi−a−GCST90007526' on 'Chronic pancreatitis || id:finn−b−K11_CHRONPANC'

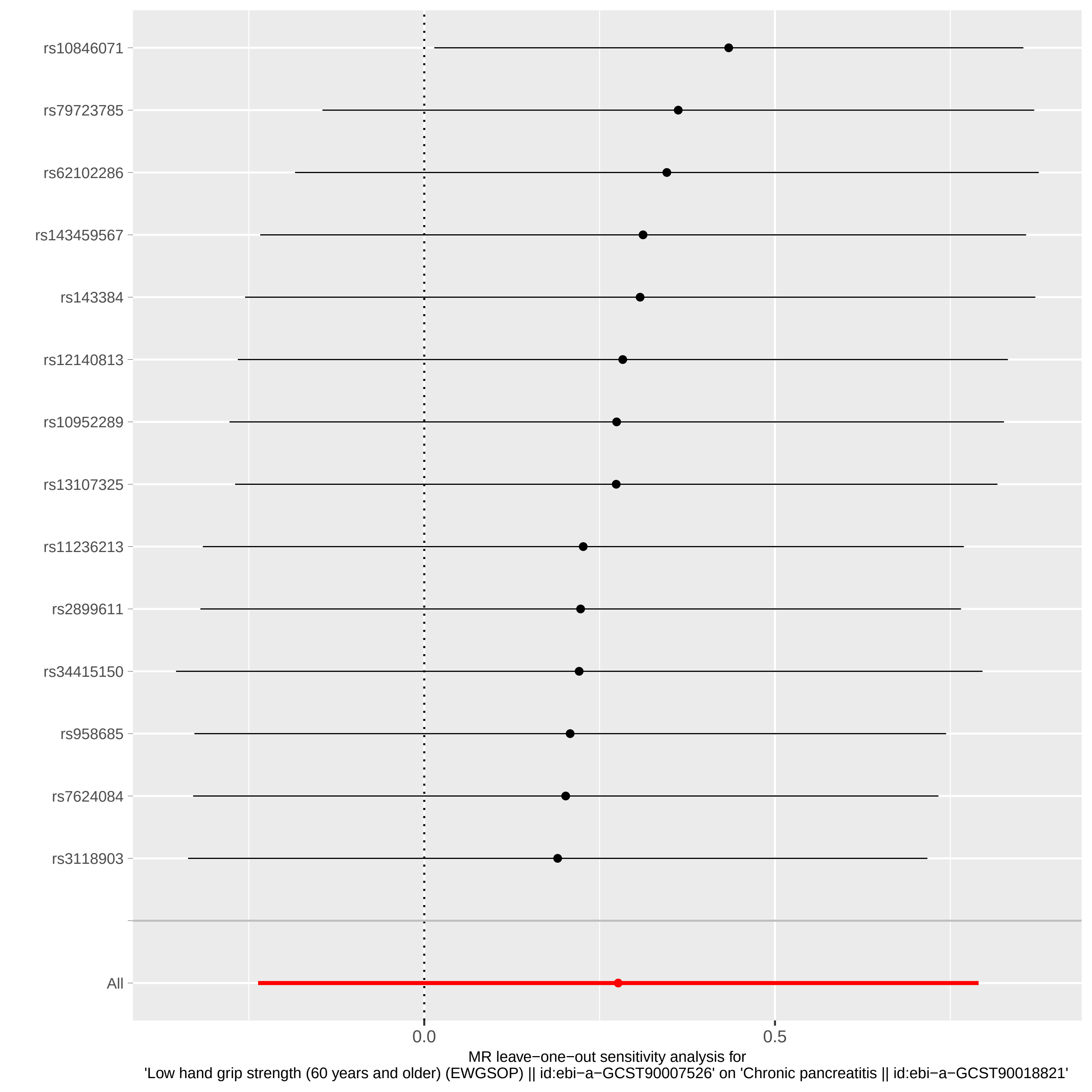
rs10846071
rs79723785
rs62102286
rs143459567
rs143384
rs12140813
rs10952289
rs13107325
rs11236213
rs2899611
rs34415150
rs958685
rs7624084
rs3118903
All
0.0
0.5
MR leave−one−out sensitivity analysis for
'Low hand grip strength (60 years and older) (EWGSOP) || id:ebi−a−GCST90007526' on 'Chronic pancreatitis || id:ebi−a−GCST90018821'

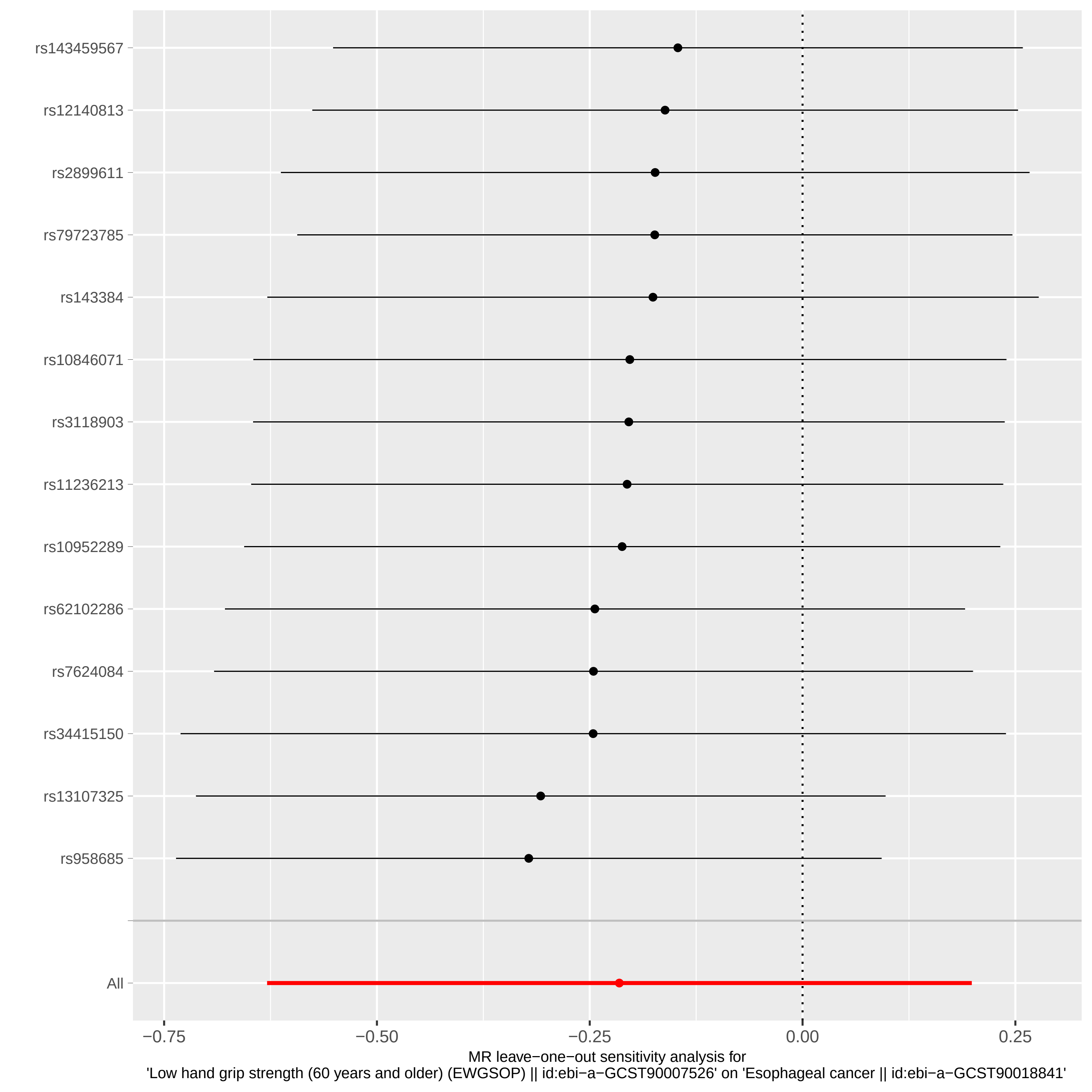
rs143459567
rs12140813
rs2899611
rs79723785
rs143384
rs10846071
rs3118903
rs11236213
rs10952289
rs62102286
rs7624084
rs34415150
rs13107325
rs958685
All
−0.75
−0.50
−0.25
0.00
0.25
MR leave−one−out sensitivity analysis for
'Low hand grip strength (60 years and older) (EWGSOP) || id:ebi−a−GCST90007526' on 'Esophageal cancer || id:ebi−a−GCST90018841'

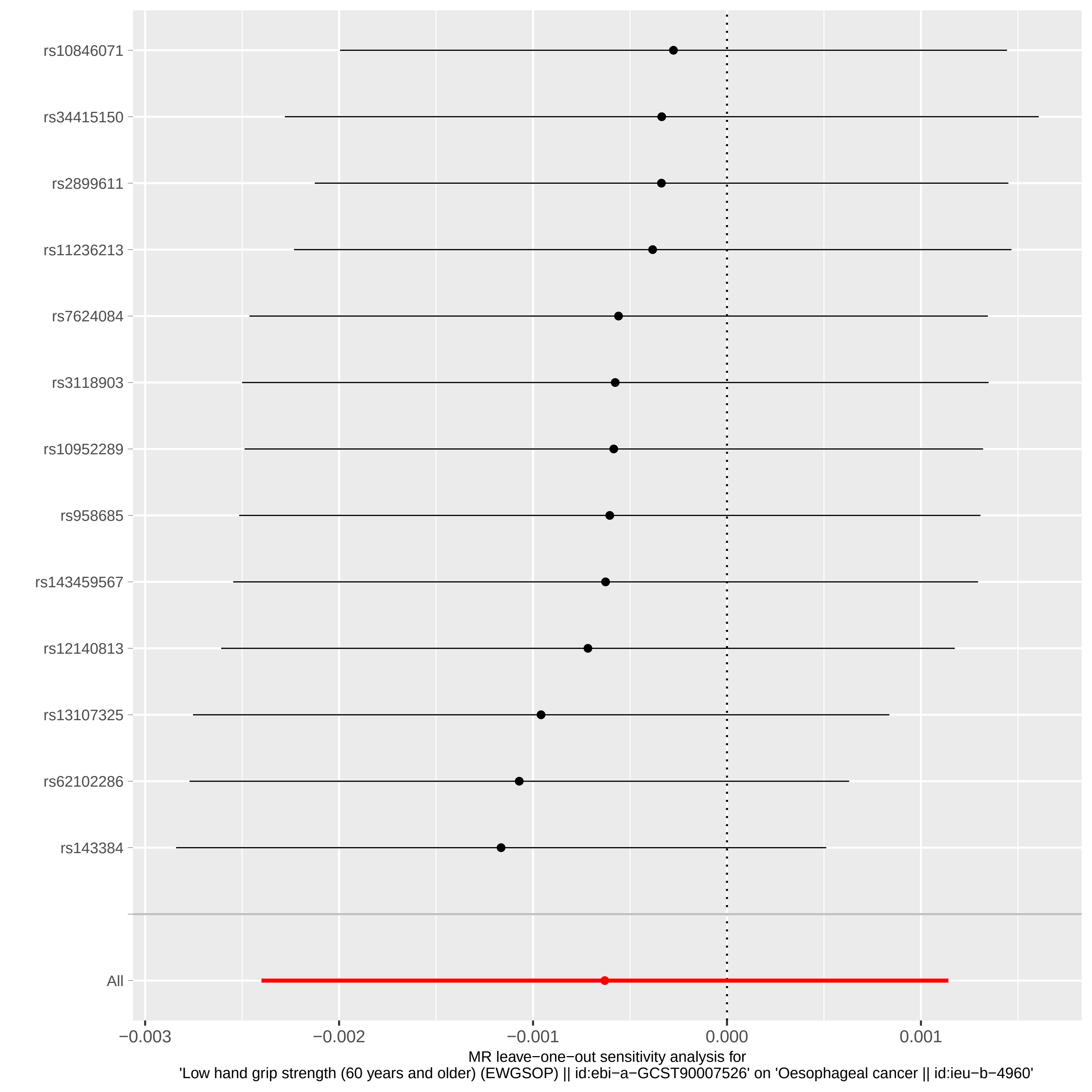
rs10846071
rs34415150
rs2899611
rs11236213
rs7624084
rs3118903
rs10952289
rs958685
rs143459567
rs12140813
rs13107325
rs62102286
rs143384
All
−0.003
−0.002
−0.001
0.000
0.001
MR leave−one−out sensitivity analysis for
'Low hand grip strength (60 years and older) (EWGSOP) || id:ebi−a−GCST90007526' on 'Oesophageal cancer || id:ieu−b−4960'

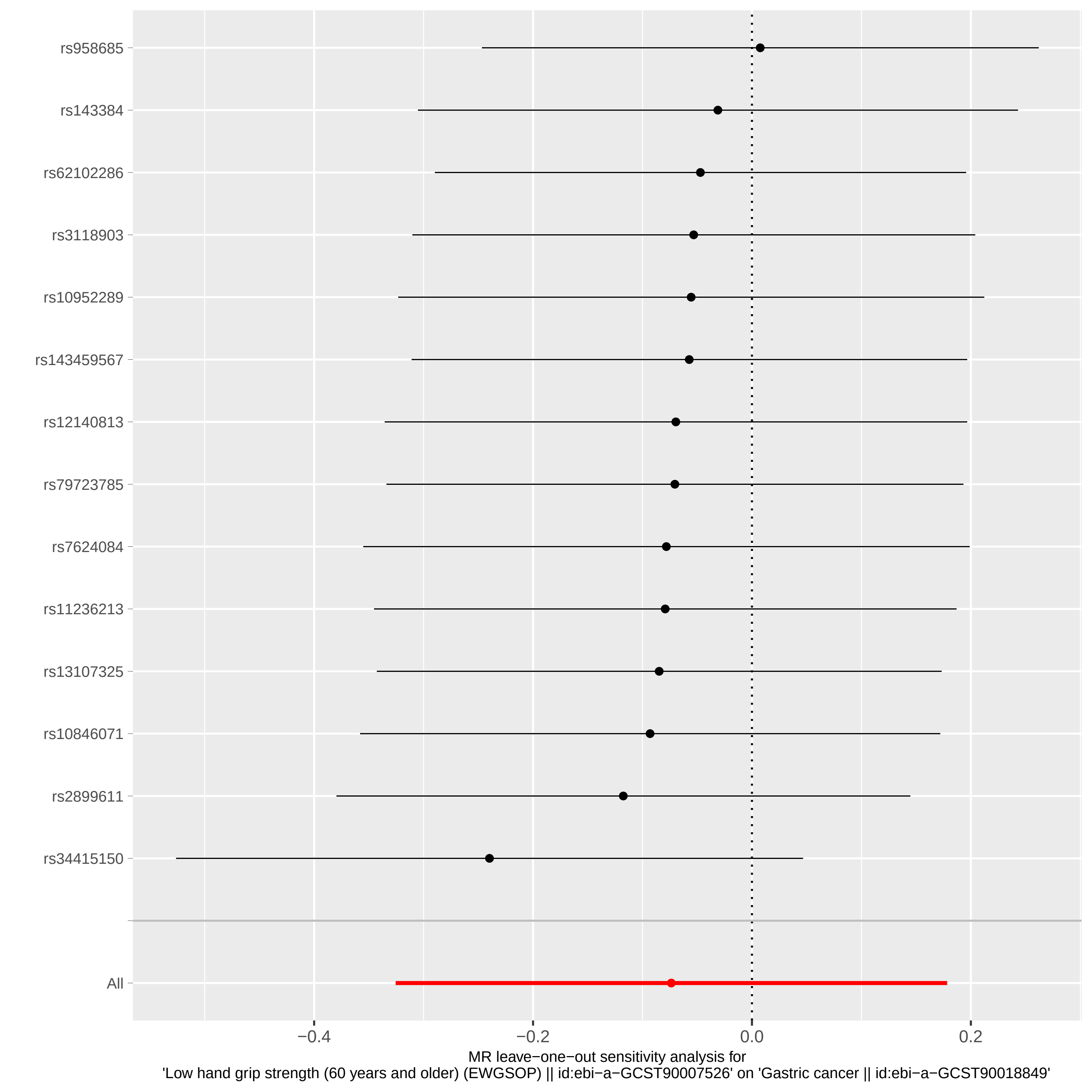
rs958685
rs143384
rs62102286
rs3118903
rs10952289
rs143459567
rs12140813
rs79723785
rs7624084
rs11236213
rs13107325
rs10846071
rs2899611
rs34415150
All
−0.4
−0.2
0.0
0.2
MR leave−one−out sensitivity analysis for
'Low hand grip strength (60 years and older) (EWGSOP) || id:ebi−a−GCST90007526' on 'Gastric cancer || id:ebi−a−GCST90018849'

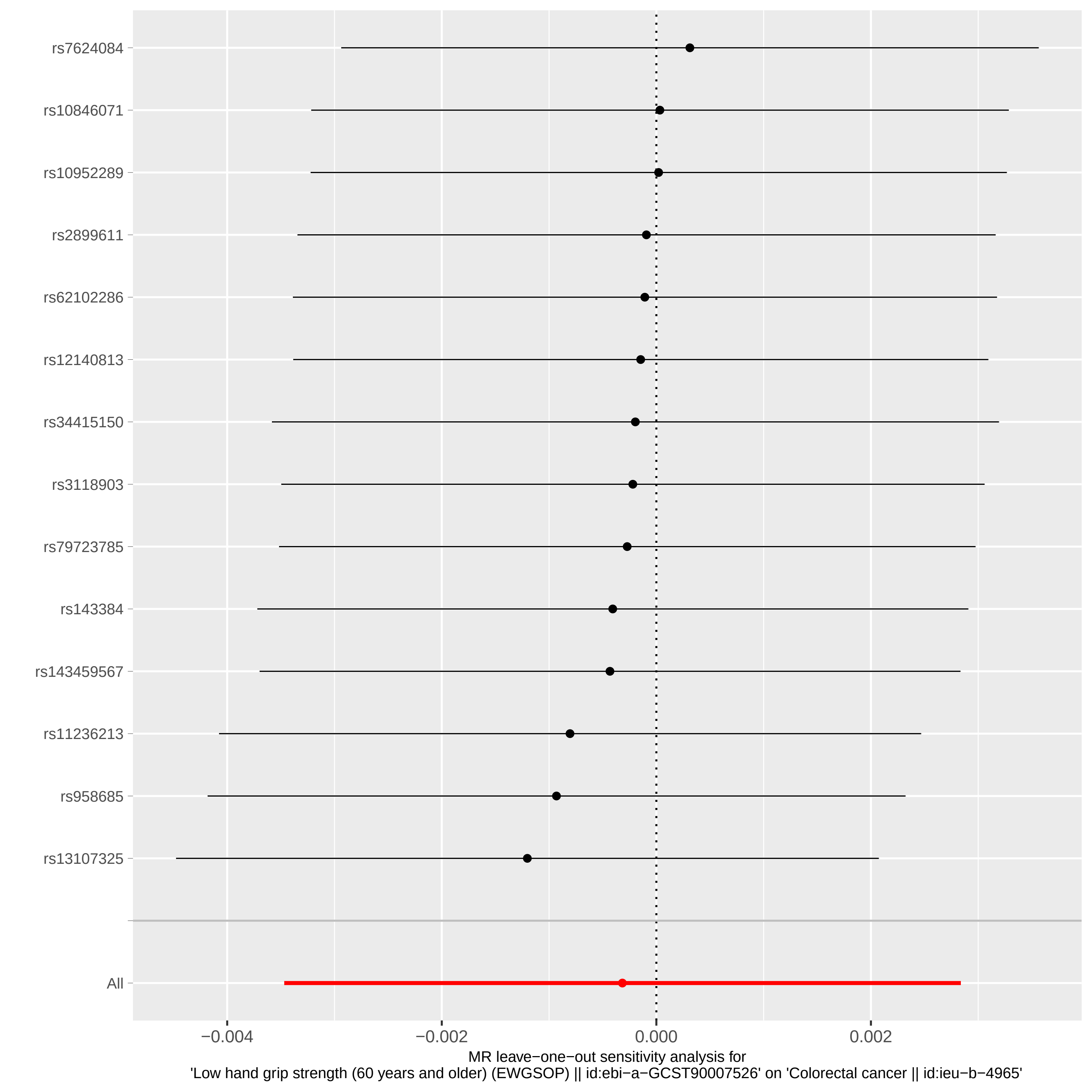
rs7624084
rs10846071
rs10952289
rs2899611
rs62102286
rs12140813
rs34415150
rs3118903
rs79723785
rs143384
rs143459567
rs11236213
rs958685
rs13107325
All
−0.004
−0.002
0.000
0.002
MR leave−one−out sensitivity analysis for
'Low hand grip strength (60 years and older) (EWGSOP) || id:ebi−a−GCST90007526' on 'Colorectal cancer || id:ieu−b−4965'

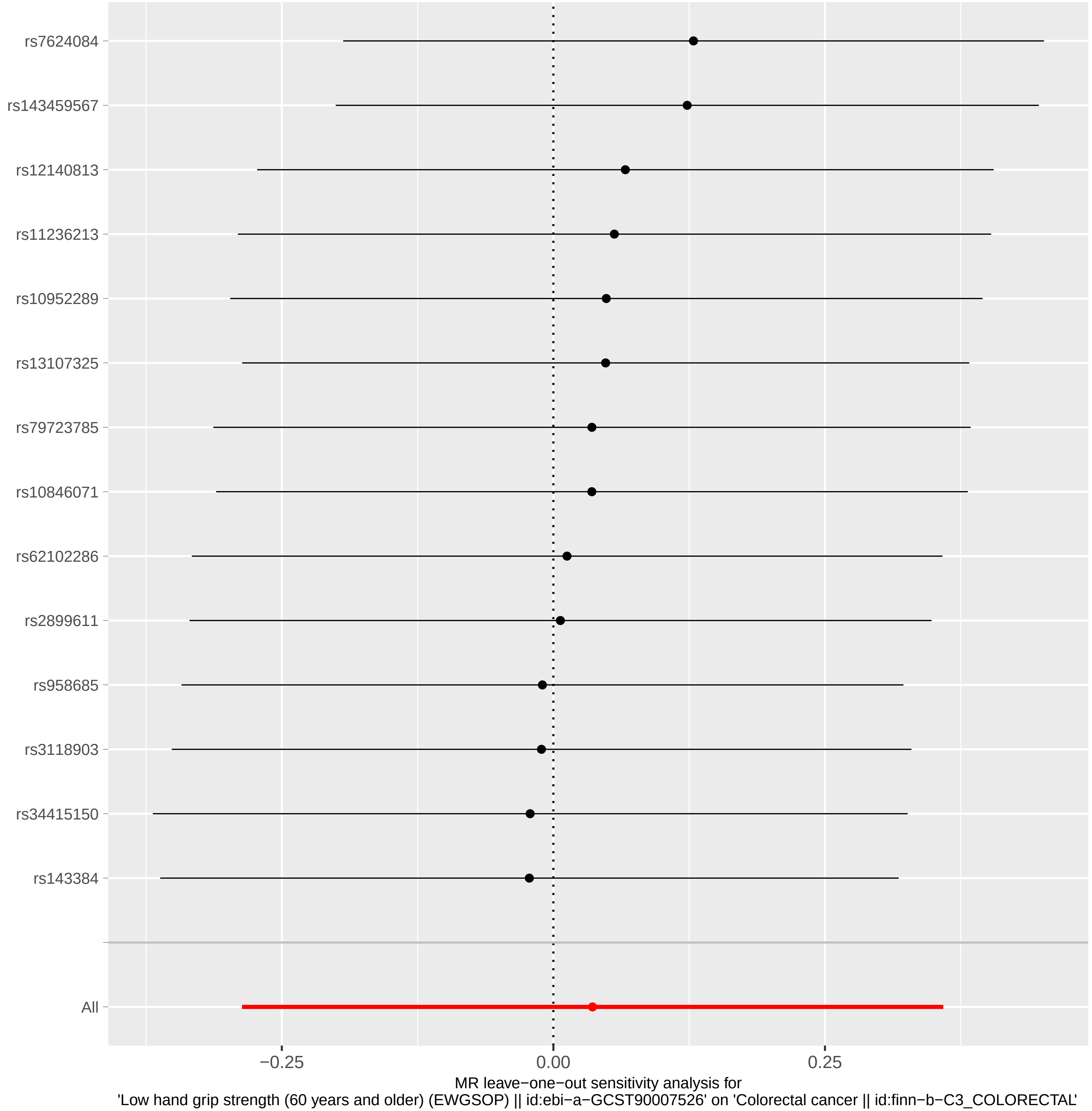

rs7624084
rs143459567
rs12140813
rs11236213
rs10952289
rs13107325
rs79723785
rs10846071
rs62102286
rs2899611
rs958685
rs3118903
rs34415150
rs143384
All
−0.25
0.00
0.25
MR leave−one−out sensitivity analysis for
'Low hand grip strength (60 years and older) (EWGSOP) || id:ebi−a−GCST90007526' on 'Colorectal cancer || id:finn−b−C3_COLORECTAL'

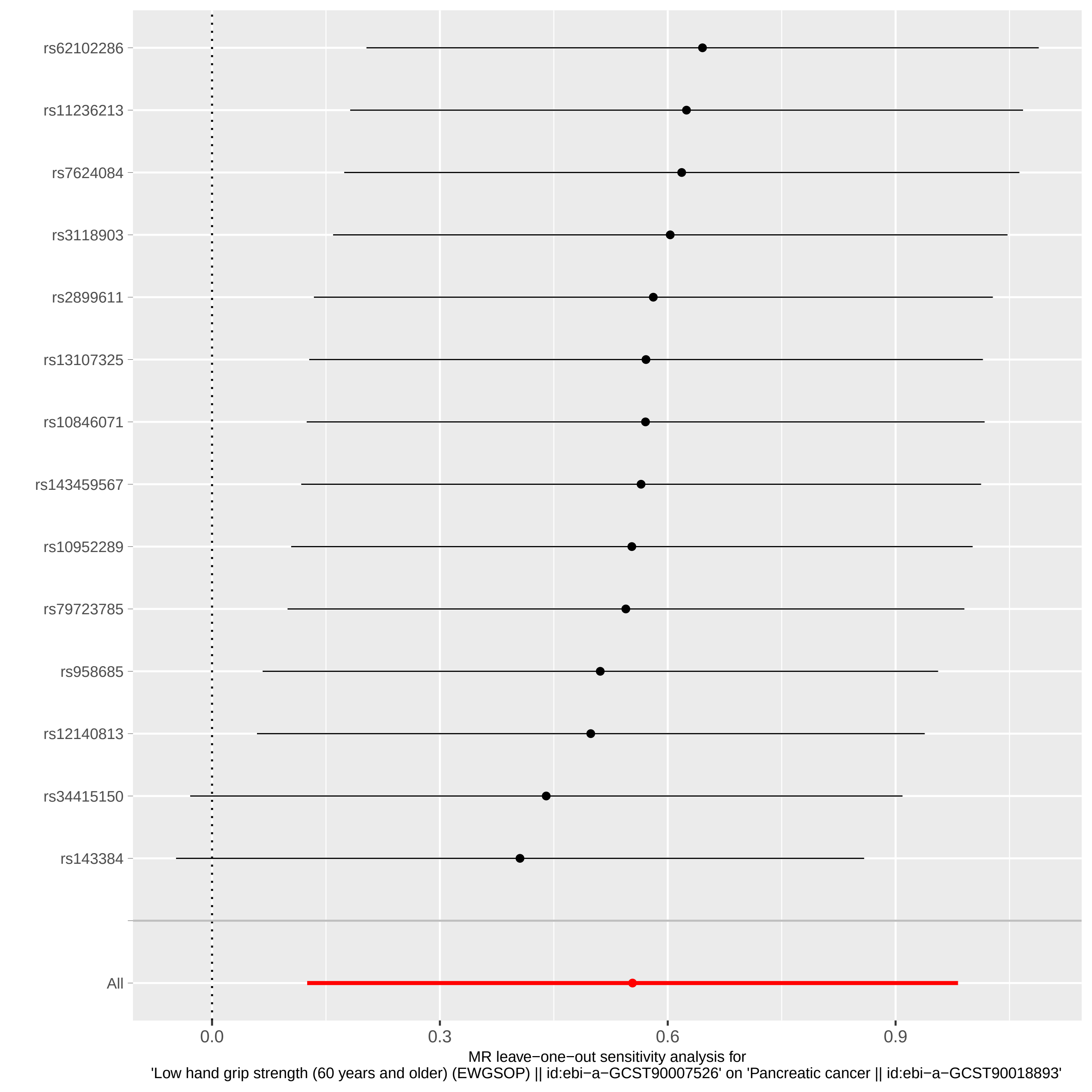
rs62102286
rs11236213
rs7624084
rs3118903
rs2899611
rs13107325
rs10846071
rs143459567
rs10952289
rs79723785
rs958685
rs12140813
rs34415150
rs143384
All
0.0
0.3
0.6
0.9
MR leave−one−out sensitivity analysis for
'Low hand grip strength (60 years and older) (EWGSOP) || id:ebi−a−GCST90007526' on 'Pancreatic cancer || id:ebi−a−GCST90018893'

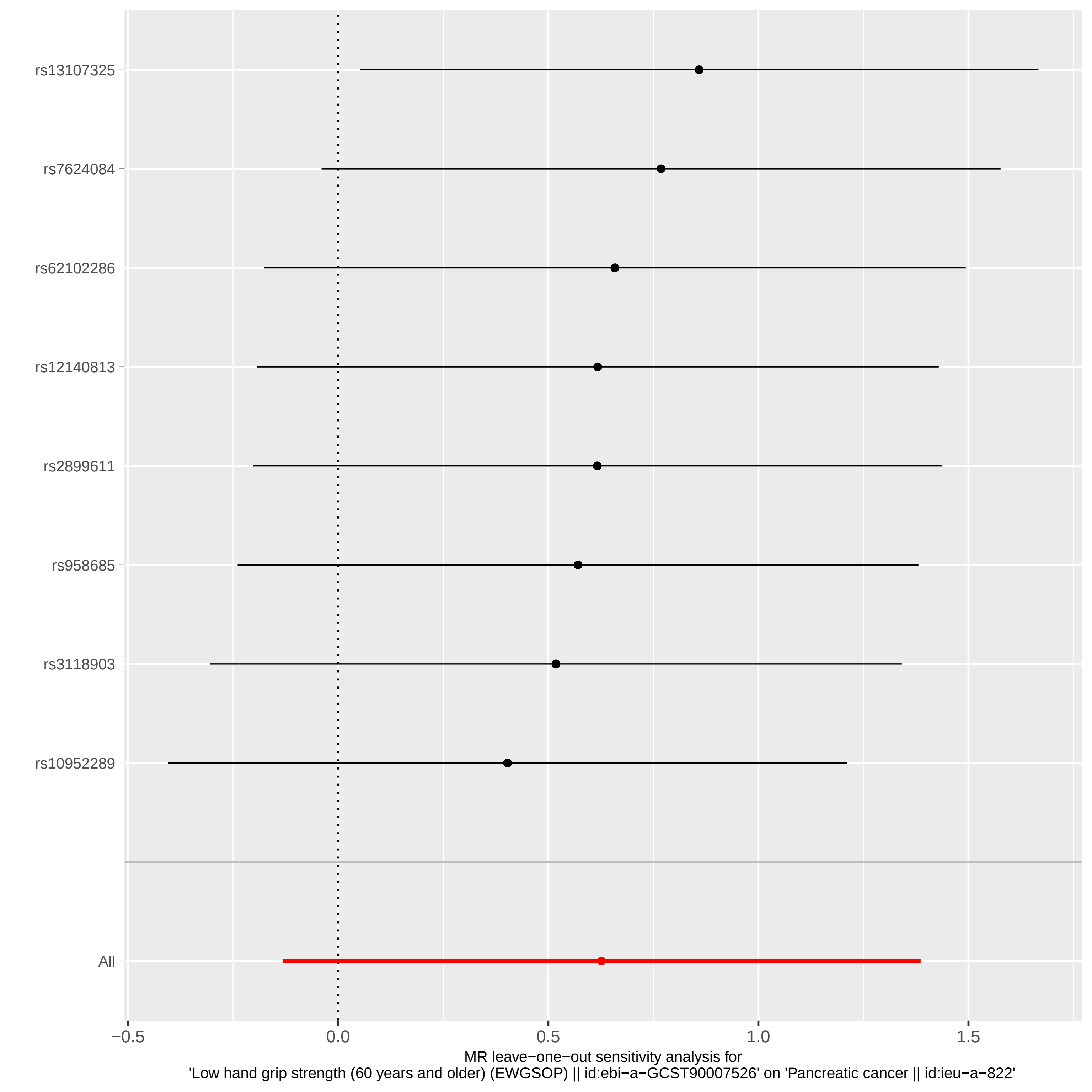
rs13107325
rs7624084
rs62102286
rs12140813
rs2899611
rs958685
rs3118903
rs10952289
All
−0.5
0.0
0.5
1.0
1.5
MR leave−one−out sensitivity analysis for
'Low hand grip strength (60 years and older) (EWGSOP) || id:ebi−a−GCST90007526' on 'Pancreatic cancer || id:ieu−a−822'

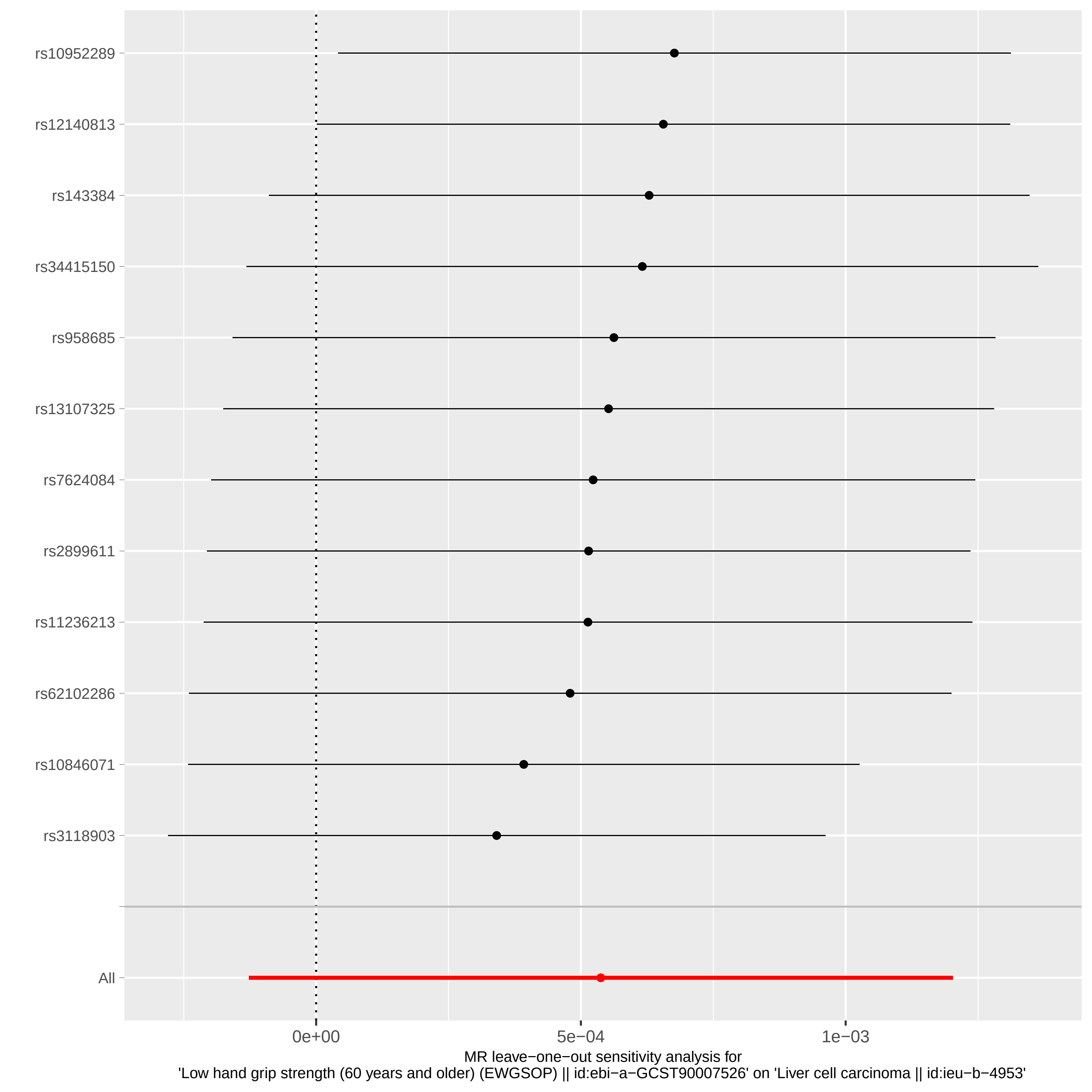
rs10952289
rs12140813
rs143384
rs34415150
rs958685
rs13107325
rs7624084
rs2899611
rs11236213
rs62102286
rs10846071
rs3118903
All
0e+00
5e−04
1e−03
MR leave−one−out sensitivity analysis for
'Low hand grip strength (60 years and older) (EWGSOP) || id:ebi−a−GCST90007526' on 'Liver cell carcinoma || id:ieu−b−4953'

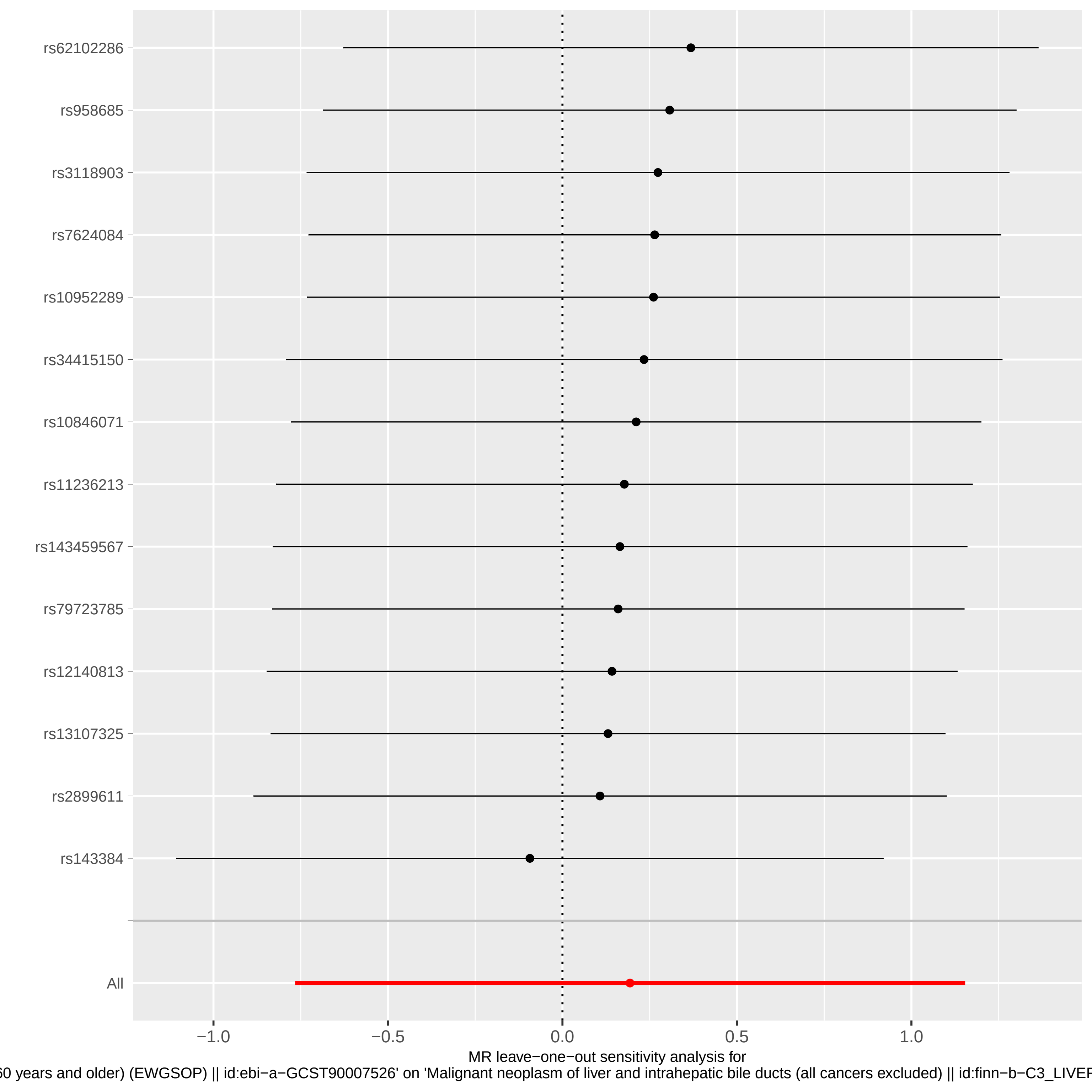
rs62102286
rs958685
rs3118903
rs7624084
rs10952289
rs34415150
rs10846071
rs11236213
rs143459567
rs79723785
rs12140813
rs13107325
rs2899611
rs143384
All
−1.0
−0.5
0.0
0.5
1.0
MR leave−one−out sensitivity analysis for
60 years and older) (EWGSOP) || id:ebi−a−GCST90007526' on 'Malignant neoplasm of liver and intrahepatic bile ducts (all cancers excluded) || id:finn−b−C3_LIVEF

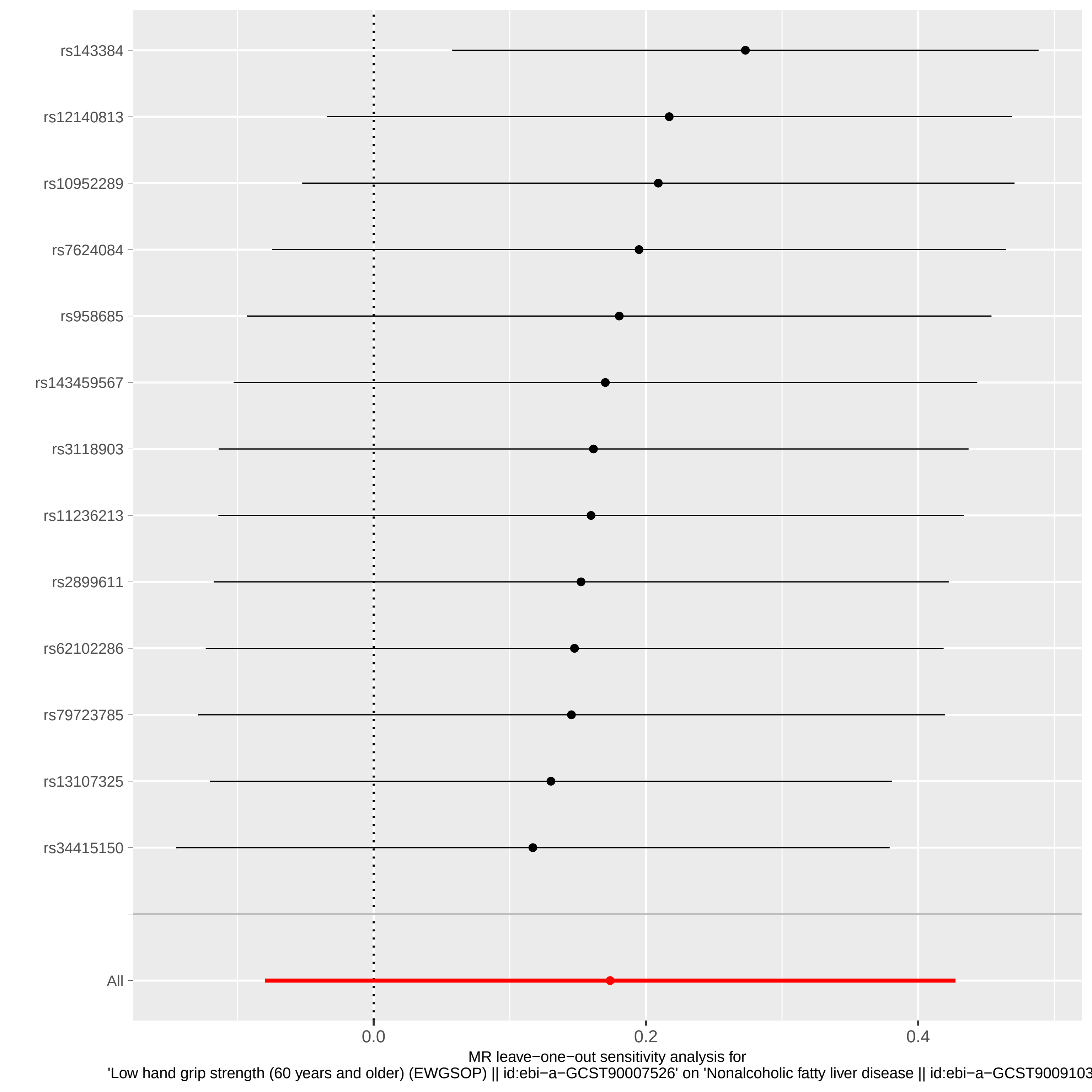
rs143384
rs12140813
rs10952289
rs7624084
rs958685
rs143459567
rs3118903
rs11236213
rs2899611
rs62102286
rs79723785
rs13107325
rs34415150
All
0.0
0.2
0.4
MR leave−one−out sensitivity analysis for
'Low hand grip strength (60 years and older) (EWGSOP) || id:ebi−a−GCST90007526' on 'Nonalcoholic fatty liver disease || id:ebi−a−GCST9009103

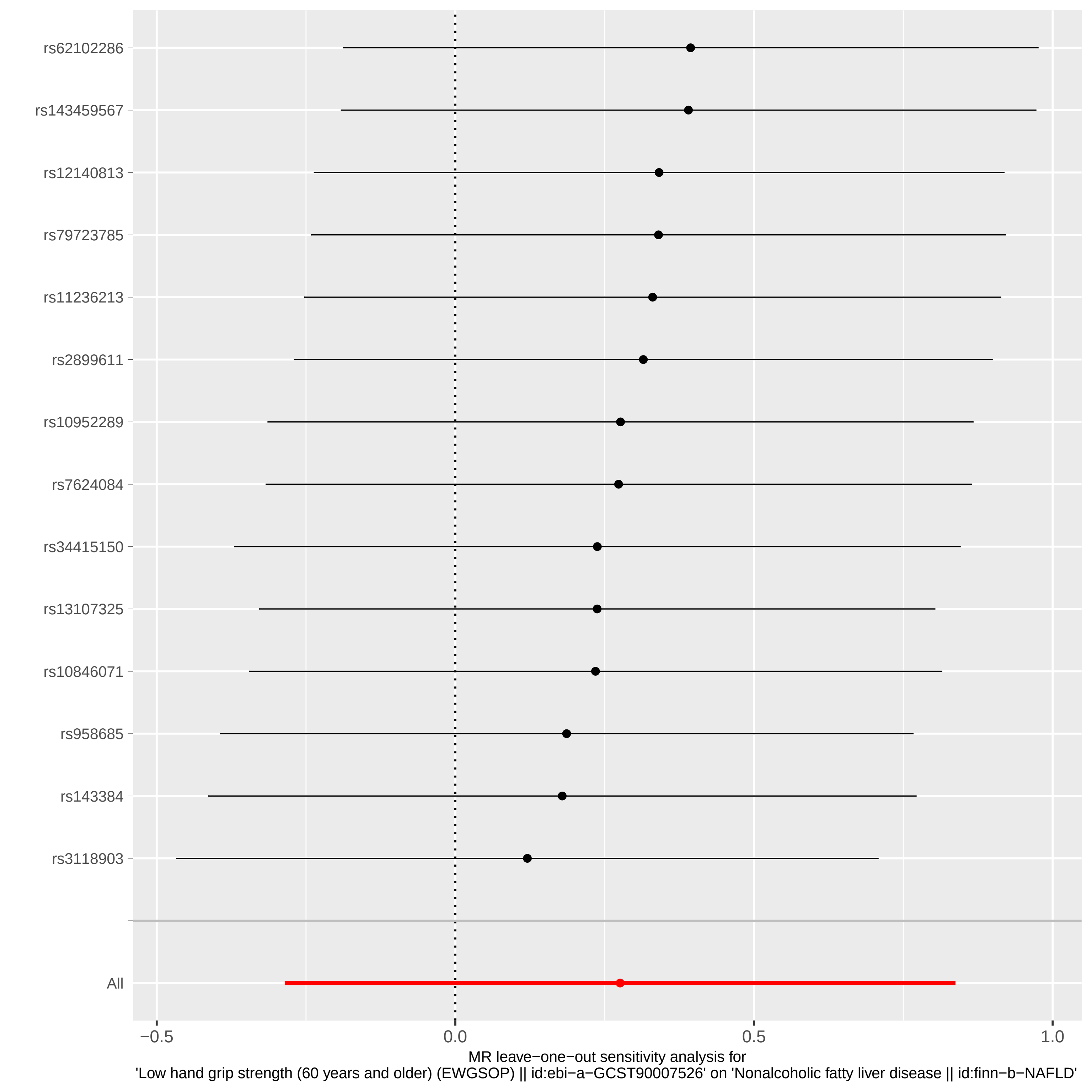
rs62102286
rs143459567
rs12140813
rs79723785
rs11236213
rs2899611
rs10952289
rs7624084
rs34415150
rs13107325
rs10846071
rs958685
rs143384
rs3118903
All
−0.5
0.0
0.5
1.0
MR leave−one−out sensitivity analysis for
'Low hand grip strength (60 years and older) (EWGSOP) || id:ebi−a−GCST90007526' on 'Nonalcoholic fatty liver disease || id:finn−b−NAFLD'

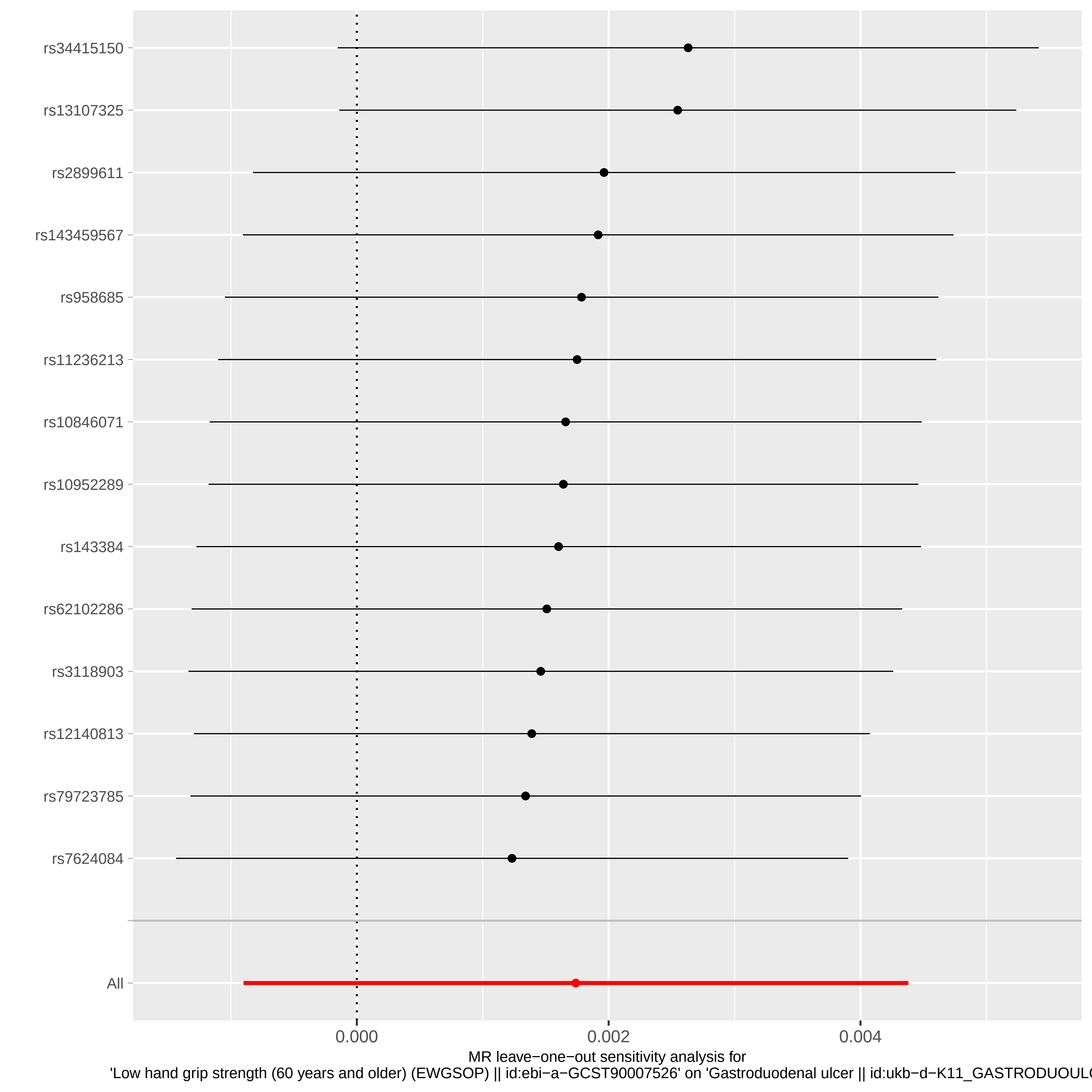

rs34415150
rs13107325
rs2899611
rs143459567
rs958685
rs11236213
rs10846071
rs10952289
rs143384
rs62102286
rs3118903
rs12140813
rs79723785
rs7624084
All
0.000
0.002
0.004
MR leave−one−out sensitivity analysis for
'Low hand grip strength (60 years and older) (EWGSOP) || id:ebi−a−GCST90007526' on 'Gastroduodenal ulcer || id:ukb−d−K11_GASTRODUOUL

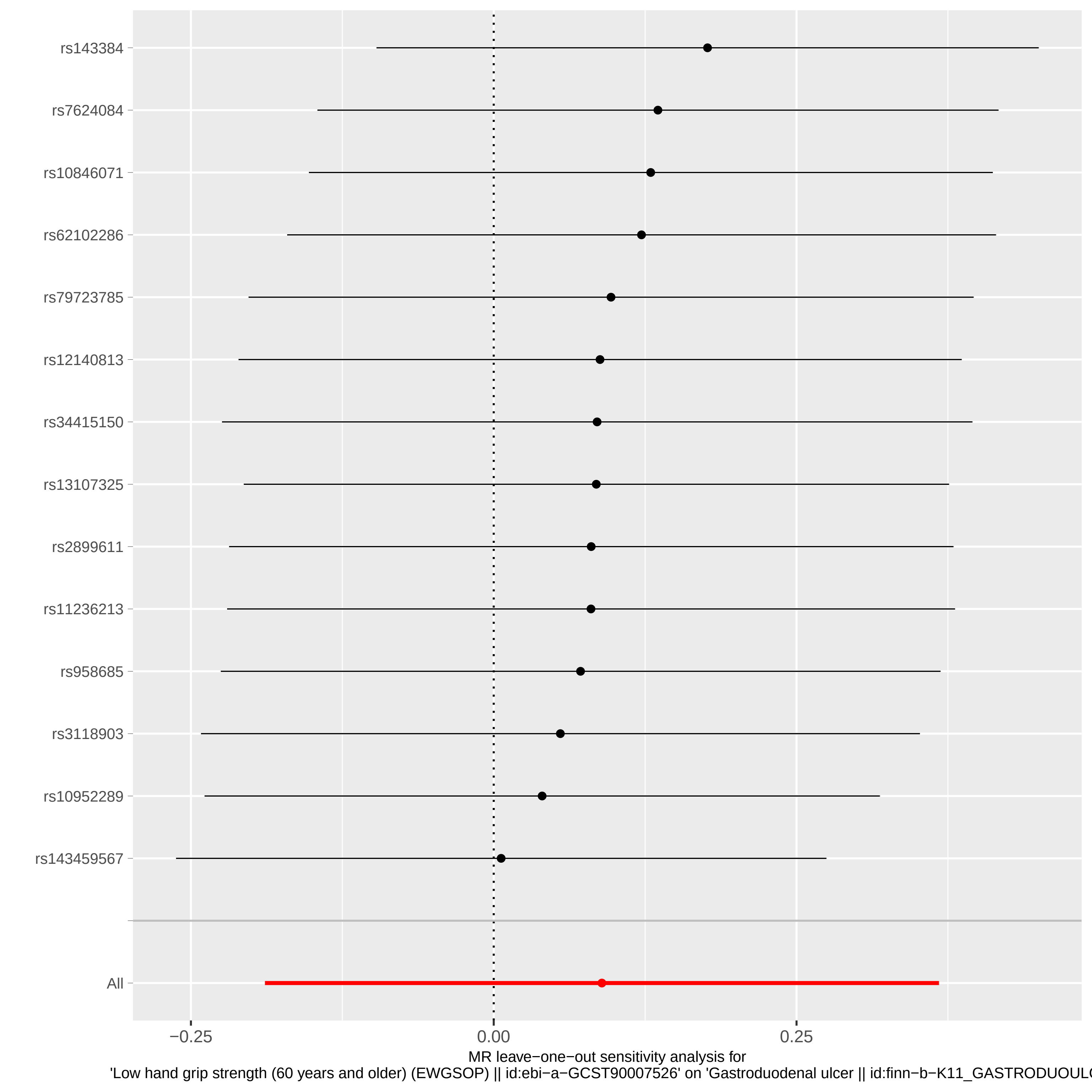
rs143384
rs7624084
rs10846071
rs62102286
rs79723785
rs12140813
rs34415150
rs13107325
rs2899611
rs11236213
rs958685
rs3118903
rs10952289
rs143459567
All
−0.25
0.00
0.25
MR leave−one−out sensitivity analysis for
'Low hand grip strength (60 years and older) (EWGSOP) || id:ebi−a−GCST90007526' on 'Gastroduodenal ulcer || id:finn−b−K11_GASTRODUOUL

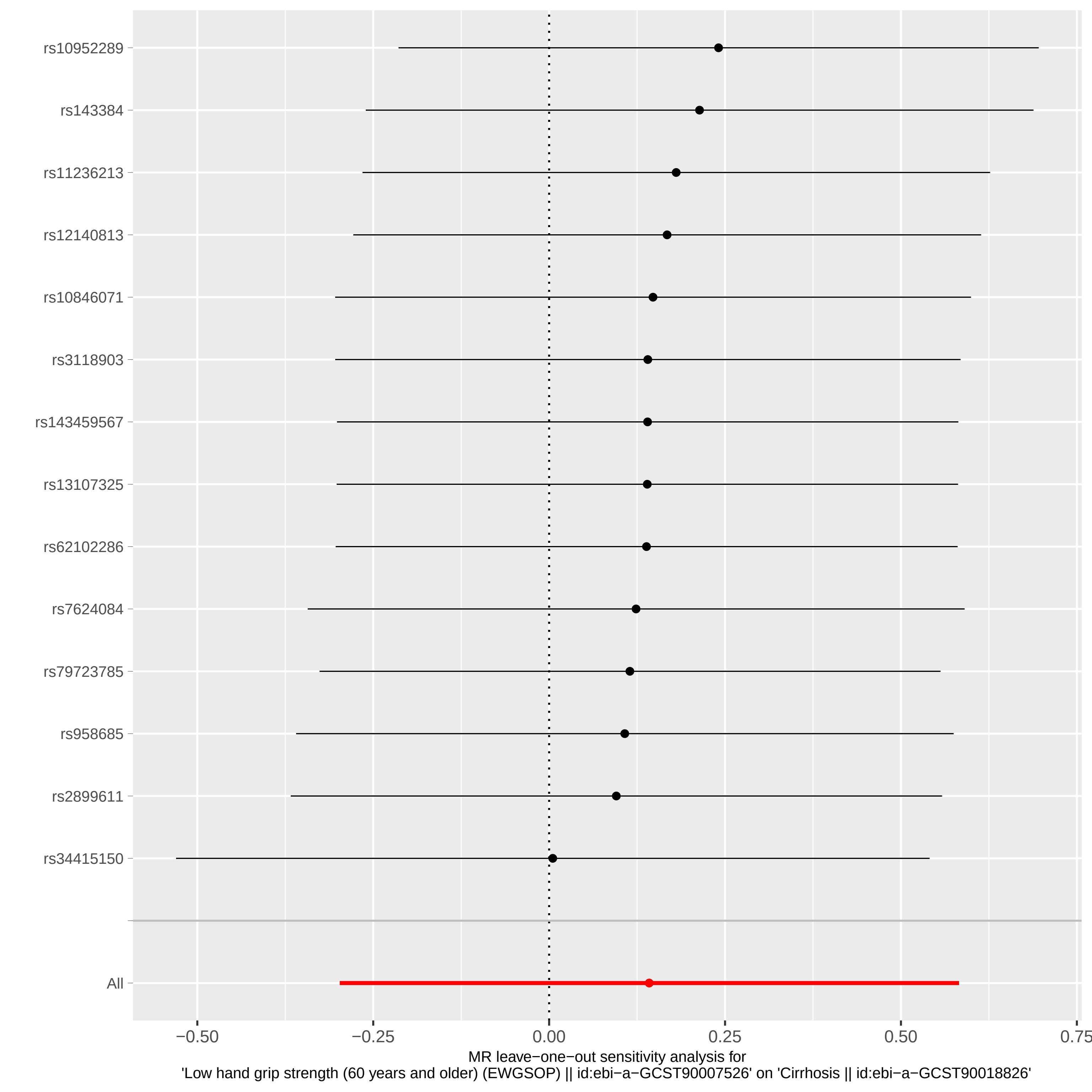
rs10952289
rs143384
rs11236213
rs12140813
rs10846071
rs3118903
rs143459567
rs13107325
rs62102286
rs7624084
rs79723785
rs958685
rs2899611
rs34415150
All
−0.50
−0.25
0.00
0.25
0.50
0.75
MR leave−one−out sensitivity analysis for
'Low hand grip strength (60 years and older) (EWGSOP) || id:ebi−a−GCST90007526' on 'Cirrhosis || id:ebi−a−GCST90018826'

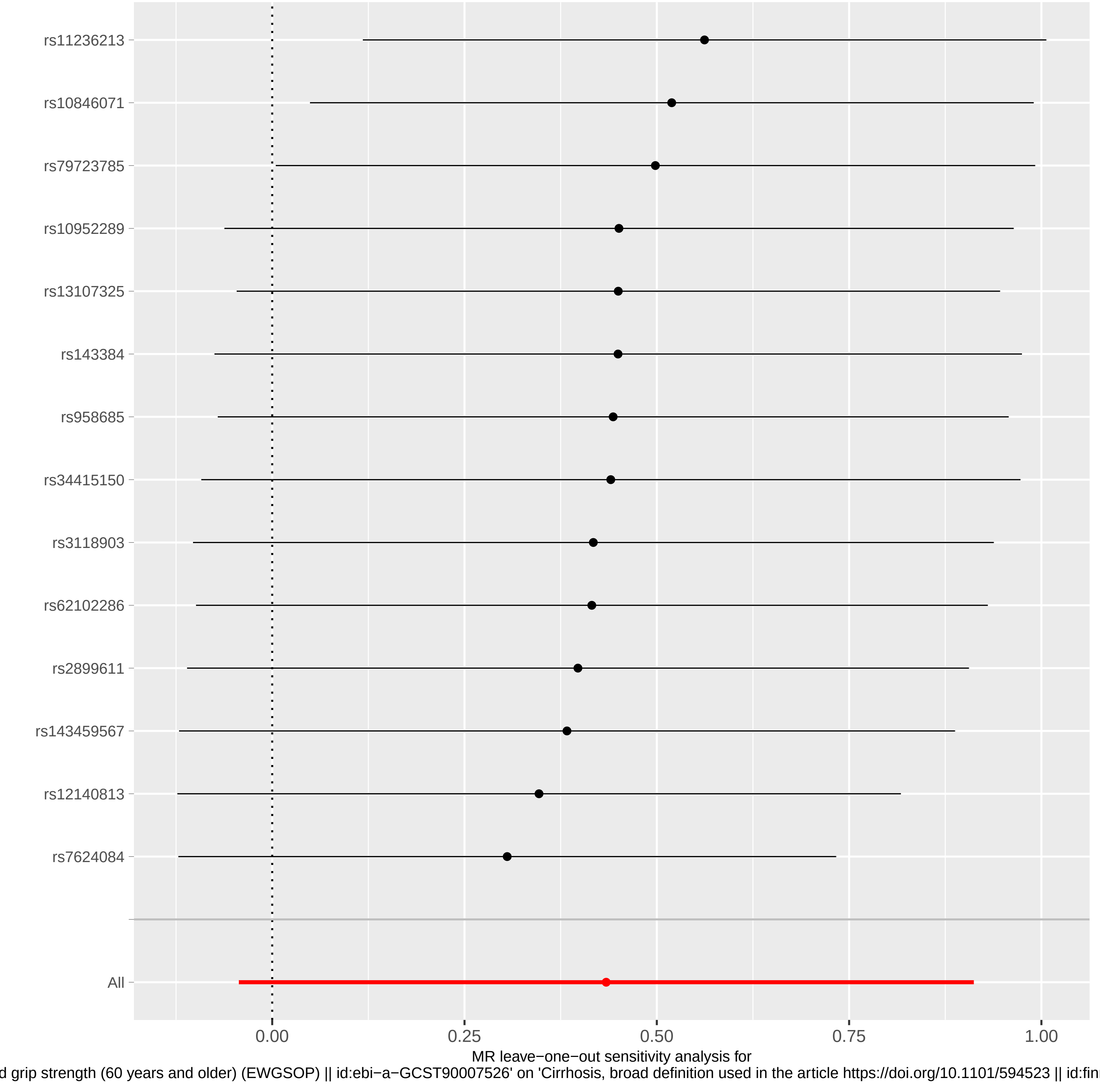
rs11236213
rs10846071
rs79723785
rs10952289
rs13107325
rs143384
rs958685
rs34415150
rs3118903
rs62102286
rs2899611
rs143459567
rs12140813
rs7624084
All
0.00
0.25
0.50
0.75
1.00
MR leave−one−out sensitivity analysis for
d grip strength (60 years and older) (EWGSOP) || id:ebi−a−GCST90007526' on 'Cirrhosis, broad definition used in the article https://doi.org/10.1101/594523 || id:fin

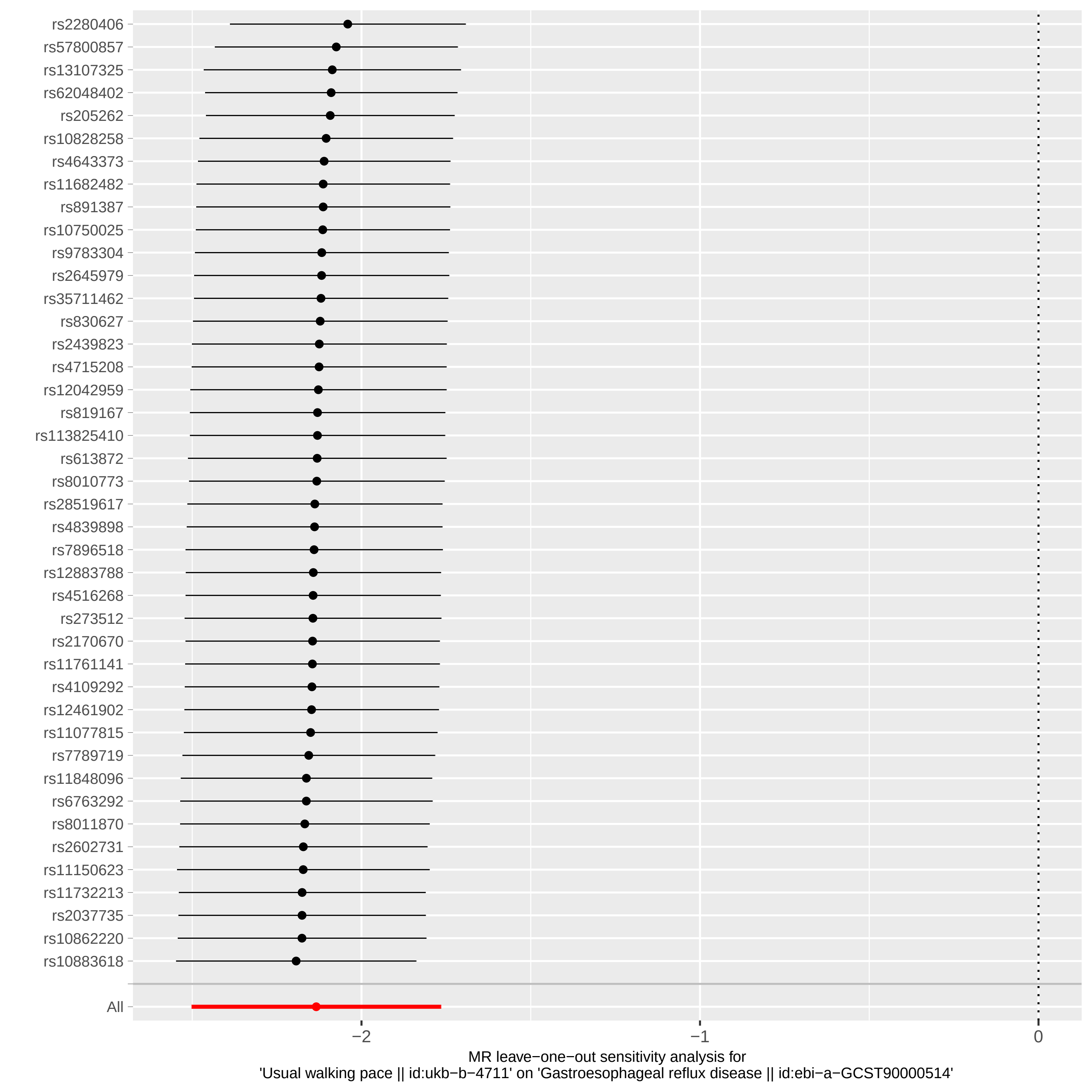
rs2280406
rs57800857
rs13107325
rs62048402
rs205262
rs10828258
rs4643373
rs11682482
rs891387
rs10750025
rs9783304
rs2645979
rs35711462
rs830627
rs2439823
rs4715208
rs12042959
rs819167
rs113825410
rs613872
rs8010773
rs28519617
rs4839898
rs7896518
rs12883788
rs4516268
rs273512
rs2170670
rs11761141
rs4109292
rs12461902
rs11077815
rs7789719
rs11848096
rs6763292
rs8011870
rs2602731
rs11150623
rs11732213
rs2037735
rs10862220
rs10883618
All
−2
−1
0
MR leave−one−out sensitivity analysis for
'Usual walking pace || id:ukb−b−4711' on 'Gastroesophageal reflux disease || id:ebi−a−GCST90000514'

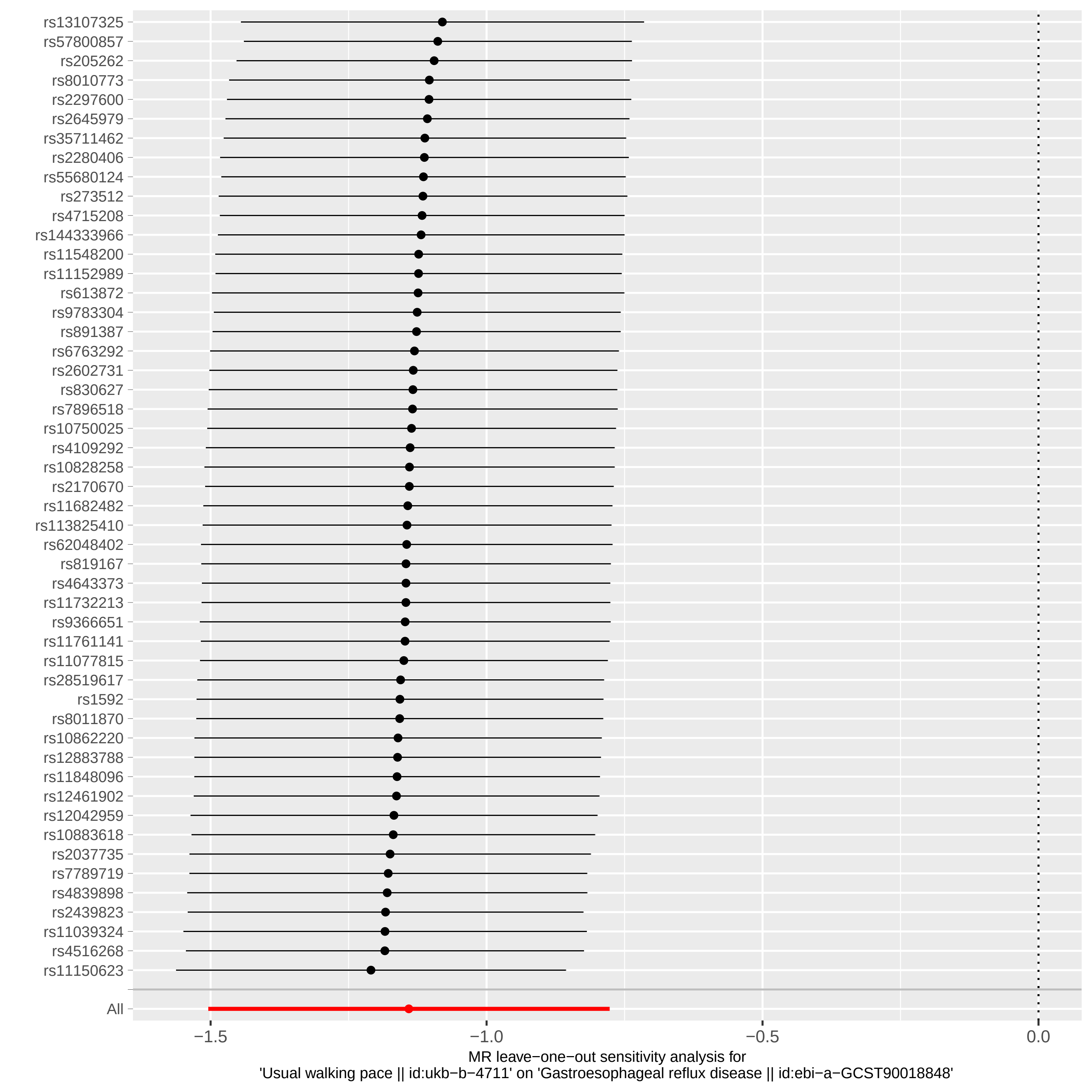

rs13107325
rs57800857
rs205262
rs8010773
rs2297600
rs2645979
rs35711462
rs2280406
rs55680124
rs273512
rs4715208
rs144333966
rs11548200
rs11152989
rs613872
rs9783304
rs891387
rs6763292
rs2602731
rs830627
rs7896518
rs10750025
rs4109292
rs10828258
rs2170670
rs11682482
rs113825410
rs62048402
rs819167
rs4643373
rs11732213
rs9366651
rs11761141
rs11077815
rs28519617
rs1592
rs8011870
rs10862220
rs12883788
rs11848096
rs12461902
rs12042959
rs10883618
rs2037735
rs7789719
rs4839898
rs2439823
rs11039324
rs4516268
rs11150623
All
−1.5
−1.0
−0.5
0.0
MR leave−one−out sensitivity analysis for
'Usual walking pace || id:ukb−b−4711' on 'Gastroesophageal reflux disease || id:ebi−a−GCST90018848'

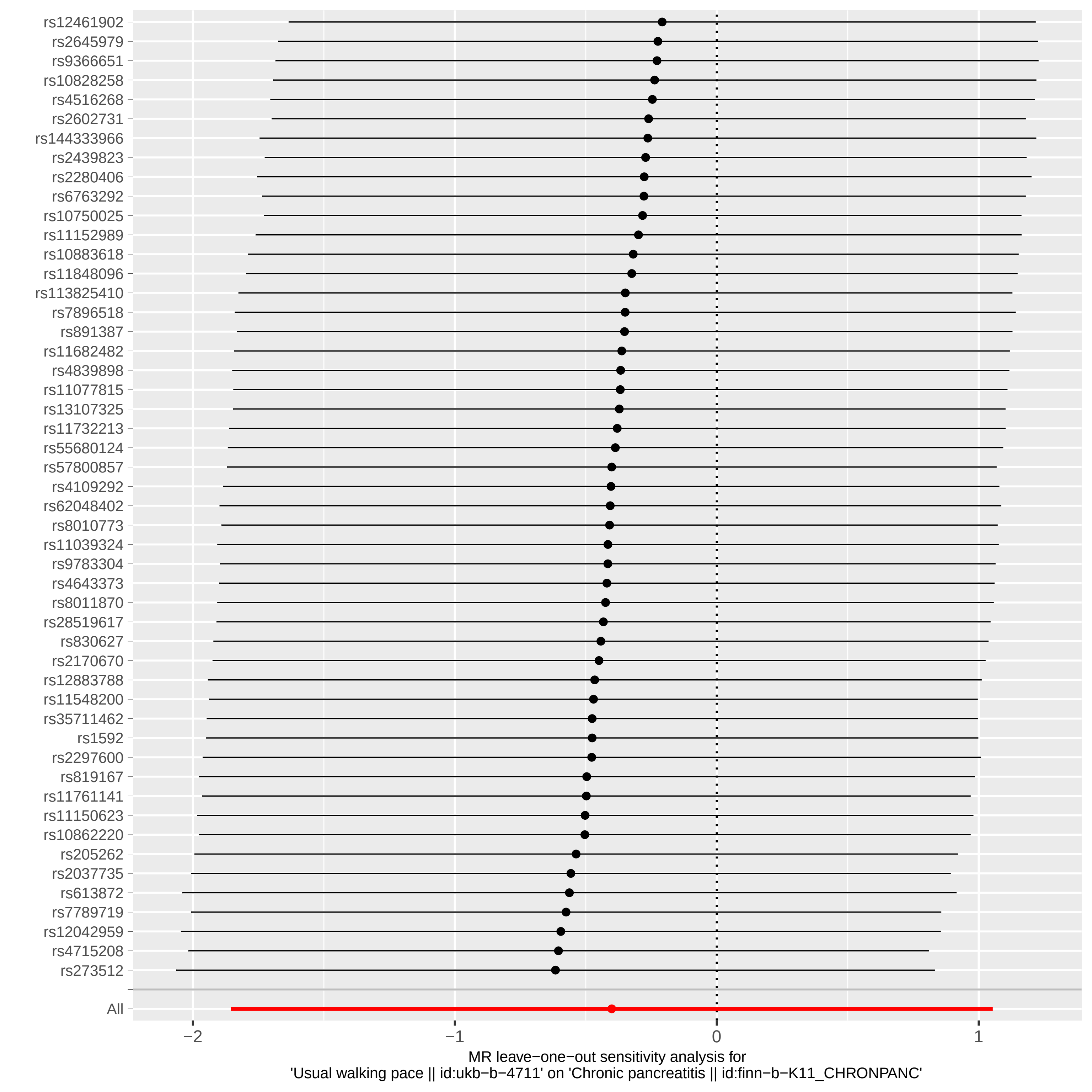
rs12461902
rs2645979
rs9366651
rs10828258
rs4516268
rs2602731
rs144333966
rs2439823
rs2280406
rs6763292
rs10750025
rs11152989
rs10883618
rs11848096
rs113825410
rs7896518
rs891387
rs11682482
rs4839898
rs11077815
rs13107325
rs11732213
rs55680124
rs57800857
rs4109292
rs62048402
rs8010773
rs11039324
rs9783304
rs4643373
rs8011870
rs28519617
rs830627
rs2170670
rs12883788
rs11548200
rs35711462
rs1592
rs2297600
rs819167
rs11761141
rs11150623
rs10862220
rs205262
rs2037735
rs613872
rs7789719
rs12042959
rs4715208
rs273512
All
−2
−1
0
1
MR leave−one−out sensitivity analysis for
'Usual walking pace || id:ukb−b−4711' on 'Chronic pancreatitis || id:finn−b−K11_CHRONPANC'

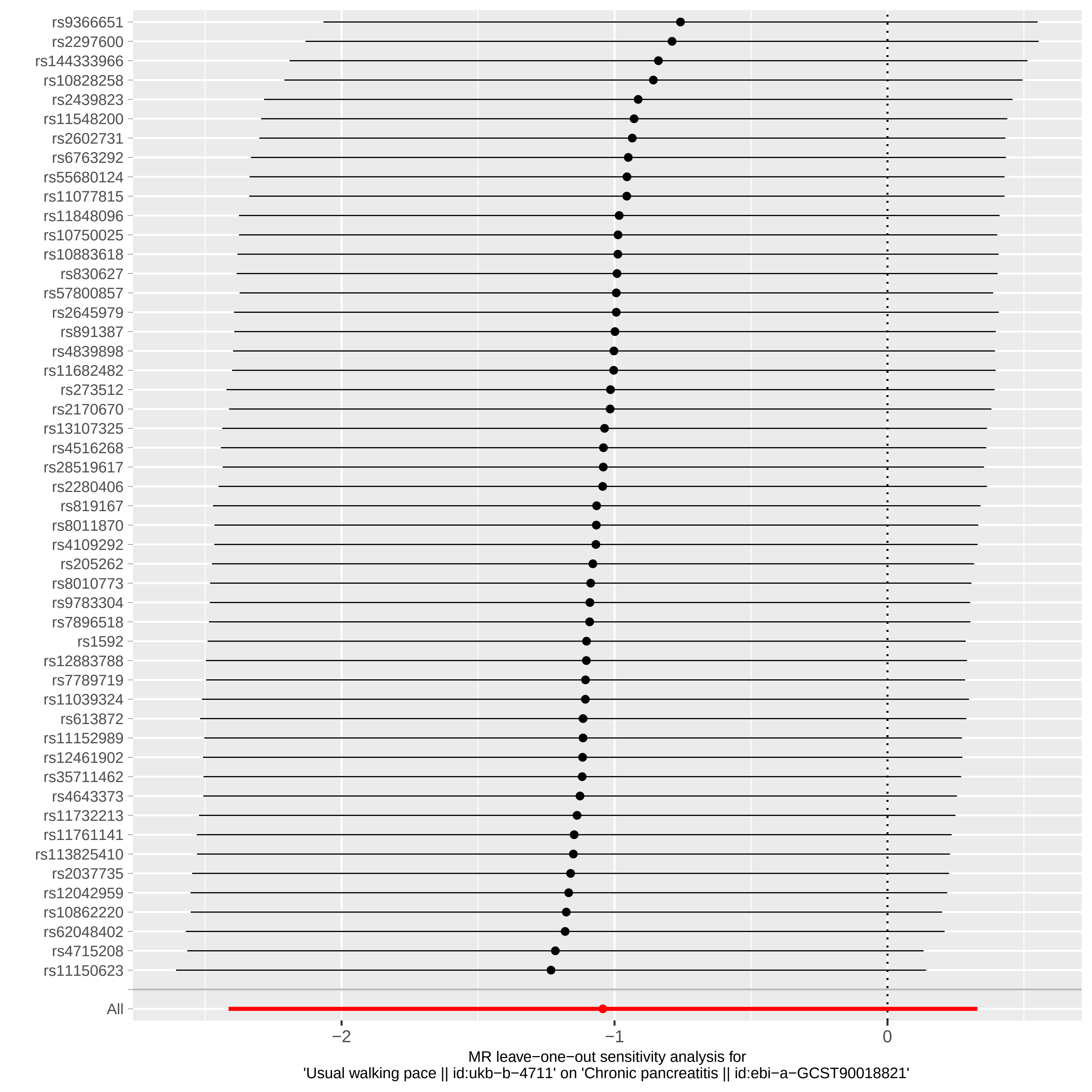
rs9366651
rs2297600
rs144333966
rs10828258
rs2439823
rs11548200
rs2602731
rs6763292
rs55680124
rs11077815
rs11848096
rs10750025
rs10883618
rs830627
rs57800857
rs2645979
rs891387
rs4839898
rs11682482
rs273512
rs2170670
rs13107325
rs4516268
rs28519617
rs2280406
rs819167
rs8011870
rs4109292
rs205262
rs8010773
rs9783304
rs7896518
rs1592
rs12883788
rs7789719
rs11039324
rs613872
rs11152989
rs12461902
rs35711462
rs4643373
rs11732213
rs11761141
rs113825410
rs2037735
rs12042959
rs10862220
rs62048402
rs4715208
rs11150623
All
−2
−1
0
MR leave−one−out sensitivity analysis for
'Usual walking pace || id:ukb−b−4711' on 'Chronic pancreatitis || id:ebi−a−GCST90018821'

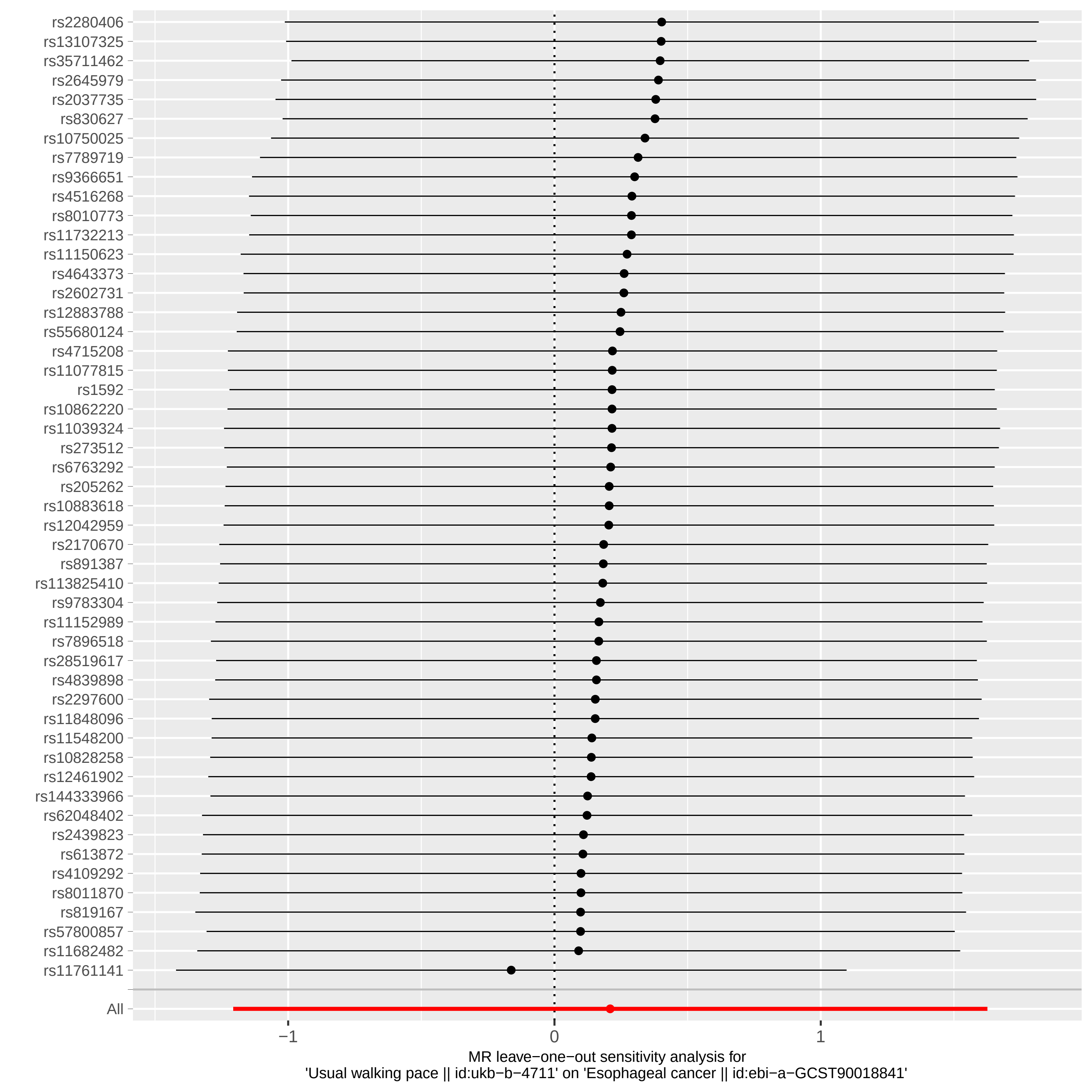
rs2280406
rs13107325
rs35711462
rs2645979
rs2037735
rs830627
rs10750025
rs7789719
rs9366651
rs4516268
rs8010773
rs11732213
rs11150623
rs4643373
rs2602731
rs12883788
rs55680124
rs4715208
rs11077815
rs1592
rs10862220
rs11039324
rs273512
rs6763292
rs205262
rs10883618
rs12042959
rs2170670
rs891387
rs113825410
rs9783304
rs11152989
rs7896518
rs28519617
rs4839898
rs2297600
rs11848096
rs11548200
rs10828258
rs12461902
rs144333966
rs62048402
rs2439823
rs613872
rs4109292
rs8011870
rs819167
rs57800857
rs11682482
rs11761141
All
−1
0
1
MR leave−one−out sensitivity analysis for
'Usual walking pace || id:ukb−b−4711' on 'Esophageal cancer || id:ebi−a−GCST90018841'

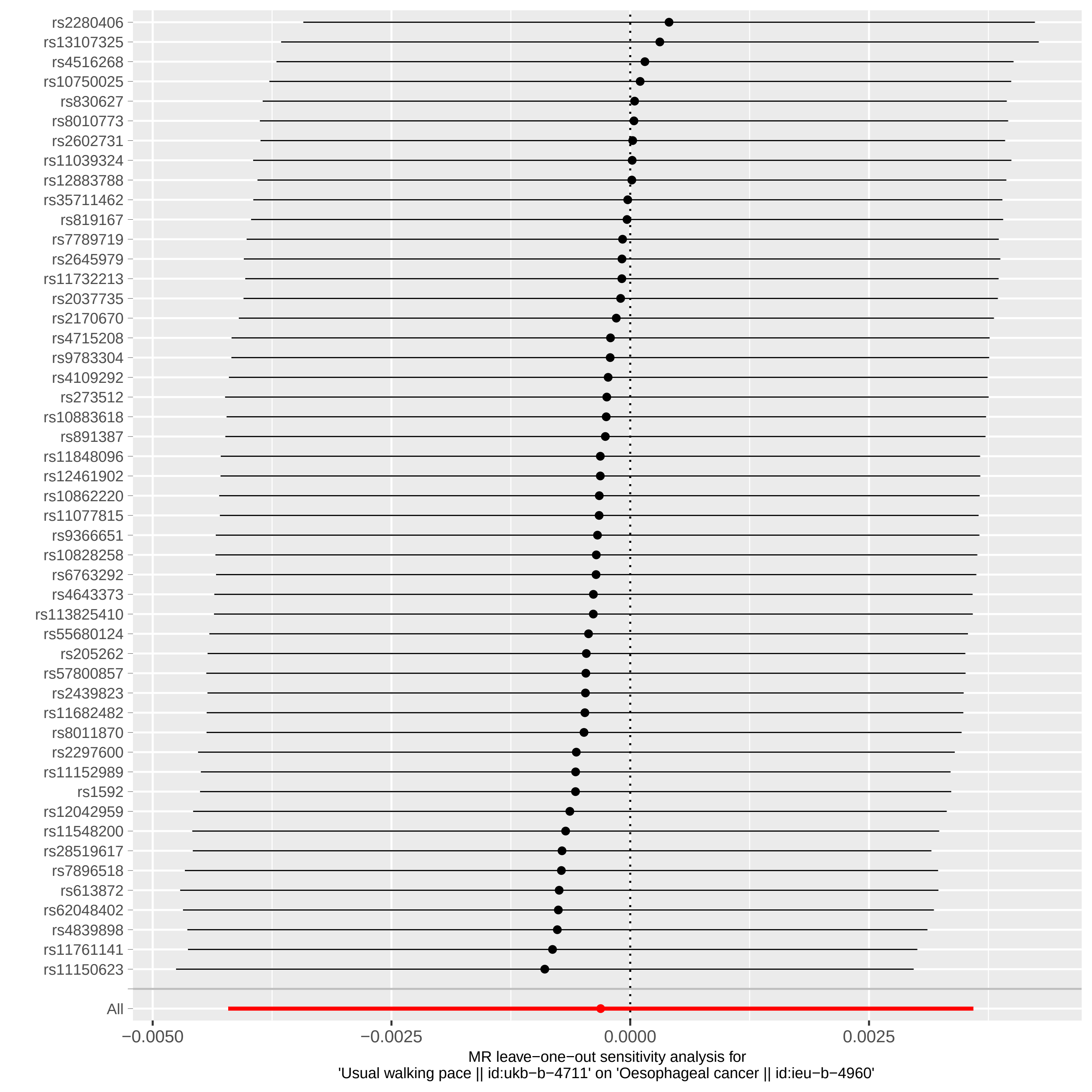
rs2280406
rs13107325
rs4516268
rs10750025
rs830627
rs8010773
rs2602731
rs11039324
rs12883788
rs35711462
rs819167
rs7789719
rs2645979
rs11732213
rs2037735
rs2170670
rs4715208
rs9783304
rs4109292
rs273512
rs10883618
rs891387
rs11848096
rs12461902
rs10862220
rs11077815
rs9366651
rs10828258
rs6763292
rs4643373
rs113825410
rs55680124
rs205262
rs57800857
rs2439823
rs11682482
rs8011870
rs2297600
rs11152989
rs1592
rs12042959
rs11548200
rs28519617
rs7896518
rs613872
rs62048402
rs4839898
rs11761141
rs11150623
All
−0.0050
−0.0025
0.0000
0.0025
MR leave−one−out sensitivity analysis for
'Usual walking pace || id:ukb−b−4711' on 'Oesophageal cancer || id:ieu−b−4960'

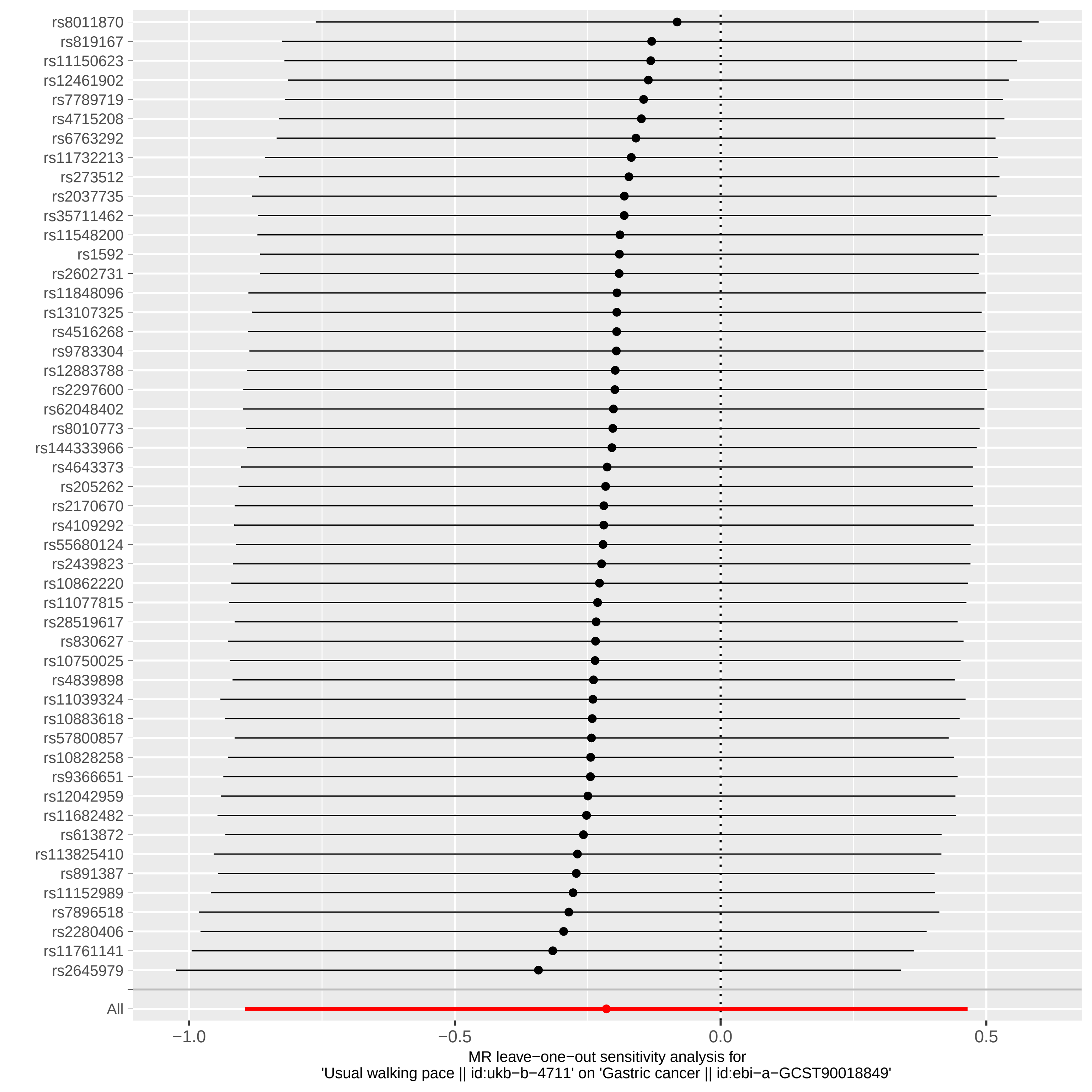

rs8011870
rs819167
rs11150623
rs12461902
rs7789719
rs4715208
rs6763292
rs11732213
rs273512
rs2037735
rs35711462
rs11548200
rs1592
rs2602731
rs11848096
rs13107325
rs4516268
rs9783304
rs12883788
rs2297600
rs62048402
rs8010773
rs144333966
rs4643373
rs205262
rs2170670
rs4109292
rs55680124
rs2439823
rs10862220
rs11077815
rs28519617
rs830627
rs10750025
rs4839898
rs11039324
rs10883618
rs57800857
rs10828258
rs9366651
rs12042959
rs11682482
rs613872
rs113825410
rs891387
rs11152989
rs7896518
rs2280406
rs11761141
rs2645979
All
−1.0
−0.5
0.0
0.5
MR leave−one−out sensitivity analysis for
'Usual walking pace || id:ukb−b−4711' on 'Gastric cancer || id:ebi−a−GCST90018849'

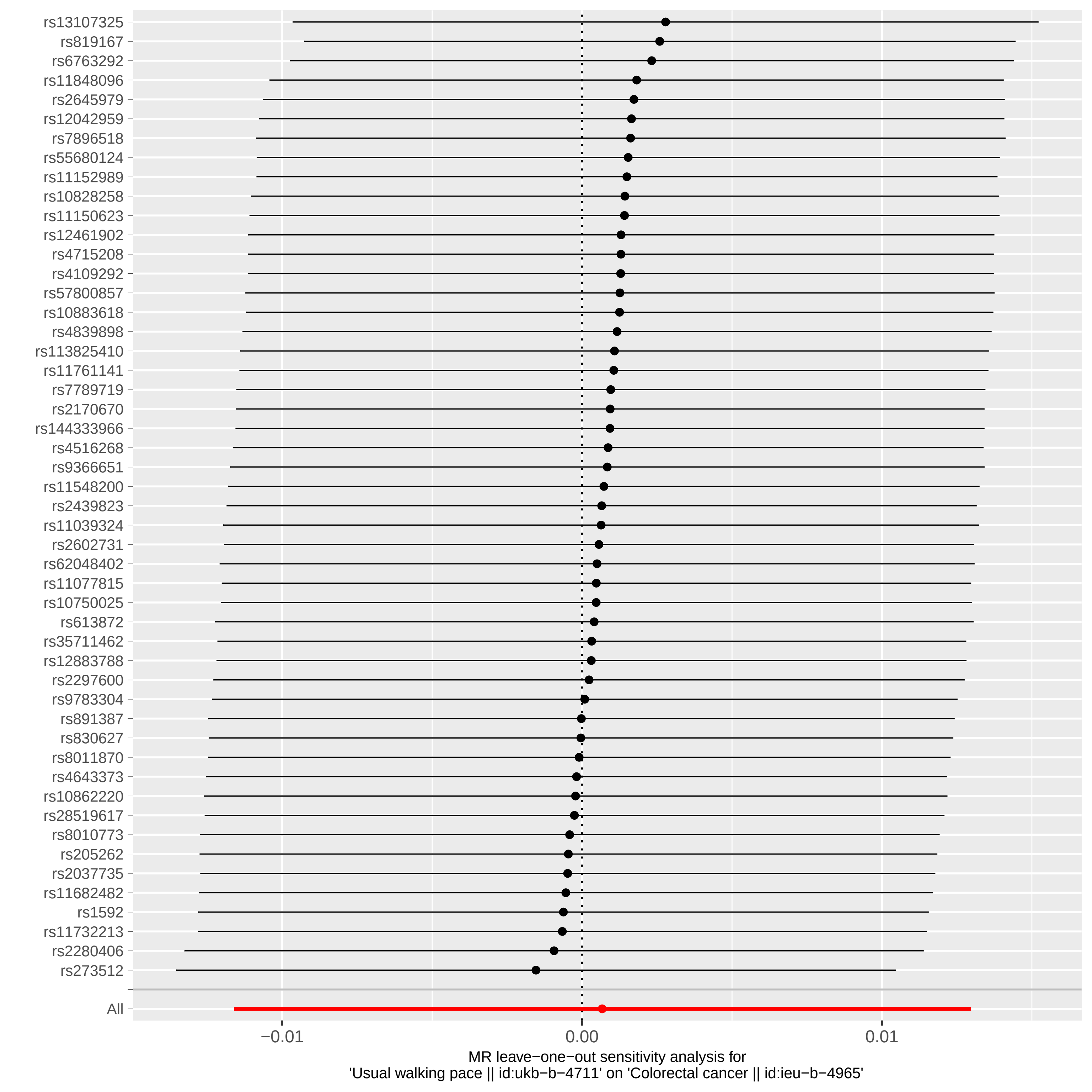
rs13107325
rs819167
rs6763292
rs11848096
rs2645979
rs12042959
rs7896518
rs55680124
rs11152989
rs10828258
rs11150623
rs12461902
rs4715208
rs4109292
rs57800857
rs10883618
rs4839898
rs113825410
rs11761141
rs7789719
rs2170670
rs144333966
rs4516268
rs9366651
rs11548200
rs2439823
rs11039324
rs2602731
rs62048402
rs11077815
rs10750025
rs613872
rs35711462
rs12883788
rs2297600
rs9783304
rs891387
rs830627
rs8011870
rs4643373
rs10862220
rs28519617
rs8010773
rs205262
rs2037735
rs11682482
rs1592
rs11732213
rs2280406
rs273512
All
−0.01
0.00
0.01
MR leave−one−out sensitivity analysis for
'Usual walking pace || id:ukb−b−4711' on 'Colorectal cancer || id:ieu−b−4965'

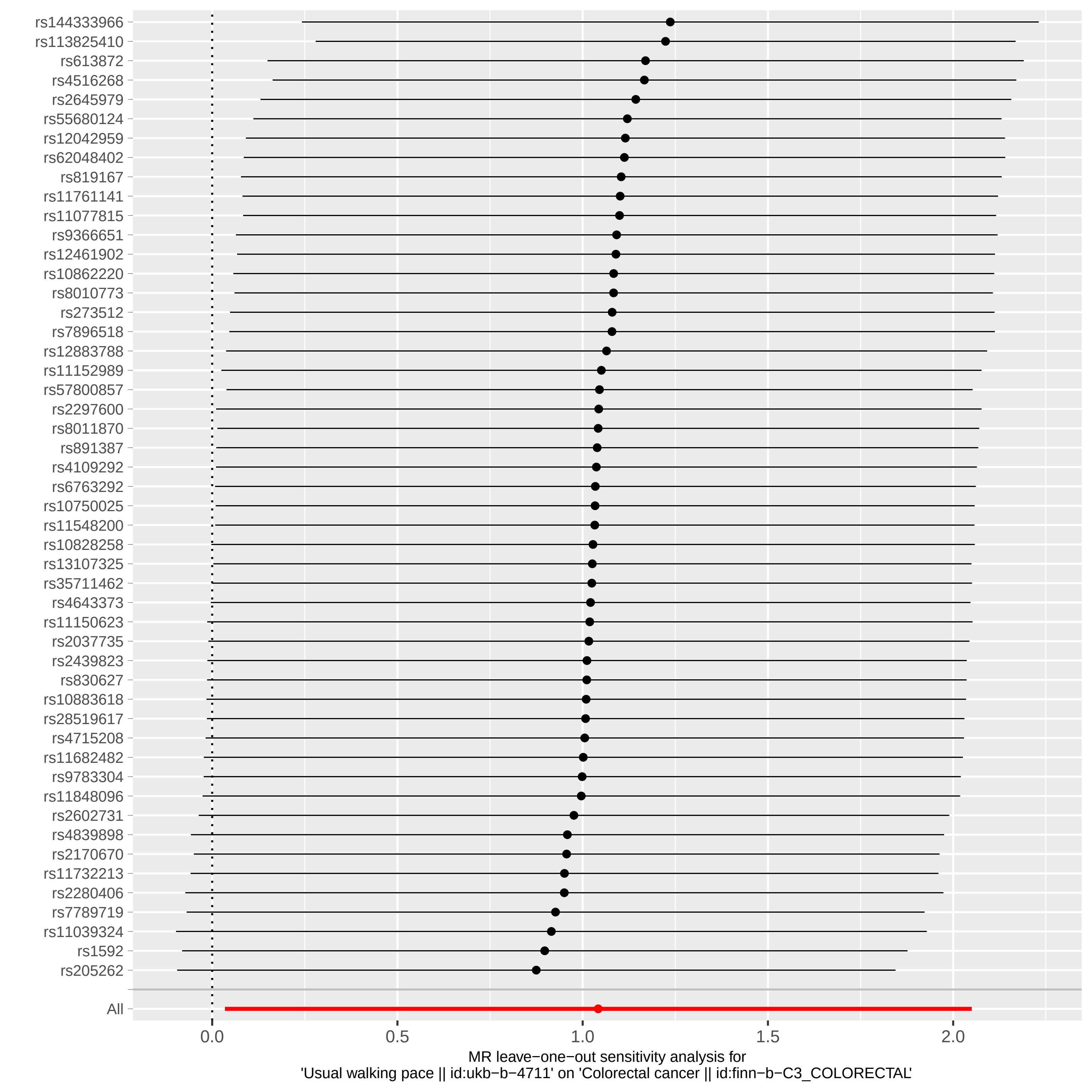
rs144333966
rs113825410
rs613872
rs4516268
rs2645979
rs55680124
rs12042959
rs62048402
rs819167
rs11761141
rs11077815
rs9366651
rs12461902
rs10862220
rs8010773
rs273512
rs7896518
rs12883788
rs11152989
rs57800857
rs2297600
rs8011870
rs891387
rs4109292
rs6763292
rs10750025
rs11548200
rs10828258
rs13107325
rs35711462
rs4643373
rs11150623
rs2037735
rs2439823
rs830627
rs10883618
rs28519617
rs4715208
rs11682482
rs9783304
rs11848096
rs2602731
rs4839898
rs2170670
rs11732213
rs2280406
rs7789719
rs11039324
rs1592
rs205262
All
0.0
0.5
1.0
1.5
2.0
MR leave−one−out sensitivity analysis for
'Usual walking pace || id:ukb−b−4711' on 'Colorectal cancer || id:finn−b−C3_COLORECTAL'

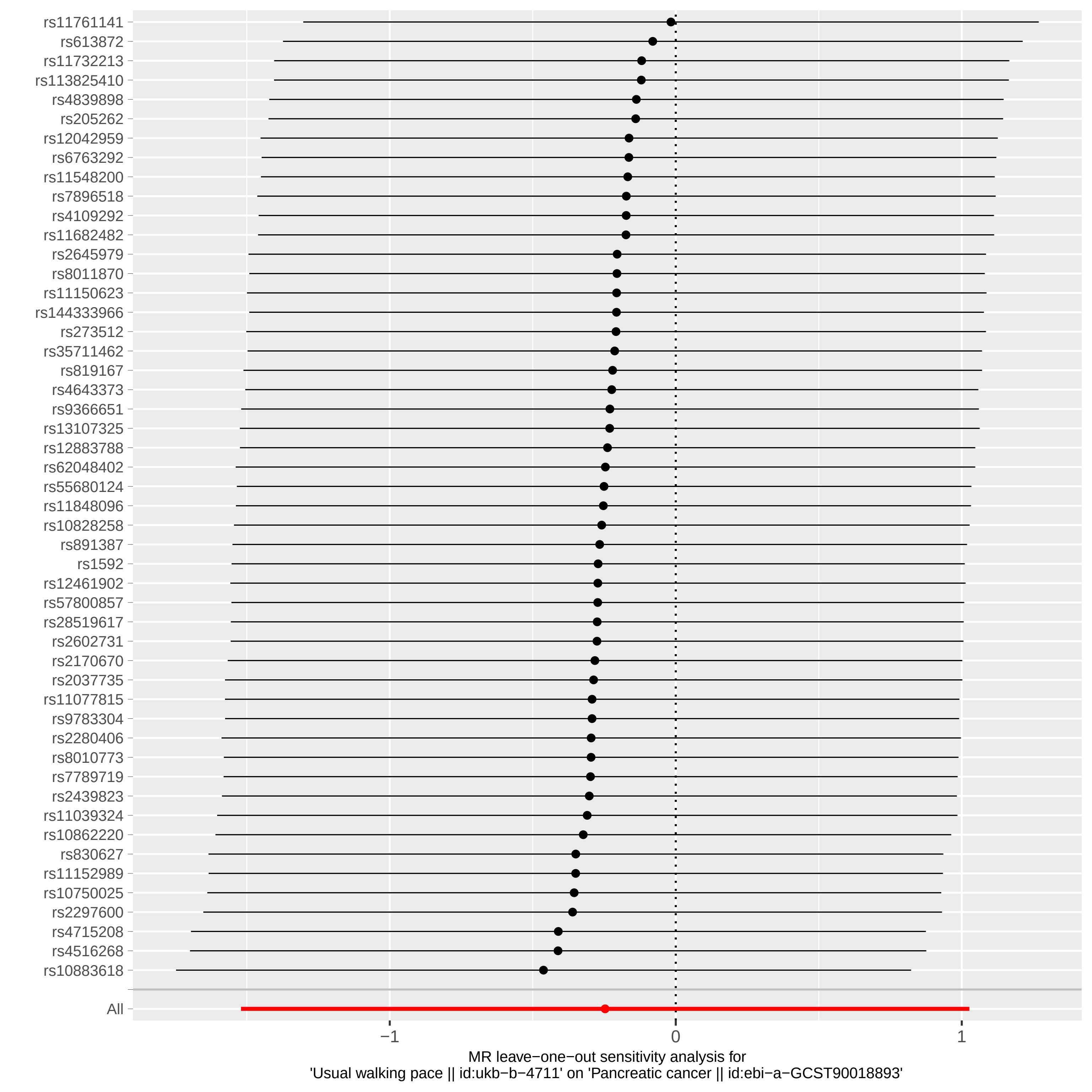
rs11761141
rs613872
rs11732213
rs113825410
rs4839898
rs205262
rs12042959
rs6763292
rs11548200
rs7896518
rs4109292
rs11682482
rs2645979
rs8011870
rs11150623
rs144333966
rs273512
rs35711462
rs819167
rs4643373
rs9366651
rs13107325
rs12883788
rs62048402
rs55680124
rs11848096
rs10828258
rs891387
rs1592
rs12461902
rs57800857
rs28519617
rs2602731
rs2170670
rs2037735
rs11077815
rs9783304
rs2280406
rs8010773
rs7789719
rs2439823
rs11039324
rs10862220
rs830627
rs11152989
rs10750025
rs2297600
rs4715208
rs4516268
rs10883618
All
−1
0
1
MR leave−one−out sensitivity analysis for
'Usual walking pace || id:ukb−b−4711' on 'Pancreatic cancer || id:ebi−a−GCST90018893'

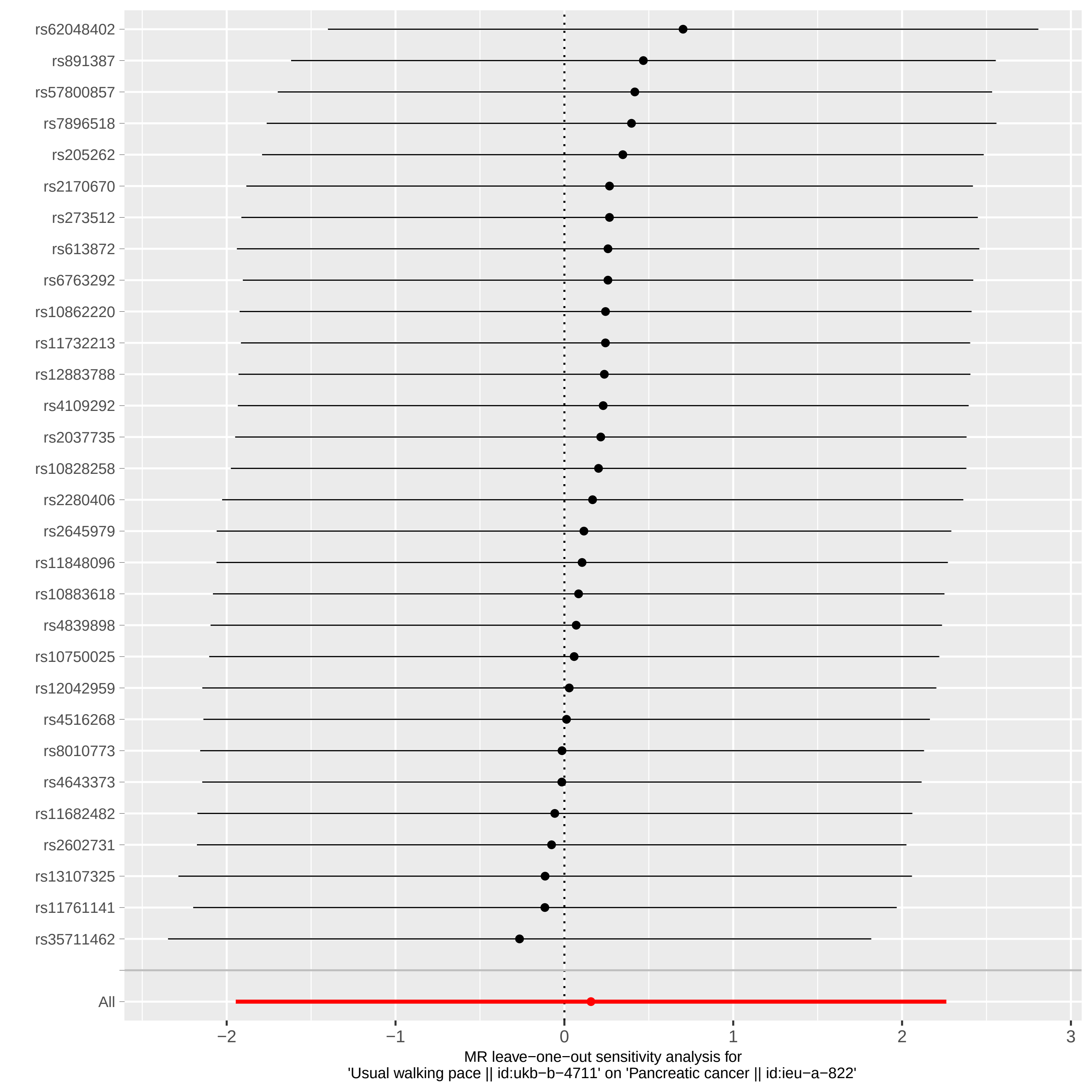

rs62048402
rs891387
rs57800857
rs7896518
rs205262
rs2170670
rs273512
rs613872
rs6763292
rs10862220
rs11732213
rs12883788
rs4109292
rs2037735
rs10828258
rs2280406
rs2645979
rs11848096
rs10883618
rs4839898
rs10750025
rs12042959
rs4516268
rs8010773
rs4643373
rs11682482
rs2602731
rs13107325
rs11761141
rs35711462
All
−2
−1
0
1
2
3
MR leave−one−out sensitivity analysis for
'Usual walking pace || id:ukb−b−4711' on 'Pancreatic cancer || id:ieu−a−822'

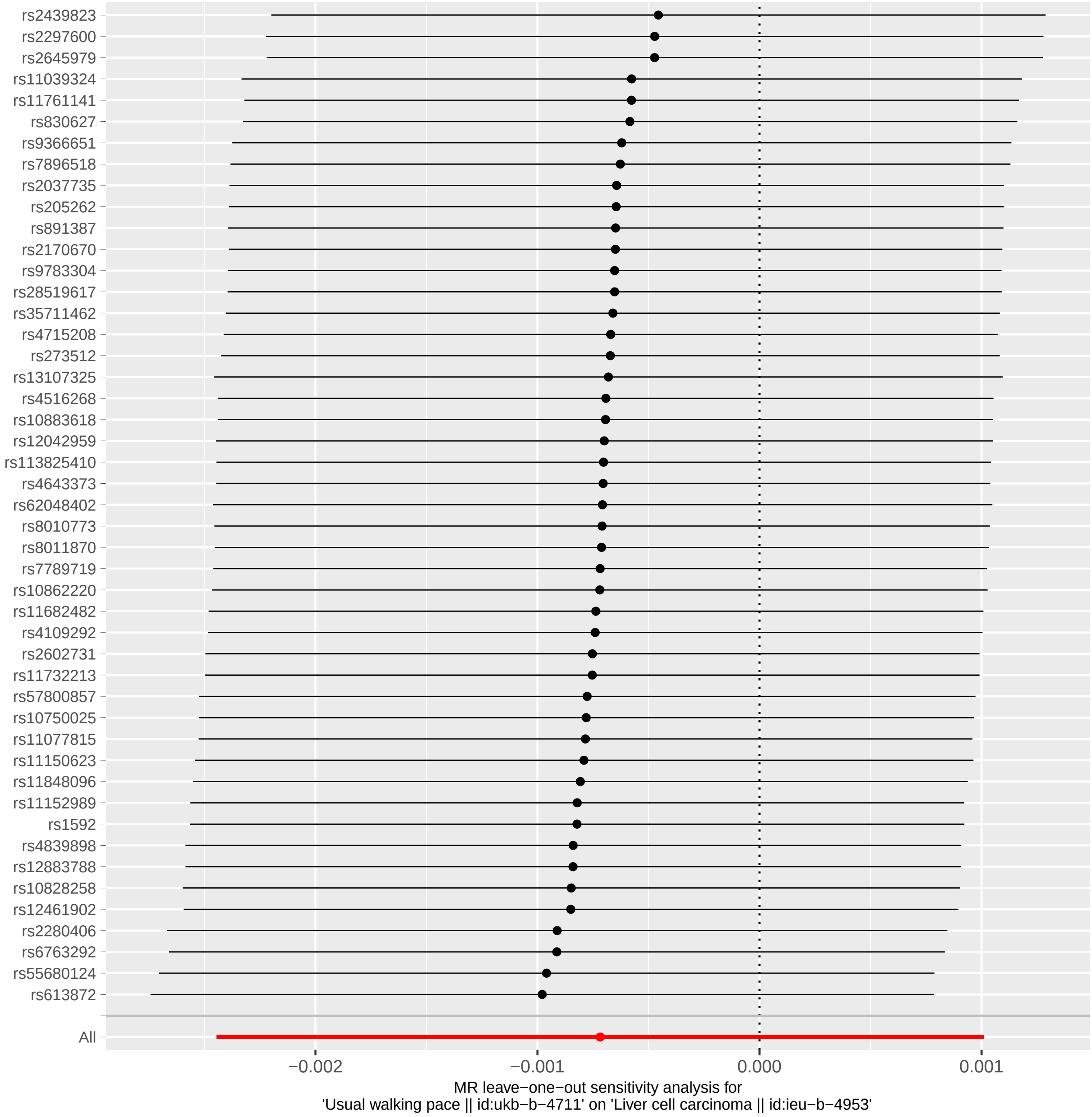

rs2439823
rs2297600
rs2645979
rs11039324
rs11761141
rs830627
rs9366651
rs7896518
rs2037735
rs205262
rs891387
rs2170670
rs9783304
rs28519617
rs35711462
rs4715208
rs273512
rs13107325
rs4516268
rs10883618
rs12042959
rs113825410
rs4643373
rs62048402
rs8010773
rs8011870
rs7789719
rs10862220
rs11682482
rs4109292
rs2602731
rs11732213
rs57800857
rs10750025
rs11077815
rs11150623
rs11848096
rs11152989
rs1592
rs4839898
rs12883788
rs10828258
rs12461902
rs2280406
rs6763292
rs55680124
rs613872
All
−0.002
−0.001
0.000
0.001
MR leave−one−out sensitivity analysis for
'Usual walking pace || id:ukb−b−4711' on 'Liver cell carcinoma || id:ieu−b−4953'

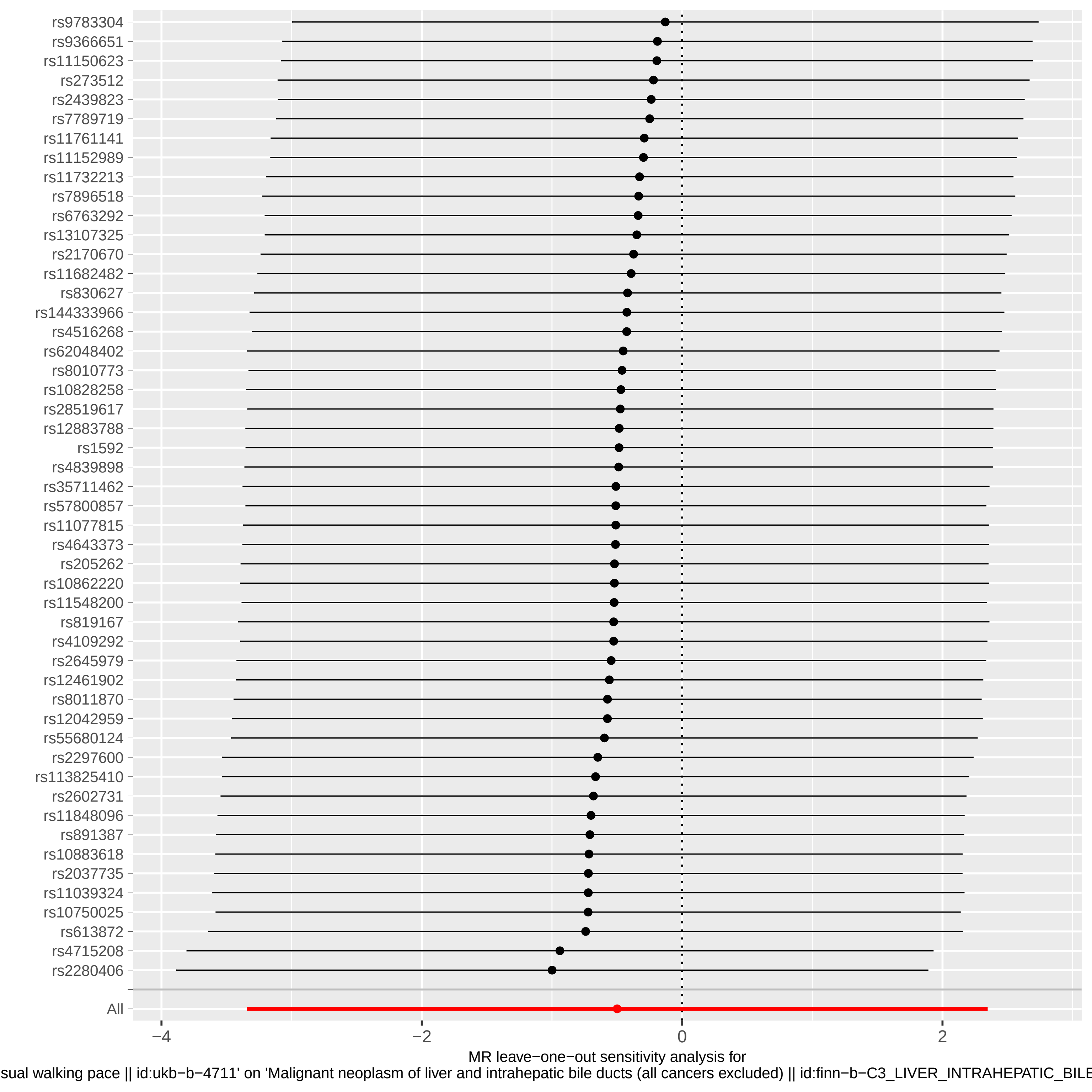
rs9783304
rs9366651
rs11150623
rs273512
rs2439823
rs7789719
rs11761141
rs11152989
rs11732213
rs7896518
rs6763292
rs13107325
rs2170670
rs11682482
rs830627
rs144333966
rs4516268
rs62048402
rs8010773
rs10828258
rs28519617
rs12883788
rs1592
rs4839898
rs35711462
rs57800857
rs11077815
rs4643373
rs205262
rs10862220
rs11548200
rs819167
rs4109292
rs2645979
rs12461902
rs8011870
rs12042959
rs55680124
rs2297600
rs113825410
rs2602731
rs11848096
rs891387
rs10883618
rs2037735
rs11039324
rs10750025
rs613872
rs4715208
rs2280406
All
−4
−2
0
2
MR leave−one−out sensitivity analysis for
sual walking pace || id:ukb−b−4711' on 'Malignant neoplasm of liver and intrahepatic bile ducts (all cancers excluded) || id:finn−b−C3_LIVER_INTRAHEPATIC_BILE

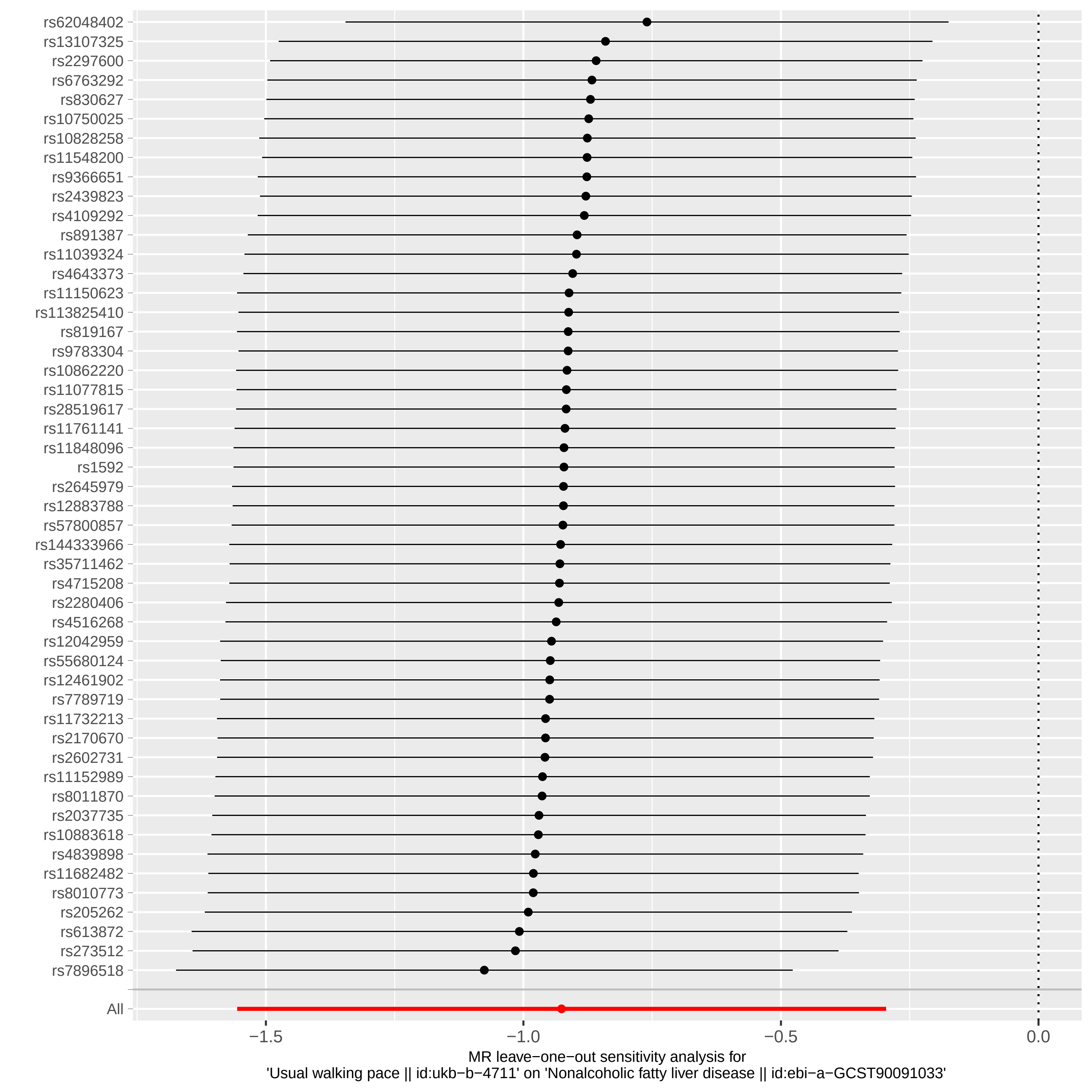
rs62048402
rs13107325
rs2297600
rs6763292
rs830627
rs10750025
rs10828258
rs11548200
rs9366651
rs2439823
rs4109292
rs891387
rs11039324
rs4643373
rs11150623
rs113825410
rs819167
rs9783304
rs10862220
rs11077815
rs28519617
rs11761141
rs11848096
rs1592
rs2645979
rs12883788
rs57800857
rs144333966
rs35711462
rs4715208
rs2280406
rs4516268
rs12042959
rs55680124
rs12461902
rs7789719
rs11732213
rs2170670
rs2602731
rs11152989
rs8011870
rs2037735
rs10883618
rs4839898
rs11682482
rs8010773
rs205262
rs613872
rs273512
rs7896518
All
−1.5
−1.0
−0.5
0.0
MR leave−one−out sensitivity analysis for
'Usual walking pace || id:ukb−b−4711' on 'Nonalcoholic fatty liver disease || id:ebi−a−GCST90091033'

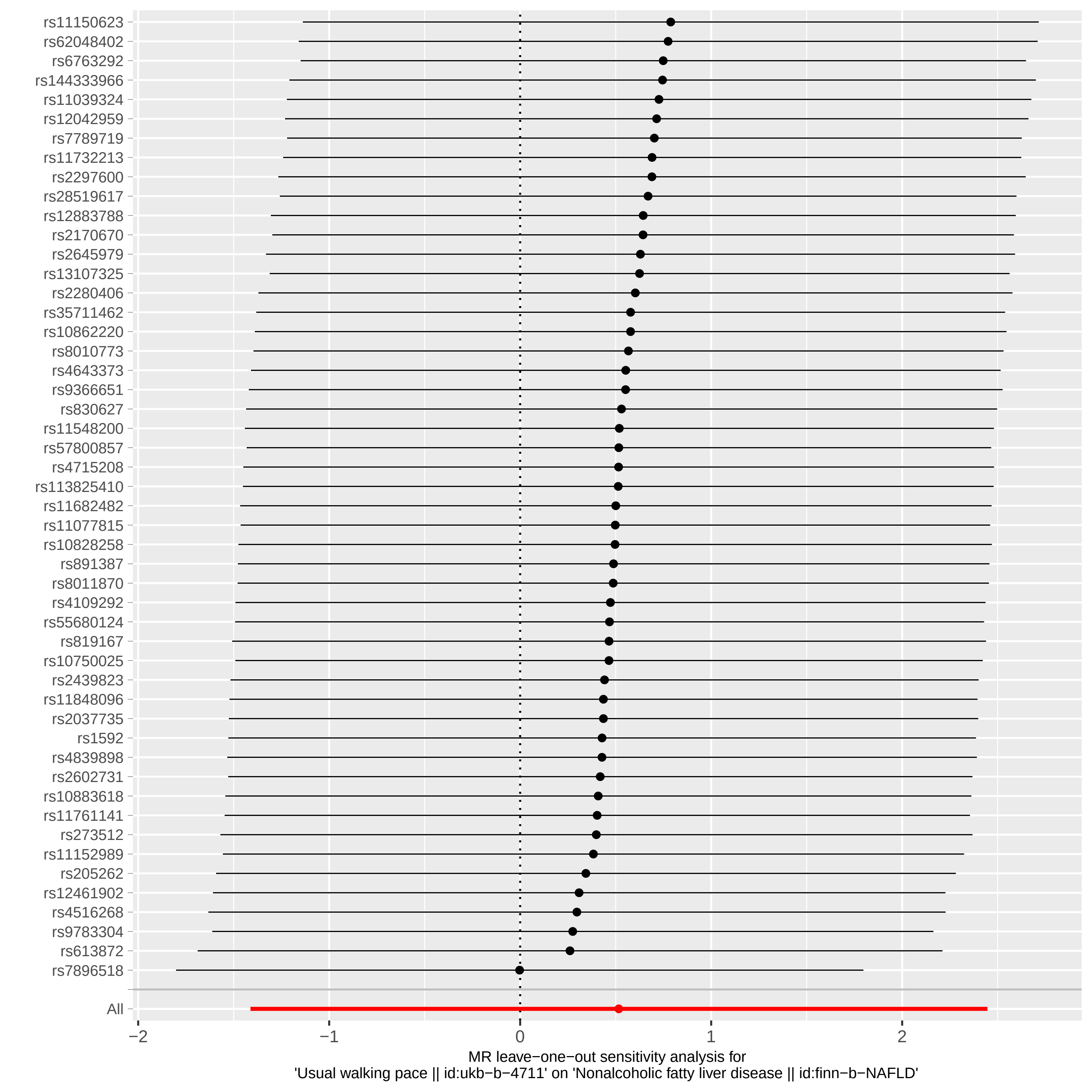
rs11150623
rs62048402
rs6763292
rs144333966
rs11039324
rs12042959
rs7789719
rs11732213
rs2297600
rs28519617
rs12883788
rs2170670
rs2645979
rs13107325
rs2280406
rs35711462
rs10862220
rs8010773
rs4643373
rs9366651
rs830627
rs11548200
rs57800857
rs4715208
rs113825410
rs11682482
rs11077815
rs10828258
rs891387
rs8011870
rs4109292
rs55680124
rs819167
rs10750025
rs2439823
rs11848096
rs2037735
rs1592
rs4839898
rs2602731
rs10883618
rs11761141
rs273512
rs11152989
rs205262
rs12461902
rs4516268
rs9783304
rs613872
rs7896518
All
−2
−1
0
1
2
MR leave−one−out sensitivity analysis for
'Usual walking pace || id:ukb−b−4711' on 'Nonalcoholic fatty liver disease || id:finn−b−NAFLD'

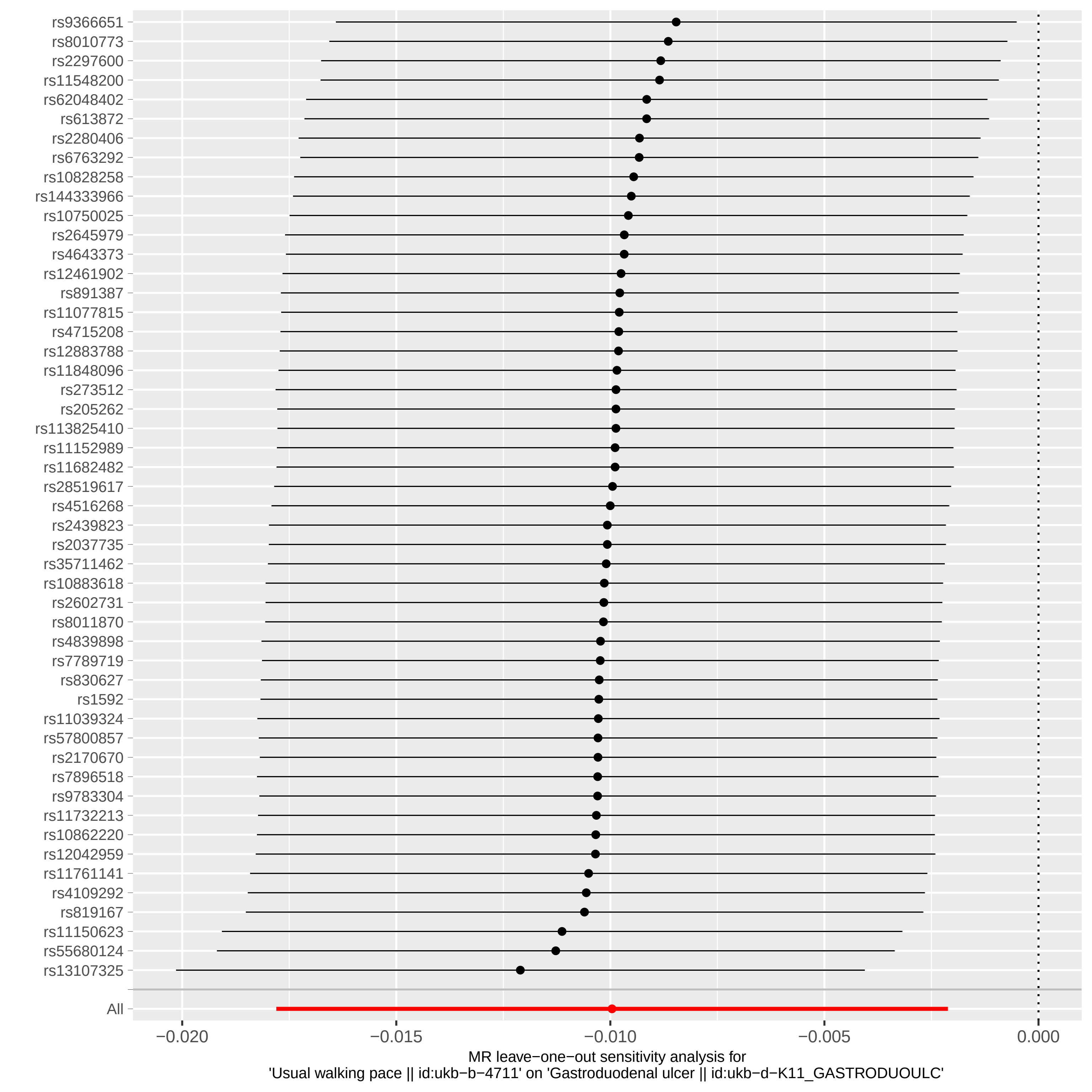
rs9366651
rs8010773
rs2297600
rs11548200
rs62048402
rs613872
rs2280406
rs6763292
rs10828258
rs144333966
rs10750025
rs2645979
rs4643373
rs12461902
rs891387
rs11077815
rs4715208
rs12883788
rs11848096
rs273512
rs205262
rs113825410
rs11152989
rs11682482
rs28519617
rs4516268
rs2439823
rs2037735
rs35711462
rs10883618
rs2602731
rs8011870
rs4839898
rs7789719
rs830627
rs1592
rs11039324
rs57800857
rs2170670
rs7896518
rs9783304
rs11732213
rs10862220
rs12042959
rs11761141
rs4109292
rs819167
rs11150623
rs55680124
rs13107325
All
−0.020
−0.015
−0.010
−0.005
0.000
MR leave−one−out sensitivity analysis for
'Usual walking pace || id:ukb−b−4711' on 'Gastroduodenal ulcer || id:ukb−d−K11_GASTRODUOULC'

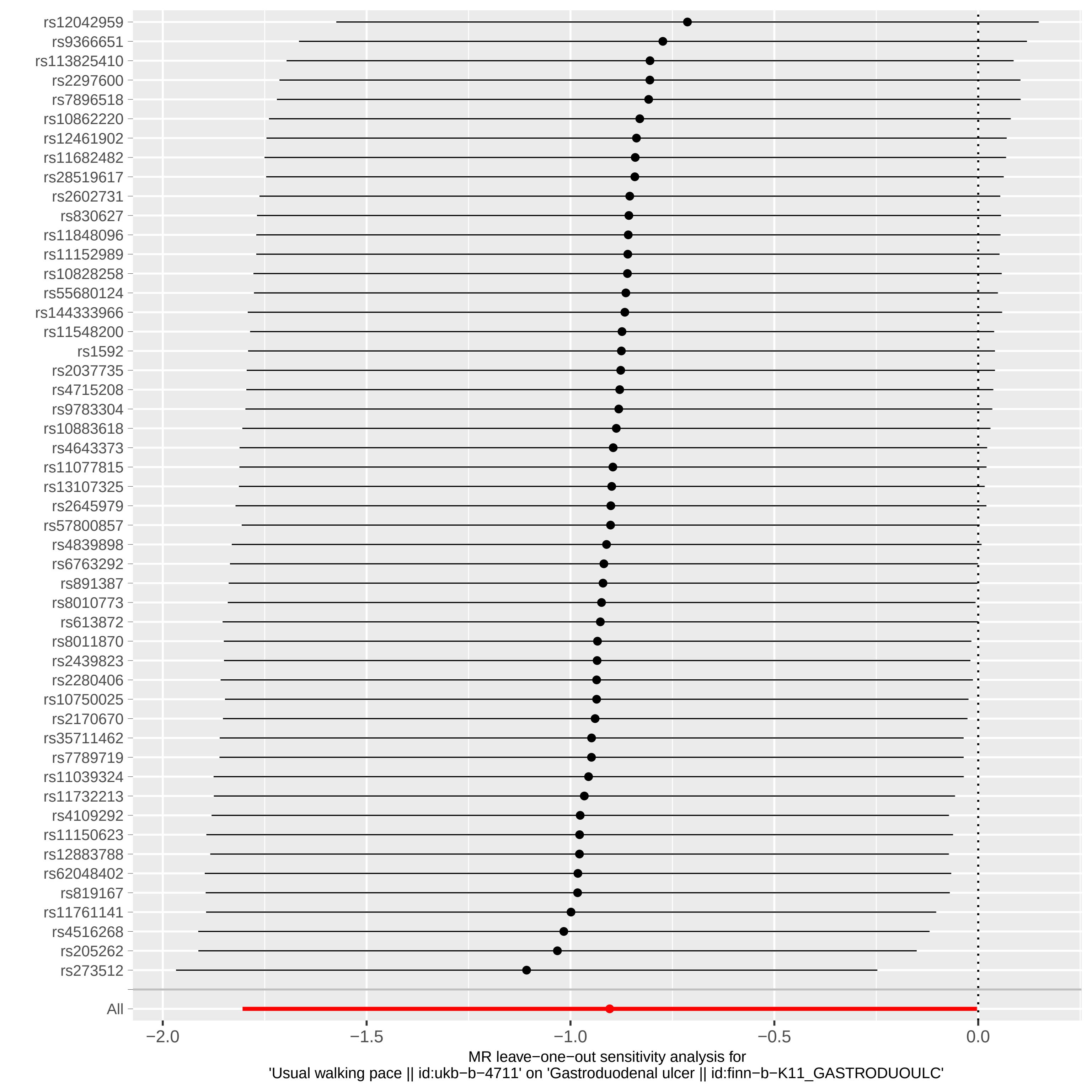
rs12042959
rs9366651
rs113825410
rs2297600
rs7896518
rs10862220
rs12461902
rs11682482
rs28519617
rs2602731
rs830627
rs11848096
rs11152989
rs10828258
rs55680124
rs144333966
rs11548200
rs1592
rs2037735
rs4715208
rs9783304
rs10883618
rs4643373
rs11077815
rs13107325
rs2645979
rs57800857
rs4839898
rs6763292
rs891387
rs8010773
rs613872
rs8011870
rs2439823
rs2280406
rs10750025
rs2170670
rs35711462
rs7789719
rs11039324
rs11732213
rs4109292
rs11150623
rs12883788
rs62048402
rs819167
rs11761141
rs4516268
rs205262
rs273512
All
−2.0
−1.5
−1.0
−0.5
0.0
MR leave−one−out sensitivity analysis for
'Usual walking pace || id:ukb−b−4711' on 'Gastroduodenal ulcer || id:finn−b−K11_GASTRODUOULC'

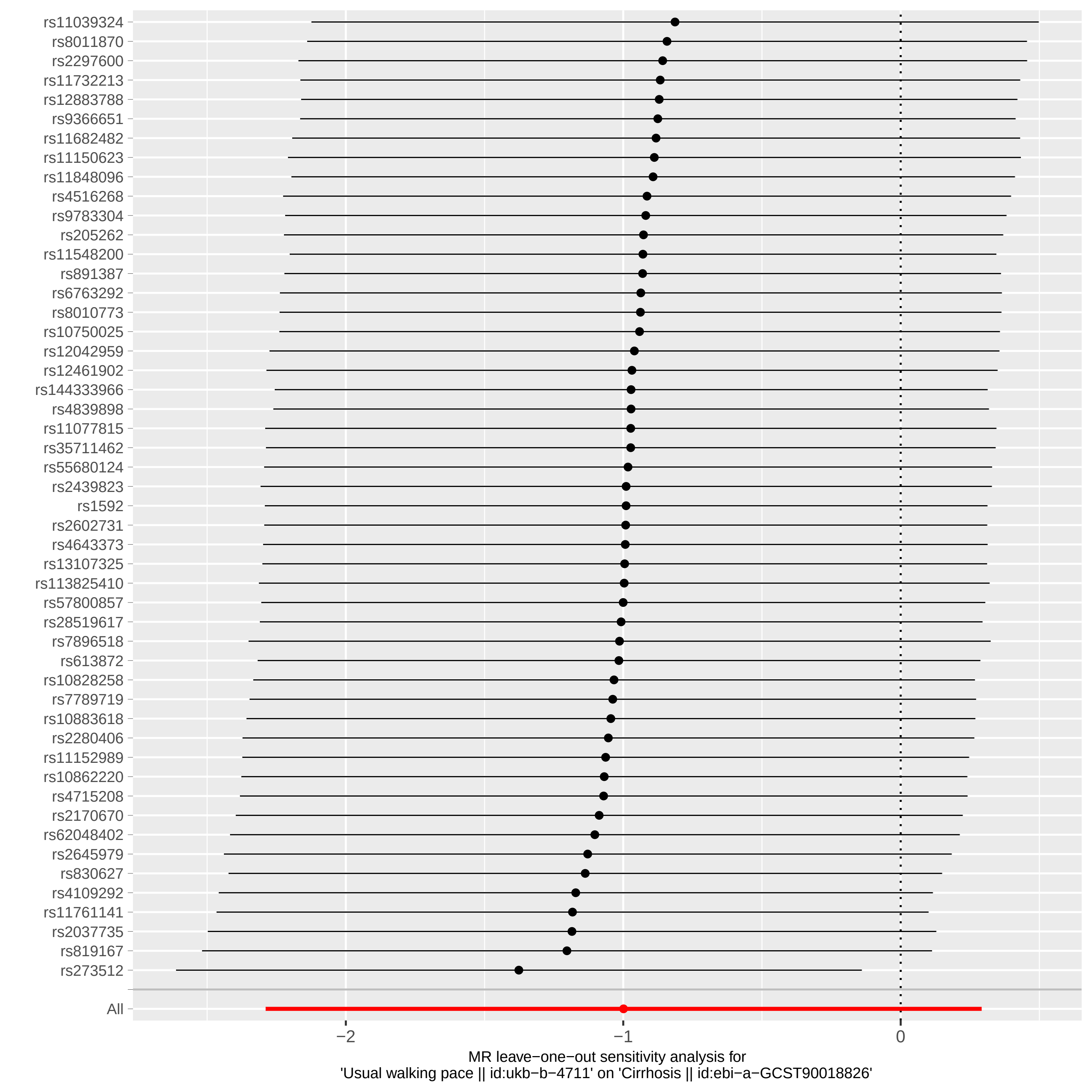

rs11039324
rs8011870
rs2297600
rs11732213
rs12883788
rs9366651
rs11682482
rs11150623
rs11848096
rs4516268
rs9783304
rs205262
rs11548200
rs891387
rs6763292
rs8010773
rs10750025
rs12042959
rs12461902
rs144333966
rs4839898
rs11077815
rs35711462
rs55680124
rs2439823
rs1592
rs2602731
rs4643373
rs13107325
rs113825410
rs57800857
rs28519617
rs7896518
rs613872
rs10828258
rs7789719
rs10883618
rs2280406
rs11152989
rs10862220
rs4715208
rs2170670
rs62048402
rs2645979
rs830627
rs4109292
rs11761141
rs2037735
rs819167
rs273512
All
−2
−1
0
MR leave−one−out sensitivity analysis for
'Usual walking pace || id:ukb−b−4711' on 'Cirrhosis || id:ebi−a−GCST90018826'

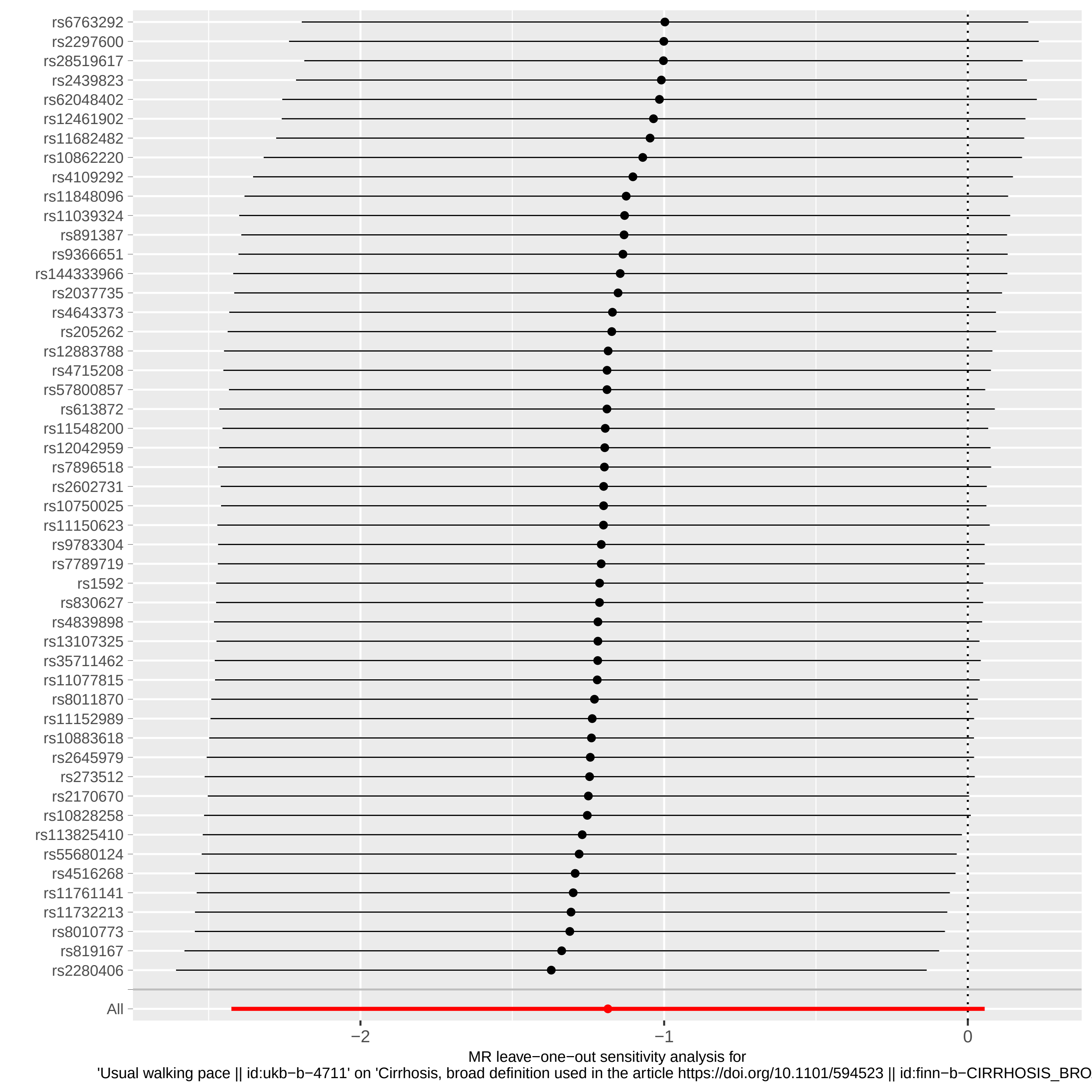
rs6763292
rs2297600
rs28519617
rs2439823
rs62048402
rs12461902
rs11682482
rs10862220
rs4109292
rs11848096
rs11039324
rs891387
rs9366651
rs144333966
rs2037735
rs4643373
rs205262
rs12883788
rs4715208
rs57800857
rs613872
rs11548200
rs12042959
rs7896518
rs2602731
rs10750025
rs11150623
rs9783304
rs7789719
rs1592
rs830627
rs4839898
rs13107325
rs35711462
rs11077815
rs8011870
rs11152989
rs10883618
rs2645979
rs273512
rs2170670
rs10828258
rs113825410
rs55680124
rs4516268
rs11761141
rs11732213
rs8010773
rs819167
rs2280406
All
−2
−1
0
MR leave−one−out sensitivity analysis for
'Usual walking pace || id:ukb−b−4711' on 'Cirrhosis, broad definition used in the article https://doi.org/10.1101/594523 || id:finn−b−CIRRHOSIS_BRO

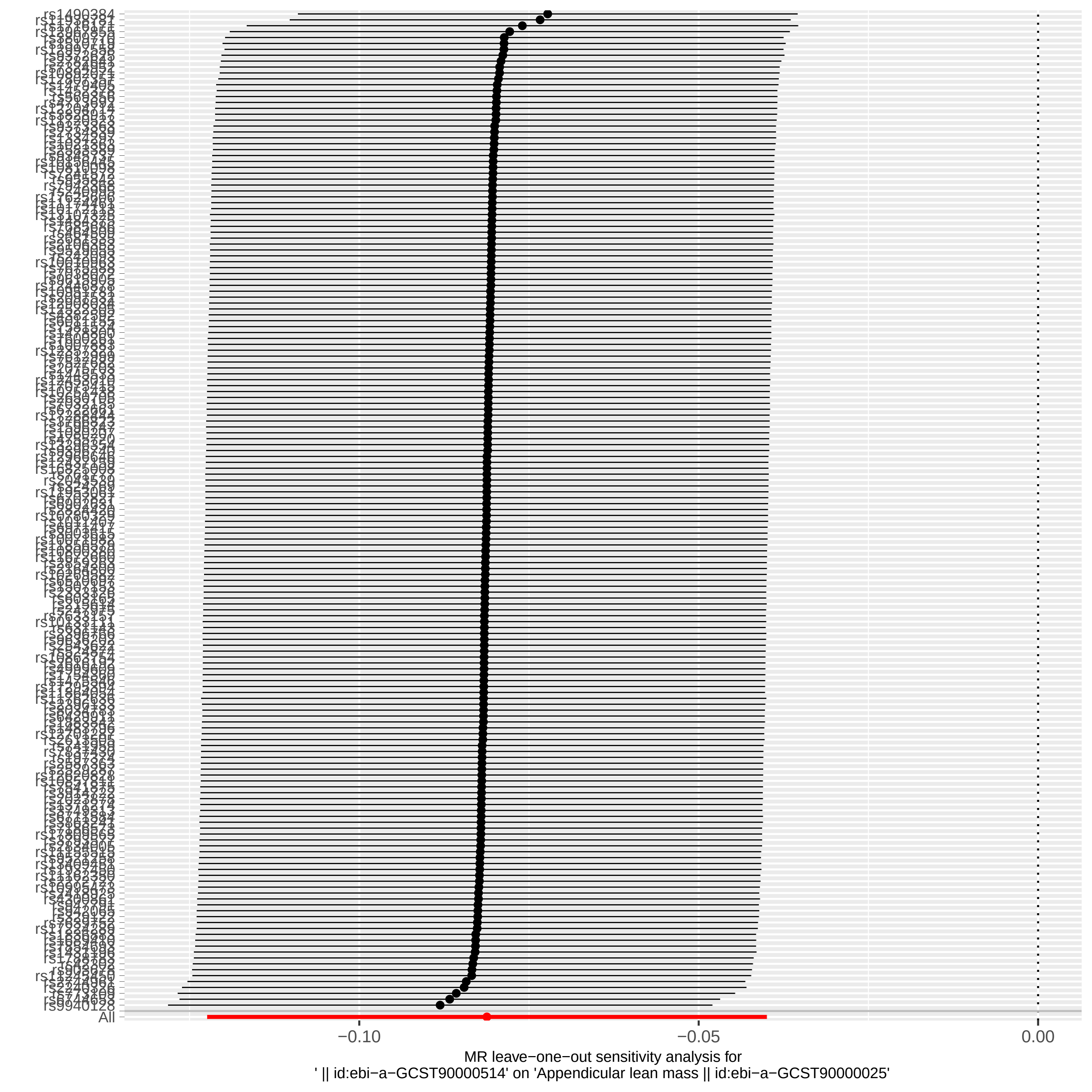
rs1490384
rs9940128
All
−0.10
−0.05
0.00
MR leave−one−out sensitivity analysis for
' || id:ebi−a−GCST90000514' on 'Appendicular lean mass || id:ebi−a−GCST90000025'

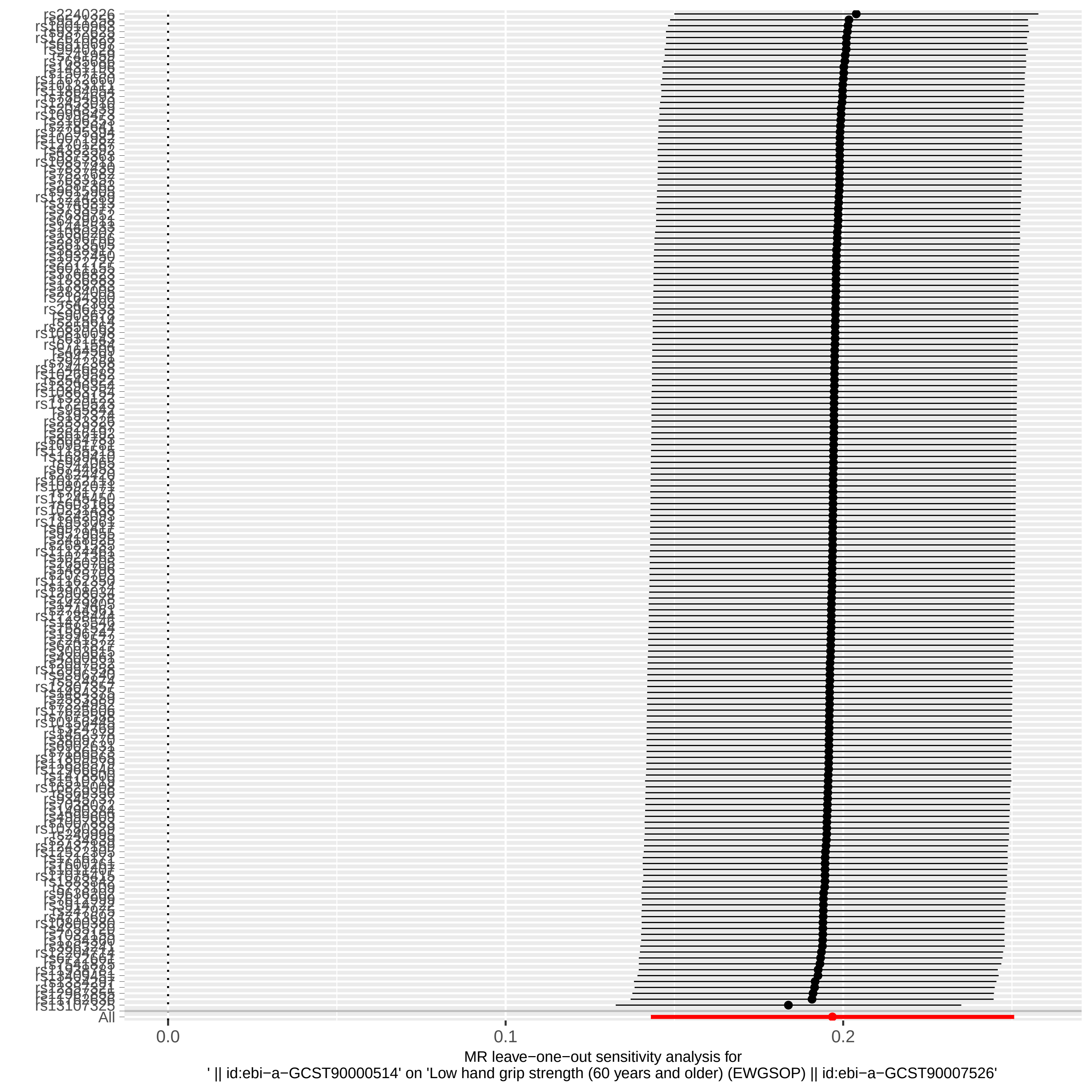
rs2240326
rs13107325
All
0.0
0.1
0.2
MR leave−one−out sensitivity analysis for
' || id:ebi−a−GCST90000514' on 'Low hand grip strength (60 years and older) (EWGSOP) || id:ebi−a−GCST90007526'

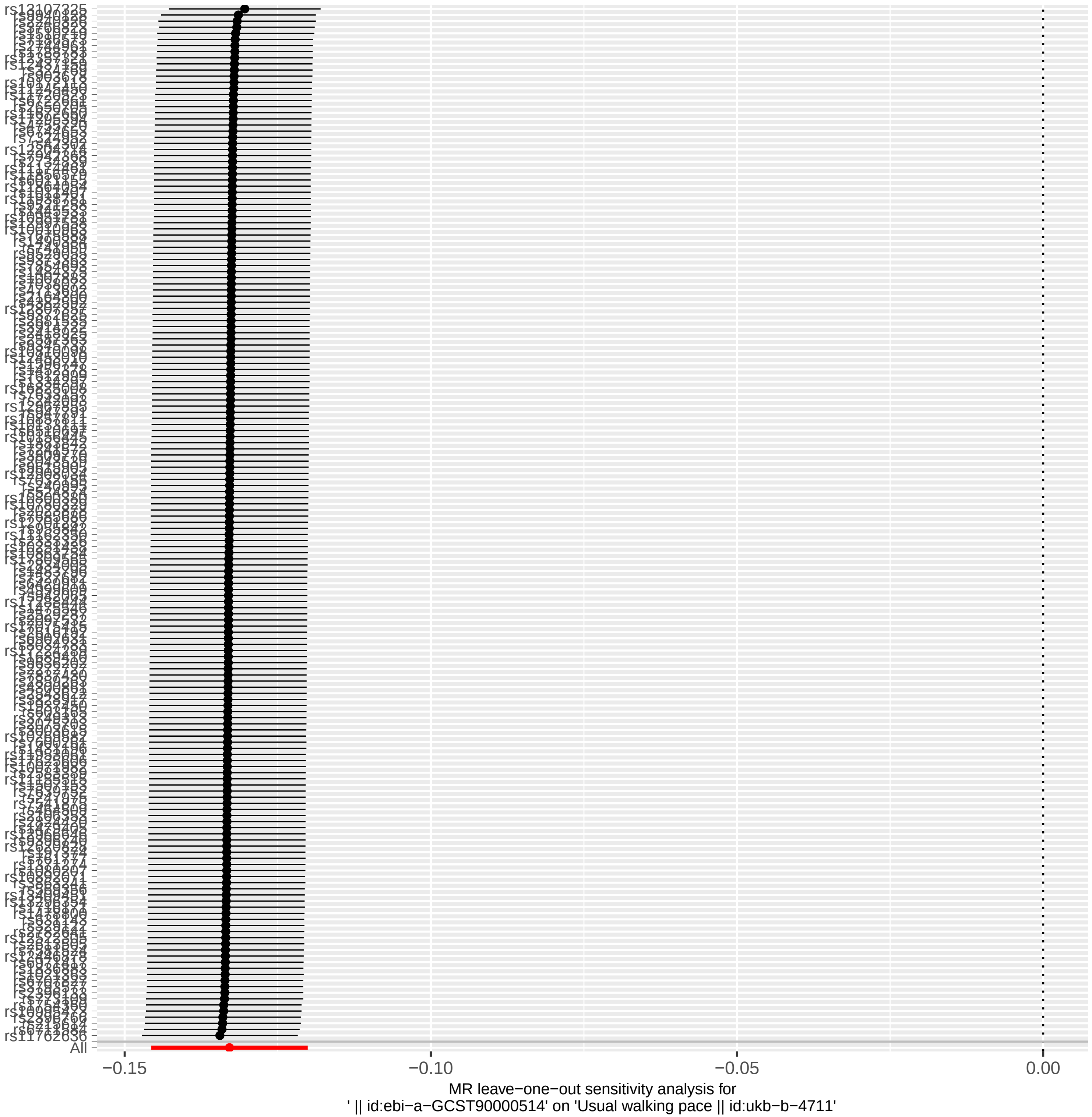
rs13107325
rs11702630
All
−0.15
−0.10
−0.05
0.00
MR leave−one−out sensitivity analysis for
' || id:ebi−a−GCST90000514' on 'Usual walking pace || id:ukb−b−4711'

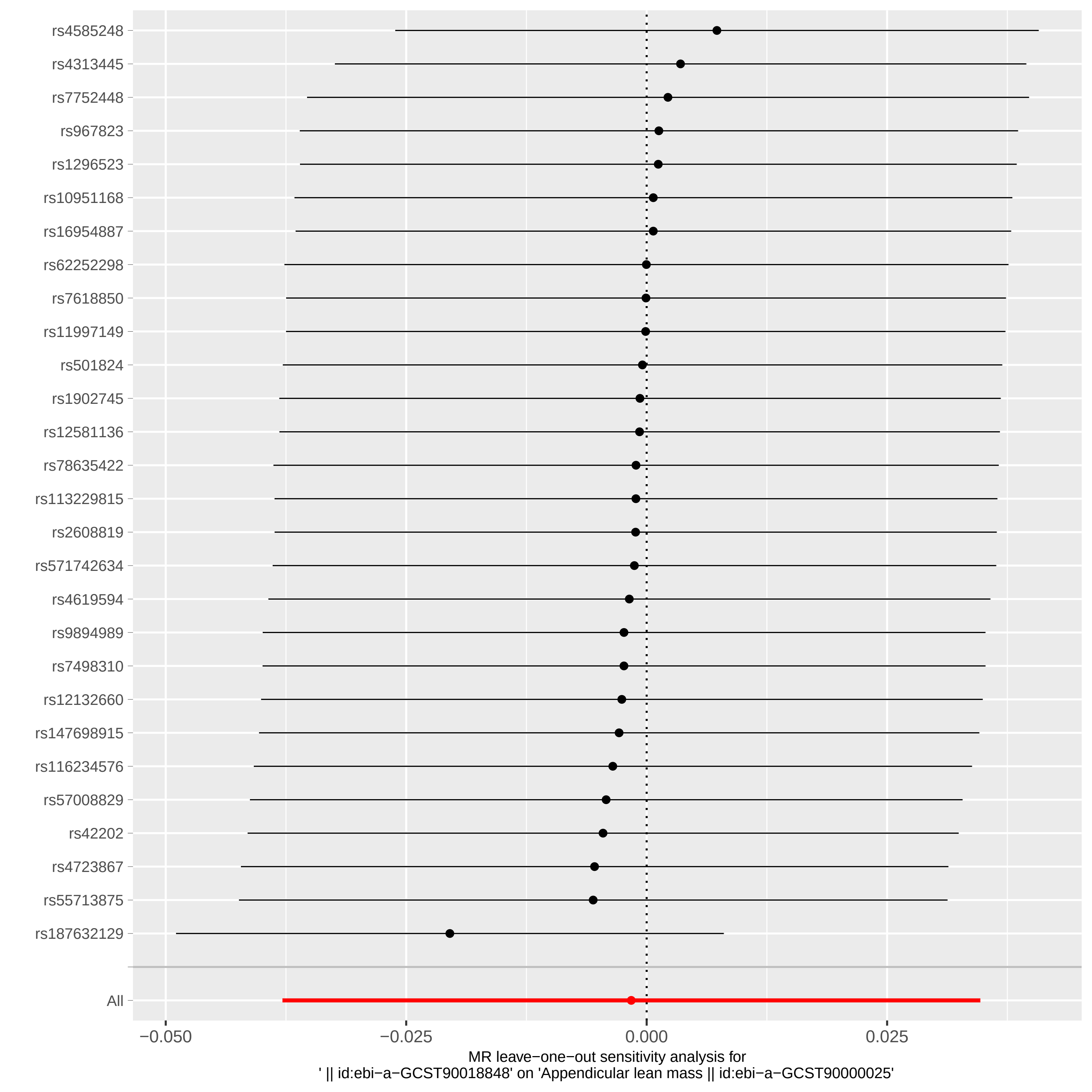

rs4585248
rs4313445
rs7752448
rs967823
rs1296523
rs10951168
rs16954887
rs62252298
rs7618850
rs11997149
rs501824
rs1902745
rs12581136
rs78635422
rs113229815
rs2608819
rs571742634
rs4619594
rs9894989
rs7498310
rs12132660
rs147698915
rs116234576
rs57008829
rs42202
rs4723867
rs55713875
rs187632129
All
−0.050
−0.025
0.000
0.025
MR leave−one−out sensitivity analysis for
' || id:ebi−a−GCST90018848' on 'Appendicular lean mass || id:ebi−a−GCST90000025'

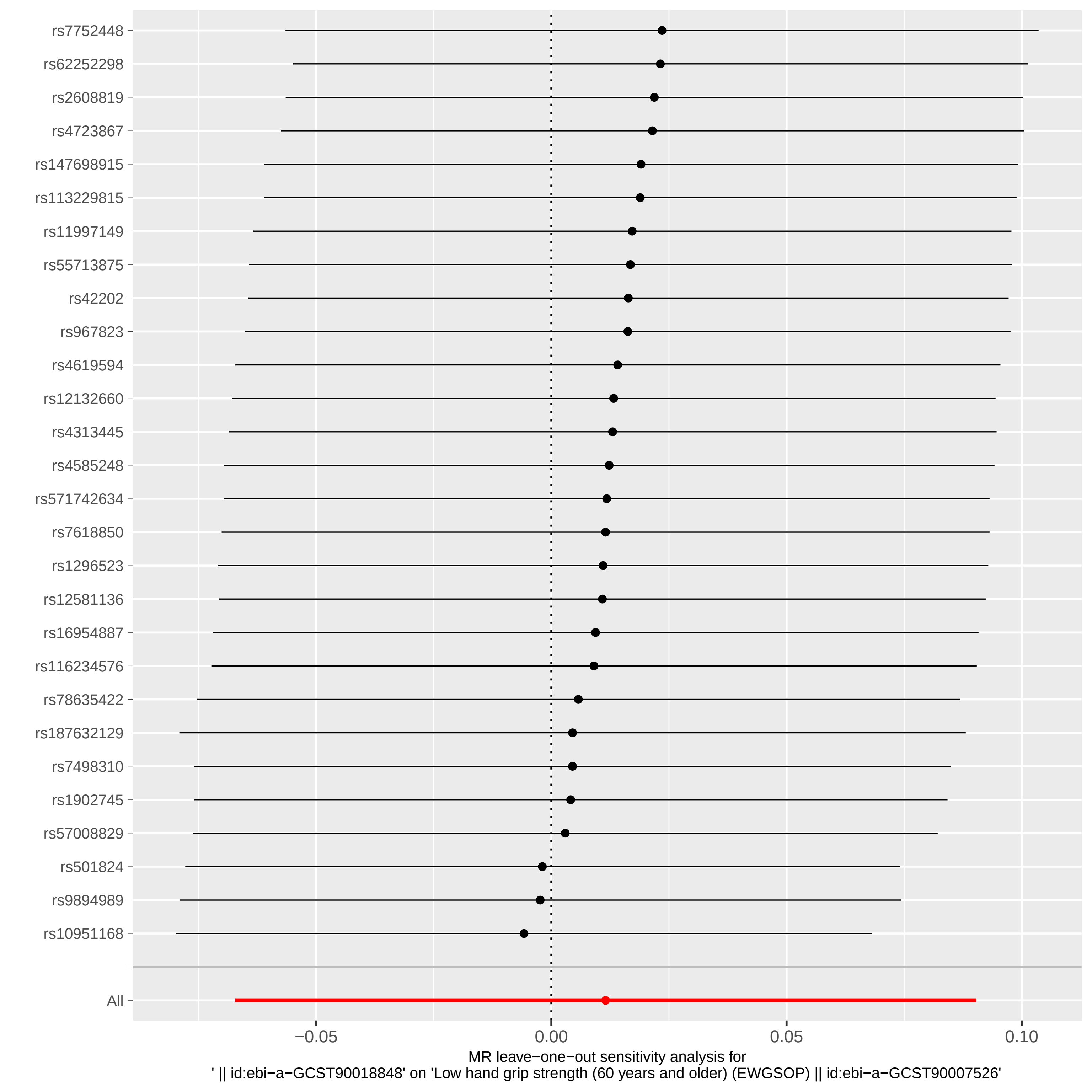
rs7752448
rs62252298
rs2608819
rs4723867
rs147698915
rs113229815
rs11997149
rs55713875
rs42202
rs967823
rs4619594
rs12132660
rs4313445
rs4585248
rs571742634
rs7618850
rs1296523
rs12581136
rs16954887
rs116234576
rs78635422
rs187632129
rs7498310
rs1902745
rs57008829
rs501824
rs9894989
rs10951168
All
−0.05
0.00
0.05
0.10
MR leave−one−out sensitivity analysis for
' || id:ebi−a−GCST90018848' on 'Low hand grip strength (60 years and older) (EWGSOP) || id:ebi−a−GCST90007526'

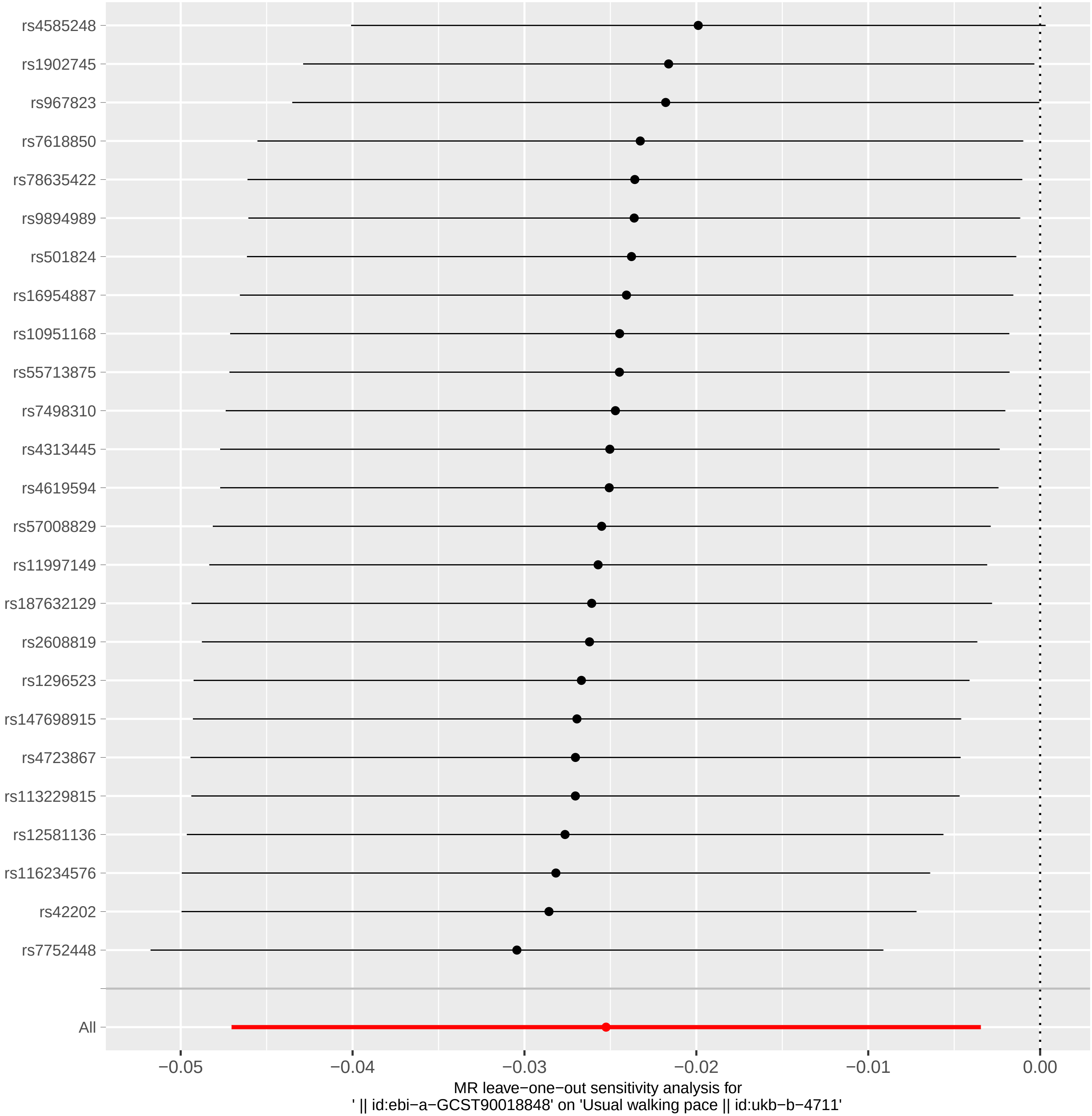
rs4585248
rs1902745
rs967823
rs7618850
rs78635422
rs9894989
rs501824
rs16954887
rs10951168
rs55713875
rs7498310
rs4313445
rs4619594
rs57008829
rs11997149
rs187632129
rs2608819
rs1296523
rs147698915
rs4723867
rs113229815
rs12581136
rs116234576
rs42202
rs7752448
All
−0.05
−0.04
−0.03
−0.02
−0.01
0.00
MR leave−one−out sensitivity analysis for
' || id:ebi−a−GCST90018848' on 'Usual walking pace || id:ukb−b−4711'

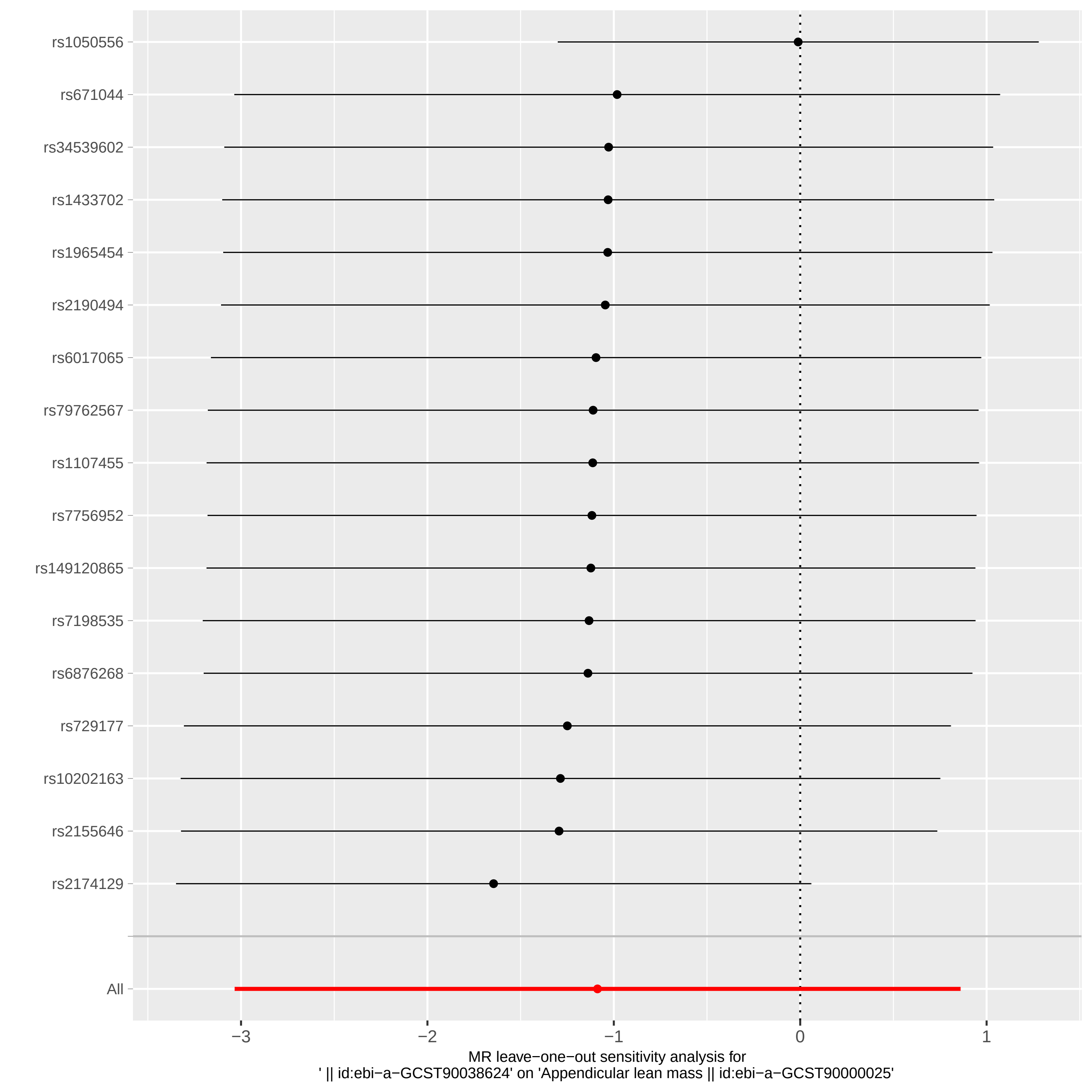
rs1050556
rs671044
rs34539602
rs1433702
rs1965454
rs2190494
rs6017065
rs79762567
rs1107455
rs7756952
rs149120865
rs7198535
rs6876268
rs729177
rs10202163
rs2155646
rs2174129
All
−3
−2
−1
0
1
MR leave−one−out sensitivity analysis for
' || id:ebi−a−GCST90038624' on 'Appendicular lean mass || id:ebi−a−GCST90000025'

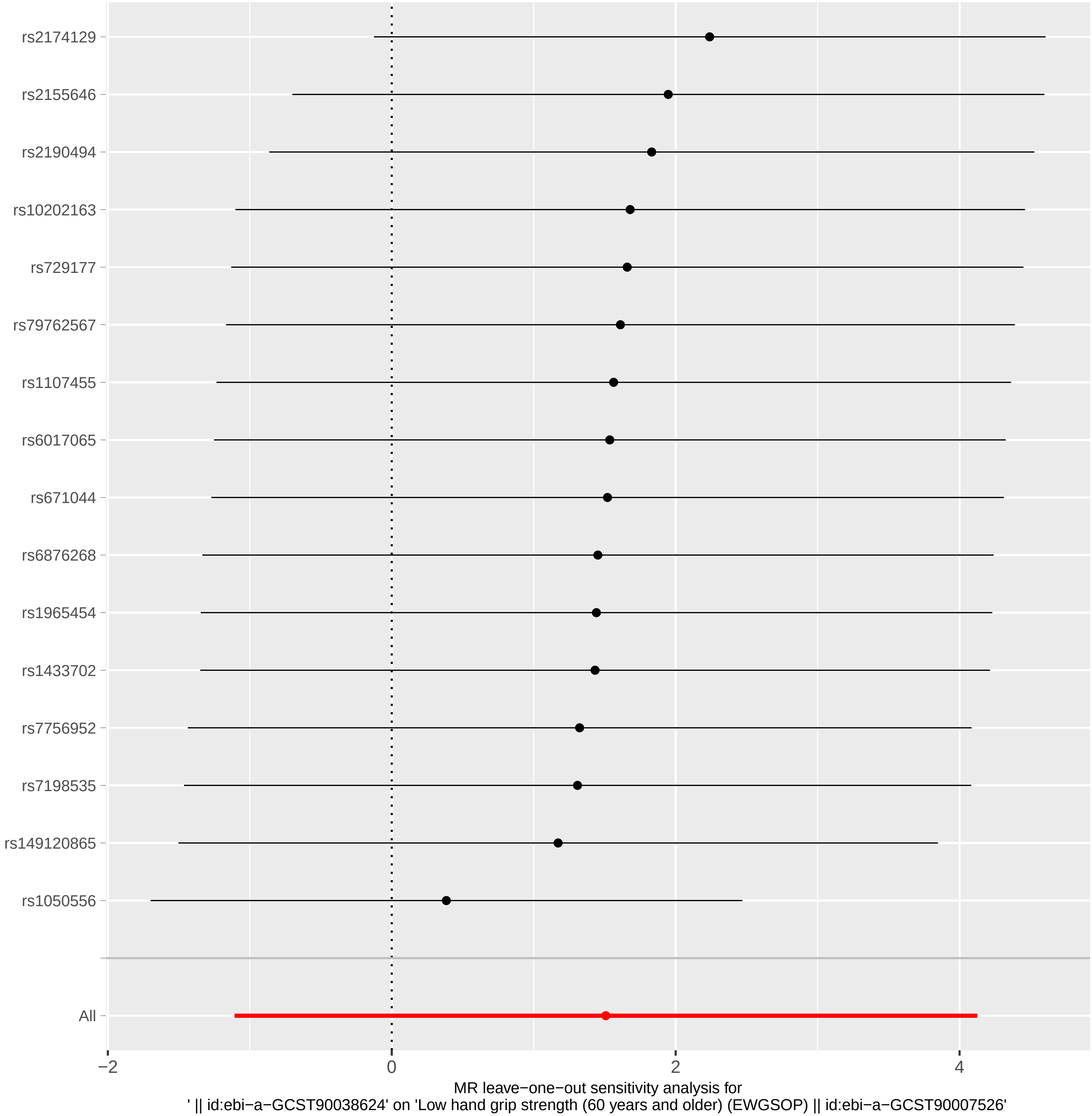

rs2174129
rs2155646
rs2190494
rs10202163
rs729177
rs79762567
rs1107455
rs6017065
rs671044
rs6876268
rs1965454
rs1433702
rs7756952
rs7198535
rs149120865
rs1050556
All
−2
0
2
4
MR leave−one−out sensitivity analysis for
' || id:ebi−a−GCST90038624' on 'Low hand grip strength (60 years and older) (EWGSOP) || id:ebi−a−GCST90007526'

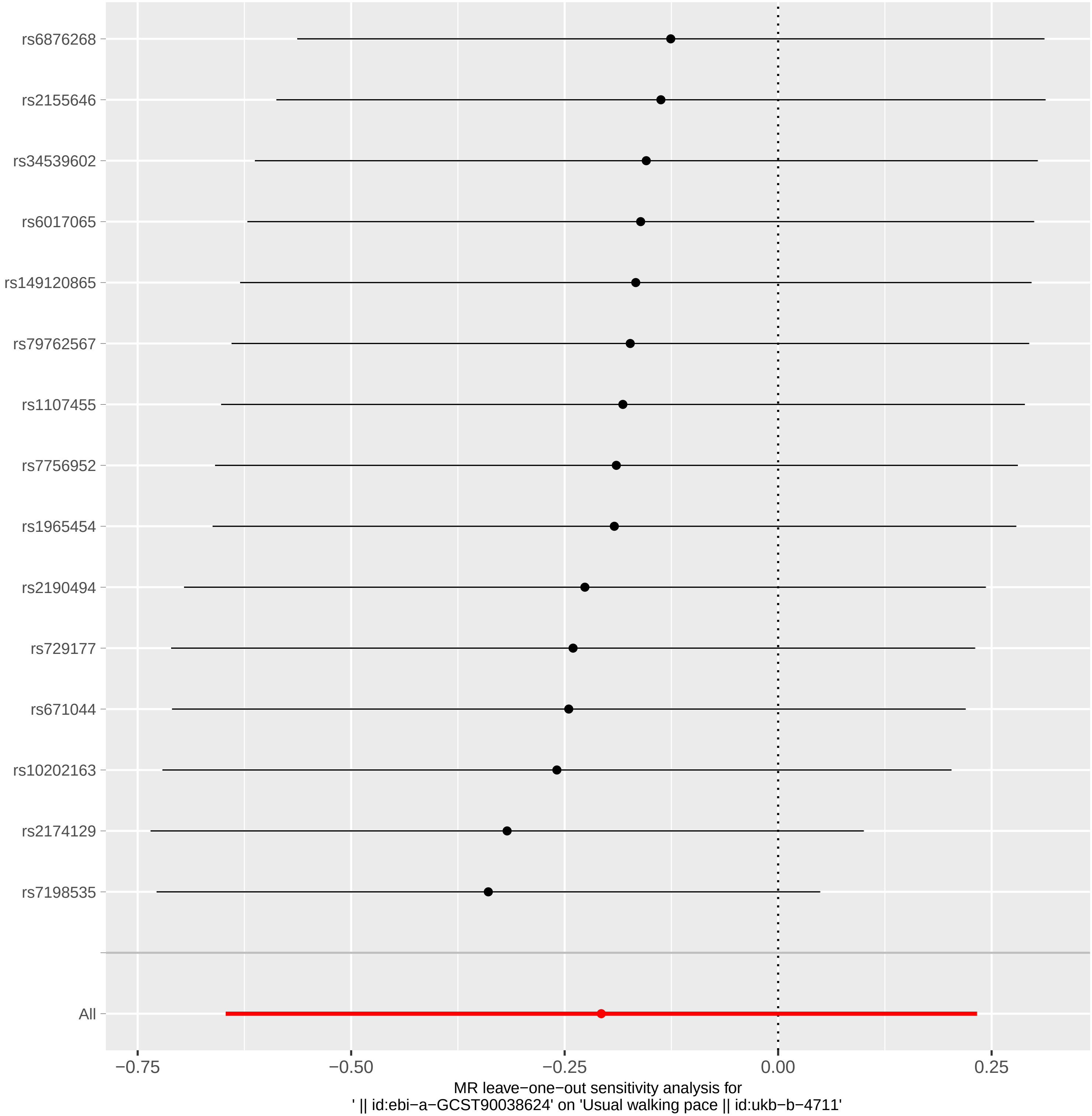
rs6876268
rs2155646
rs34539602
rs6017065
rs149120865
rs79762567
rs1107455
rs7756952
rs1965454
rs2190494
rs729177
rs671044
rs10202163
rs2174129
rs7198535
All
−0.75
−0.50
−0.25
0.00
0.25
MR leave−one−out sensitivity analysis for
' || id:ebi−a−GCST90038624' on 'Usual walking pace || id:ukb−b−4711'

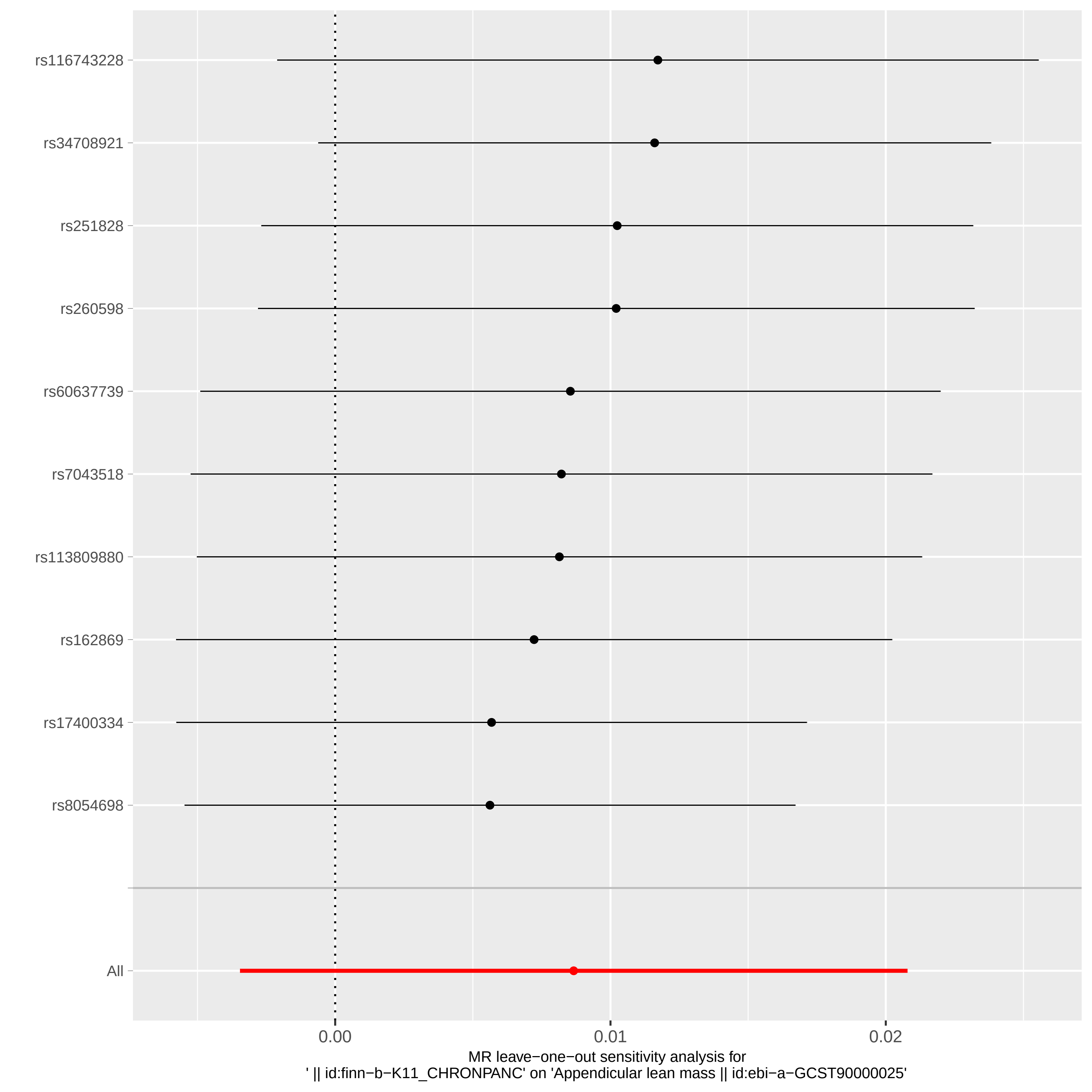

rs116743228
rs34708921
rs251828
rs260598
rs60637739
rs7043518
rs113809880
rs162869
rs17400334
rs8054698
All
0.00
0.01
0.02
MR leave−one−out sensitivity analysis for
' || id:finn−b−K11_CHRONPANC' on 'Appendicular lean mass || id:ebi−a−GCST90000025'

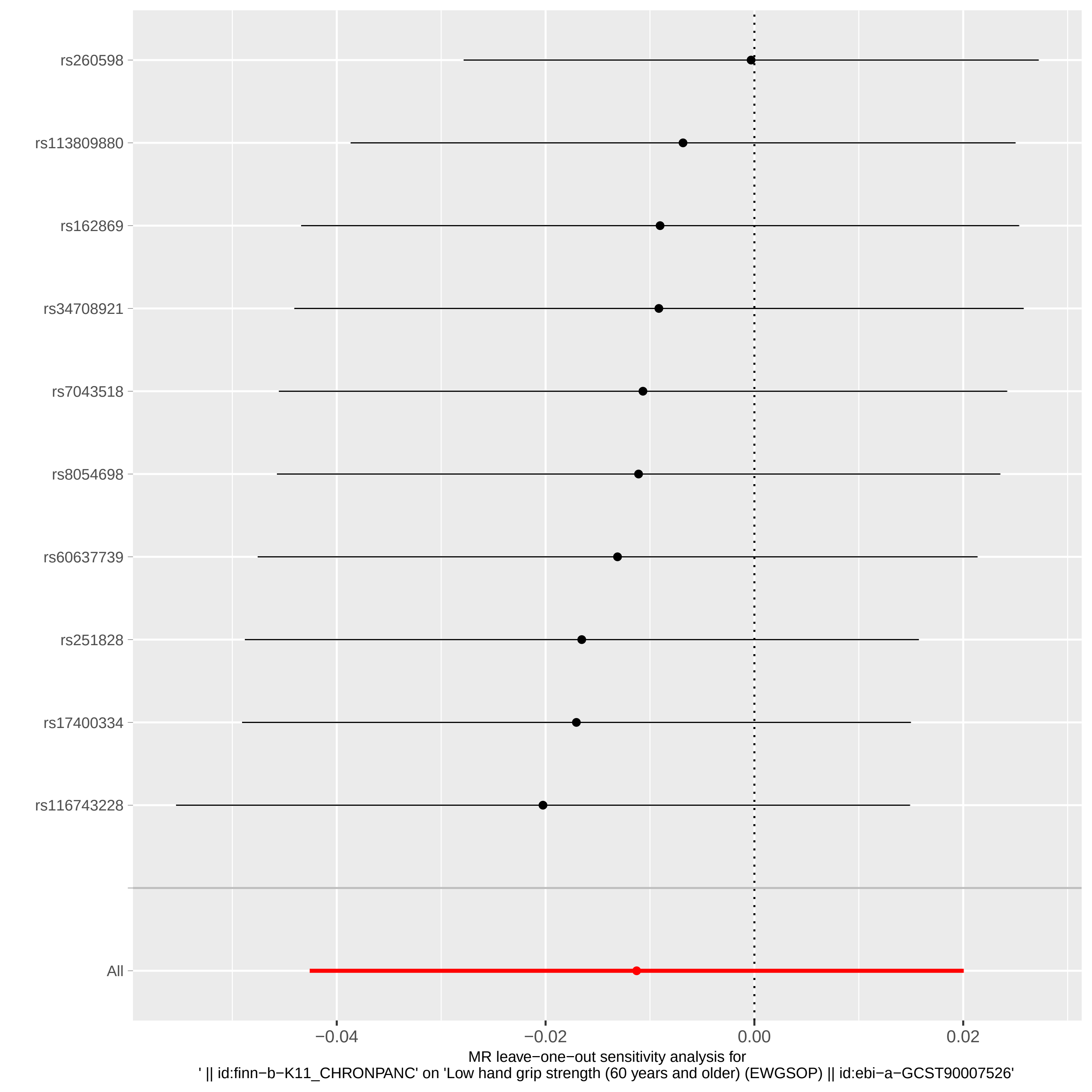
rs260598
rs113809880
rs162869
rs34708921
rs7043518
rs8054698
rs60637739
rs251828
rs17400334
rs116743228
All
−0.04
−0.02
0.00
0.02
MR leave−one−out sensitivity analysis for
' || id:finn−b−K11_CHRONPANC' on 'Low hand grip strength (60 years and older) (EWGSOP) || id:ebi−a−GCST90007526'

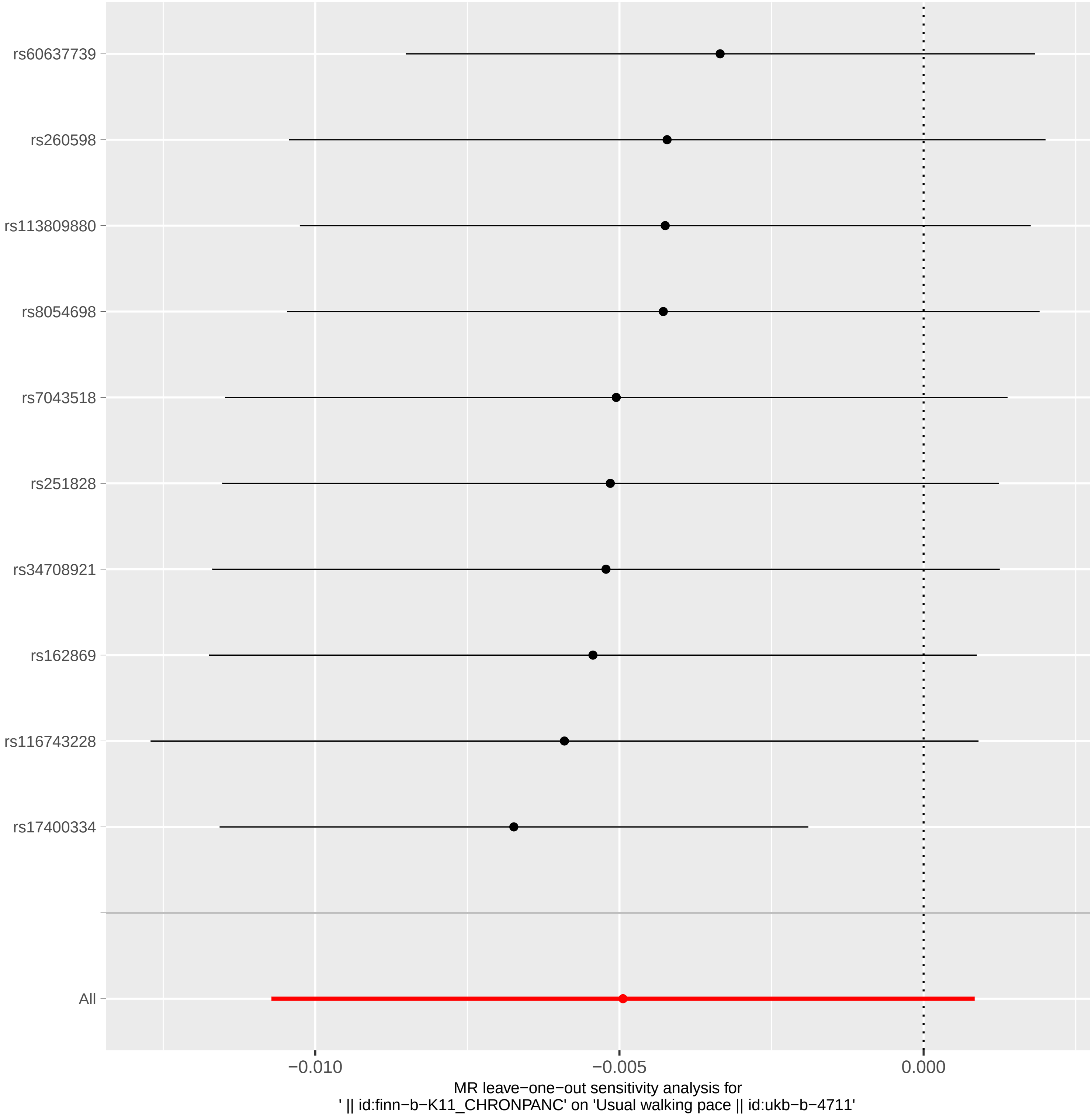

rs60637739
rs260598
rs113809880
rs8054698
rs7043518
rs251828
rs34708921
rs162869
rs116743228
rs17400334
All
−0.010
−0.005
0.000
MR leave−one−out sensitivity analysis for
' || id:finn−b−K11_CHRONPANC' on 'Usual walking pace || id:ukb−b−4711'

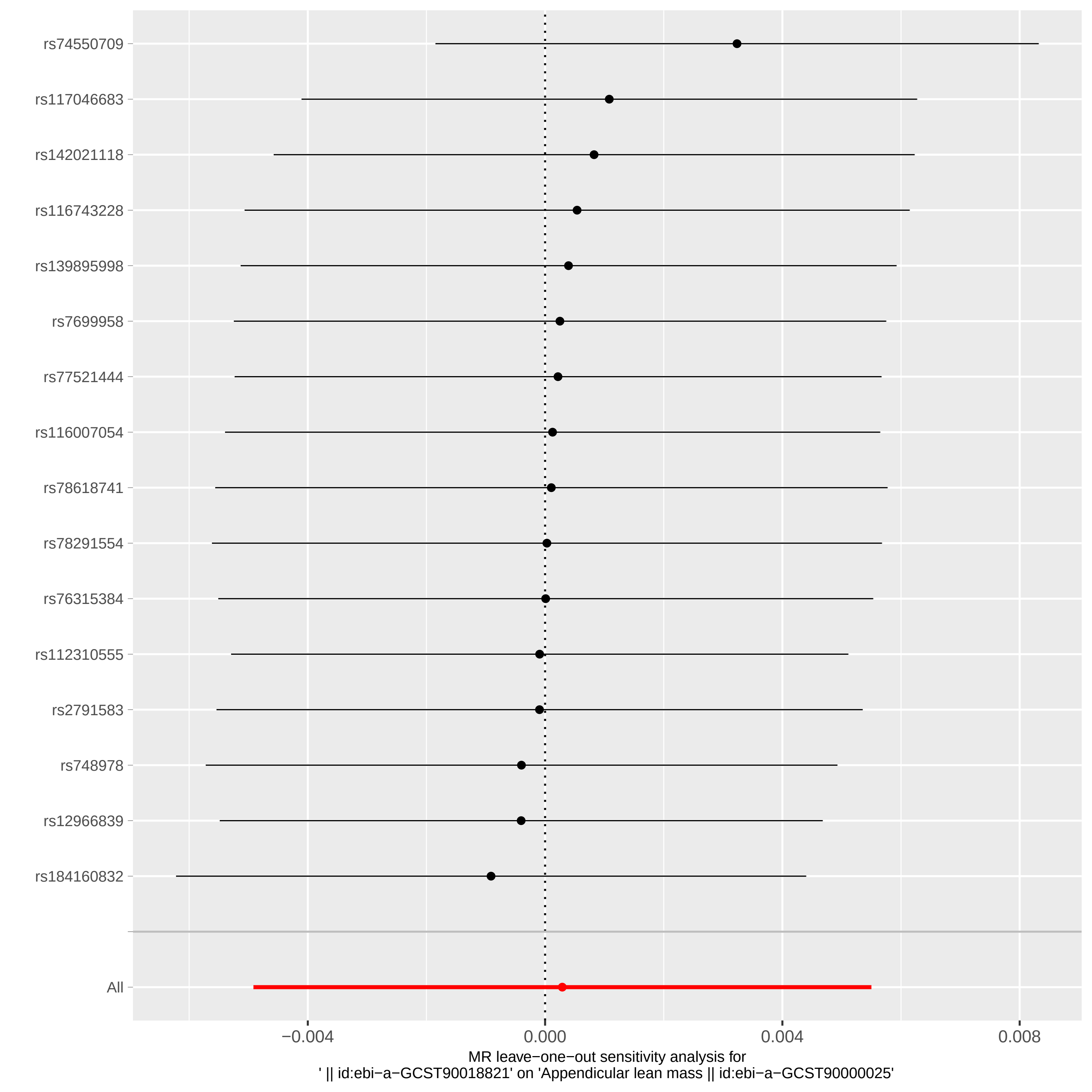

rs74550709
rs117046683
rs142021118
rs116743228
rs139895998
rs7699958
rs77521444
rs116007054
rs78618741
rs78291554
rs76315384
rs112310555
rs2791583
rs748978
rs12966839
rs184160832
All
−0.004
0.000
0.004
0.008
MR leave−one−out sensitivity analysis for
' || id:ebi−a−GCST90018821' on 'Appendicular lean mass || id:ebi−a−GCST90000025'

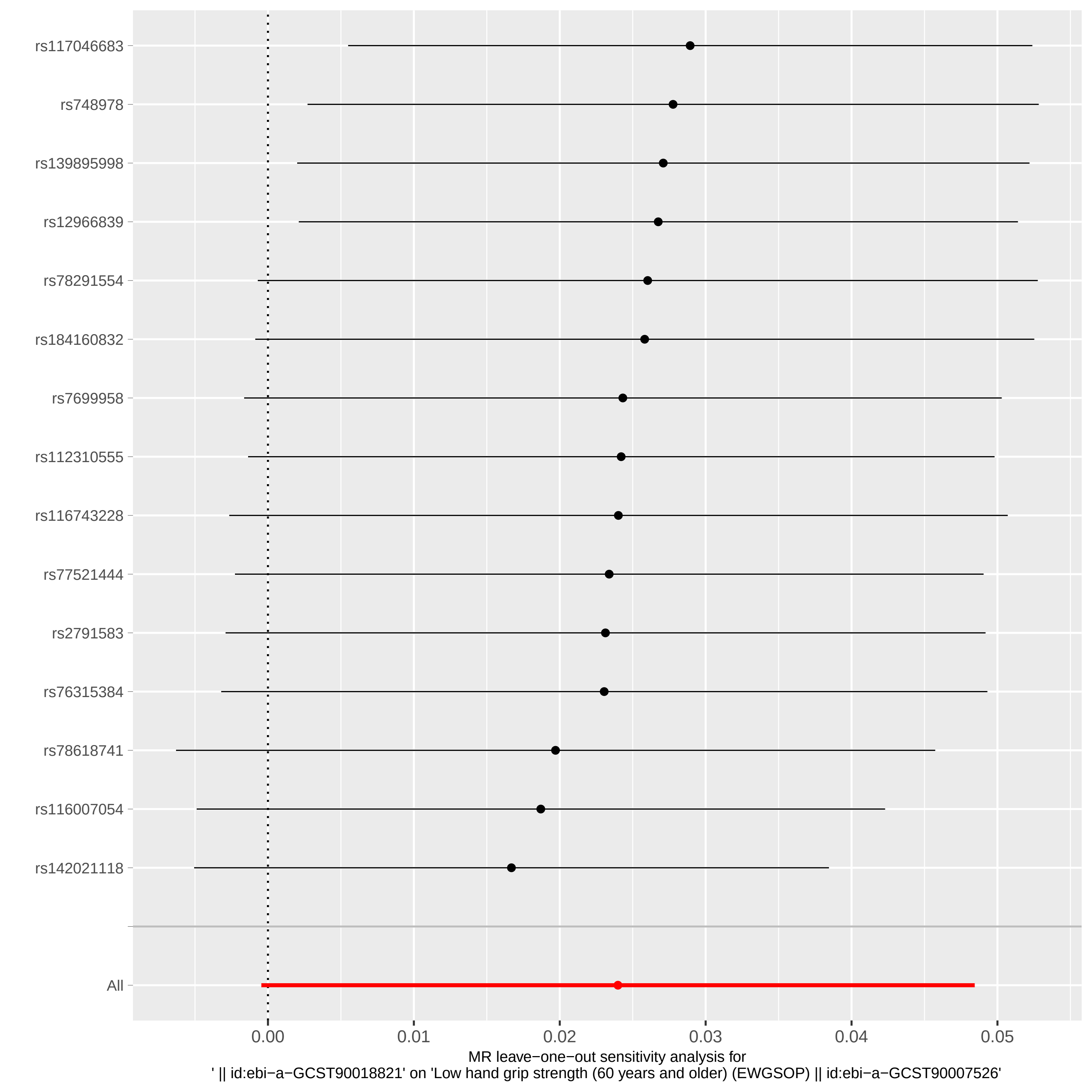

rs117046683
rs748978
rs139895998
rs12966839
rs78291554
rs184160832
rs7699958
rs112310555
rs116743228
rs77521444
rs2791583
rs76315384
rs78618741
rs116007054
rs142021118
All
0.00
0.01
0.02
0.03
0.04
0.05
MR leave−one−out sensitivity analysis for
' || id:ebi−a−GCST90018821' on 'Low hand grip strength (60 years and older) (EWGSOP) || id:ebi−a−GCST90007526'

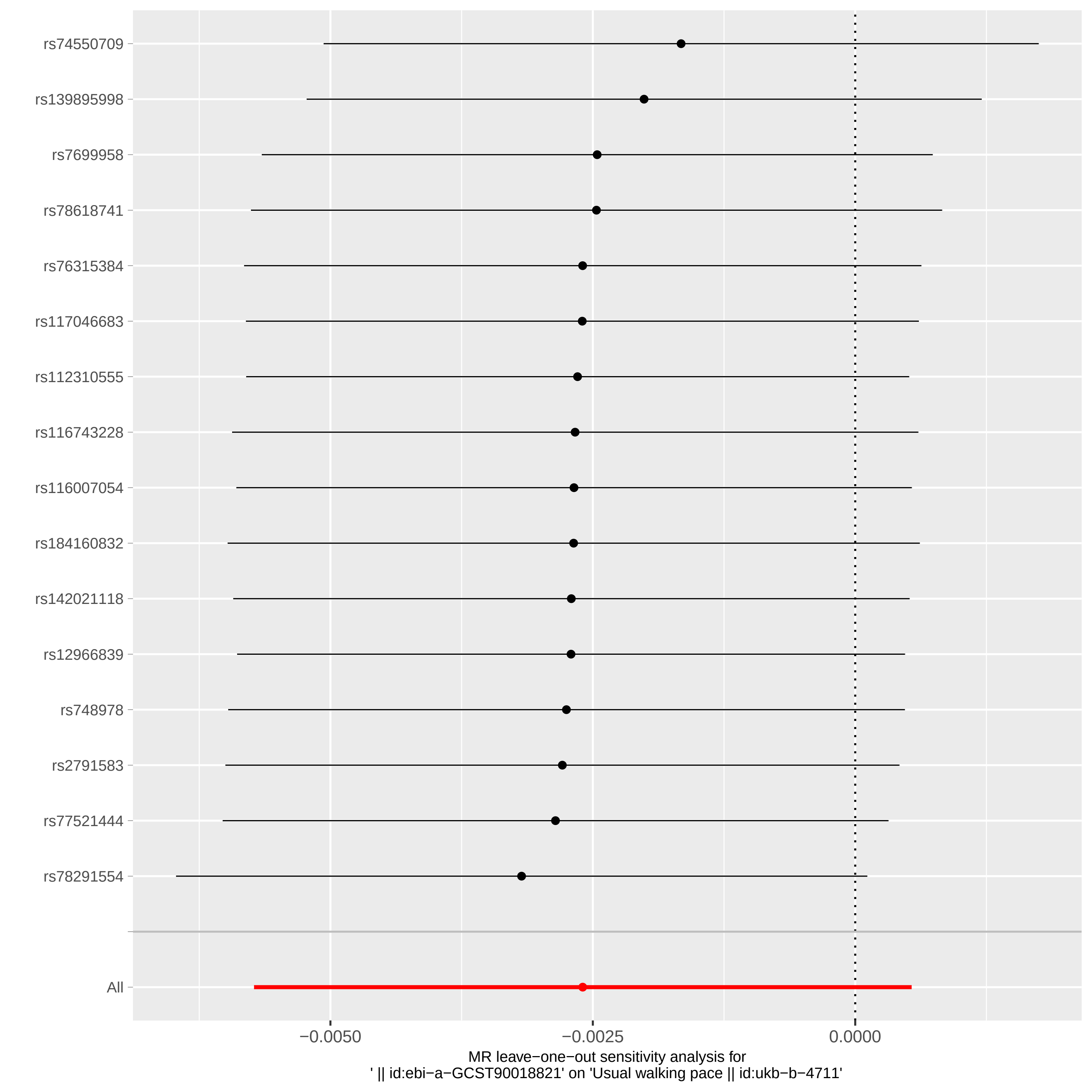

rs74550709
rs139895998
rs7699958
rs78618741
rs76315384
rs117046683
rs112310555
rs116743228
rs116007054
rs184160832
rs142021118
rs12966839
rs748978
rs2791583
rs77521444
rs78291554
All
−0.0050
−0.0025
0.0000
MR leave−one−out sensitivity analysis for
' || id:ebi−a−GCST90018821' on 'Usual walking pace || id:ukb−b−4711'

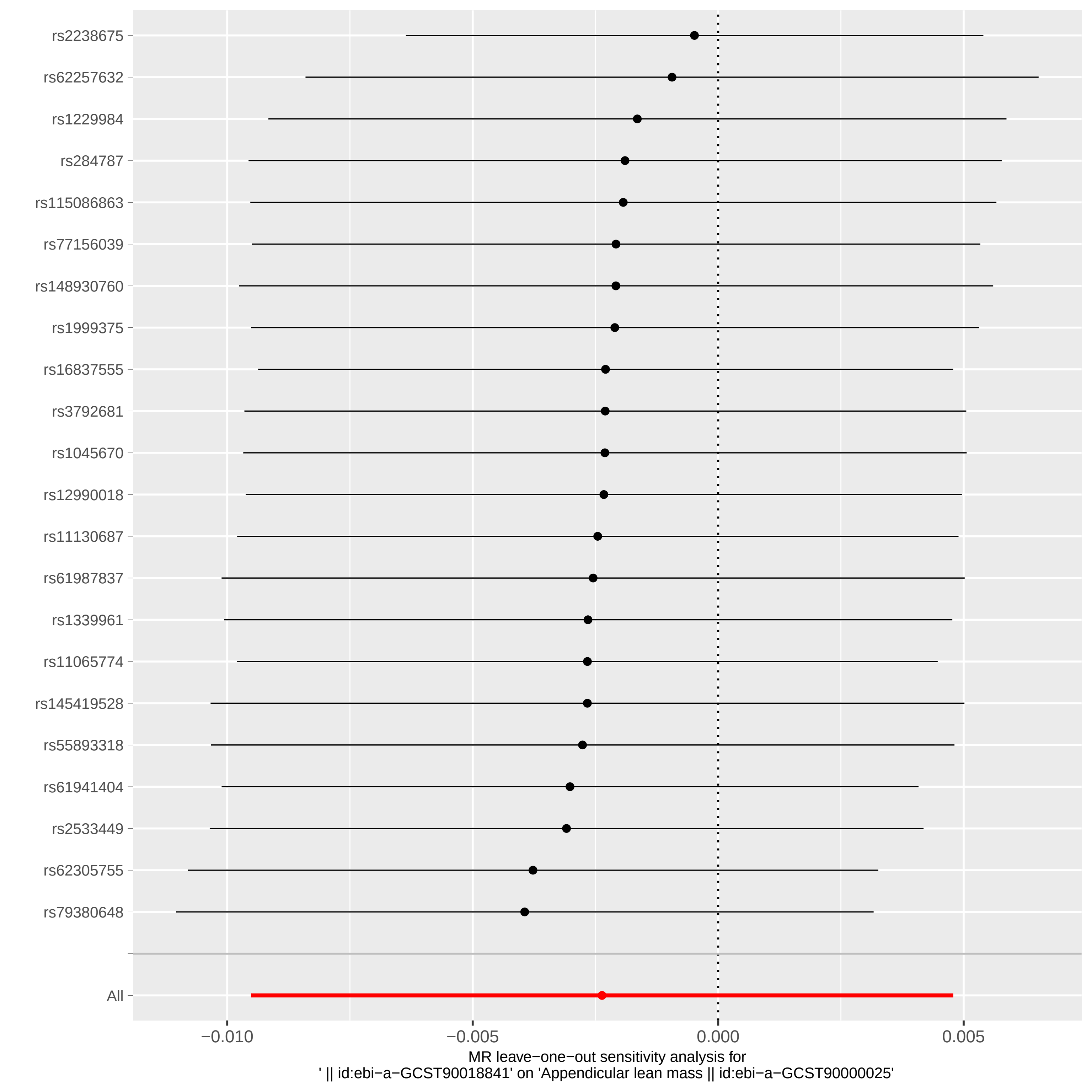
rs2238675
rs62257632
rs1229984
rs284787
rs115086863
rs77156039
rs148930760
rs1999375
rs16837555
rs3792681
rs1045670
rs12990018
rs11130687
rs61987837
rs1339961
rs11065774
rs145419528
rs55893318
rs61941404
rs2533449
rs62305755
rs79380648
All
−0.010
−0.005
0.000
0.005
MR leave−one−out sensitivity analysis for
' || id:ebi−a−GCST90018841' on 'Appendicular lean mass || id:ebi−a−GCST90000025'

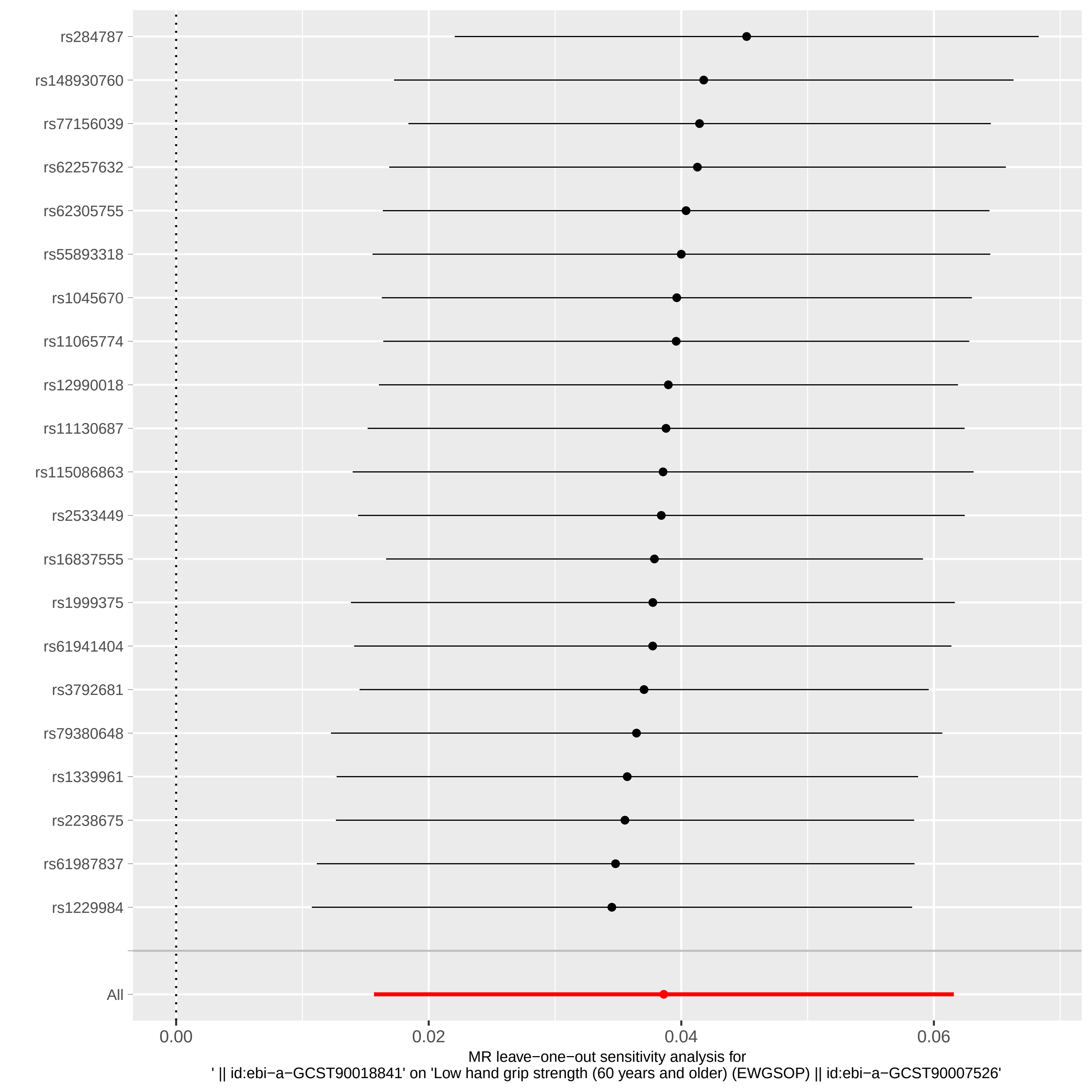
rs284787
rs148930760
rs77156039
rs62257632
rs62305755
rs55893318
rs1045670
rs11065774
rs12990018
rs11130687
rs115086863
rs2533449
rs16837555
rs1999375
rs61941404
rs3792681
rs79380648
rs1339961
rs2238675
rs61987837
rs1229984
All
0.00
0.02
0.04
0.06
MR leave−one−out sensitivity analysis for
' || id:ebi−a−GCST90018841' on 'Low hand grip strength (60 years and older) (EWGSOP) || id:ebi−a−GCST90007526'

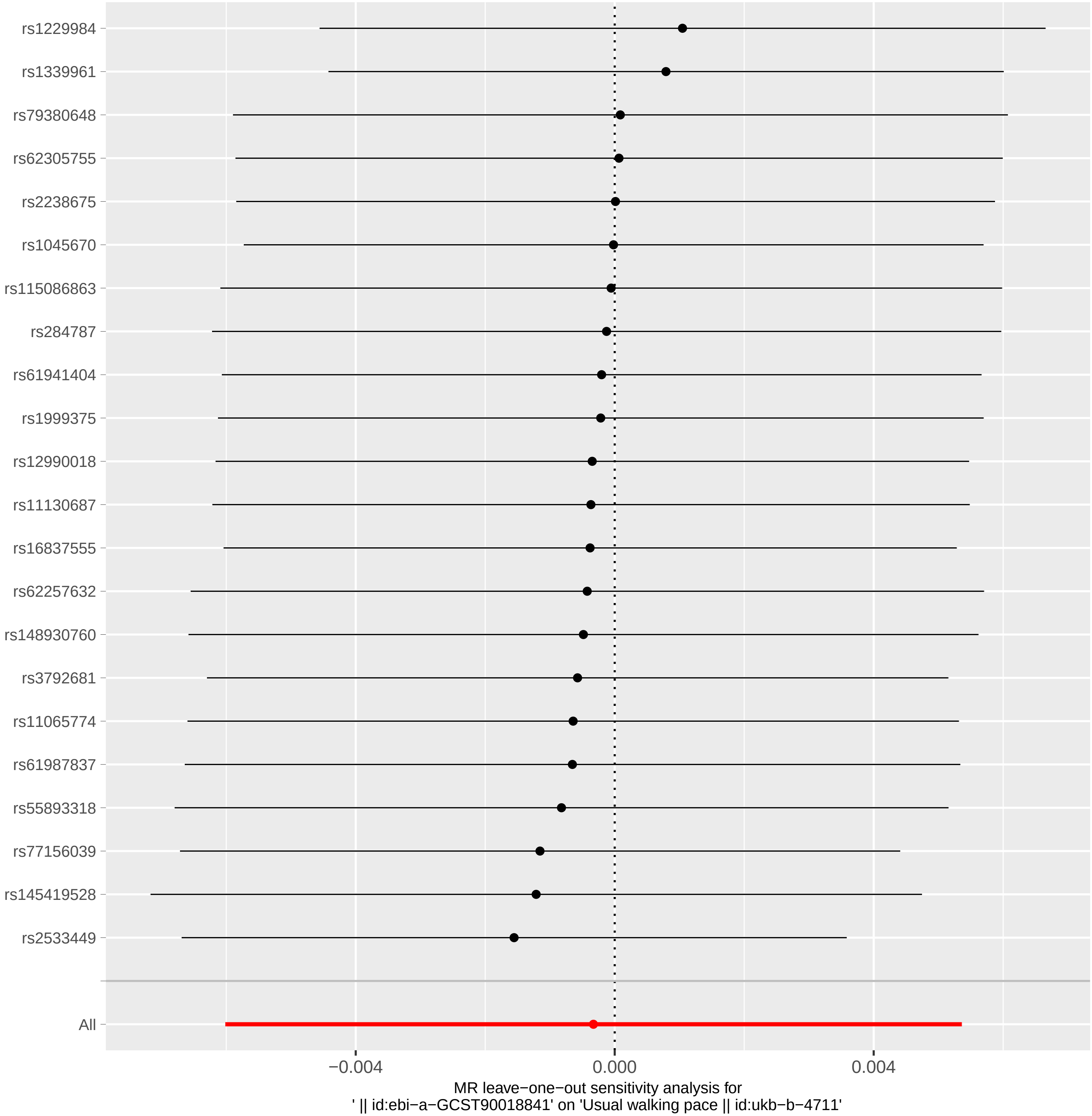
rs1229984
rs1339961
rs79380648
rs62305755
rs2238675
rs1045670
rs115086863
rs284787
rs61941404
rs1999375
rs12990018
rs11130687
rs16837555
rs62257632
rs148930760
rs3792681
rs11065774
rs61987837
rs55893318
rs77156039
rs145419528
rs2533449
All
−0.004
0.000
0.004
MR leave−one−out sensitivity analysis for
' || id:ebi−a−GCST90018841' on 'Usual walking pace || id:ukb−b−4711'

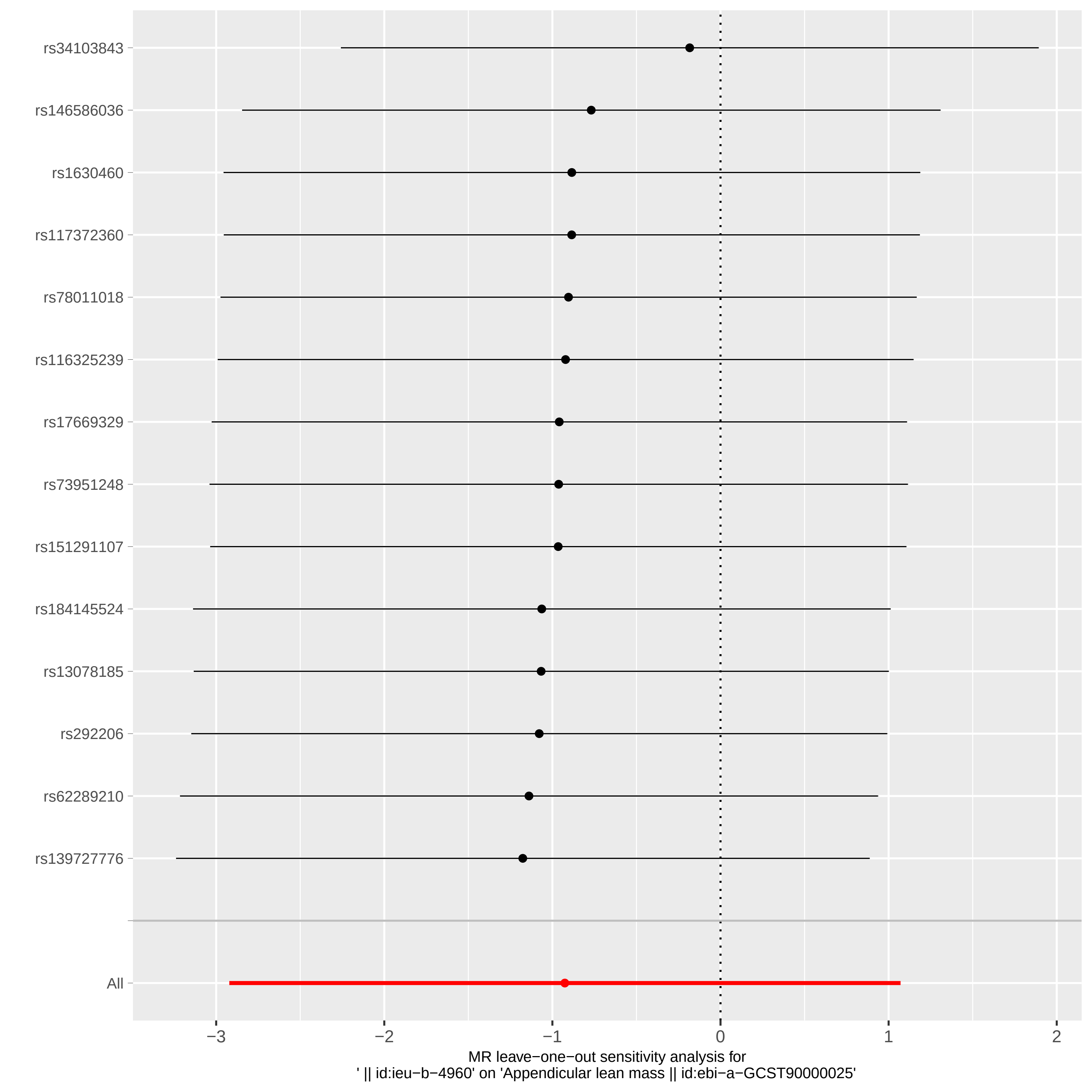
rs34103843
rs146586036
rs1630460
rs117372360
rs78011018
rs116325239
rs17669329
rs73951248
rs151291107
rs184145524
rs13078185
rs292206
rs62289210
rs139727776
All
−3
−2
−1
0
1
2
MR leave−one−out sensitivity analysis for
' || id:ieu−b−4960' on 'Appendicular lean mass || id:ebi−a−GCST90000025'

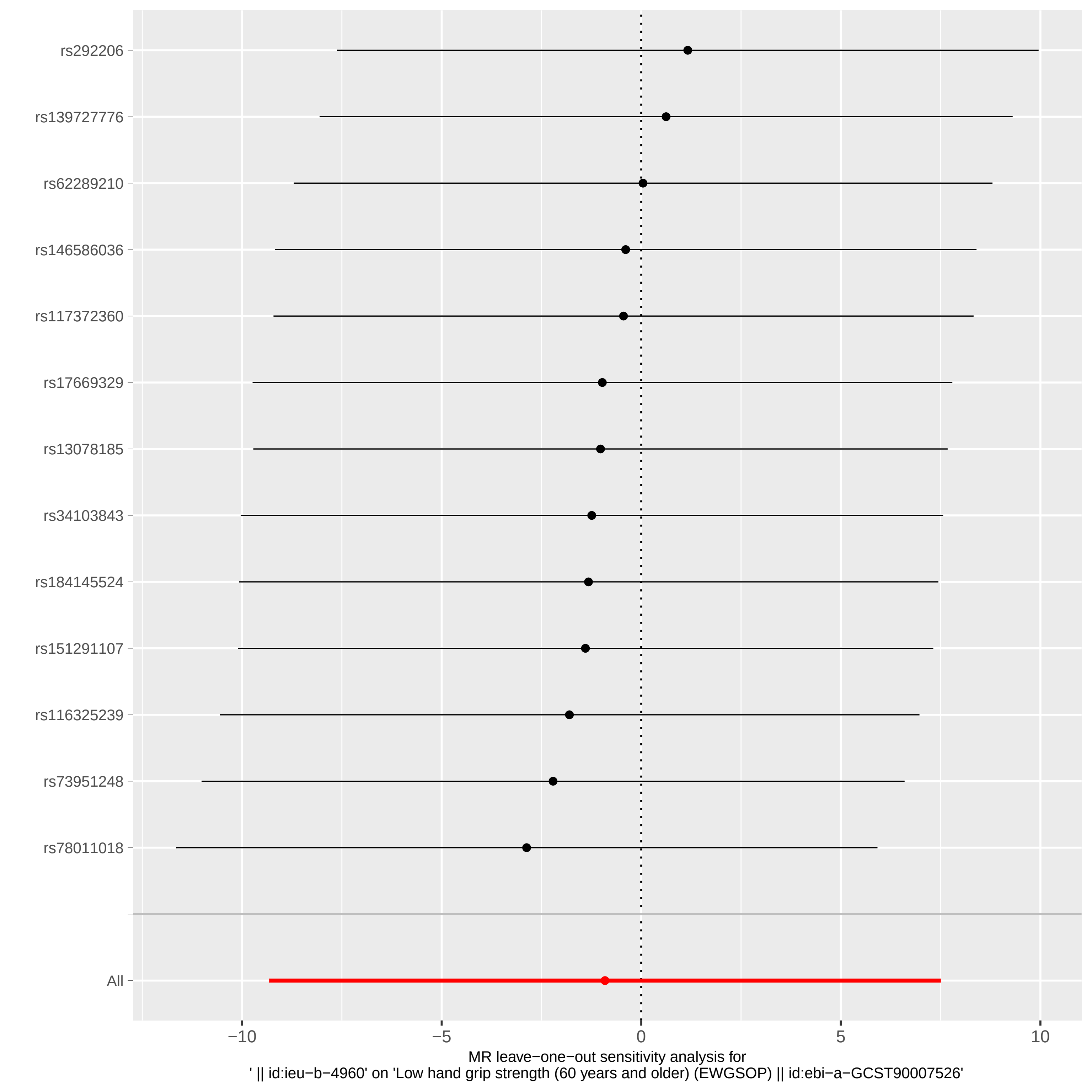
rs292206
rs139727776
rs62289210
rs146586036
rs117372360
rs17669329
rs13078185
rs34103843
rs184145524
rs151291107
rs116325239
rs73951248
rs78011018
All
−10
−5
0
5
10
MR leave−one−out sensitivity analysis for
' || id:ieu−b−4960' on 'Low hand grip strength (60 years and older) (EWGSOP) || id:ebi−a−GCST90007526'

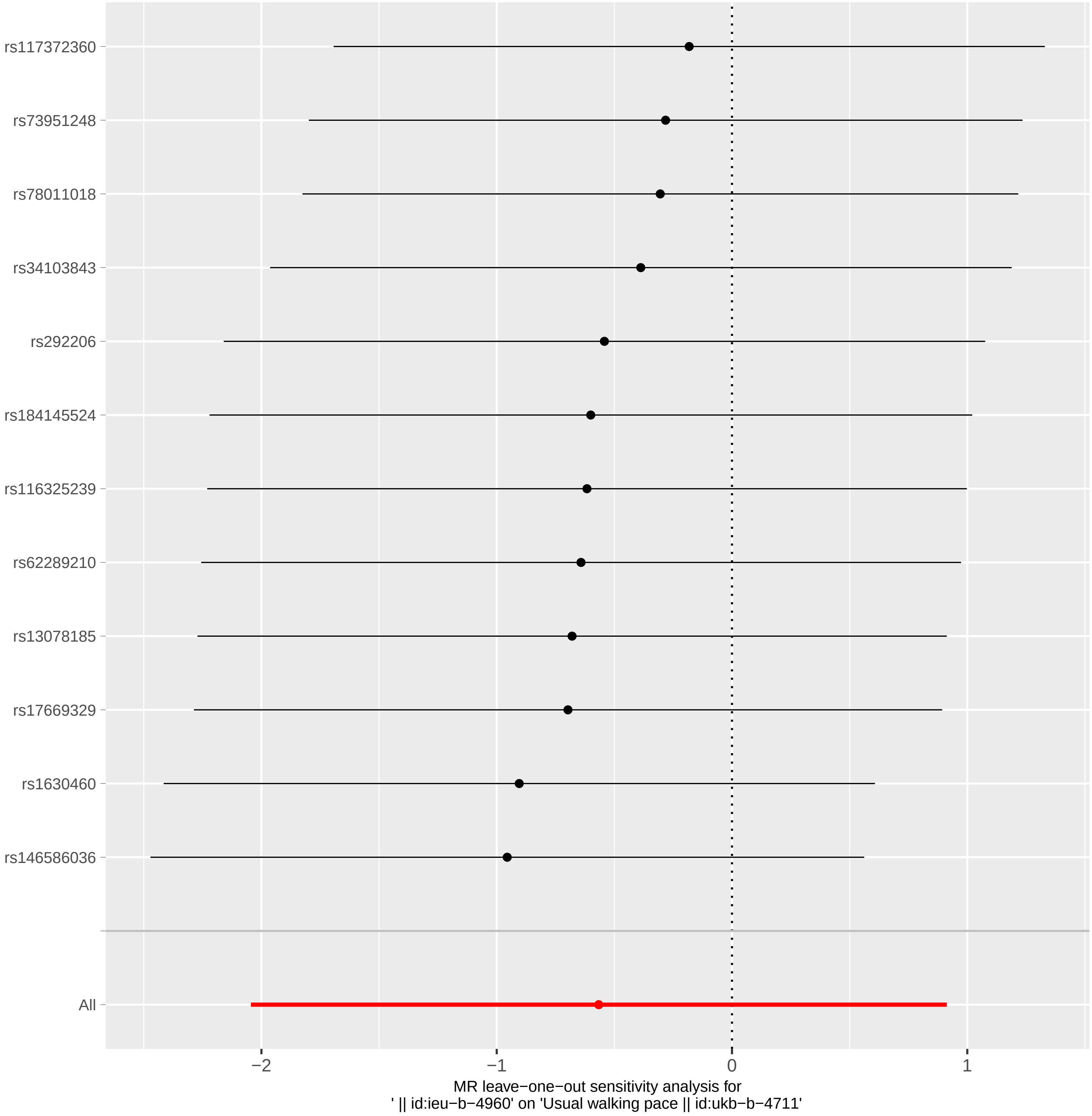

rs117372360
rs73951248
rs78011018
rs34103843
rs292206
rs184145524
rs116325239
rs62289210
rs13078185
rs17669329
rs1630460
rs146586036
All
−2
−1
0
1
MR leave−one−out sensitivity analysis for
' || id:ieu−b−4960' on 'Usual walking pace || id:ukb−b−4711'

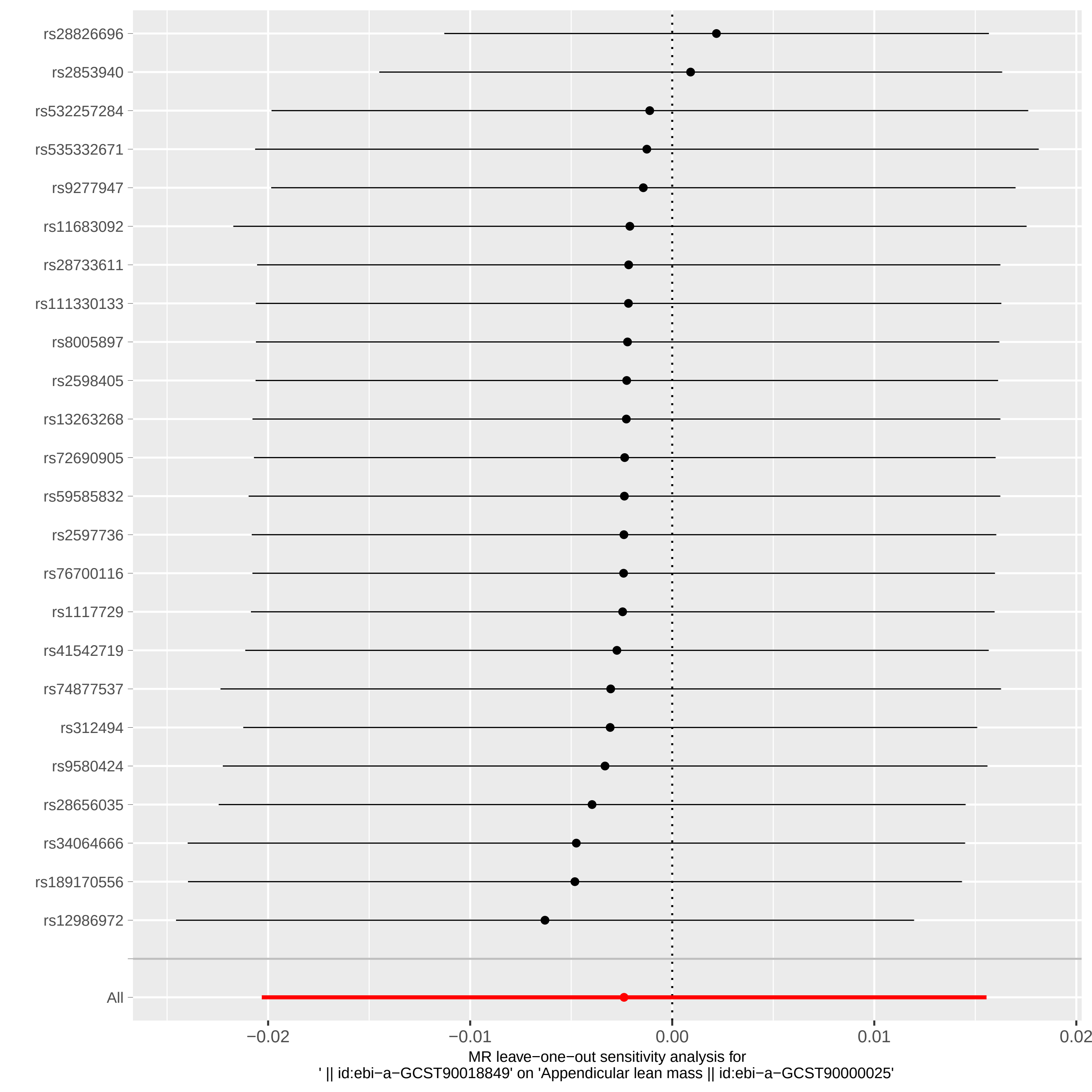
rs28826696
rs2853940
rs532257284
rs535332671
rs9277947
rs11683092
rs28733611
rs111330133
rs8005897
rs2598405
rs13263268
rs72690905
rs59585832
rs2597736
rs76700116
rs1117729
rs41542719
rs74877537
rs312494
rs9580424
rs28656035
rs34064666
rs189170556
rs12986972
All
−0.02
−0.01
0.00
0.01
0.02
MR leave−one−out sensitivity analysis for
' || id:ebi−a−GCST90018849' on 'Appendicular lean mass || id:ebi−a−GCST90000025'

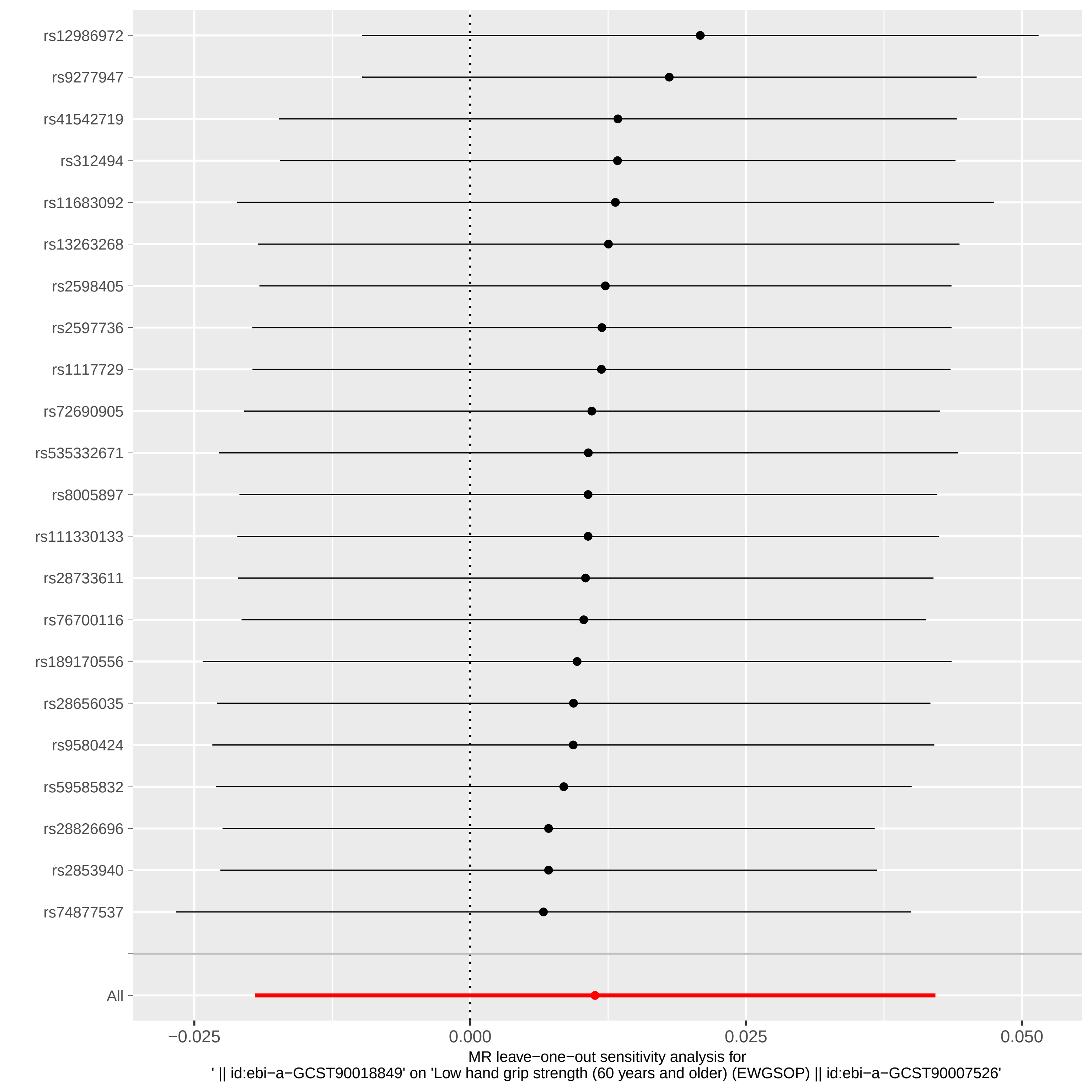

rs12986972
rs9277947
rs41542719
rs312494
rs11683092
rs13263268
rs2598405
rs2597736
rs1117729
rs72690905
rs535332671
rs8005897
rs111330133
rs28733611
rs76700116
rs189170556
rs28656035
rs9580424
rs59585832
rs28826696
rs2853940
rs74877537
All
−0.025
0.000
0.025
0.050
MR leave−one−out sensitivity analysis for
' || id:ebi−a−GCST90018849' on 'Low hand grip strength (60 years and older) (EWGSOP) || id:ebi−a−GCST90007526'

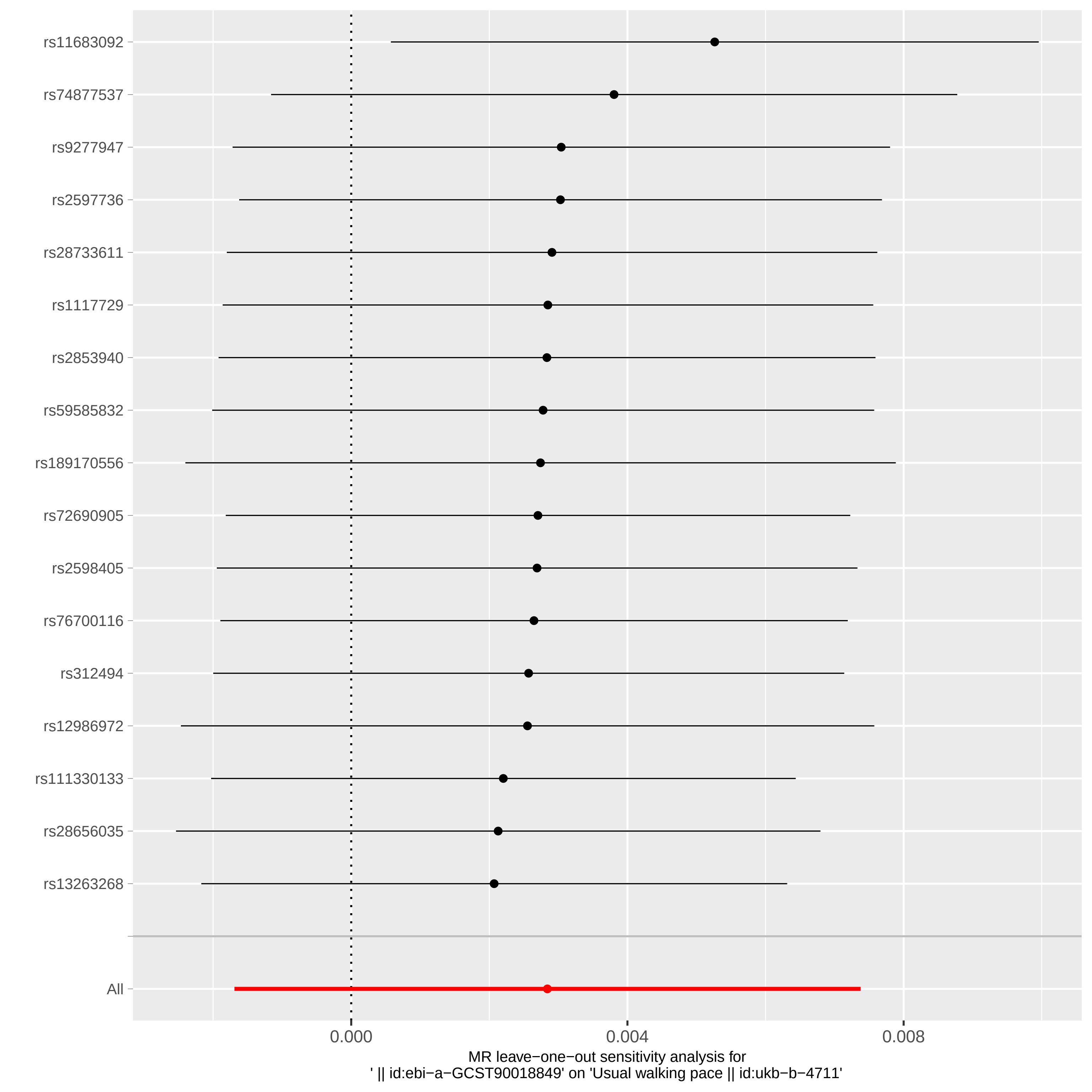

rs11683092
rs74877537
rs9277947
rs2597736
rs28733611
rs1117729
rs2853940
rs59585832
rs189170556
rs72690905
rs2598405
rs76700116
rs312494
rs12986972
rs111330133
rs28656035
rs13263268
All
0.000
0.004
0.008
MR leave−one−out sensitivity analysis for
' || id:ebi−a−GCST90018849' on 'Usual walking pace || id:ukb−b−4711'

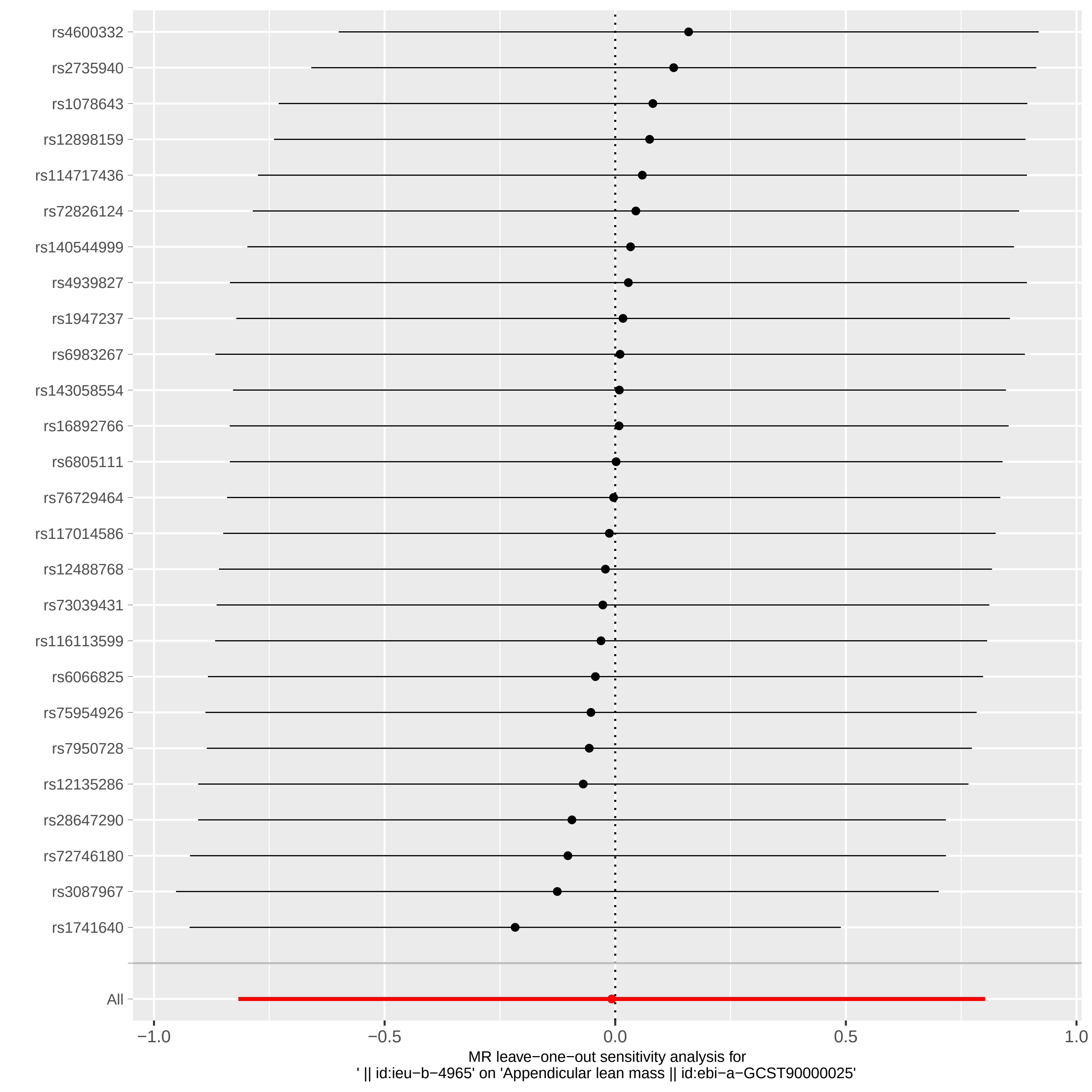

rs4600332
rs2735940
rs1078643
rs12898159
rs114717436
rs72826124
rs140544999
rs4939827
rs1947237
rs6983267
rs143058554
rs16892766
rs6805111
rs76729464
rs117014586
rs12488768
rs73039431
rs116113599
rs6066825
rs75954926
rs7950728
rs12135286
rs28647290
rs72746180
rs3087967
rs1741640
All
−1.0
−0.5
0.0
0.5
1.0
MR leave−one−out sensitivity analysis for
' || id:ieu−b−4965' on 'Appendicular lean mass || id:ebi−a−GCST90000025'

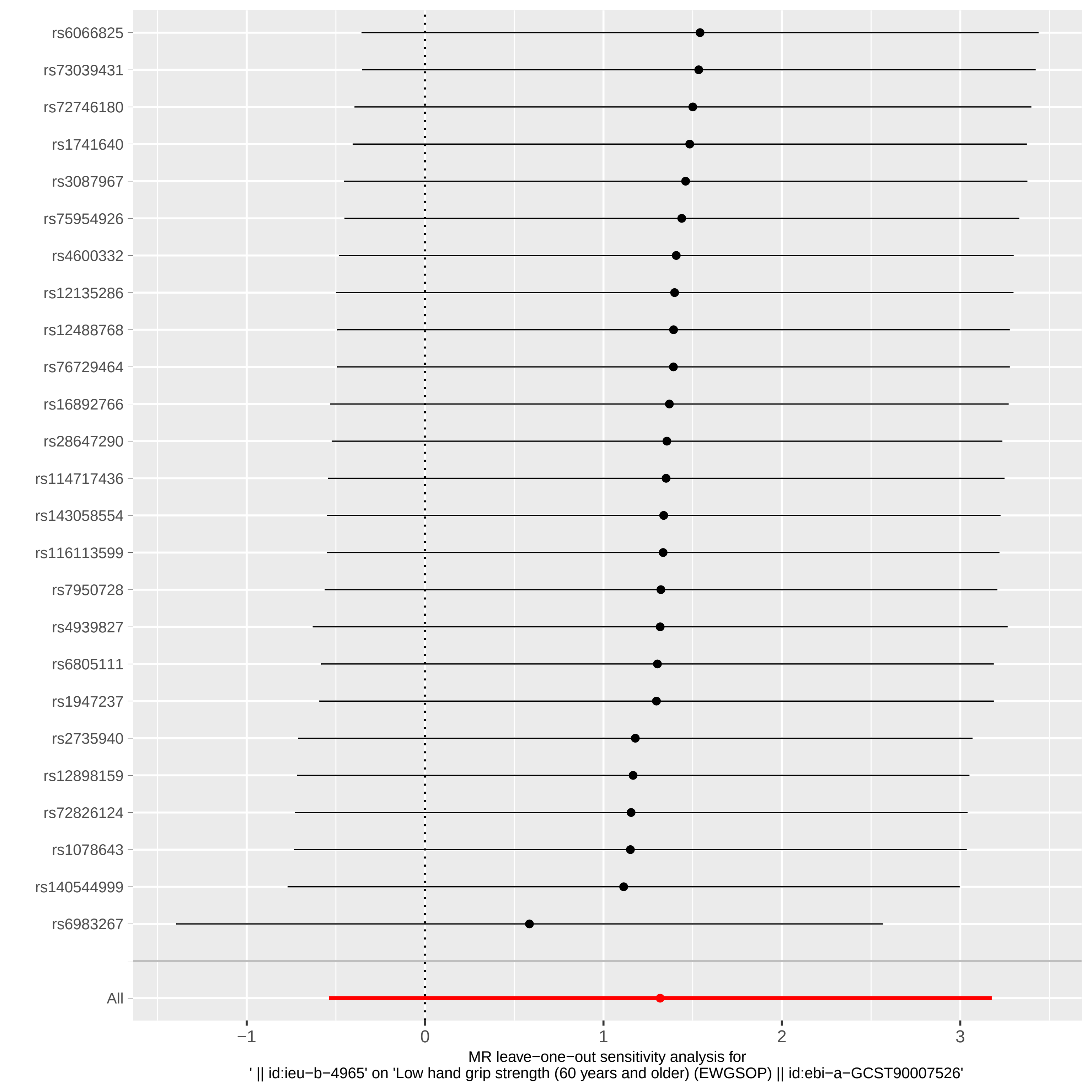

rs6066825
rs73039431
rs72746180
rs1741640
rs3087967
rs75954926
rs4600332
rs12135286
rs12488768
rs76729464
rs16892766
rs28647290
rs114717436
rs143058554
rs116113599
rs7950728
rs4939827
rs6805111
rs1947237
rs2735940
rs12898159
rs72826124
rs1078643
rs140544999
rs6983267
All
−1
0
1
2
3
MR leave−one−out sensitivity analysis for
' || id:ieu−b−4965' on 'Low hand grip strength (60 years and older) (EWGSOP) || id:ebi−a−GCST90007526'

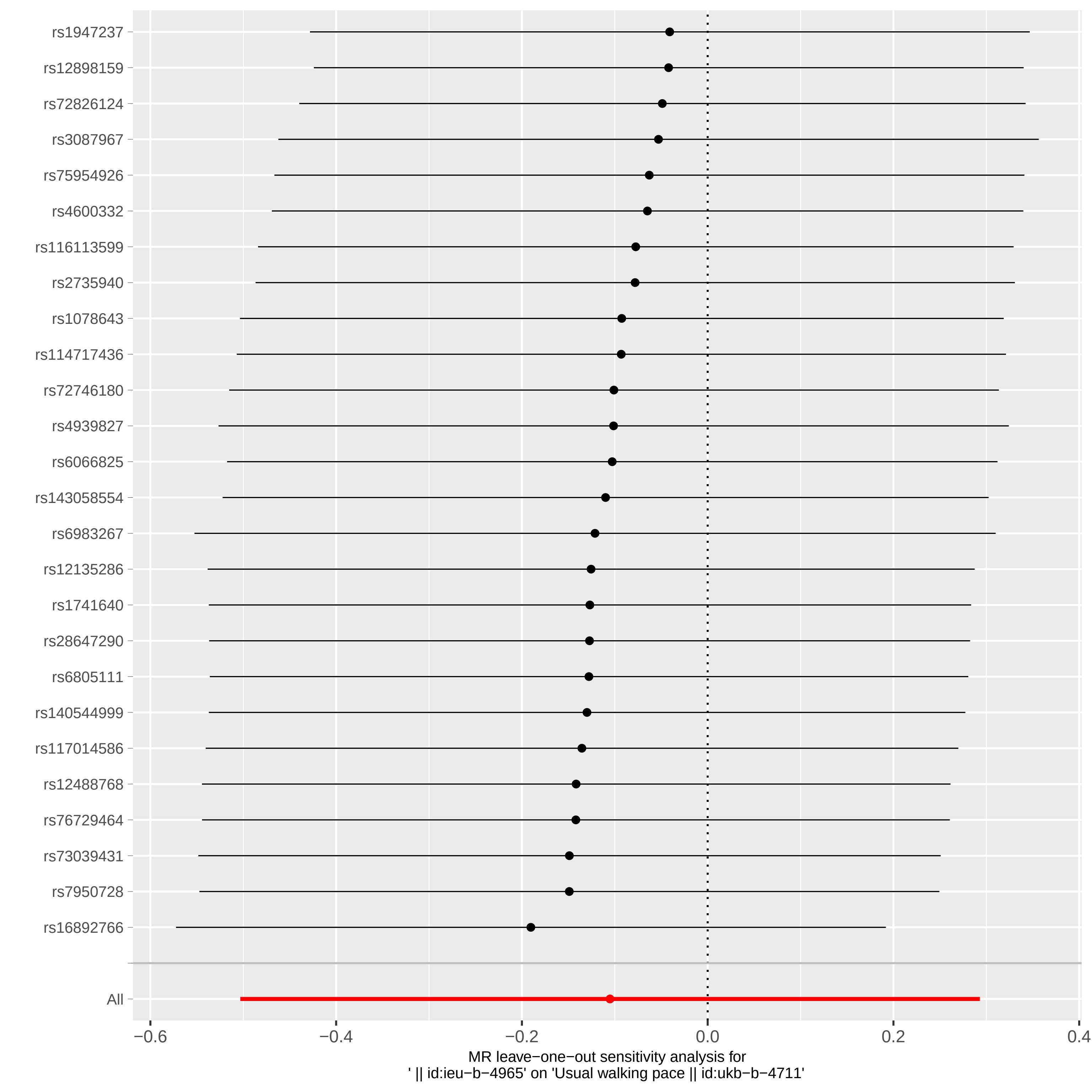
rs1947237
rs12898159
rs72826124
rs3087967
rs75954926
rs4600332
rs116113599
rs2735940
rs1078643
rs114717436
rs72746180
rs4939827
rs6066825
rs143058554
rs6983267
rs12135286
rs1741640
rs28647290
rs6805111
rs140544999
rs117014586
rs12488768
rs76729464
rs73039431
rs7950728
rs16892766
All
−0.6
−0.4
−0.2
0.0
0.2
0.4
MR leave−one−out sensitivity analysis for
' || id:ieu−b−4965' on 'Usual walking pace || id:ukb−b−4711'

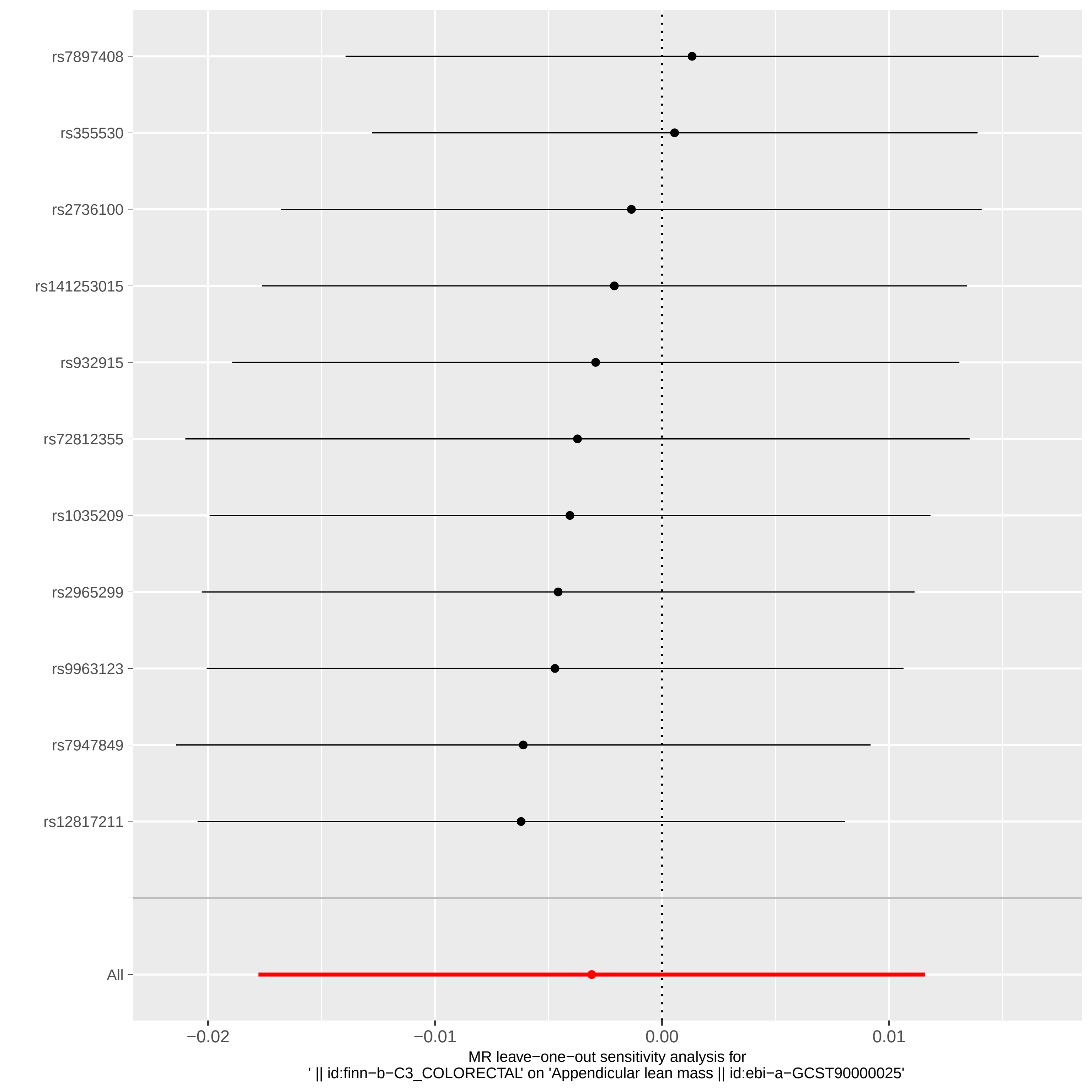
rs7897408
rs355530
rs2736100
rs141253015
rs932915
rs72812355
rs1035209
rs2965299
rs9963123
rs7947849
rs12817211
All
−0.02
−0.01
0.00
0.01
MR leave−one−out sensitivity analysis for
' || id:finn−b−C3_COLORECTAL' on 'Appendicular lean mass || id:ebi−a−GCST90000025'

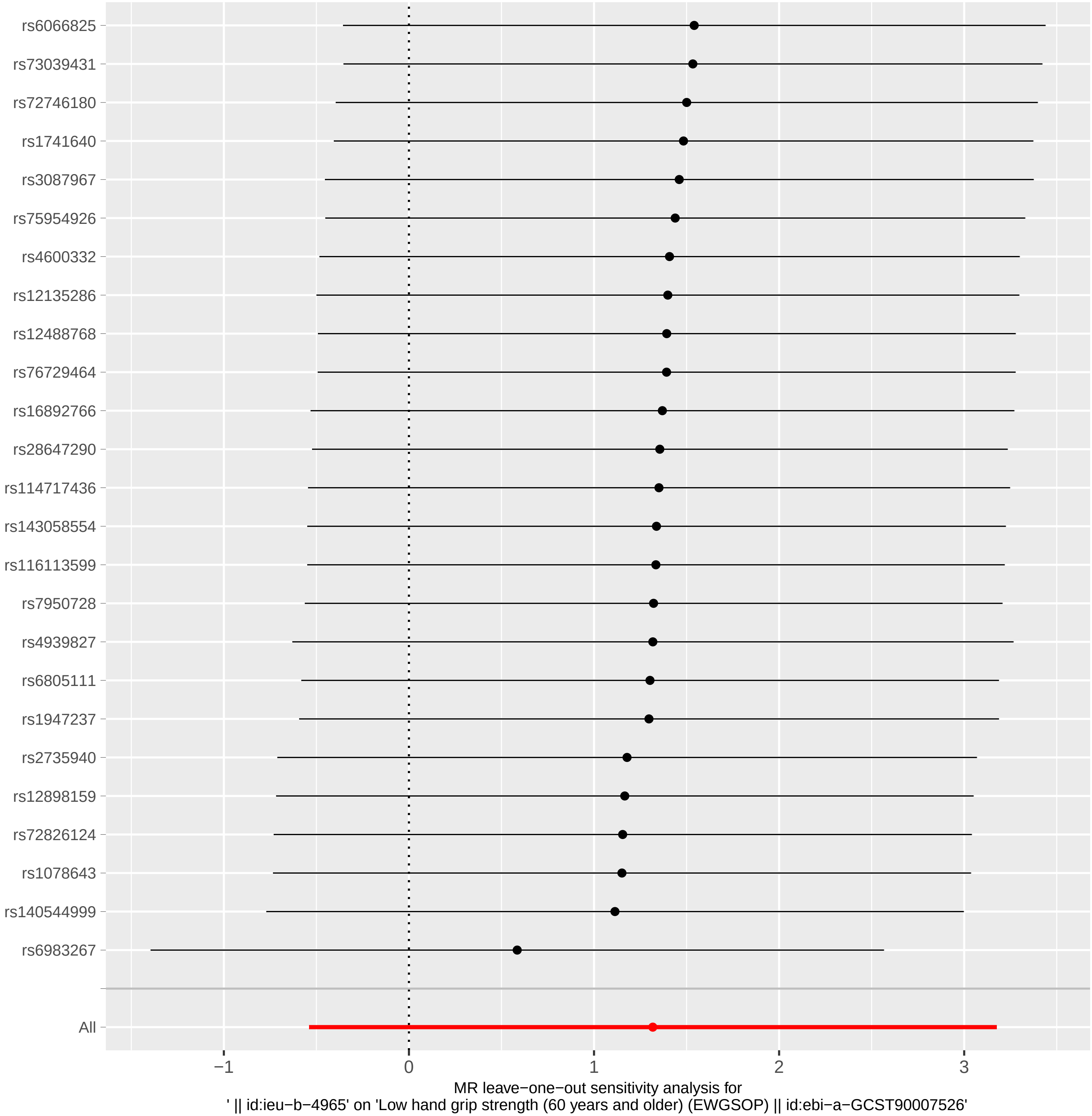
rs6066825
rs73039431
rs72746180
rs1741640
rs3087967
rs75954926
rs4600332
rs12135286
rs12488768
rs76729464
rs16892766
rs28647290
rs114717436
rs143058554
rs116113599
rs7950728
rs4939827
rs6805111
rs1947237
rs2735940
rs12898159
rs72826124
rs1078643
rs140544999
rs6983267
All
−1
0
1
2
3
MR leave−one−out sensitivity analysis for
' || id:ieu−b−4965' on 'Low hand grip strength (60 years and older) (EWGSOP) || id:ebi−a−GCST90007526'

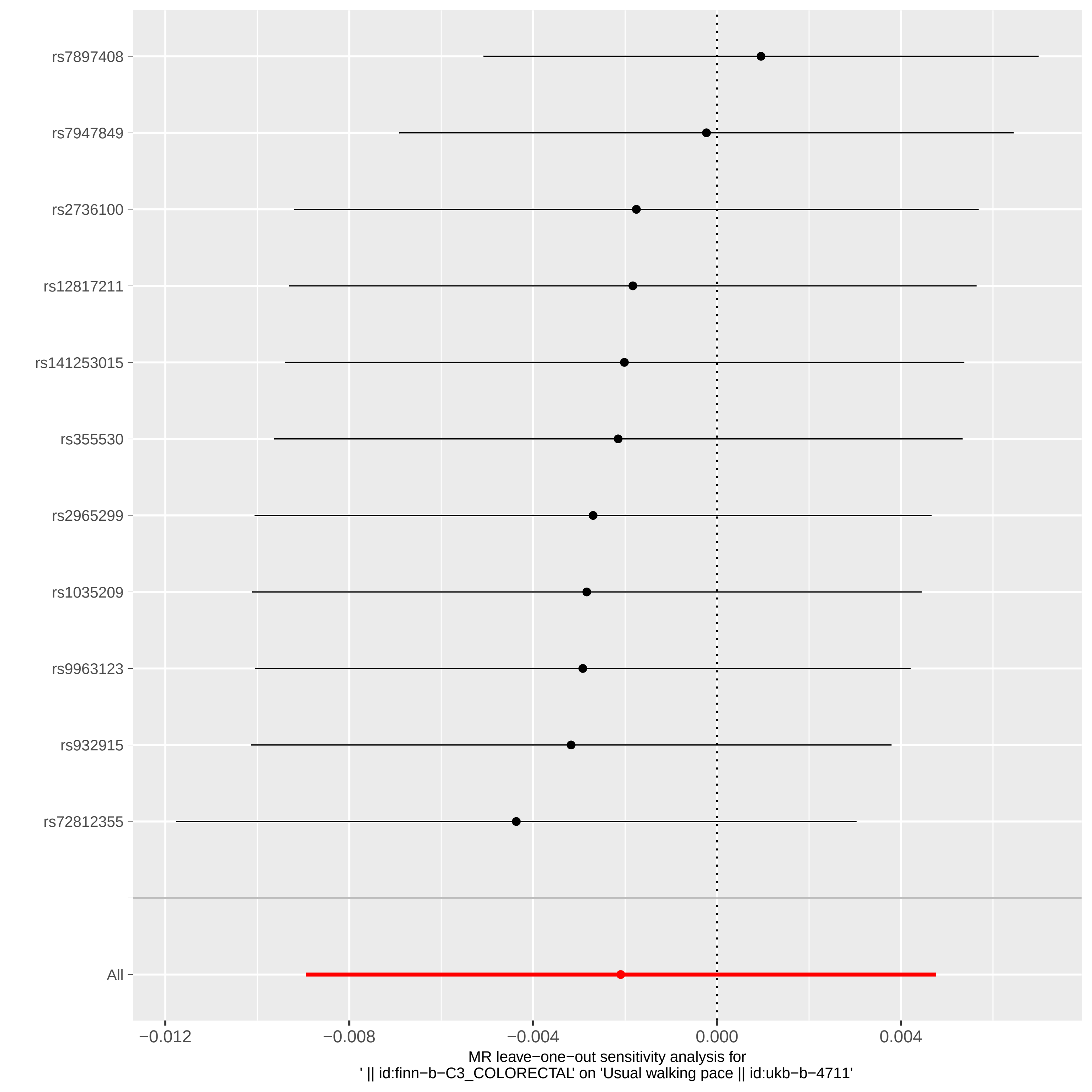

rs7897408
rs7947849
rs2736100
rs12817211
rs141253015
rs355530
rs2965299
rs1035209
rs9963123
rs932915
rs72812355
All
−0.012
−0.008
−0.004
0.000
0.004
MR leave−one−out sensitivity analysis for
' || id:finn−b−C3_COLORECTAL' on 'Usual walking pace || id:ukb−b−4711'

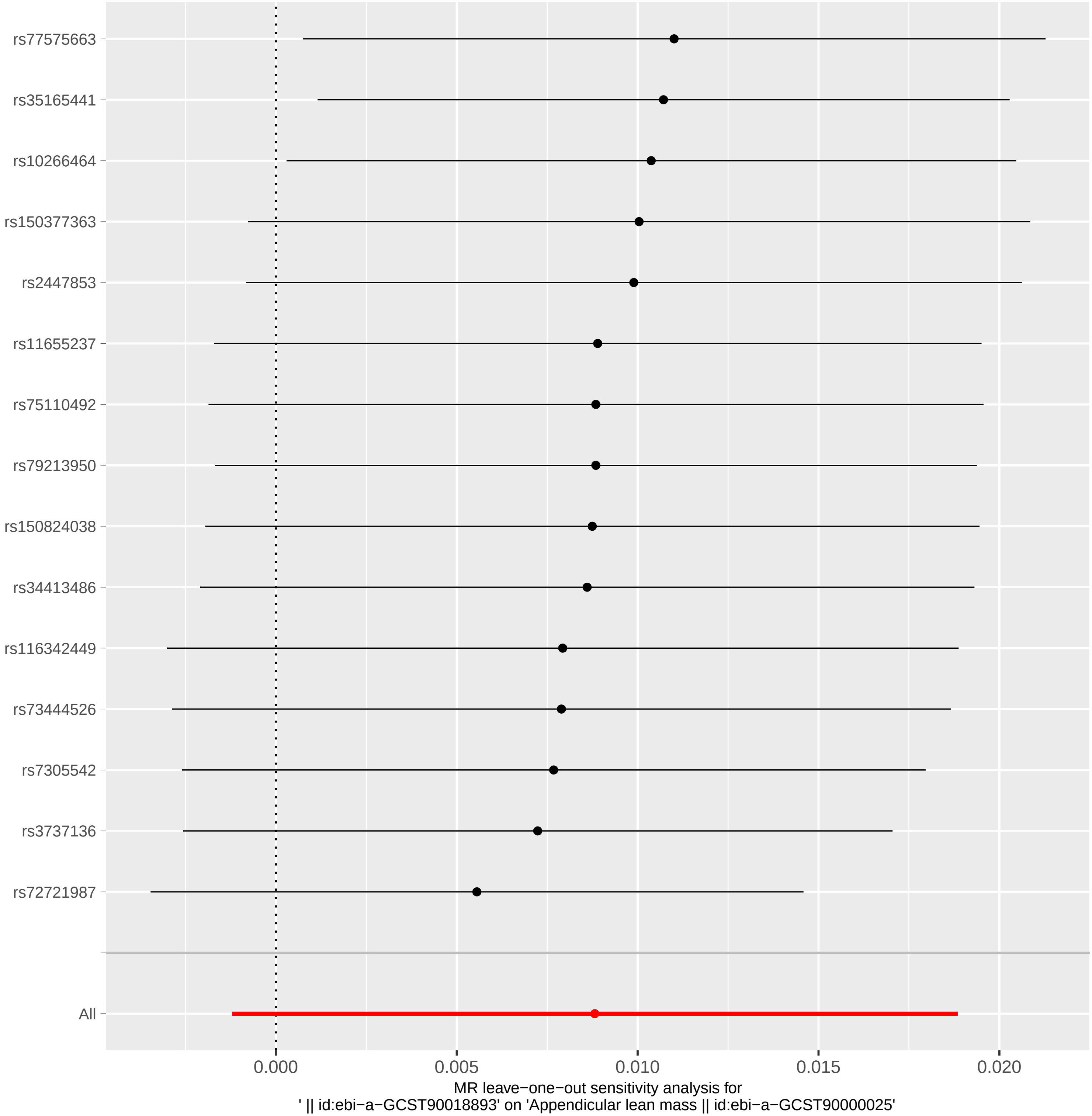

rs77575663
rs35165441
rs10266464
rs150377363
rs2447853
rs11655237
rs75110492
rs79213950
rs150824038
rs34413486
rs116342449
rs73444526
rs7305542
rs3737136
rs72721987
All
0.000
0.005
0.010
0.015
0.020
MR leave−one−out sensitivity analysis for
' || id:ebi−a−GCST90018893' on 'Appendicular lean mass || id:ebi−a−GCST90000025'

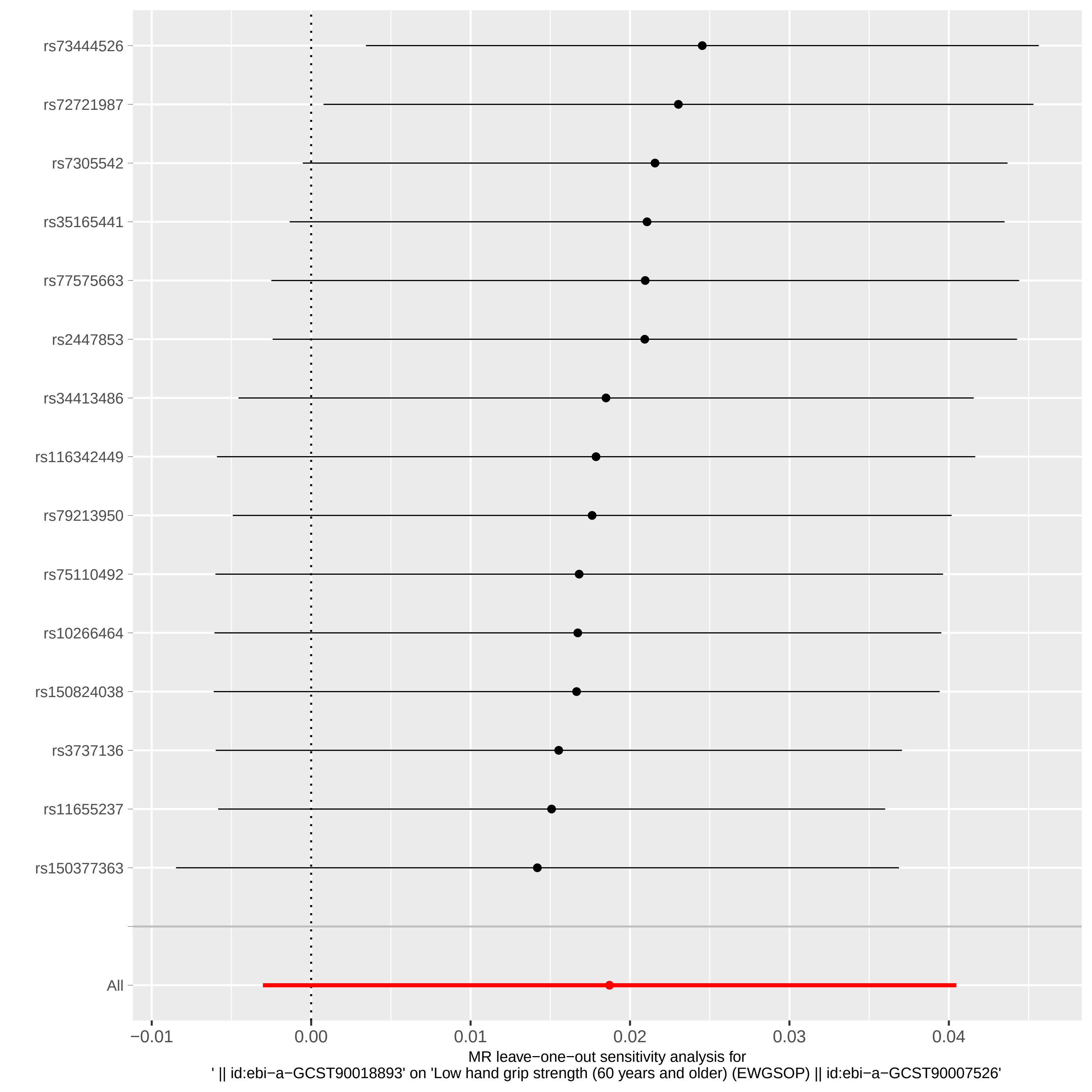

rs73444526
rs72721987
rs7305542
rs35165441
rs77575663
rs2447853
rs34413486
rs116342449
rs79213950
rs75110492
rs10266464
rs150824038
rs3737136
rs11655237
rs150377363
All
−0.01
0.00
0.01
0.02
0.03
0.04
MR leave−one−out sensitivity analysis for
' || id:ebi−a−GCST90018893' on 'Low hand grip strength (60 years and older) (EWGSOP) || id:ebi−a−GCST90007526'

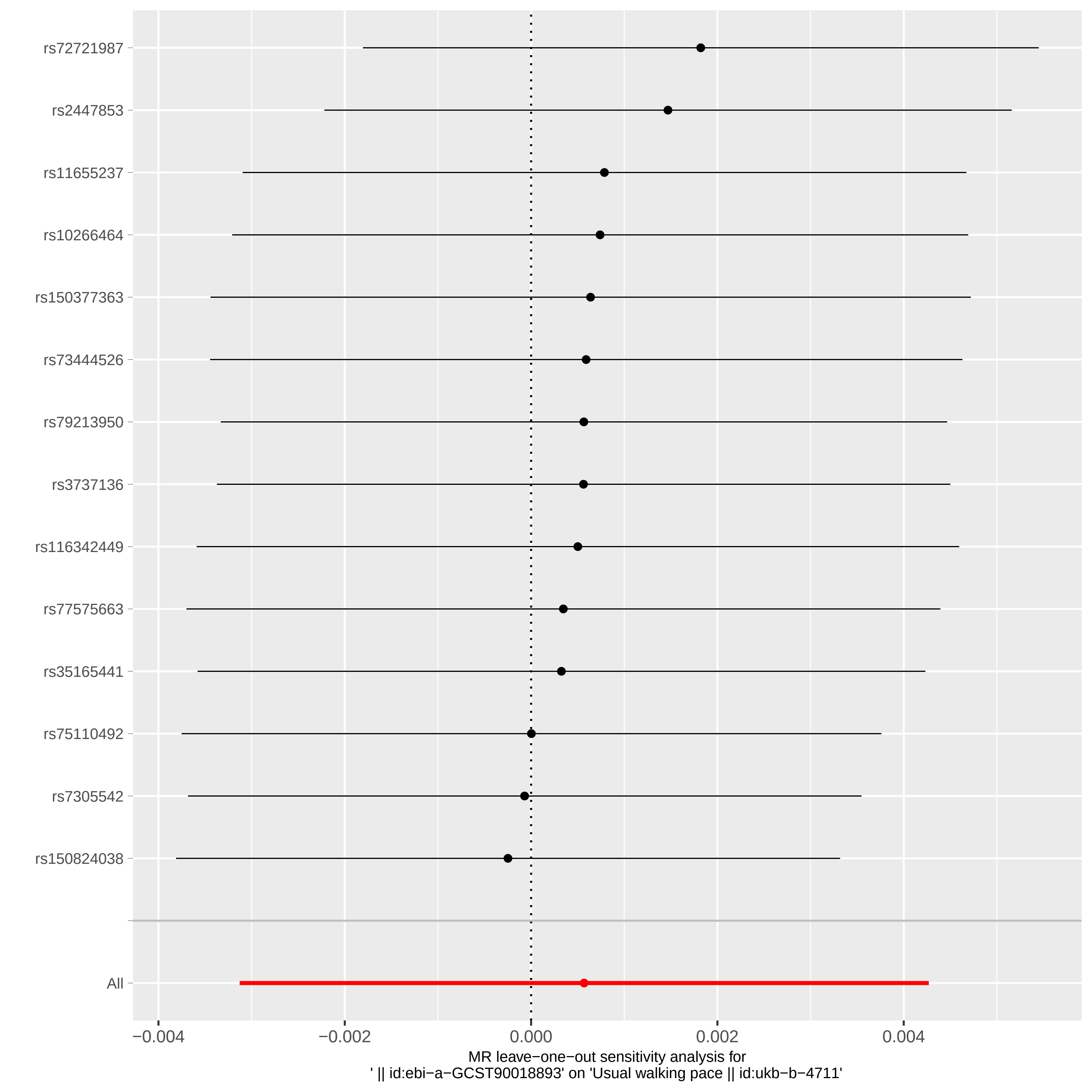

rs72721987
rs2447853
rs11655237
rs10266464
rs150377363
rs73444526
rs79213950
rs3737136
rs116342449
rs77575663
rs35165441
rs75110492
rs7305542
rs150824038
All
−0.004
−0.002
0.000
0.002
0.004
MR leave−one−out sensitivity analysis for
' || id:ebi−a−GCST90018893' on 'Usual walking pace || id:ukb−b−4711'

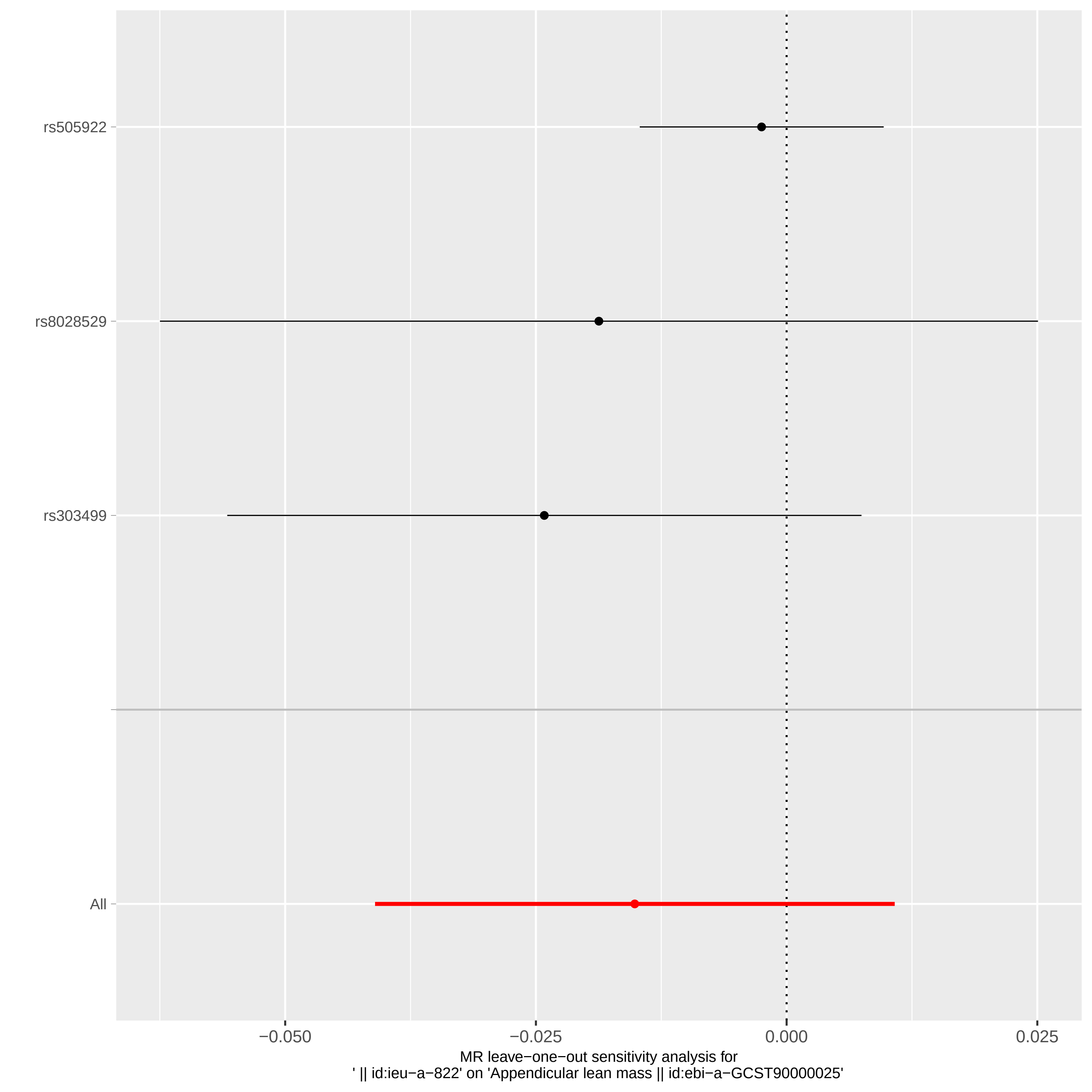
rs505922
rs8028529
rs303499
All
−0.050
−0.025
0.000
0.025
MR leave−one−out sensitivity analysis for
' || id:ieu−a−822' on 'Appendicular lean mass || id:ebi−a−GCST90000025'

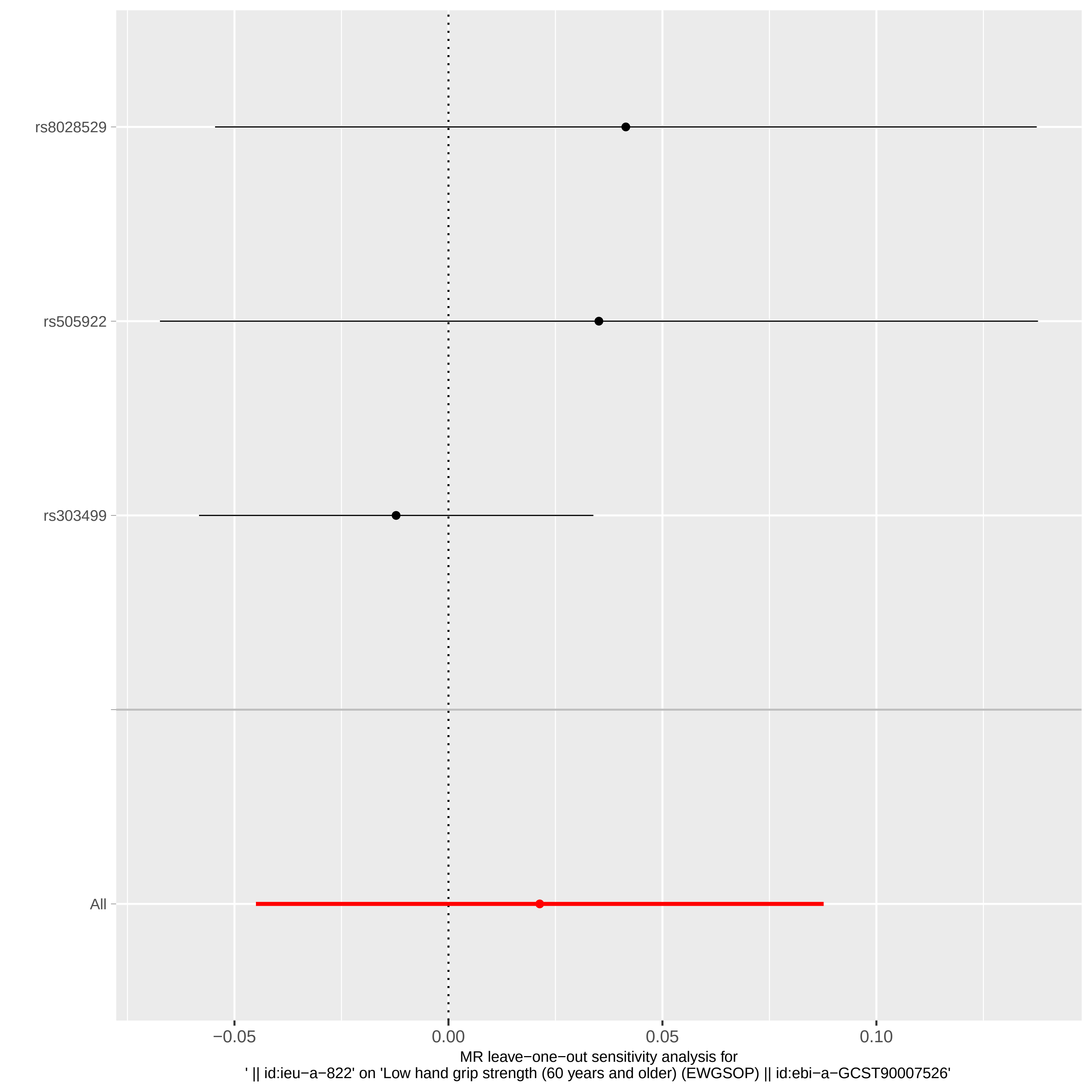

rs8028529
rs505922
rs303499
All
−0.05
0.00
0.05
0.10
MR leave−one−out sensitivity analysis for
' || id:ieu−a−822' on 'Low hand grip strength (60 years and older) (EWGSOP) || id:ebi−a−GCST90007526'

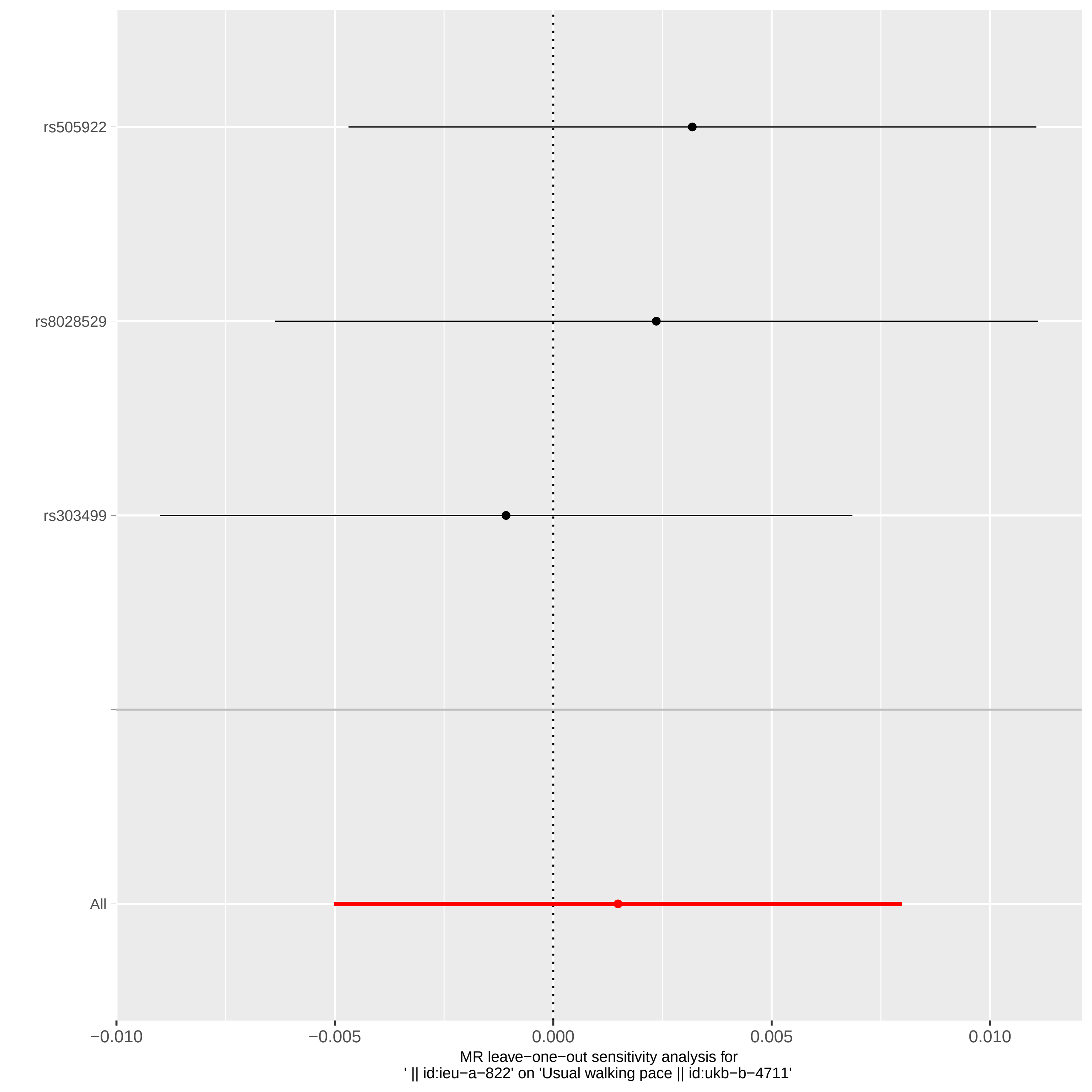
rs505922
rs8028529
rs303499
All
−0.010
−0.005
0.000
0.005
0.010
MR leave−one−out sensitivity analysis for
' || id:ieu−a−822' on 'Usual walking pace || id:ukb−b−4711'

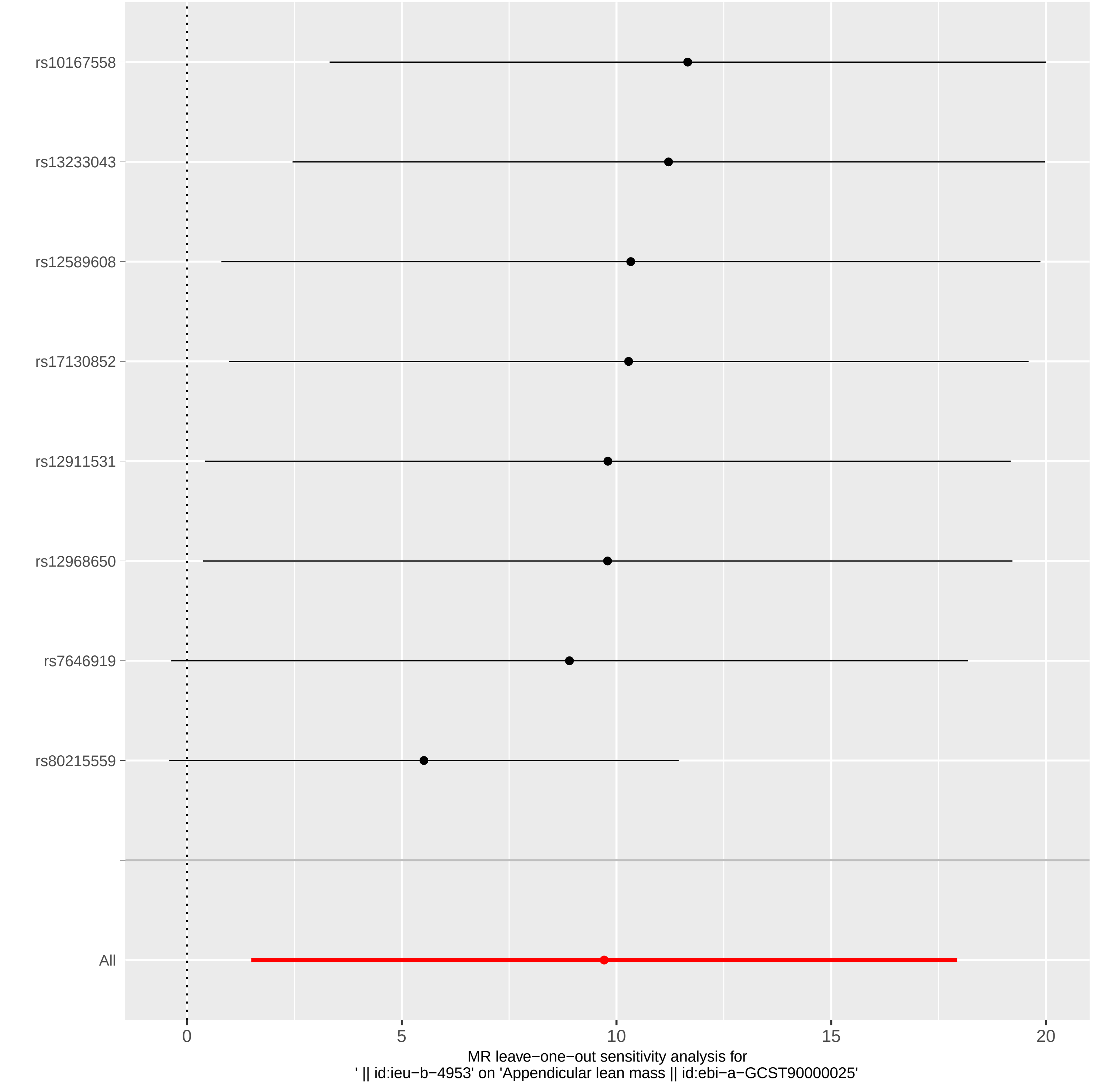
rs10167558
rs13233043
rs12589608
rs17130852
rs12911531
rs12968650
rs7646919
rs80215559
All
0
5
10
15
20
MR leave−one−out sensitivity analysis for
' || id:ieu−b−4953' on 'Appendicular lean mass || id:ebi−a−GCST90000025'

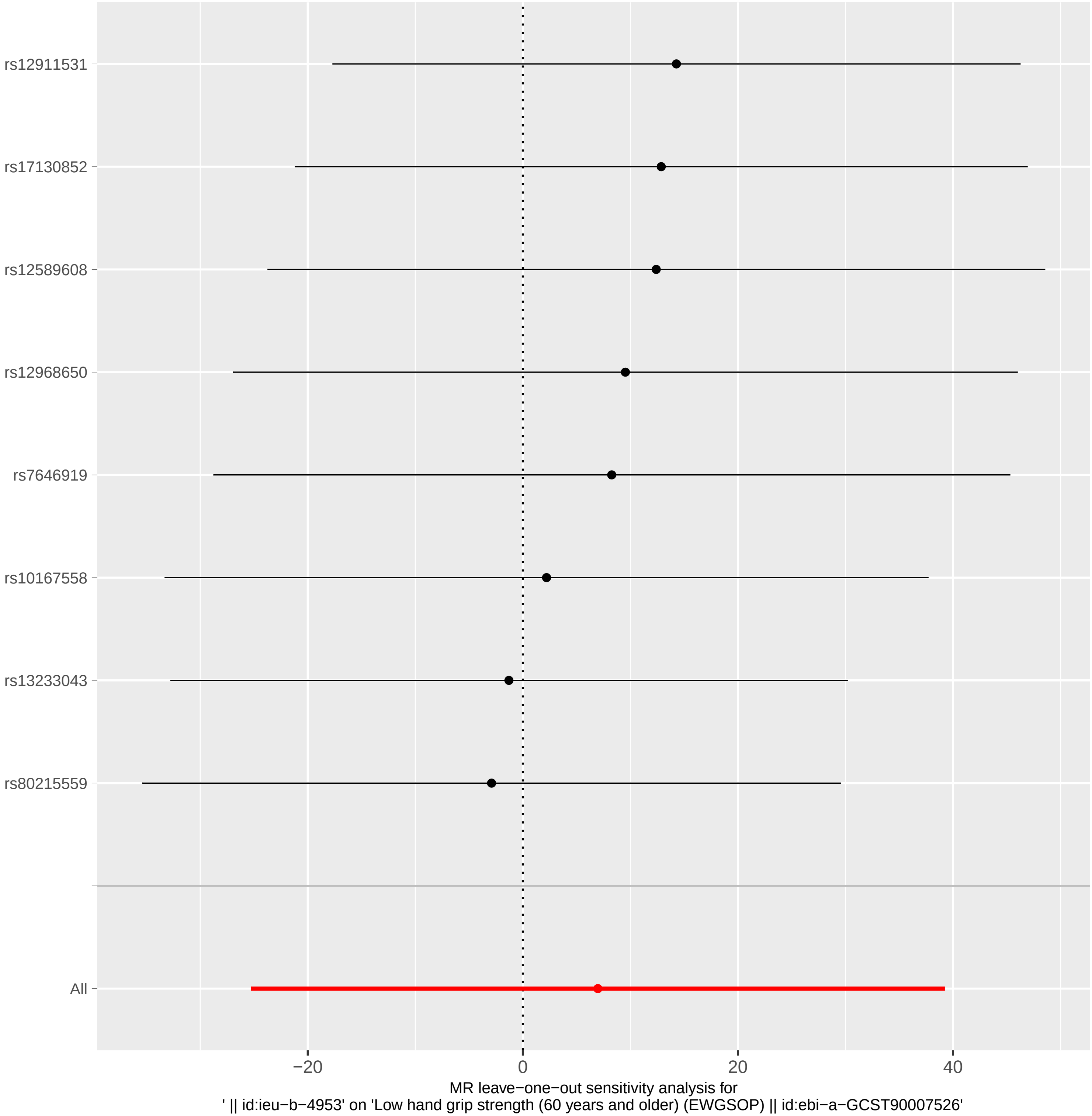

rs12911531
rs17130852
rs12589608
rs12968650
rs7646919
rs10167558
rs13233043
rs80215559
All
−20
0
20
40
MR leave−one−out sensitivity analysis for
' || id:ieu−b−4953' on 'Low hand grip strength (60 years and older) (EWGSOP) || id:ebi−a−GCST90007526'

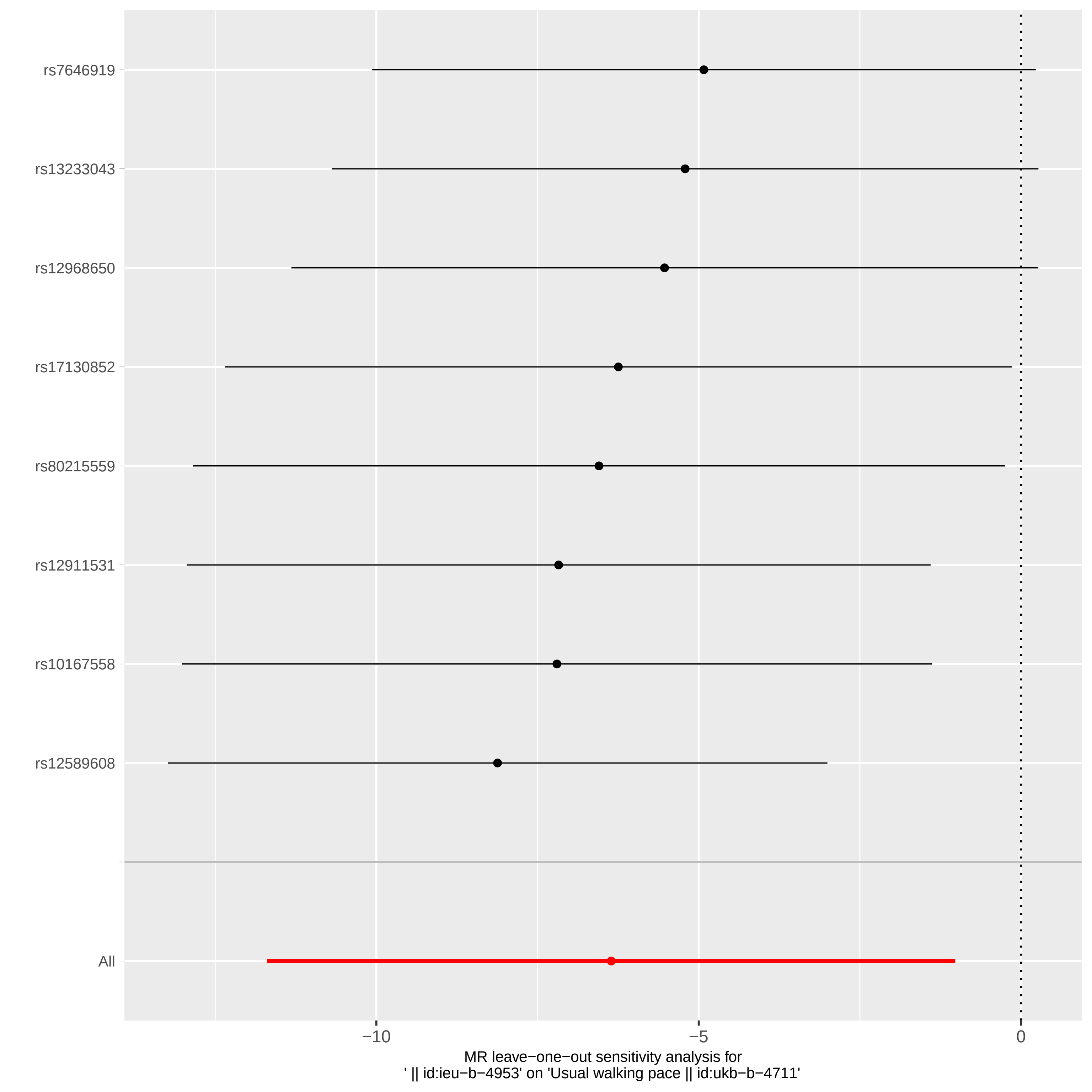
rs7646919
rs13233043
rs12968650
rs17130852
rs80215559
rs12911531
rs10167558
rs12589608
All
−10
−5
0
MR leave−one−out sensitivity analysis for
' || id:ieu−b−4953' on 'Usual walking pace || id:ukb−b−4711'

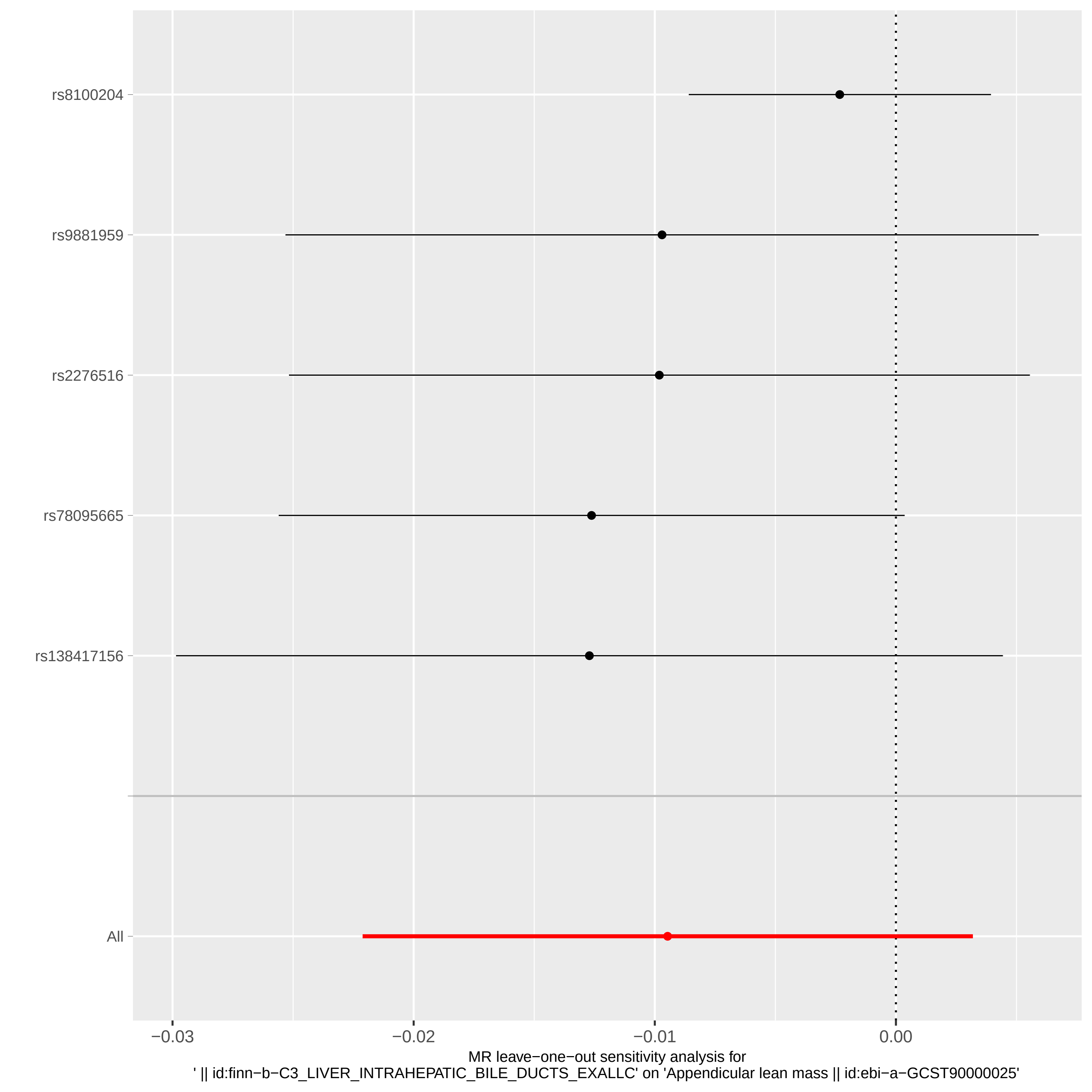

rs8100204
rs9881959
rs2276516
rs78095665
rs138417156
All
−0.03
−0.02
−0.01
0.00
MR leave−one−out sensitivity analysis for
' || id:finn−b−C3_LIVER_INTRAHEPATIC_BILE_DUCTS_EXALLC' on 'Appendicular lean mass || id:ebi−a−GCST90000025'

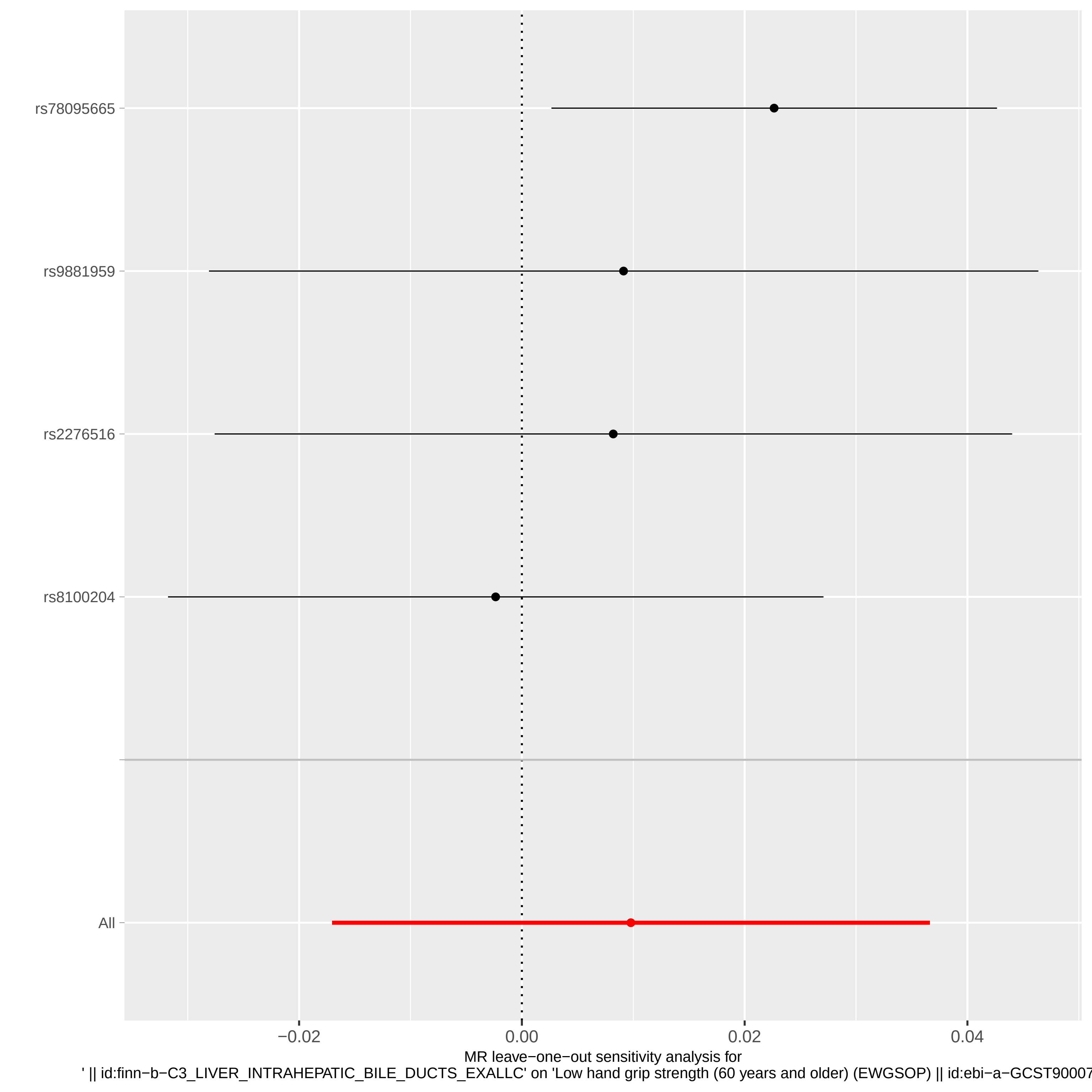
rs78095665
rs9881959
rs2276516
rs8100204
All
−0.02
0.00
0.02
0.04
MR leave−one−out sensitivity analysis for
' || id:finn−b−C3_LIVER_INTRAHEPATIC_BILE_DUCTS_EXALLC' on 'Low hand grip strength (60 years and older) (EWGSOP) || id:ebi−a−GCST90007

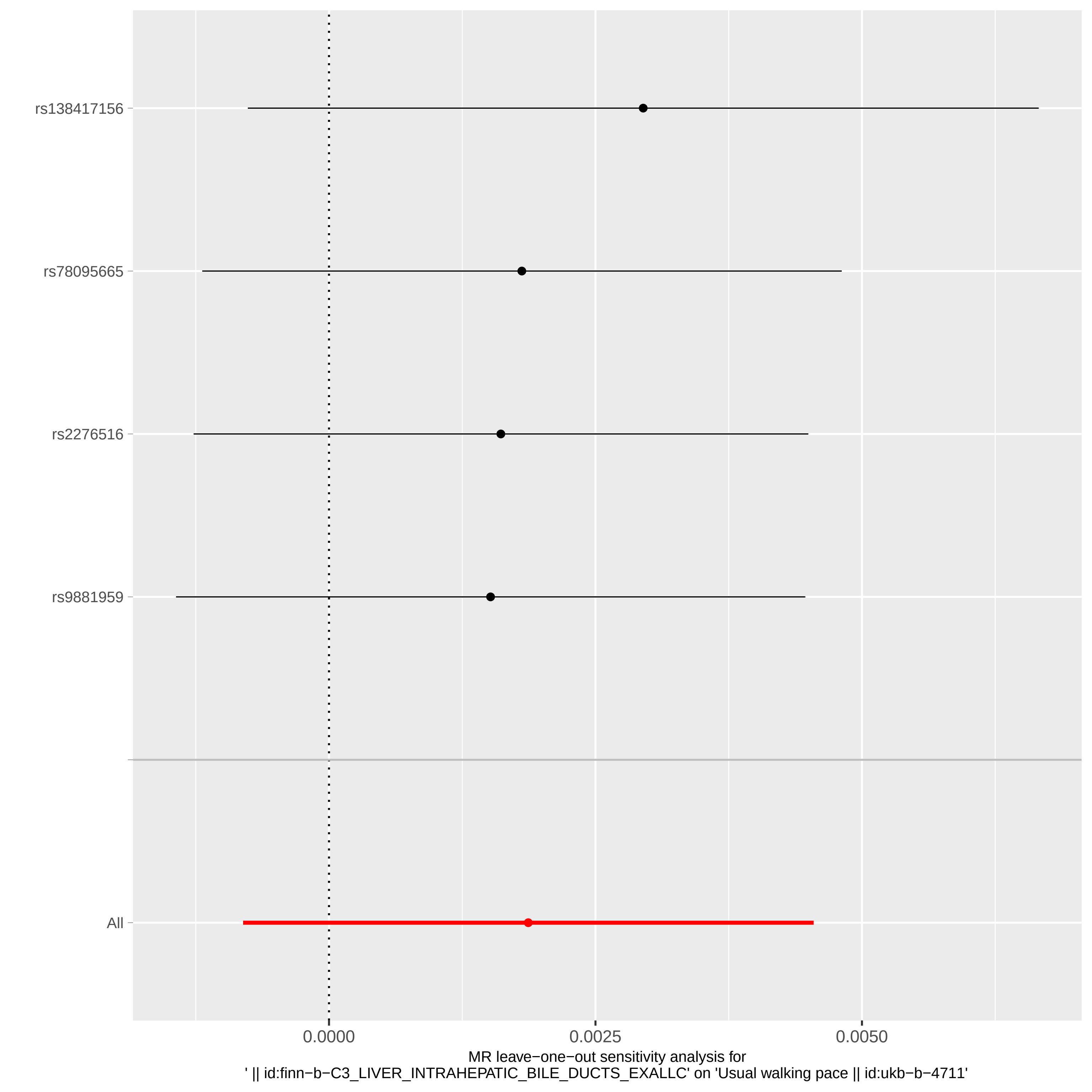
rs138417156
rs78095665
rs2276516
rs9881959
All
0.0000
0.0025
0.0050
MR leave−one−out sensitivity analysis for
' || id:finn−b−C3_LIVER_INTRAHEPATIC_BILE_DUCTS_EXALLC' on 'Usual walking pace || id:ukb−b−4711'

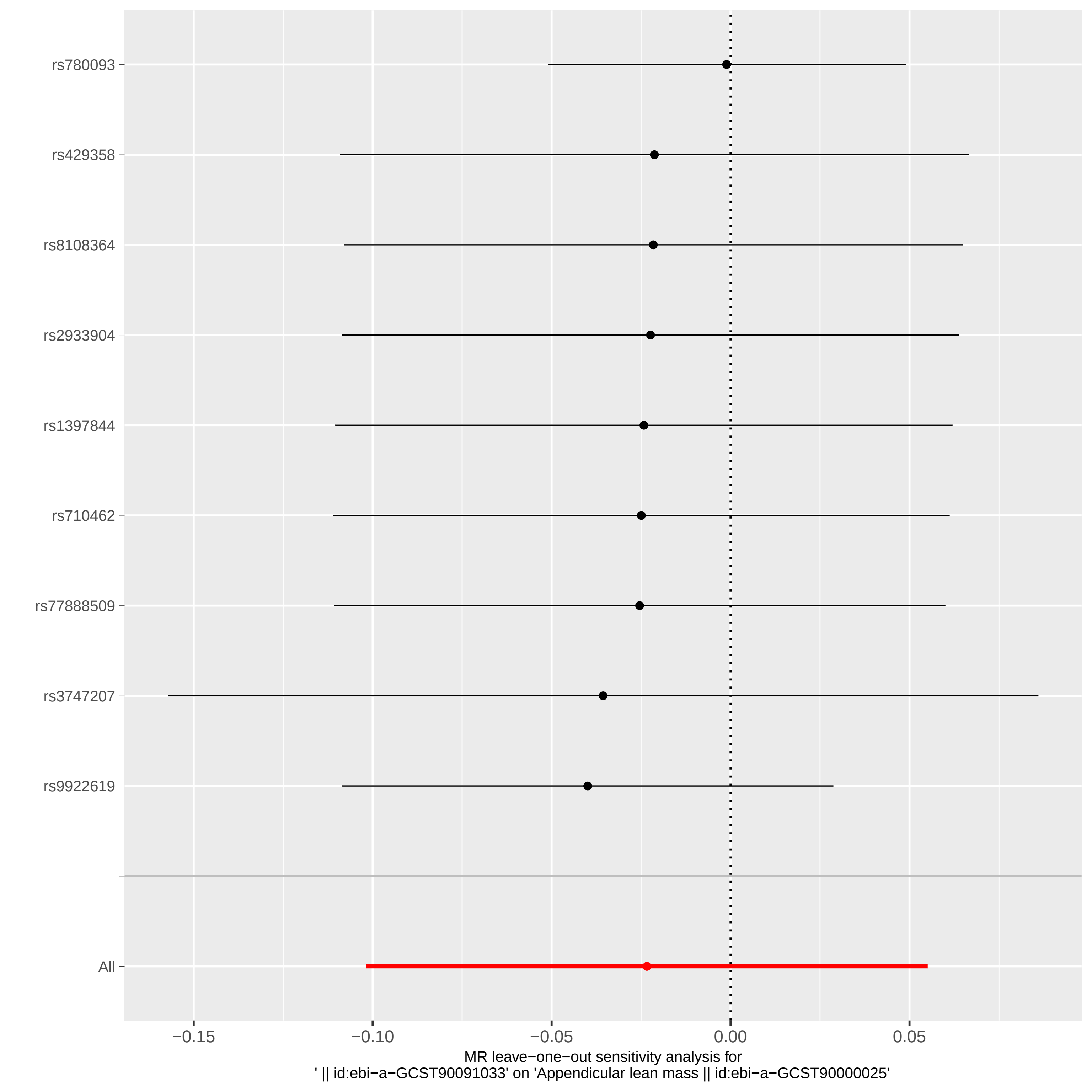
rs780093
rs429358
rs8108364
rs2933904
rs1397844
rs710462
rs77888509
rs3747207
rs9922619
All
−0.15
−0.10
−0.05
0.00
0.05
MR leave−one−out sensitivity analysis for
' || id:ebi−a−GCST90091033' on 'Appendicular lean mass || id:ebi−a−GCST90000025'

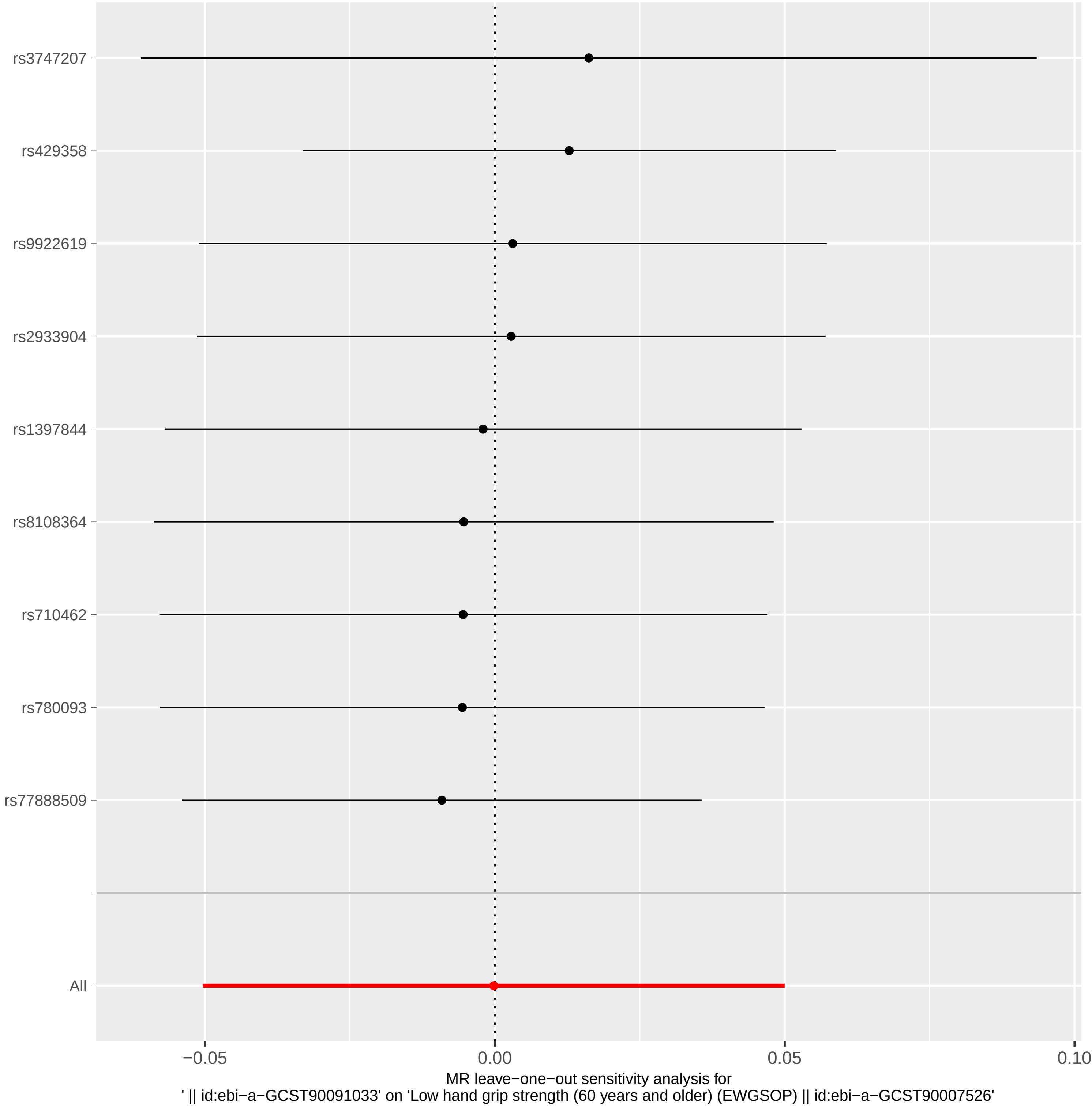

rs3747207
rs429358
rs9922619
rs2933904
rs1397844
rs8108364
rs710462
rs780093
rs77888509
All
−0.05
0.00
0.05
0.10
MR leave−one−out sensitivity analysis for
' || id:ebi−a−GCST90091033' on 'Low hand grip strength (60 years and older) (EWGSOP) || id:ebi−a−GCST90007526'

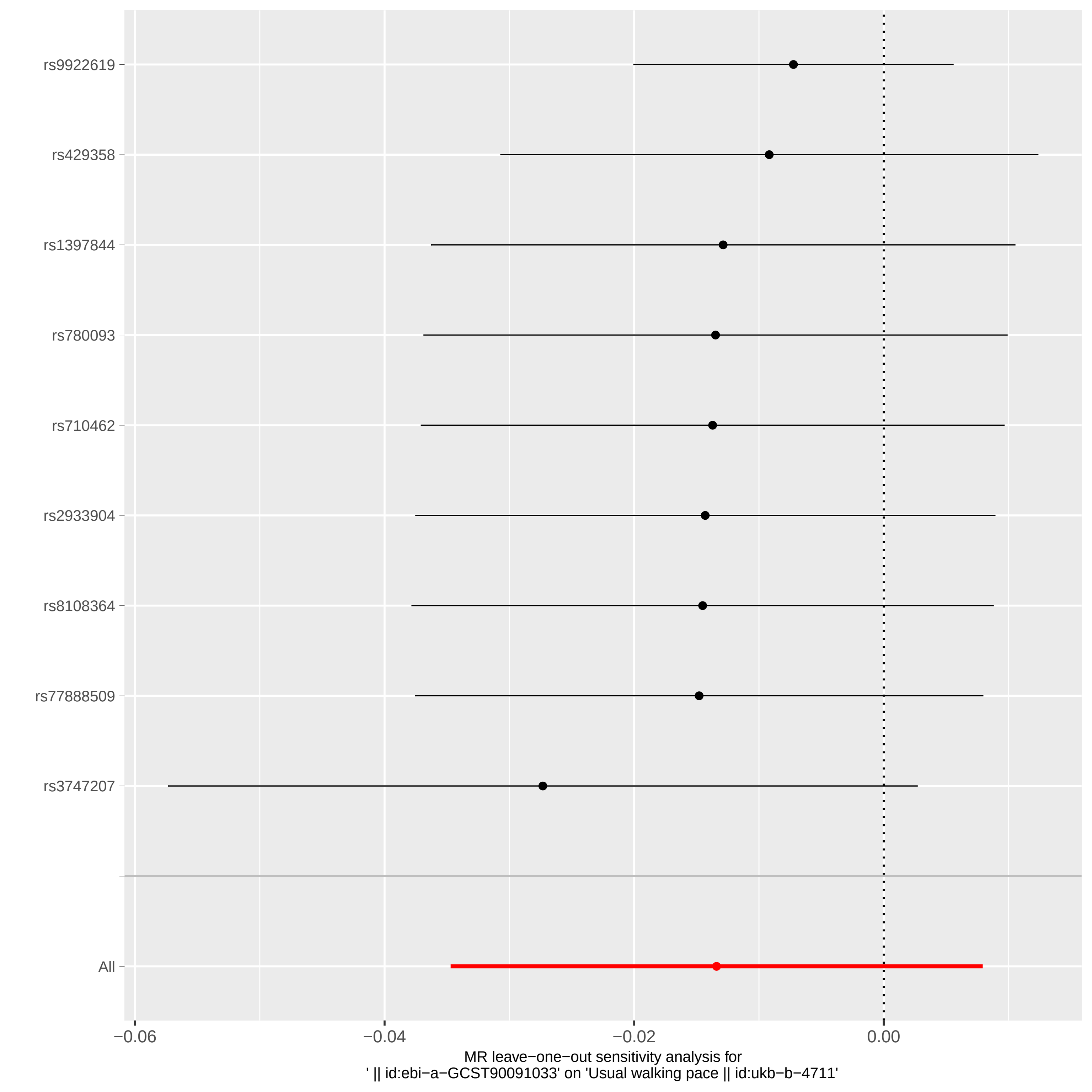

rs9922619
rs429358
rs1397844
rs780093
rs710462
rs2933904
rs8108364
rs77888509
rs3747207
All
−0.06
−0.04
−0.02
0.00
MR leave−one−out sensitivity analysis for
' || id:ebi−a−GCST90091033' on 'Usual walking pace || id:ukb−b−4711'

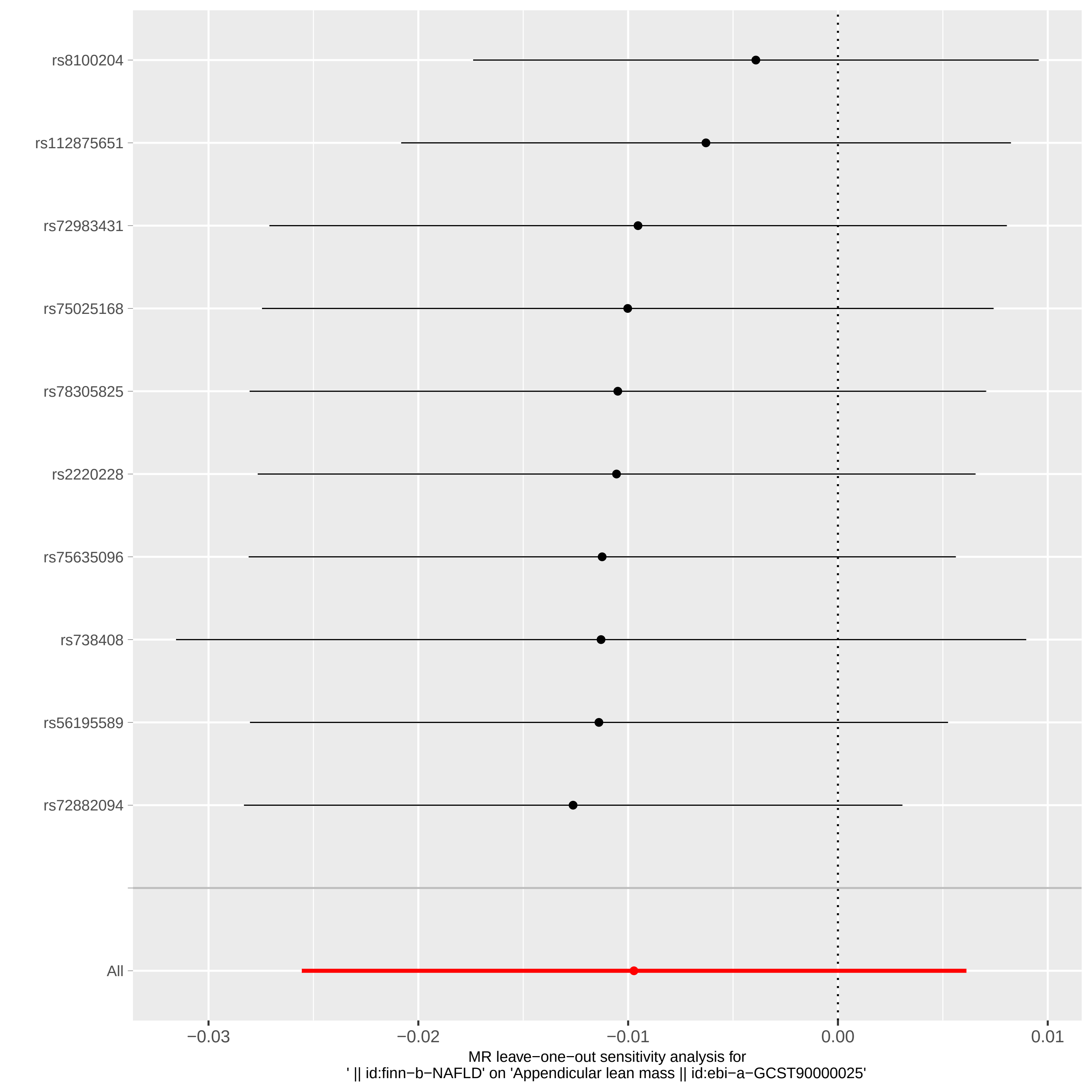

rs8100204
rs112875651
rs72983431
rs75025168
rs78305825
rs2220228
rs75635096
rs738408
rs56195589
rs72882094
All
−0.03
−0.02
−0.01
0.00
0.01
MR leave−one−out sensitivity analysis for
' || id:finn−b−NAFLD' on 'Appendicular lean mass || id:ebi−a−GCST90000025'

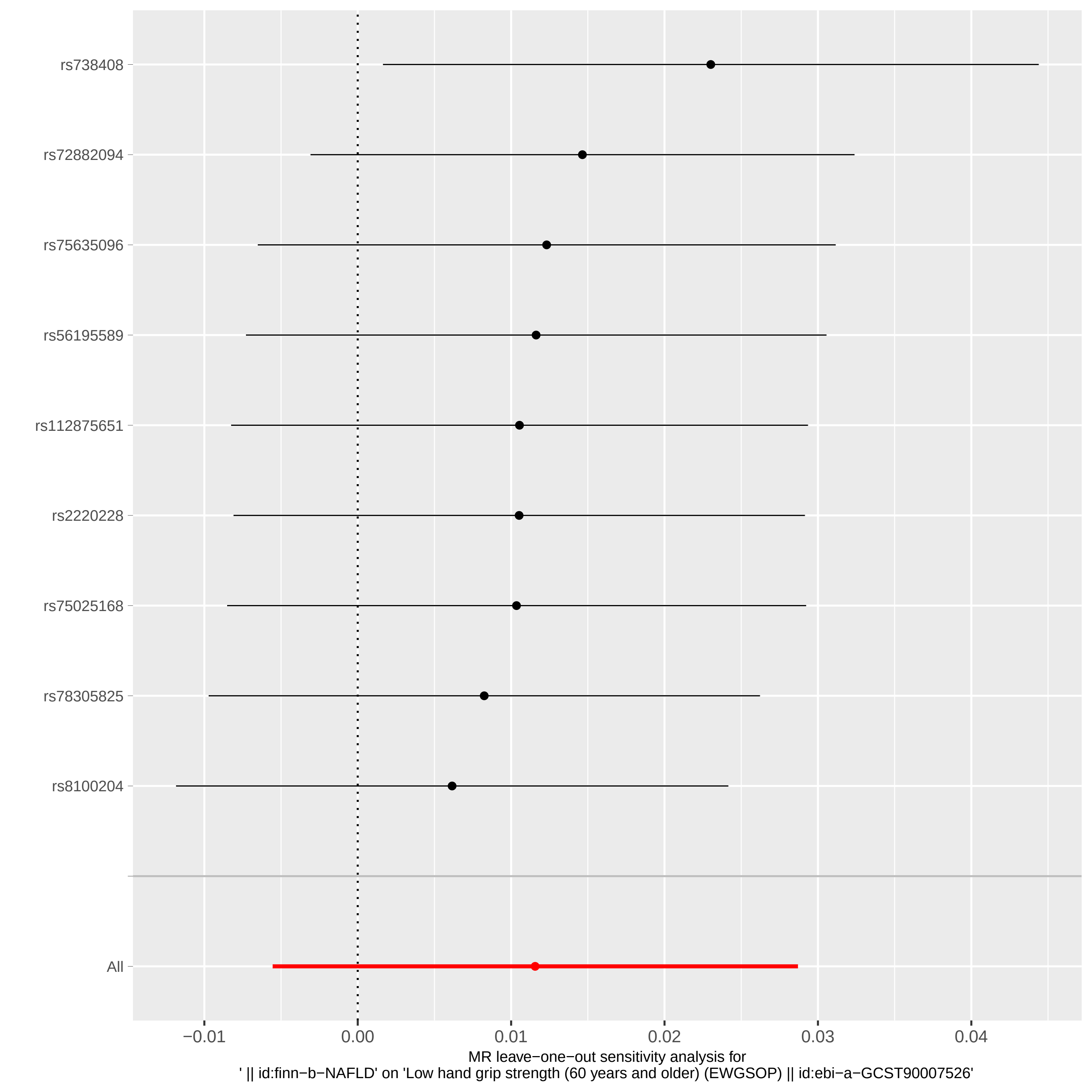
rs738408
rs72882094
rs75635096
rs56195589
rs112875651
rs2220228
rs75025168
rs78305825
rs8100204
All
−0.01
0.00
0.01
0.02
0.03
0.04
MR leave−one−out sensitivity analysis for
' || id:finn−b−NAFLD' on 'Low hand grip strength (60 years and older) (EWGSOP) || id:ebi−a−GCST90007526'

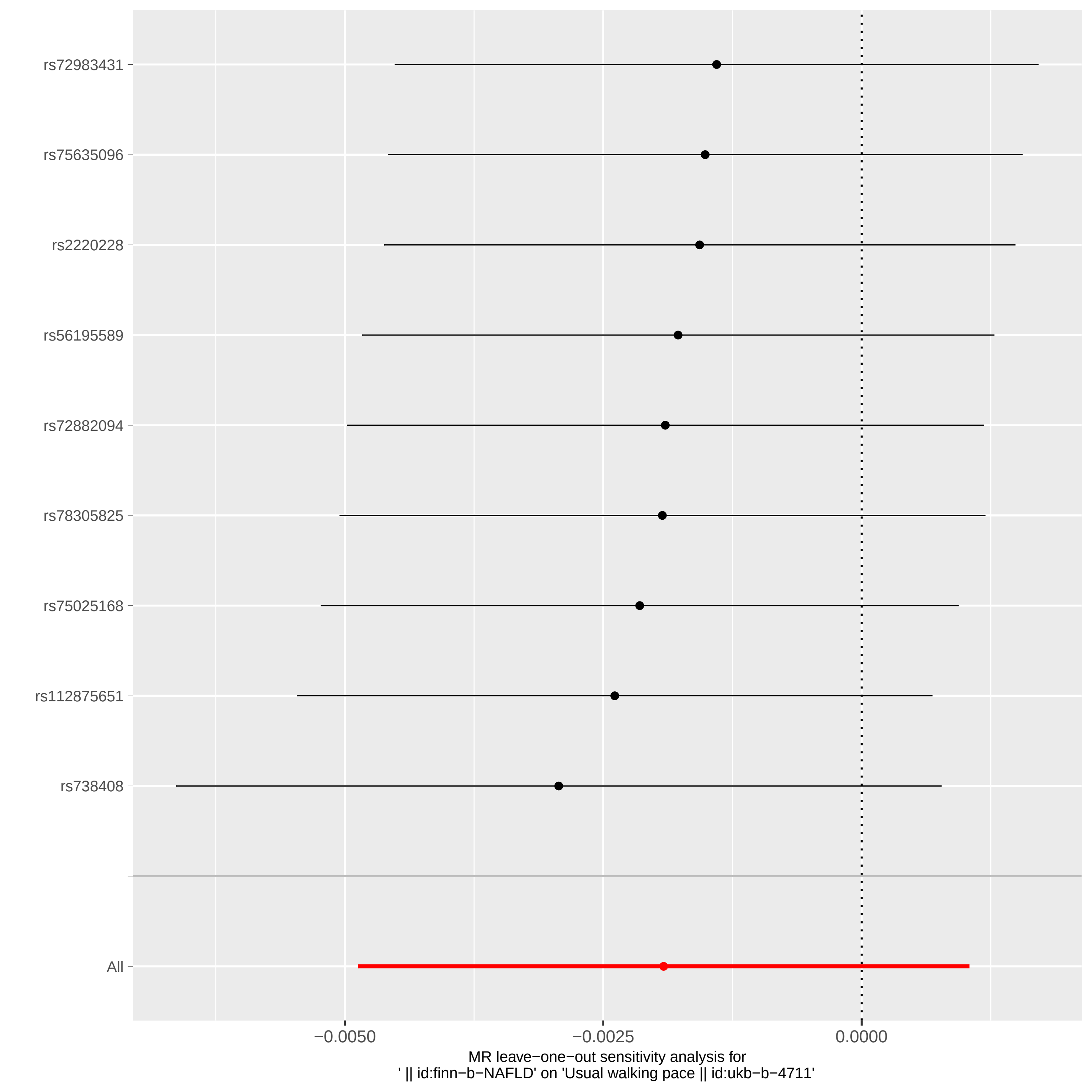
rs72983431
rs75635096
rs2220228
rs56195589
rs72882094
rs78305825
rs75025168
rs112875651
rs738408
All
−0.0050
−0.0025
0.0000
MR leave−one−out sensitivity analysis for
' || id:finn−b−NAFLD' on 'Usual walking pace || id:ukb−b−4711'

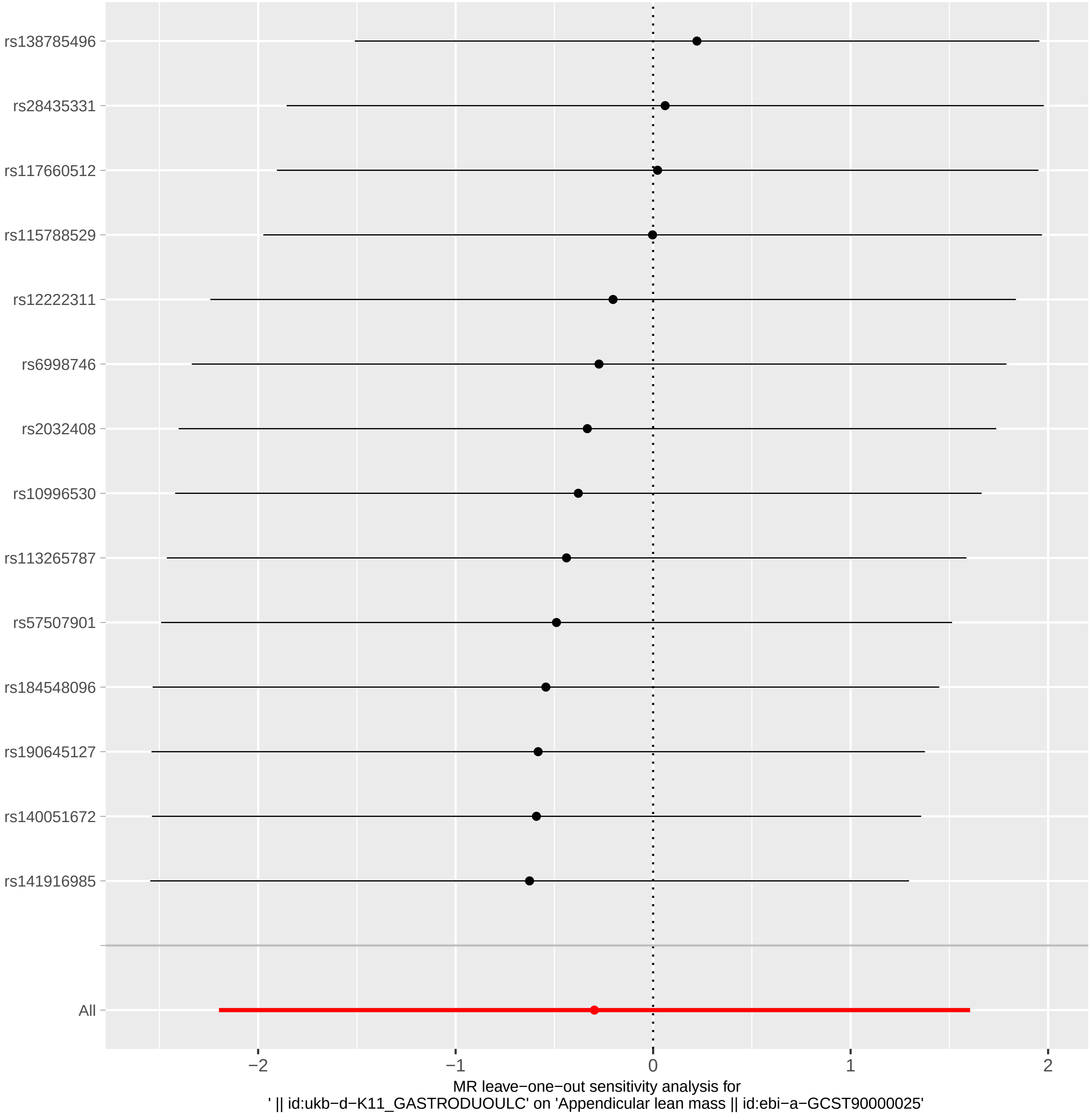
rs138785496
rs28435331
rs117660512
rs115788529
rs12222311
rs6998746
rs2032408
rs10996530
rs113265787
rs57507901
rs184548096
rs190645127
rs140051672
rs141916985
All
−2
−1
0
1
2
MR leave−one−out sensitivity analysis for
' || id:ukb−d−K11_GASTRODUOULC' on 'Appendicular lean mass || id:ebi−a−GCST90000025'

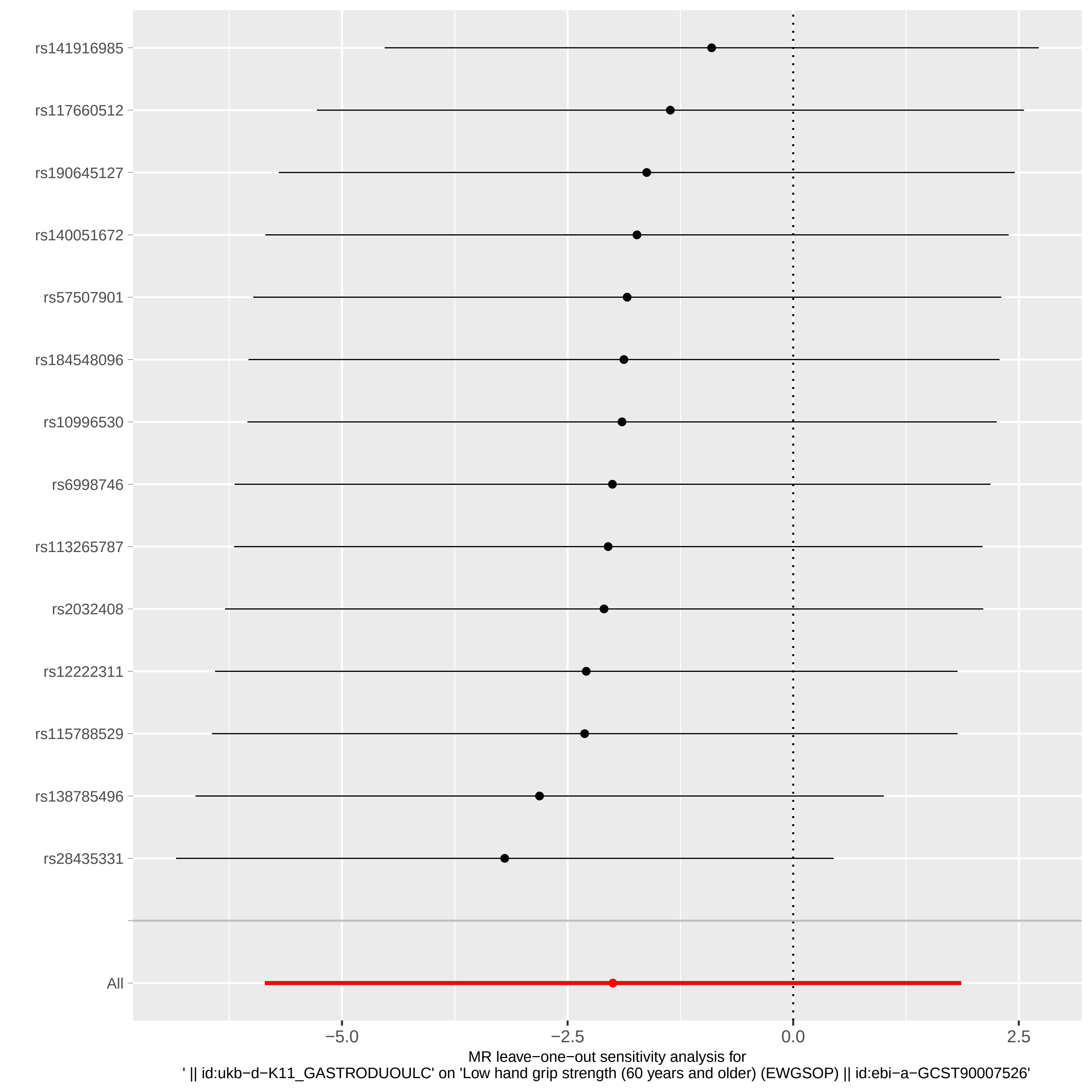

rs141916985
rs117660512
rs190645127
rs140051672
rs57507901
rs184548096
rs10996530
rs6998746
rs113265787
rs2032408
rs12222311
rs115788529
rs138785496
rs28435331
All
−5.0
−2.5
0.0
2.5
MR leave−one−out sensitivity analysis for
' || id:ukb−d−K11_GASTRODUOULC' on 'Low hand grip strength (60 years and older) (EWGSOP) || id:ebi−a−GCST90007526'

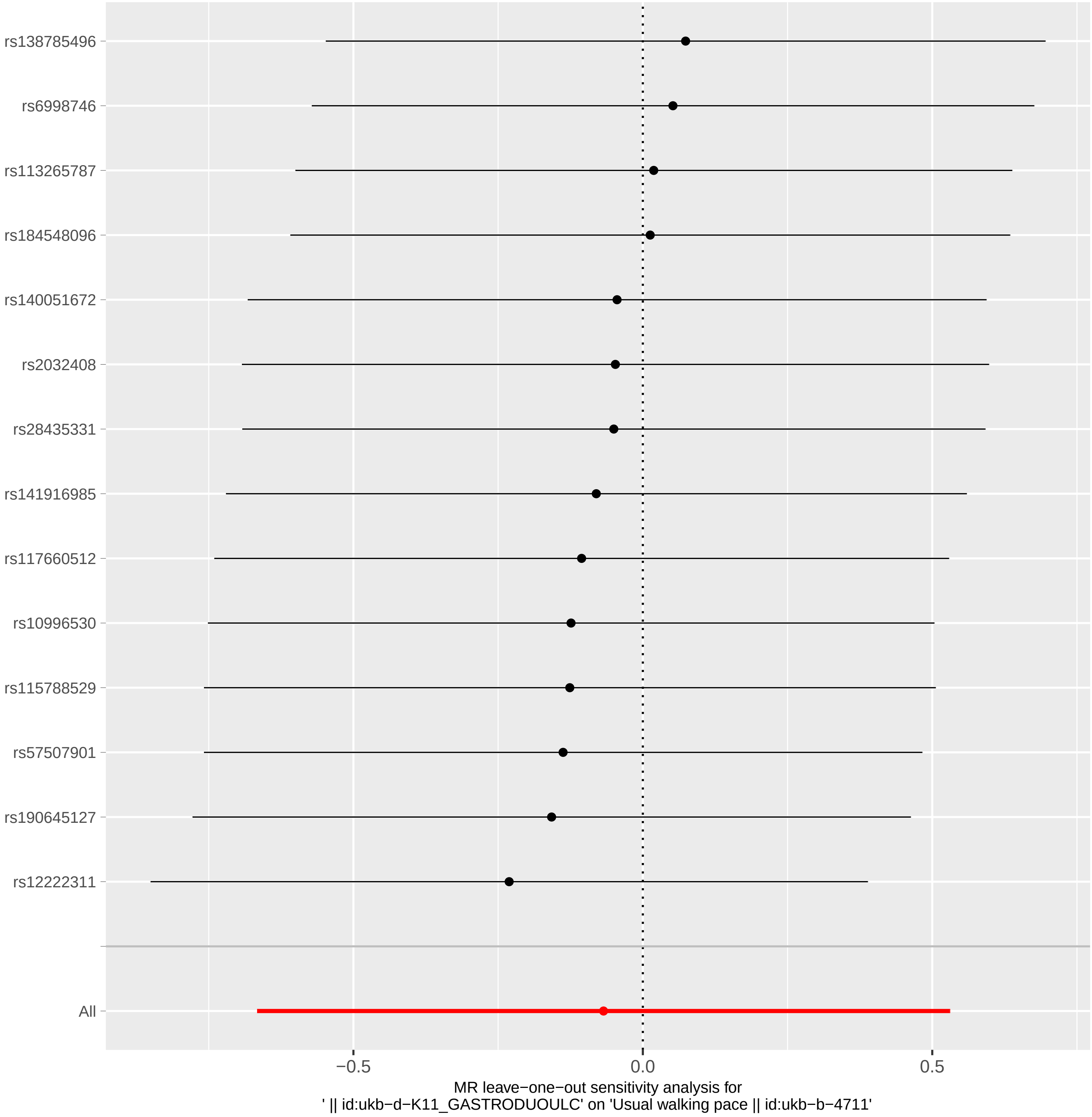
rs138785496
rs6998746
rs113265787
rs184548096
rs140051672
rs2032408
rs28435331
rs141916985
rs117660512
rs10996530
rs115788529
rs57507901
rs190645127
rs12222311
All
−0.5
0.0
0.5
MR leave−one−out sensitivity analysis for
' || id:ukb−d−K11_GASTRODUOULC' on 'Usual walking pace || id:ukb−b−4711'

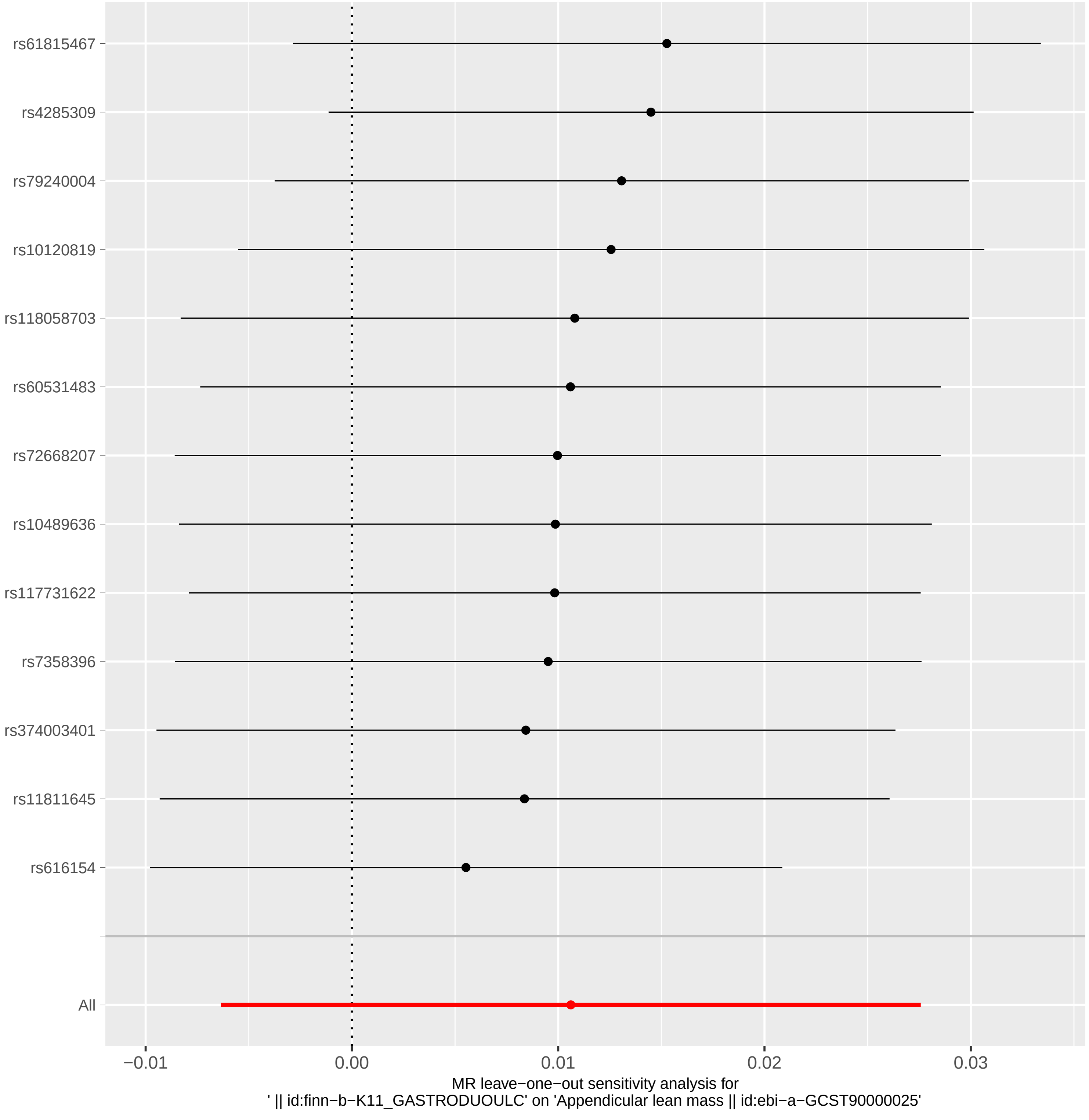
rs61815467
rs4285309
rs79240004
rs10120819
rs118058703
rs60531483
rs72668207
rs10489636
rs117731622
rs7358396
rs374003401
rs11811645
rs616154
All
−0.01
0.00
0.01
0.02
0.03
MR leave−one−out sensitivity analysis for
' || id:finn−b−K11_GASTRODUOULC' on 'Appendicular lean mass || id:ebi−a−GCST90000025'

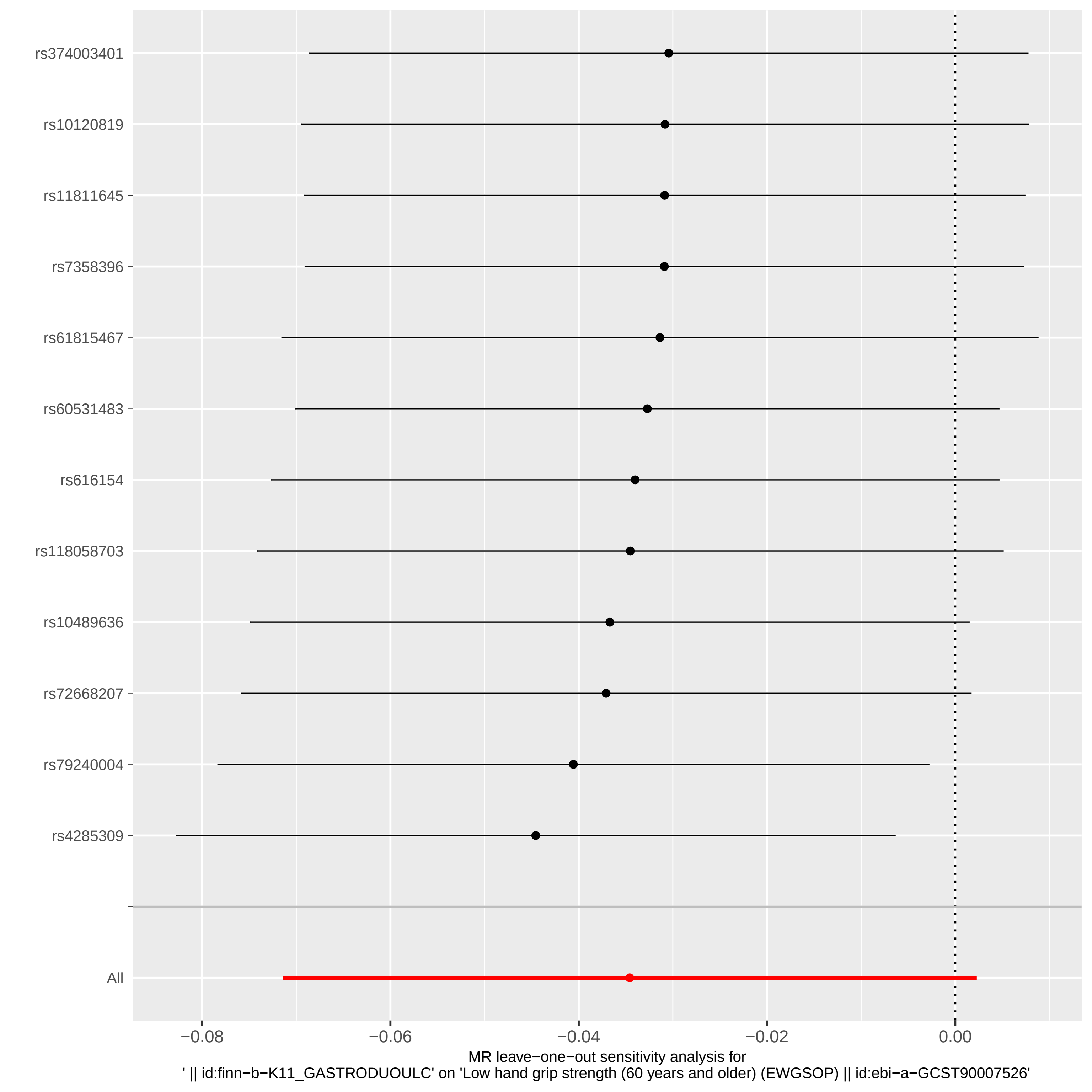

rs374003401
rs10120819
rs11811645
rs7358396
rs61815467
rs60531483
rs616154
rs118058703
rs10489636
rs72668207
rs79240004
rs4285309
All
−0.08
−0.06
−0.04
−0.02
0.00
MR leave−one−out sensitivity analysis for
' || id:finn−b−K11_GASTRODUOULC' on 'Low hand grip strength (60 years and older) (EWGSOP) || id:ebi−a−GCST90007526'

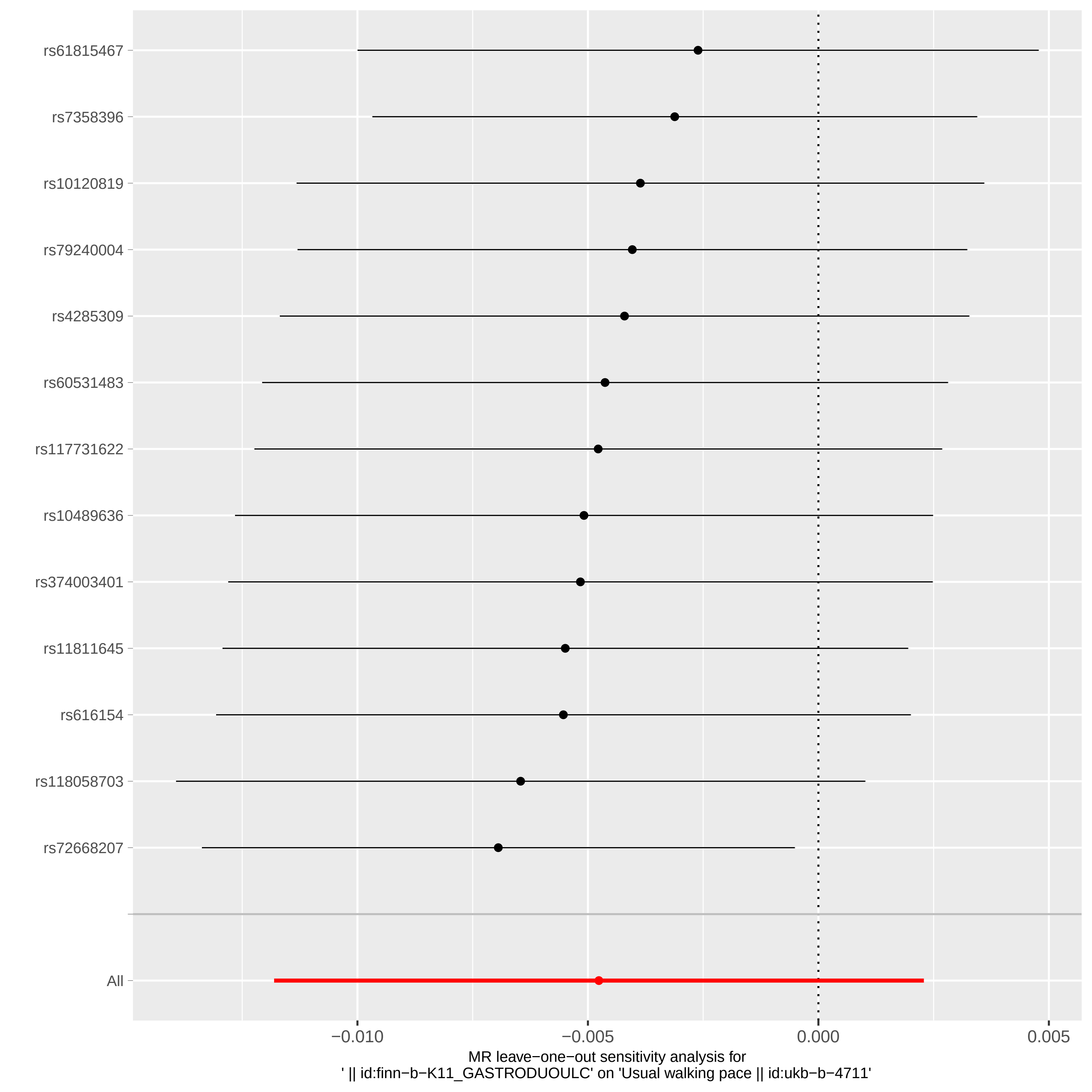
rs61815467
rs7358396
rs10120819
rs79240004
rs4285309
rs60531483
rs117731622
rs10489636
rs374003401
rs11811645
rs616154
rs118058703
rs72668207
All
−0.010
−0.005
0.000
0.005
MR leave−one−out sensitivity analysis for
' || id:finn−b−K11_GASTRODUOULC' on 'Usual walking pace || id:ukb−b−4711'

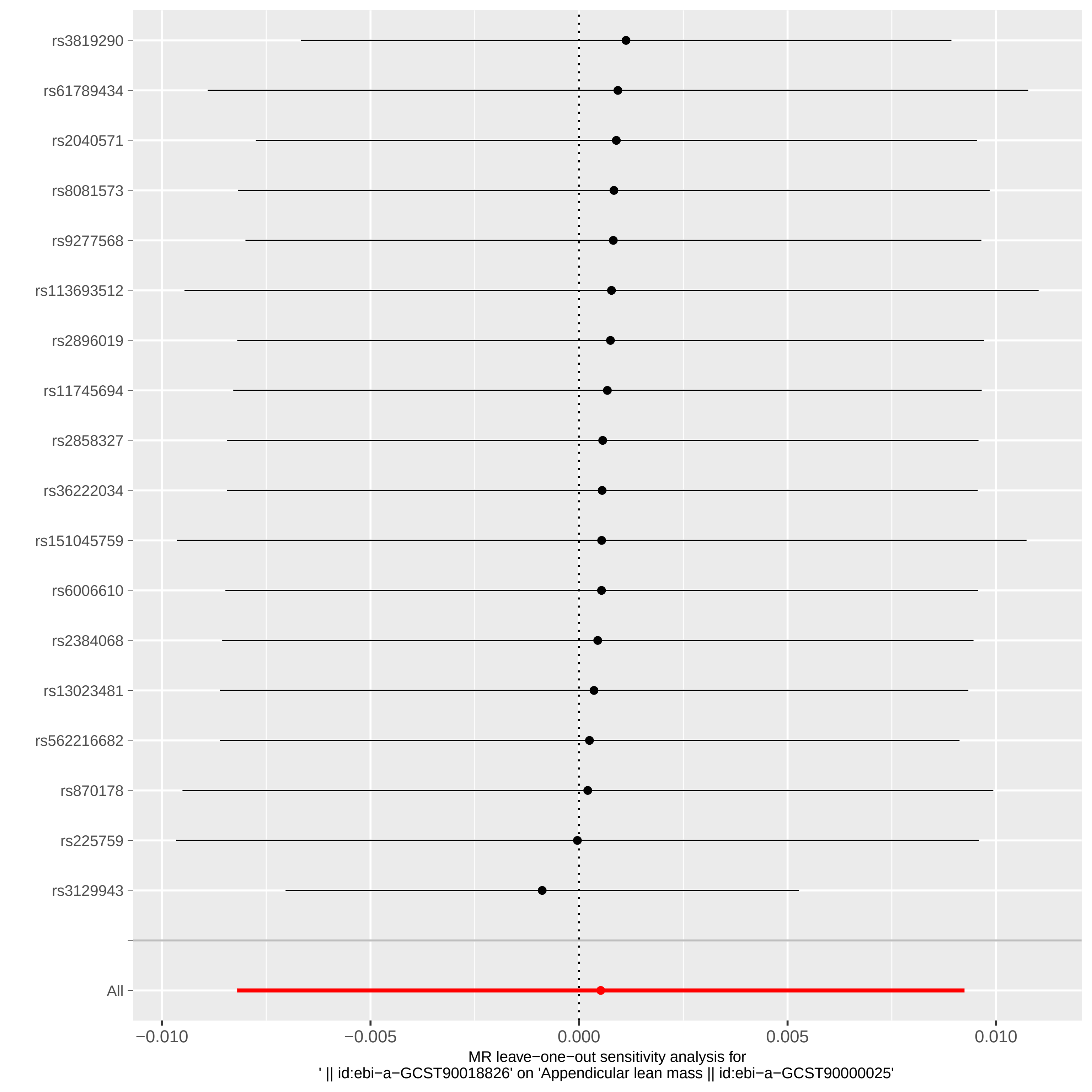
rs3819290
rs61789434
rs2040571
rs8081573
rs9277568
rs113693512
rs2896019
rs11745694
rs2858327
rs36222034
rs151045759
rs6006610
rs2384068
rs13023481
rs562216682
rs870178
rs225759
rs3129943
All
−0.010
−0.005
0.000
0.005
0.010
MR leave−one−out sensitivity analysis for
' || id:ebi−a−GCST90018826' on 'Appendicular lean mass || id:ebi−a−GCST90000025'

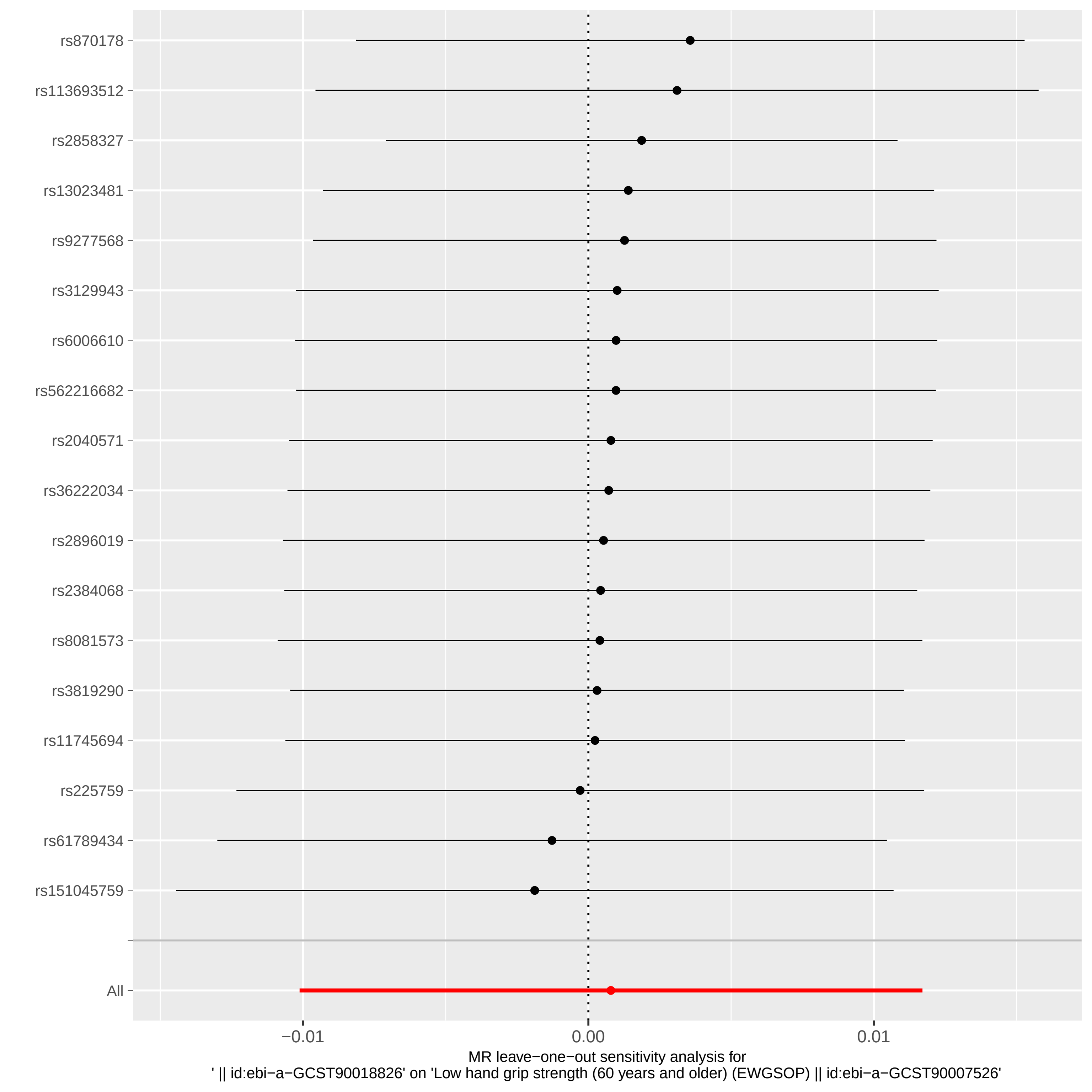
rs870178
rs113693512
rs2858327
rs13023481
rs9277568
rs3129943
rs6006610
rs562216682
rs2040571
rs36222034
rs2896019
rs2384068
rs8081573
rs3819290
rs11745694
rs225759
rs61789434
rs151045759
All
−0.01
0.00
0.01
MR leave−one−out sensitivity analysis for
' || id:ebi−a−GCST90018826' on 'Low hand grip strength (60 years and older) (EWGSOP) || id:ebi−a−GCST90007526'

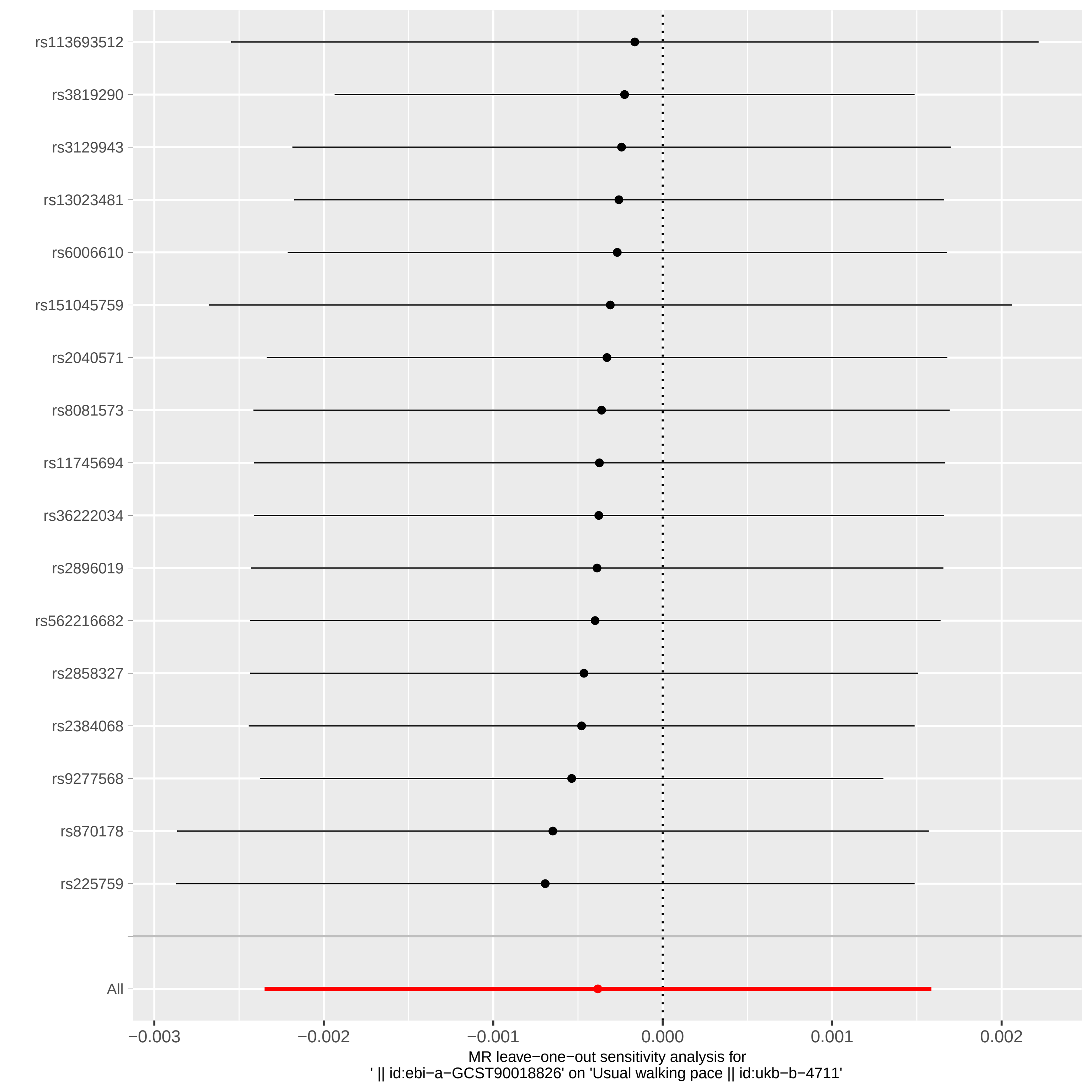

rs113693512
rs3819290
rs3129943
rs13023481
rs6006610
rs151045759
rs2040571
rs8081573
rs11745694
rs36222034
rs2896019
rs562216682
rs2858327
rs2384068
rs9277568
rs870178
rs225759
All
−0.003
−0.002
−0.001
0.000
0.001
0.002
MR leave−one−out sensitivity analysis for
' || id:ebi−a−GCST90018826' on 'Usual walking pace || id:ukb−b−4711'

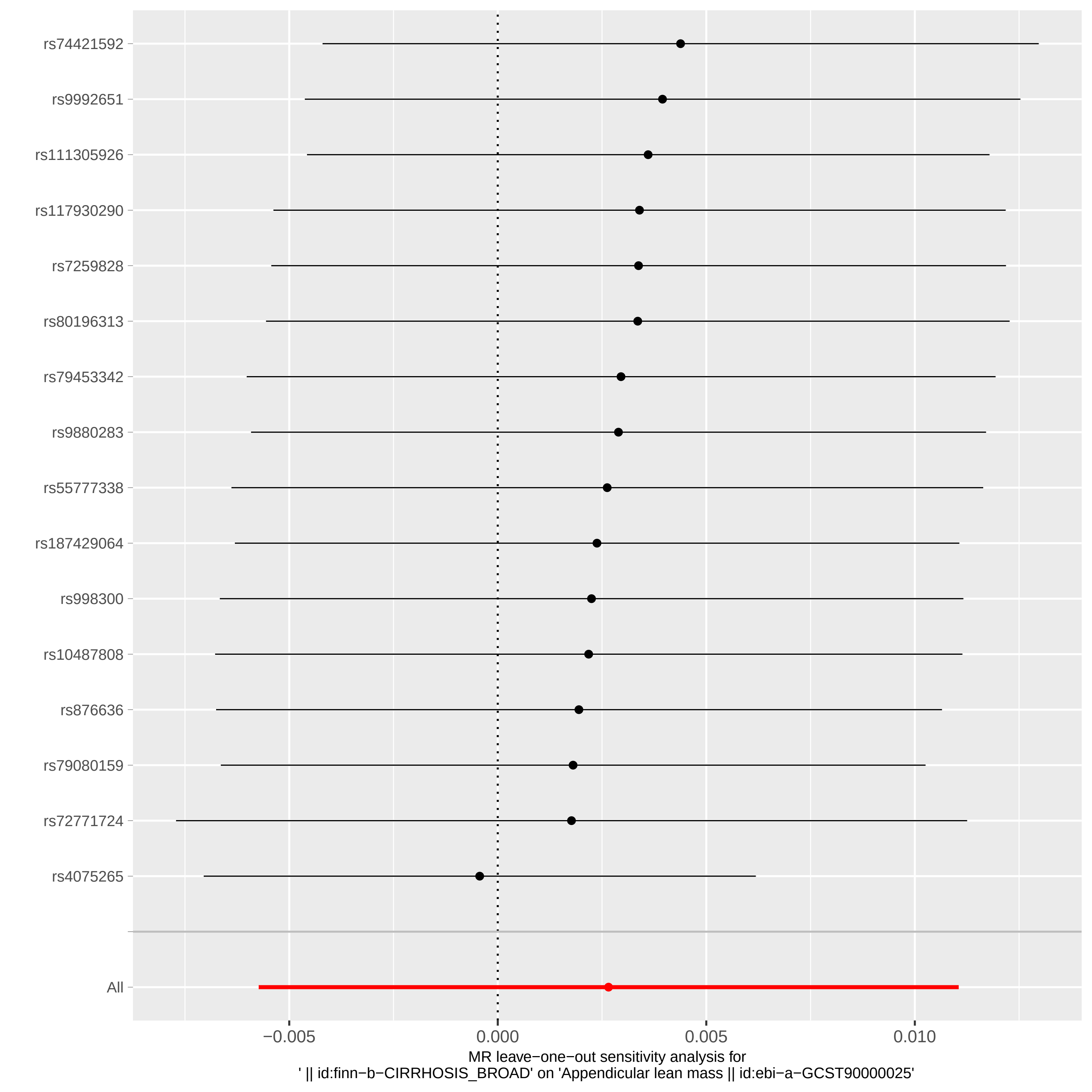
rs74421592
rs9992651
rs111305926
rs117930290
rs7259828
rs80196313
rs79453342
rs9880283
rs55777338
rs187429064
rs998300
rs10487808
rs876636
rs79080159
rs72771724
rs4075265
All
−0.005
0.000
0.005
0.010
MR leave−one−out sensitivity analysis for
' || id:finn−b−CIRRHOSIS_BROAD' on 'Appendicular lean mass || id:ebi−a−GCST90000025'

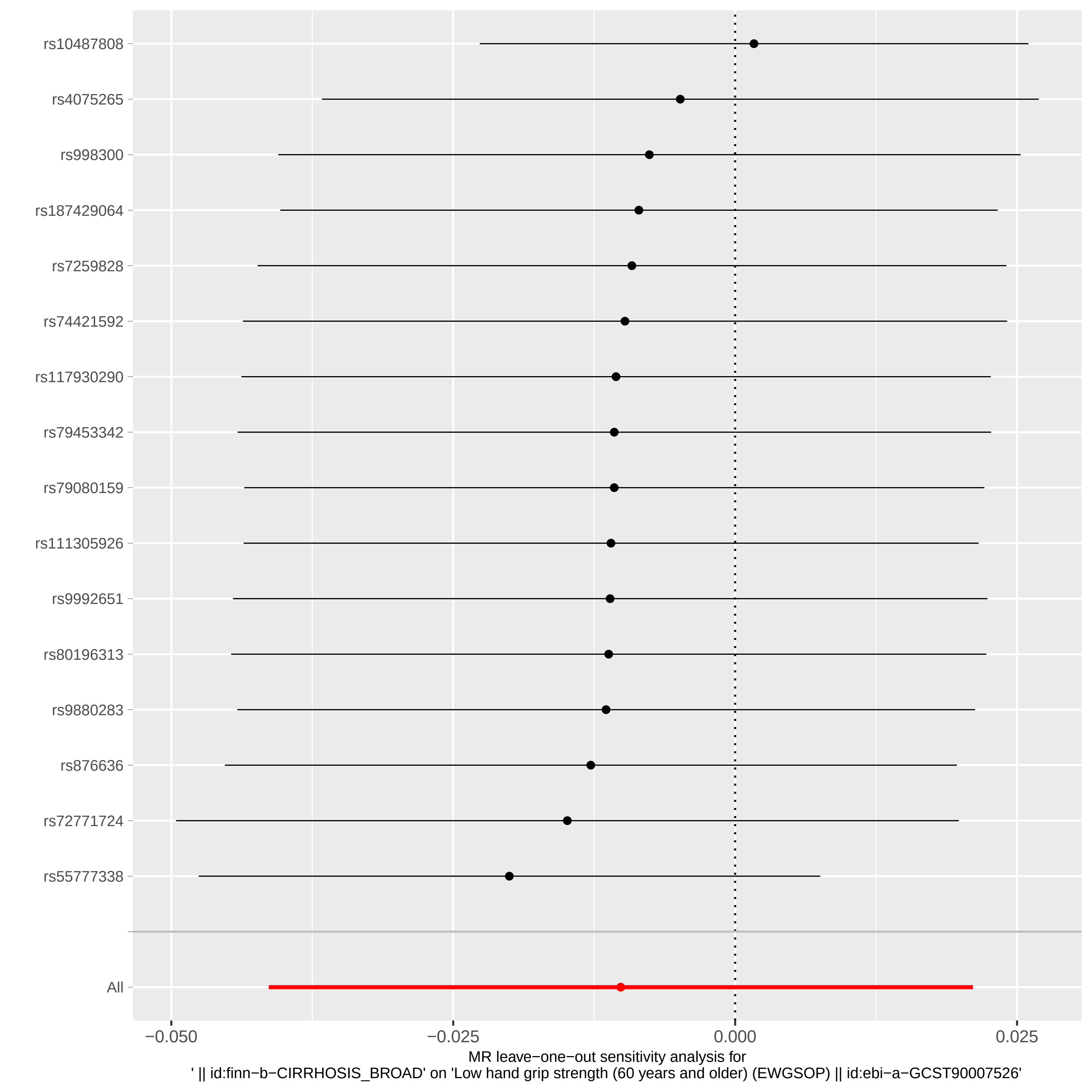

rs10487808
rs4075265
rs998300
rs187429064
rs7259828
rs74421592
rs117930290
rs79453342
rs79080159
rs111305926
rs9992651
rs80196313
rs9880283
rs876636
rs72771724
rs55777338
All
−0.050
−0.025
0.000
0.025
MR leave−one−out sensitivity analysis for
' || id:finn−b−CIRRHOSIS_BROAD' on 'Low hand grip strength (60 years and older) (EWGSOP) || id:ebi−a−GCST90007526'

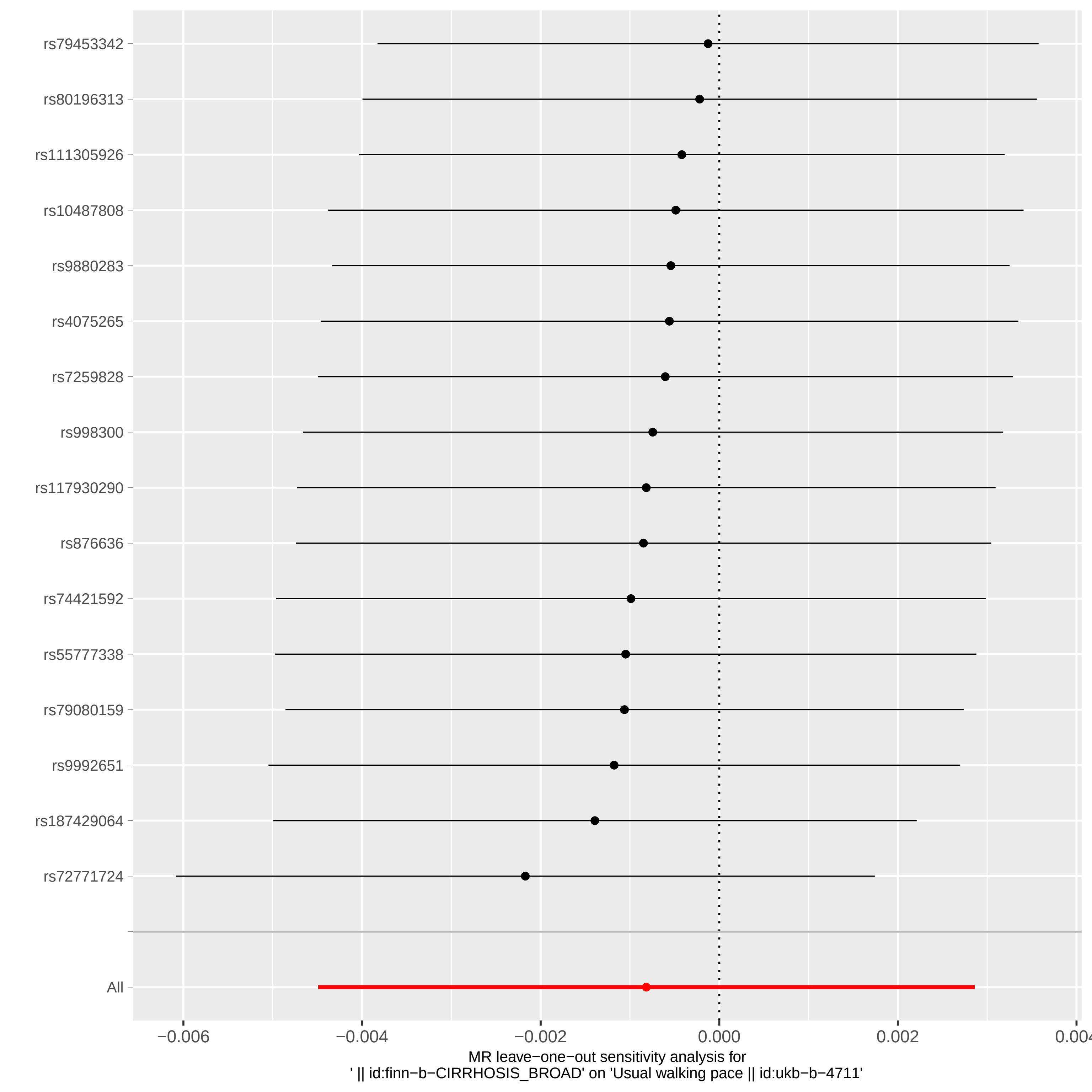

rs79453342
rs80196313
rs111305926
rs10487808
rs9880283
rs4075265
rs7259828
rs998300
rs117930290
rs876636
rs74421592
rs55777338
rs79080159
rs9992651
rs187429064
rs72771724
All
−0.006
−0.004
−0.002
0.000
0.002
0.004
MR leave−one−out sensitivity analysis for
' || id:finn−b−CIRRHOSIS_BROAD' on 'Usual walking pace || id:ukb−b−4711'
