# Supplementary material for: Sarcopenia-related traits and 10 digestive system disorders: insight from genetic correlation and Mendelian randomization
Source: Front Public Health. 2024 Jul 10;12:1412842. doi: 10.3389/fpubh.2024.1412842 (PMC11267997; doi:10.3389/fpubh.2024.1412842)
Supplement: Supplementary file 2 [file Table_1.DOCX]

Supplementary Material

# Supplementary Data

## Supplementary Figures

**
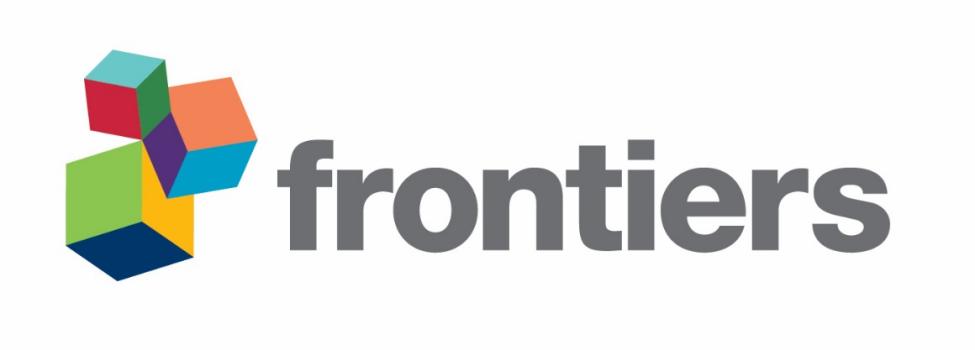
**

**Supplementary Figure 1.** The leave-one-out analysis reuslts

## Supplementary Tables

Supplementary Table S1:Comprehensive details on data sources for sarcopenia and 10 digestive system diseases;

Supplementary Table S2:The genetic correlation results;

Supplementary Table S3:The Mendelian Randomization analysis results;

Supplementary Table S4:The heterogeneity analyses results;

Supplementary Table S5:The pleiotropy analyses results;

Supplementary Table S6: The meta-analysis results;

Supplementary Table S7: The mediation analysis results
